# Isothiourea-catalysed enantioselective pyrrolizine synthesis: synthetic and computational studies†
†Electronic supplementary information (ESI) available: NMR spectra, HPLC analysis and computational co-ordinates. Data available.^12^ CCDC 1483759. For ESI and crystallographic data in CIF or other electronic format see DOI: 10.1039/c6ob01557c



**DOI:** 10.1039/c6ob01557c

**Published:** 2016-07-21

**Authors:** Daniel G. Stark, Patrick Williamson, Emma R. Gayner, Stefania F. Musolino, Ryan W. F. Kerr, James E. Taylor, Alexandra M. Z. Slawin, Timothy J. C. O'Riordan, Stuart A. Macgregor, Andrew D. Smith

**Affiliations:** a EaStCHEM , School of Chemistry , University of St Andrews , North Haugh , St Andrews , Fife KY16 9ST , UK . Email: ads10@st-andrews.ac.uk; b Institute of Chemical Sciences , Heriot-Watt University , Edinburgh , EH14 4AS , UK . Email: s.a.macgregor@hw.ac.uk; c Syngenta , Jealott's Hill International Research Centre , Bracknell , RG42 6EY , UK

## Abstract

The catalytic enantioselective synthesis of a range of *cis*-pyrrolizine carboxylate derivatives with outstanding stereocontrol (14 examples, >95 : 5 dr, >98 : 2 er) through an isothiourea-catalyzed intramolecular Michael addition-lactonisation and ring-opening approach from the corresponding enone acid is reported. An optimised and straightforward three-step synthetic route to the enone acid starting materials from readily available pyrrole-2-carboxaldehydes is delineated, with benzotetramisole (5 mol%) proving the optimal catalyst for the enantioselective process. Ring-opening of the pyrrolizine dihydropyranone products with either MeOH or a range of amines leads to the desired products in excellent yield and enantioselectivity. Computation has been used to probe the factors leading to high stereocontrol, with the formation of the observed *cis*-steroisomer predicted to be kinetically and thermodynamically favoured.

## Abstract 2

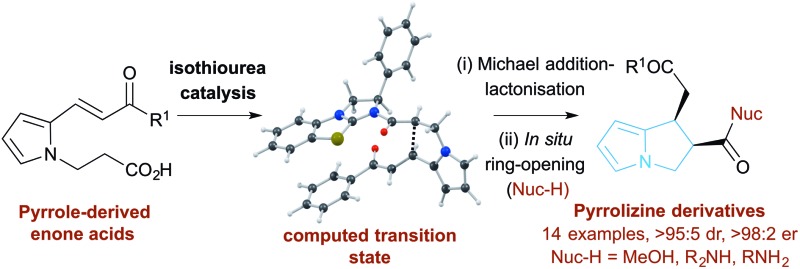
The isothiourea-catalysed enantioselective synthesis of *cis*-pyrrolizines is explored synthetically and computationally.

## Introduction

**Fig. 1:**
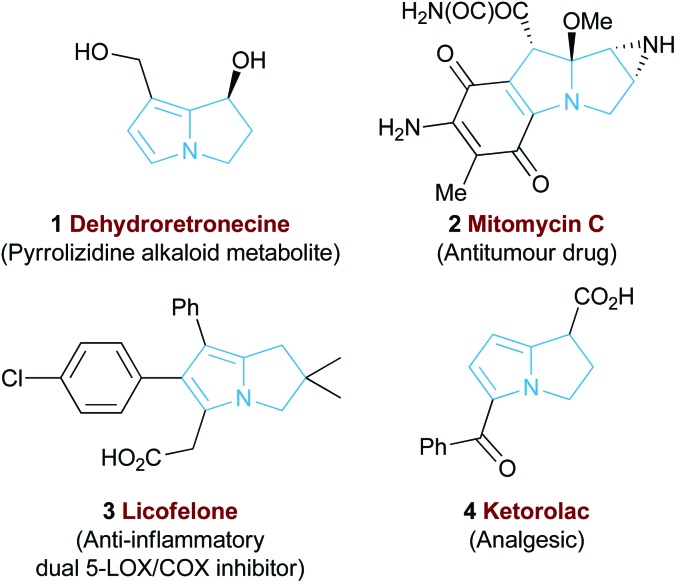
Representative biologically active pyrrolizines.

The 5,5-bicyclic pyrrolizine and pyrrolizidine structural motifs that contain a bridgehead nitrogen atom are present within the core of many biologically active natural products^1^ such as that of dehydroretronecine **1** (Fig. 1).^2^ This natural product, along with many other derivatives, originates from metabolism of pyrrolizidine alkaloids (PAs), a natural alkaloid prevalent in plant life throughout nature.^3^ Such PA-derived molecules are known to be potent heptatoxins,^4^ carcinogens,^5^ teratogens^6^ and genotoxins.^7^ Compelled by such levels of biological activity, many of these natural products and their derivatives have become commercial therapeutic agents. For example, the partially reduced pyrrolizine mitomycin C **2**, isolated from *Streptomyces caespitosus* or *Streptomyces lavendulae*, is a potent antitumour drug with a broad scope of applications.^8^ The non-steroidal anti-inflammatory drug (NSAID), Licofelone **3** has shown great promise in osteoarthritis treatment through a dual inhibition of 5-LOX/COX.^9^ Additionally, another pyrrolizine based NSAID, Ketorolac **4**, has found success as a commercial analgesic.^10^

**Fig. 2:**
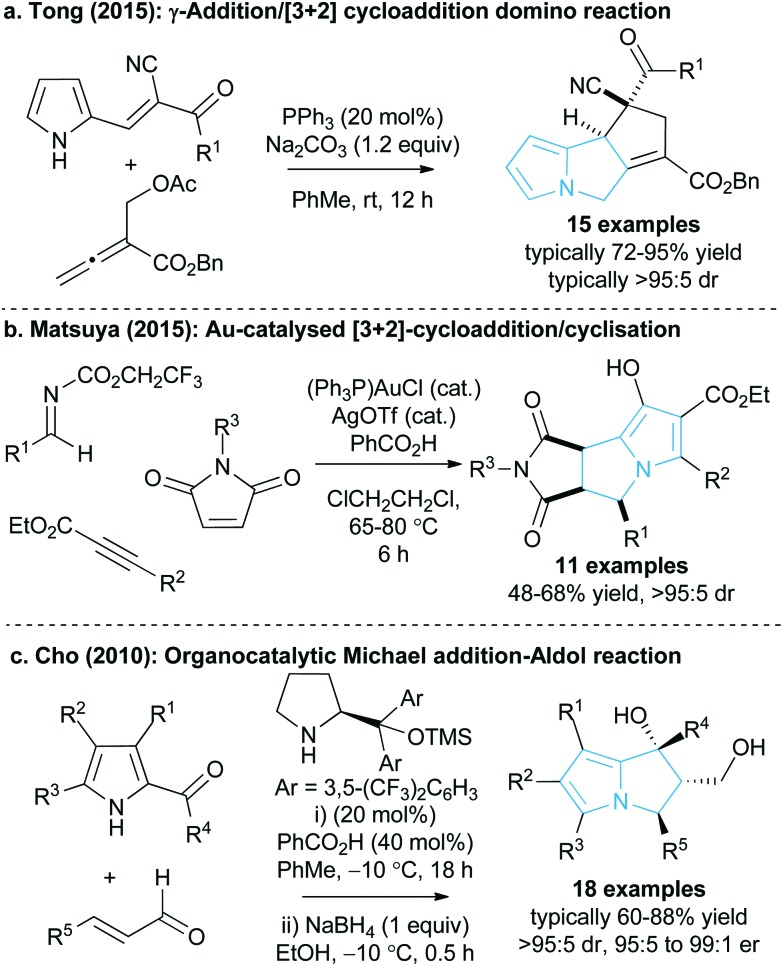
Current catalytic methods for pyrrolizine synthesis.

Given the value and potential of these bicyclic compounds, a variety of synthetic routes towards these motifs have been designed, with many syntheses involving classic total synthesis approaches towards specific target molecules.^11^ In recent years the state-of-the-art in catalytic pyrrolizine syntheses has involved diastereoselective multi-step reaction processes such as the phosphine-catalysed domino reaction developed by Tong and co-workers^13^ (Fig. 2a) or the gold-catalysed process by Matsuya and co-workers (Fig. 2b).^14^ Catalytic enantioselective methodologies that enable efficient access to this desirable structural motif are relatively limited. Within this area, Cho and co-workers showed that an enantioselective organocatalysed Michael addition-aldol approach could generate functionalised pyrrolizines with excellent diastereo- and enantiocontrol (18 examples, >95 : 5 dr and 95 : 5 to 99 : 1 er, Fig. 2c), although relatively high catalyst loadings were employed to promote this process.^15^ In spite of these advances there is still a clear requirement for easily accessible and reliable catalytic methodologies that can produce stereodefined chiral pyrrolizine derivatives with high levels of efficiency and enantiocontrol.

**Fig. 3:**
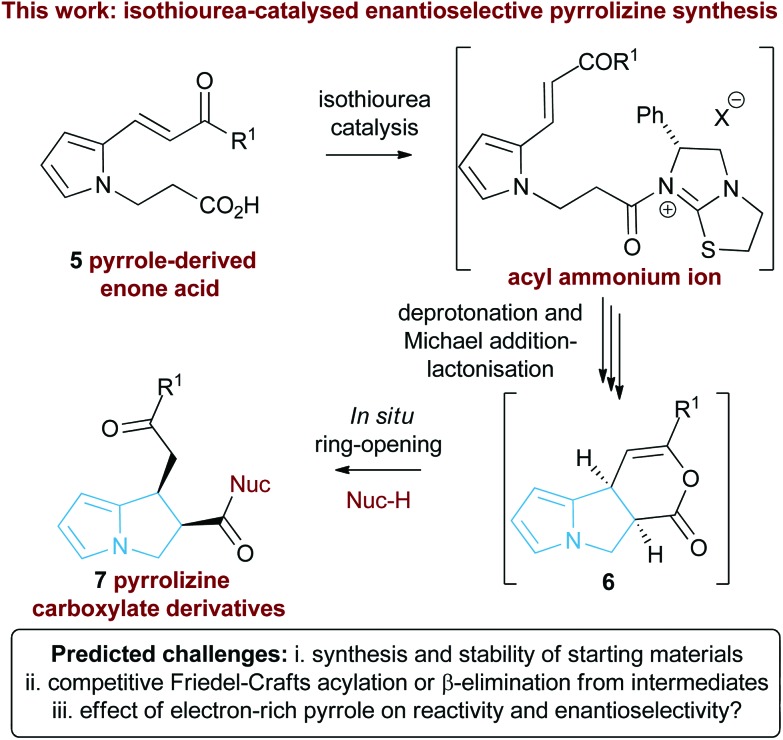
Proposed isothiourea-catalysed Michael addition-lactonisation methodology for pyrrolizine synthesis.

Following seminal work from Romo and co-workers using ammonium enolates generated from carboxylic acids,^16^ ourselves and others,^17^ have used isothioureas^18^ to catalyse a range of formal [2 + 2],^19^ [3 + 2]^20^ and [4 + 2]^21^ cycloaddition processes that employ an ammonium enolate intermediate.^22^ Related intramolecular Michael addition-lactonisation cascades from enone-acid substrates have been used to generate simple heterocyclic products such as THFs and pyrrolidines with excellent enantioselectivity.^23^ Building upon this previous work, the application of this strategy to construct the highly desirable pyrrolizine core in a catalytic enantioselective fashion starting from pyrrole-derived enone acid substrates such as **5** is investigated (Fig. 3). At the onset of these studies the main challenges were envisaged to arise from the incorporation of the electron-rich *N*-functionalised pyrrole core within the target enone-acid. A robust method to access this structural motif has not been reported previously, while the effect of incorporating this planar electron-rich aromatic structure upon stability and reactivity, as well as the conformational and steric effects upon diastereo- and enantioselectivity were unknown. Furthermore, the potential for competitive intramolecular Friedel–Crafts acylation of the pyrrole *via* a mixed anhydride or acyl ammonium ion intermediate,^24^ or alternatively β-elimination from an ammonium enolate, needed to be assessed. To further enhance our understanding of this process, we also wanted to probe the course of the proposed cascade cyclisation process *via* computation in order to understand the factors leading to stereocontrol. Very limited computational analysis of the use of isothiourea-derived ammonium enolates in catalysis has been reported.^25^ To date only the single report from Muck-Lichtenfeld and Studer concerning the intermolecular formal 1,3-dipolar cycloaddition of azomethine imines with mixed anhydrides under isothiourea catalysis has incorporated DFT analysis.^20*a*^

In this manuscript we report the realisation of this strategy to facilitate the catalytic enantioselective synthesis of these valuable heterocyclic products **6** and **7**. A straightforward three-step synthetic route to the enone acid starting materials from readily available pyrrole-2-carboxaldehydes is delineated, with commercially available benzotetramisole (BTM) proving the optimal catalyst for the enantioselective process. Furthermore, the use of computational analysis allows insight into the origin of stereocontrol in this intramolecular cascade process.

## Results and discussion

### Substrate synthesis

**Scheme 1:**
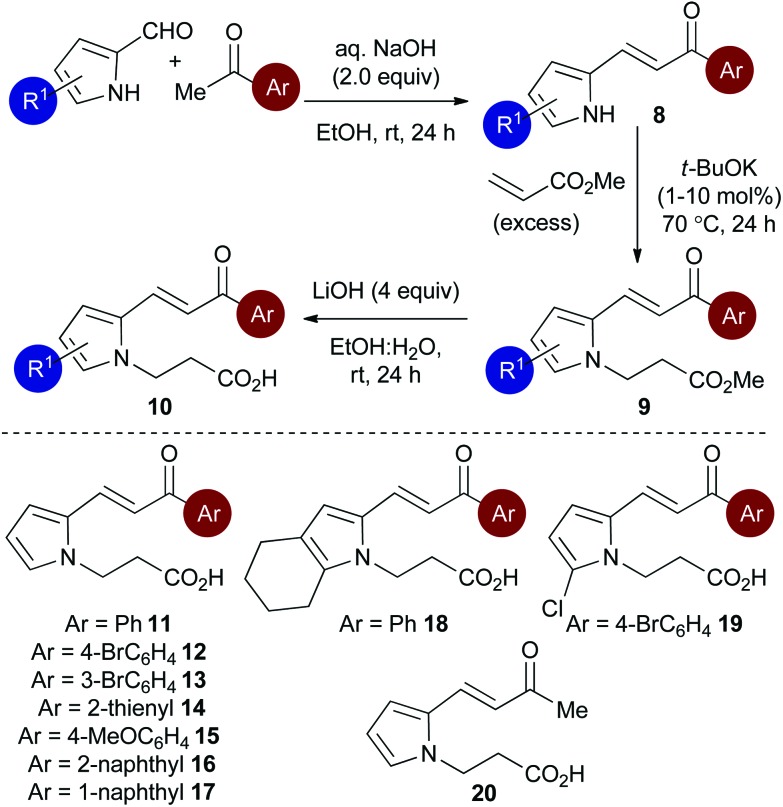
Optimised synthesis of pyrrole-derived enone-acid substrates.

Initial studies set out to devise a practical synthetic route towards the target pyrrole-derived enone-acid substrates. Substrate **11** was identified as a model system, and was initially prepared from pyrrole 2-carboxaldehyde by *N*-alkylation, ester hydrolysis and Wittig olefination. However, attempted synthesis of a range of pyrrole-derived enone-acid substrates or scale-up of this synthetic route proved low yielding and irreproducible when directed towards alternative substrates. After a thorough investigation into alternative synthetic approaches, a reliable three-step sequence starting from the corresponding pyrrole 2-carboxaldehyde was established (Scheme 1). Aldol-condensation with the requisite ketone provides a general and chromatography-free preparation of pyrrole-enones **8**. Treatment with sub-stoichiometric *t*-BuOK and methyl acrylate gives the *N*-alkylation product **9**, with subsequent ester hydrolysis providing the desired enone-acid substrates **10** in good overall yield with a wide scope available. This reliable synthetic sequence allowed the preparation of a range of substrates **11–18** in up to 58% yield over three steps and on multigram scale, and allows for substituent variation within both the pyrrole and enone components.^26^ Direct chlorination of **11** with *N*-chlorosuccinimide (NCS) led to substrate **19**, while an analogous procedure using acetone for the aldol reaction procedure, followed by alkylation and ester hydrolysis gave methyl enone **20**.

### Reaction optimisation

With a reliable synthetic route to pyrrole-derived enone acid substrates in hand, the feasibility and subsequent optimisation of the isothiourea-catalysed Michael addition-lactonisation protocol was conducted (Table 1). Utilising achiral isothiourea DHPB **22**, *i*-Pr_2_NEt and *t*-BuCOCl provided the desired pyrrolizine dihydropyranone **21** from enone-acid **11** in a moderate 41% isolated yield but with excellent >95 : 5 dr (entry 1). Alternative methods for *in situ* generation of a reactive carboxylate derivative, such as Mukaiyama's reagent **26**, did not improve conversion or isolated yield (entry 2). A significant improvement in yield was observed when the equivalents of both *t*-BuCOCl and *i*-Pr_2_NEt were increased from 1.5 to 3.0 equiv., with **21** obtained in 84% yield. Subsequent studies assessed the feasibility of an enantioselective process, with chiral isothiourea catalysts **23–25** examined. Tetramisole·HCl **23** and benzotetramisole (BTM) **24**-mediated reactions (entries 4 and 5) provided **21** in excellent yield, >95 : 5 dr and with >99 : 1 er. Application of HyperBTM **25** proved marginally less effective, with the product **21** obtained in high yield and dr but with reduced 96.5 : 3.5 er (entry 6). Notably in all of these catalytic processes no competitive products arising from either Friedel–Crafts acylation or β-elimination were observed, despite the proposed intermediacy of an acyl ammonium ion. The effect of reduced catalyst loading using BTM **24** and tetramisole·HCl **23** was next evaluated. Using 5 mol% BTM **24** maintained excellent levels of diastereo- and enantioselectivity, giving product **21** in 82% yield, while using 5 mol% tetramisole·HCl **23** led to reduced but still acceptable 67% yield (entries 7 and 8). Further reduction of the catalyst loading to 1 mol% showed the same trend in reactivity, with BTM-**24** giving **21** in 42% isolated yield, and tetramisole·HCl **23** a lower 34% yield, yet still in high dr and er (entries 9 and 10). All further studies used BTM **24** (5 mol%) as the optimal reaction conditions for this enantioselective process.

**Table 1:** Enantioselective Michael addition-lactonisation optimisation

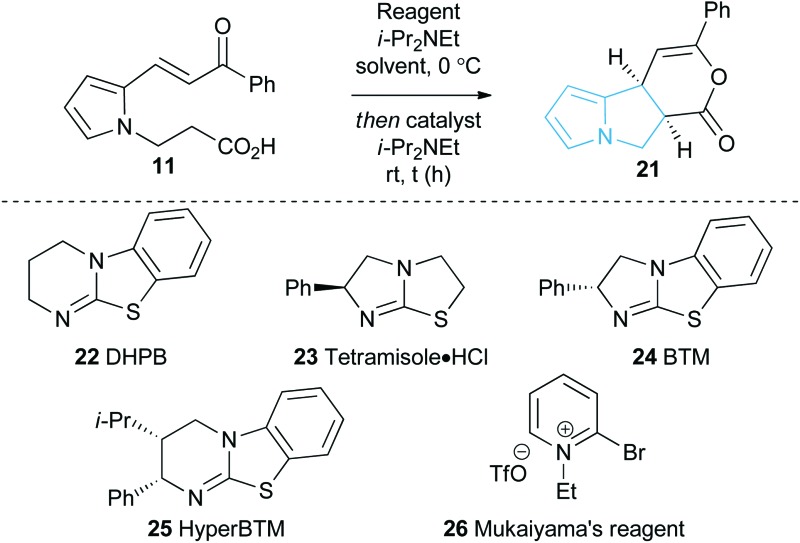					
Entry	Reagent^*a*^ (equiv.)	Catalyst (mol%)	Yield^*b*^ (%)	dr^*c*^	er^*d*^
1	*t*-BuCOCl (1.5)	**22** (10)	41	>95 : 5	—
2	**26** (1.5)	**22** (10)	45	>95 : 5	—
3	*t*-BuCOCl (3.0)	**22** (10)	84	>95 : 5	—
4	*t*-BuCOCl (3.0)	**23** (10)	84	>95 : 5	>99 : 1(*ent*)
5	*t*-BuCOCl (3.0)	**24** (10)	81	>95 : 5	>99 : 1
6	*t*-BuCOCl (3.0)	**25** (10)	81	>95 : 5	96.5 : 3.5
7	*t*-BuCOCl (3.0)	**24** (5)	82	>95 : 5	>99 : 1
8	*t*-BuCOCl (3.0)	**23** (5)	67	>95 : 5	>99 : 1(*ent*)
9	*t*-BuCOCl (3.0)	**24** (1)	42	>95 : 5	>99 : 1
10	*t*-BuCOCl (3.0)	**23** (1)	34	>95 : 5	99 : 1(*ent*)

^*a*^Applied in 1 : 1 ratio with *i*-Pr_2_NEt.

^*b*^Isolated yield.

^*c*^Determined by ^1^H NMR of the crude reaction product.

^*d*^Determined by chiral HPLC.

As an alternative strategy to the isolation of dihydropyranone **21** *in situ* ring-opening with a suitable nucleophile was investigated to provide access to pyrrolizine carboxylate derivatives (Table 2). Taking pyrrole-derived enone-acid **11** under the optimum catalysis conditions the resulting dihydropyranone **21** can be readily ring-opened *in situ* with MeOH giving pyrrolizine methyl ester **27** in excellent 86% yield, >95 : 5 dr and >99 : 1 er. Ring-opening using both primary and secondary amines to give pyrrolizine amides also proved successful. For example, use of allylamine provided **28** in 81% yield, >95 : 5 dr and 98.5 : 1.5 er, while ring-opening with pyrrolidine gave pyrrolizine amide **29** in quantitative yield, >95 : 5 dr and >99 : 1 er. Morpholine, *N*-Boc piperazine and tetrahydrothieno[3,2-*c*]pyridine could also be utilised, giving the corresponding pyrrolizine amides **30–32** in good to excellent yield (60–75%), >95 : 5 dr and >99 : 1 er. Upon standing the pyrrolizine carboxylate products proved considerably more stable to storage than the dihydropyranone **21**, therefore ring-opening with MeOH was used as the general procedure when exploring further substrate scope.

**Table 2:** Michael addition-lactonisation/ring-opening protocol^*a*^ ^,^ ^*b*^ ^,^ ^*c*^

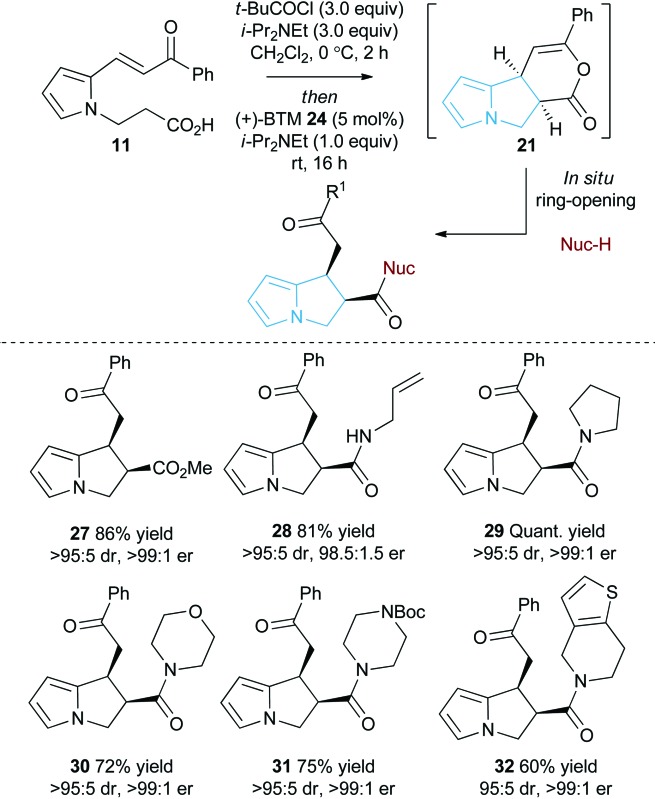

^*a*^Isolated yield.

^*b*^dr determined by ^1^H NMR of the crude reaction product.

^*c*^er determined by chiral HPLC.

### Substrate scope: Michael addition-lactonisation/methanolysis

With a reliable synthetic route to pyrrole-derived enone acid substrates available, the generality of the isothiourea catalysis using BTM **24** as the catalyst and MeOH as the nucleophile for *in situ* ring-opening was evaluated (Table 3). Variation of the enone-substituent of the substrate was first investigated with a range of aryl groups accommodated. Brominated aryl units can be included to give **33** and **34** in 78% and 74% yield, respectively, with both generated as single diastereoisomers in >99 : 1 er. Incorporation of the heterocyclic 2-thienyl substituent was readily tolerated giving **35** in 77% yield, >95 : 5 dr and 99 : 1 er. Electron-rich groups (4-MeOC_6_H_4_) can be installed, giving **36** in excellent 93% yield, >95 : 5 dr and >99 : 1 er.

**Table 3:** Michael addition-lactonisation/methanolysis scope: variation of Michael acceptor^*a*^ ^,^ ^*b*^ ^,^ ^*c*^

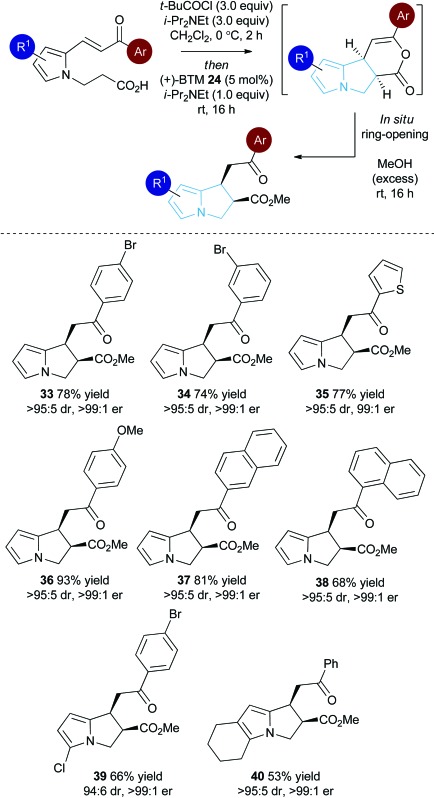

^*a*^Isolated yield.

^*b*^dr determined by ^1^H NMR of the crude reaction product.

^*c*^er determined by chiral HPLC.

**Fig. 4:**
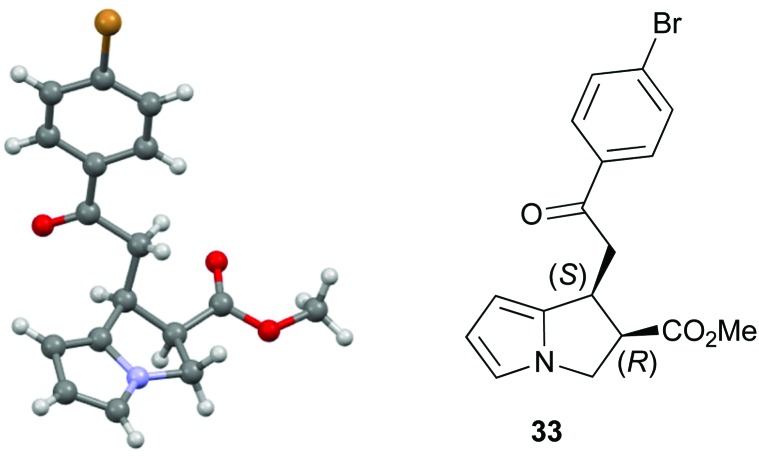
Molecular representation of the X-ray structure of **33**.

Both 1-naphthyl and 2-naphthyl units can also be incorporated to access the corresponding products **37** and **38** in good yields and excellent enantioselectivity, although attempted catalysis using Me-enone **20** did not result in any conversion to product. Variation within the pyrrole core of the pyrrolizine carboxylate product was also assessed. Chlorinated product **39** was produced in 66% yield with good 94 : 6 dr and as a single enantiomer (>99 : 1 er). The core motif can be expanded to the hexahydro-1*H*-pyrroloindole structure with the corresponding product **40** achieved in 53% yield and excellent stereoselectivity (>95 : 5 dr and >99 : 1 er).^27^ The reaction to form **33** was readily carried out on a 1 gram scale, giving the desired product in 67% yield in >95 : 5 dr and >99 : 1 er. The relative and absolute configuration within **33** was assigned by X-ray crystallography analysis, with the configuration within all other products assigned by analogy (Fig. 4).^28^

### Product derivatisation

**Scheme 2:**
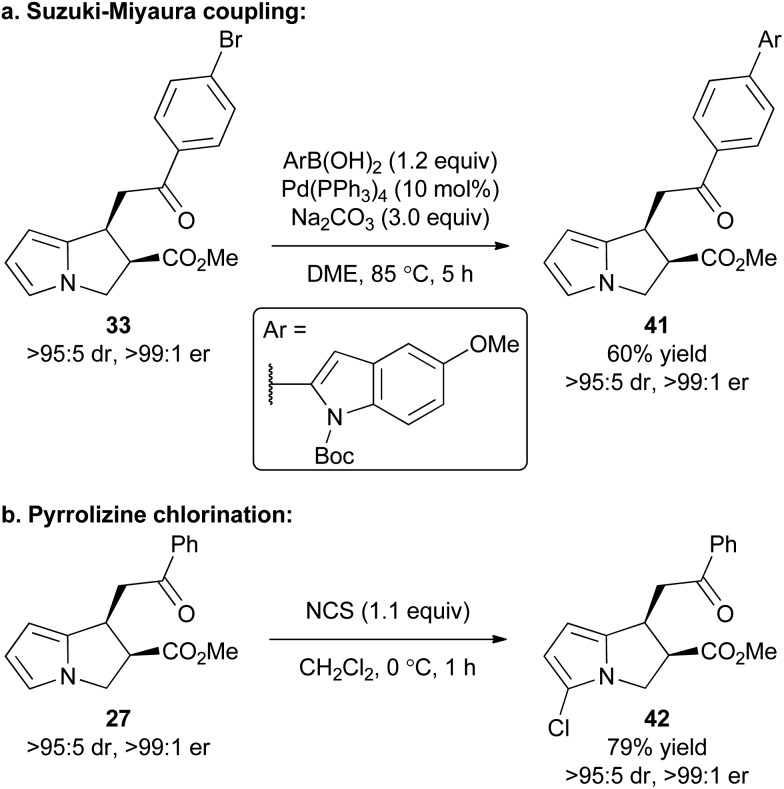
Product derivatisation.

To exemplify that this methodology can potentially be used as a basis for further synthetic elaboration, product derivatisation within both the pyrrole core and aromatic ketone substituent was explored (Scheme 2). For example, the synthesis of product **33** using this cascade methodology gives a product with the bromine functional handle. Through the use of a Suzuki–Miyaura coupling **33** was readily elaborated to access the polyheteroaromatic pyrrolizine **41** in 60% isolated yield and with no loss of stereointegrity. Alternatively, a simple chlorination of the pyrrole core within **27** was conducted using NCS to access chloropyrrolizine **42** in 79% yield.

### Proposed mechanism and computational studies

**Scheme 3:**
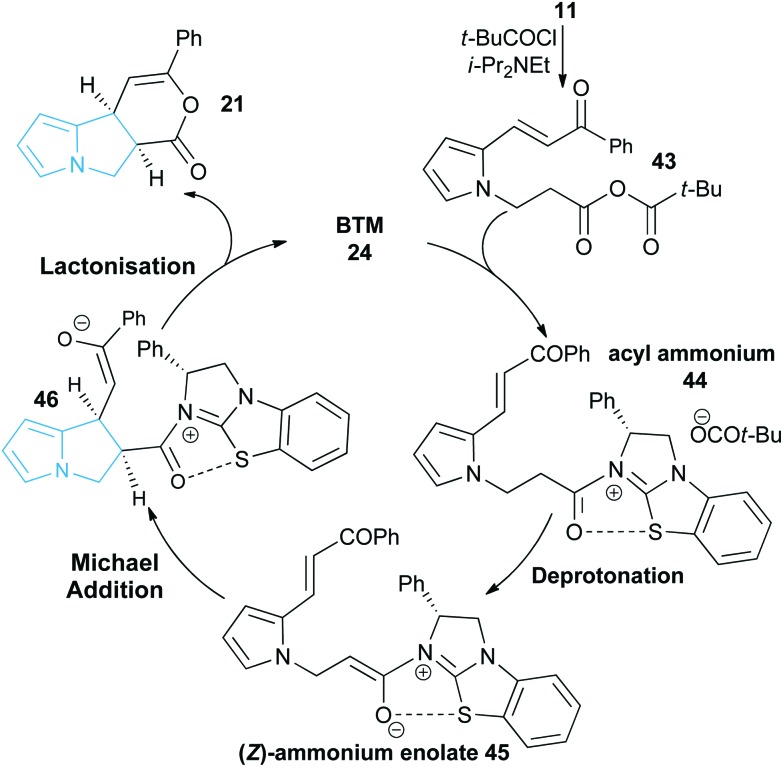
Proposed mechanism of isothiourea-catalysed Michael addition-lactonisation.

A proposed catalytic cycle for the synthesis of pyrrolizine dihydropyranone **21** from enone-acid **11** is shown in Scheme 3. Firstly, pivaloyl chloride reacts with the enone-acid to form mixed anhydride **43**, with subsequent nucleophilic attack from the Lewis base catalyst BTM **24** generating acyl ammonium ion **44**. Deprotonation to form (*Z*)-ammonium enolate **45**, followed by intramolecular Michael-addition forms the new C–C bond and two stereocentres in the initial cyclisation step. Intermediate **45** is believed to be stabilised by a favourable non-bonding or electrostatic O to S interaction and this has been investigated computationally (*vide infra*). Subsequent lactonisation releases catalyst **24** and the polycyclic dihydropyranone product **21**, which can be ring-opened upon addition of a nucleophilic amine or alcohol to give the isolated pyrrolizine carboxylate derivative.

On the basis of this mechanistic hypothesis, the origin of the diastereo- and enantioselective formation of the pyrrolizine dihydropyranone products in this BTM **24**-catalysed cascade process was further probed through density functional theory (DFT) calculations. Calculations employed the M06-2X functional and investigated the cyclisation of the parent enone-acid **21** reacting in the presence of **24** in dichloromethane solvent from either the (*E*)- or (*Z*)-form of the ammonium enolate. Full computational details, including results obtained with the B3LYP functional, are supplied in the ESI.† ^29^

**Fig. 5:**
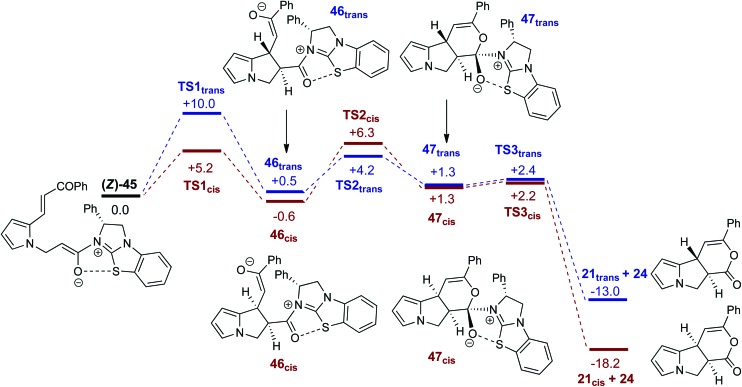
Computed free energy profiles (M06-2X(CH_2_Cl_2_), kcal mol^–1^) for the formation of *cis*- and *trans*-pyrrolizine dihydropyranones **21** from (*Z*)-ammonium enolate (*Z*)-**45**.

Fig. 5 shows computed profiles for the alternative reactions of the (*Z*)-ammonium enolate, (*Z*)-**45**, to form the *cis*- and *trans*-isomers of pyrrolizine dihydropyranone **21**. From (*Z*)-**45** two low energy transition states were located for the Michael cyclisation step: **TS1_*cis*_** at +5.2 kcal mol^–1^ and **TS1_*trans*_** at +10.0 kcal mol^–1^. The onward reaction *via* **TS1_*cis*_** (highlighted in red) leads to **46_*cis*_** at –0.6 kcal mol^–1^ in which the newly formed C4–C9 bond is rather long at 1.62 Å. Attack of the enolate oxygen then permits formation of a zwitterionic tetrahedral dihydropyran intermediate **47_*cis*_** *via* **TS2_*cis*_** at +6.3 kcal mol^–1^. Facile dissociation of BTM **24** from **47_*cis*_** *via* **TS3_*cis*_** forms **21_*cis*_** at –18.2 kcal mol^–1^. An analogous series of steps (highlighted in blue) was also characterized for the formation of **21_*trans*_** at –13.0 kcal mol^–1^. Formation of **21_*cis*_** is therefore both thermodynamically and kinetically favoured, as both **TS1_*cis*_** and **TS2_*cis*_** are significantly lower than **TS1_*trans*_** *en route* to **21_*trans*_**. A third pathway starting from the (*E*)-ammonium enolate precursor, (*E*)-**45** (+3.7 kcal mol^–1^) and leading to the enantiomeric-*trans* product was also characterised, but has a significantly higher barrier of 16.7 kcal mol^–1^ and can therefore be discounted (see ESI†).

It is notable that all the computed structures for both reaction pathways in Fig. 5 from enolate (*Z*)-**45** up to the final BTM dissociation steps feature a co-planar [1,5]-S···O motif with [1,5]-S···O interatom distances ranging from 2.60 Å to 2.82 Å. The importance of non-bonding S···O interactions has been widely recognized in structural and medicinal chemistry in the solid state (often described as a stabilising *n*_O_ to 
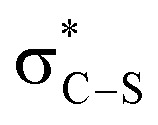
 interaction),^30^ and has been previously recognised as a controlling element in enantioselective isothiourea-catalyzed reaction processes.^31^ Although the origin of this interaction is still under debate,^32^ in all calculated structures the [1,5]-S···O distance is considerably below the sum of the van der Waals radii. Interestingly, the shortest distances calculated are found within (*Z*)-**45** (2.64 Å) and zwitterionic intermediates (**47_*cis*_** 2.60 Å and **47_*trans*_** 2.64 Å), presumably reflecting the formal negative charge on the oxygen atom within these structures. However, as this feature appears within all the structures in the computed reaction profiles, and varies in a similar way along those profiles, it does not appear to be a discriminating factor between the two pathways in Fig. 5.

**Fig. 6:**
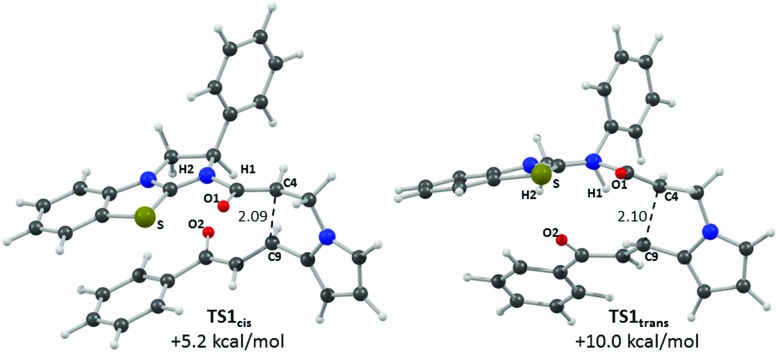
Computed selectivity determining transition states **TS1_*cis*_** and **TS1_*trans*_** with key atom labels and the forming C4···C9 distance highlighted in Å.

Computed structures for the selectivity-determining transition states **TS1_*cis*_** and **TS1_*trans*_** are compared in Fig. 6. Both structures have similar distances (2.09 and 2.10 Å) for the forming C4···C9 bond, with Michael addition occurring *anti*-to the stereodirecting phenyl substituent and thus accounting for the observed enantioselectivity. In the favoured **TS1_*cis*_** arrangement the prostereogenic centres adopt an approximately eclipsed conformation with a H–C4···C9–H dihedral of 3°, while in **TS1_*trans*_** the corresponding H–C4···C9–H dihedral is 149.5°. Both transition states also exhibit short and approximately co-planar [1,5]-S···O1 contacts of *ca*. 2.7 Å between the BTM sulfur atom and the enolate oxygen. The near-parallel arrangement of the BTM moiety and the enone aryl substituent in **TS1_*cis*_** (inter-plane angle = 5.7°) appears set up for stabilising π-stacking interactions that should be captured by the M06-2X functional.^29^ Indeed with the B3LYP functional (where such dispersion effects are not treated) the equivalent inter-plane angle = 28.1° and the preference for **TS1_*cis*_** is reduced to only 1.4 kcal mol^–1^ (see ESI†). Also noticeable in **TS1_*cis*_** are two short contacts between the forming oxy-anion on the enone (O2) and two C–H hydrogens upon the positively charged isothiouronium core (O1···H1 2.19 Å and O1···H2 2.37 Å). The enhanced acidity of these hydrogen atoms may facilitate some non-classical H-bonding and so confer greater stability on **TS1_*cis*_** over **TS1_*trans*_** where the analogous contacts are distinctly longer (O1···H1 2.72 Å and O1···H2 2.65 Å). While not particularly strong individually, these various effects likely combine to stabilise **TS1_*cis*_** over **TS1_*trans*_** and so account for the observed selectivity for the *cis* product.

## Conclusions

In conclusion, an optimised and straightforward three-step synthetic route to a range of pyrrole-derived enone acid starting materials from readily available pyrrole-2-carboxaldehydes is delineated. The catalytic enantioselective synthesis of a range of *cis*-pyrrolizine carboxylate derivatives proceeds with outstanding stereocontrol (14 examples, >95 : 5 dr, >98 : 2 er) with benzotetramisole proving the optimal catalyst for this process. *In situ* ring-opening of the pyrrolizine dihydropyranone products with either MeOH or a range of amines leads to the desired products in excellent yield and enantioselectivity. Computation has been used to probe the factors leading to high stereocontrol in this reaction process, with the formation of the observed *cis*-stereoisomer predicted to be both kinetically and thermodynamically favoured. Further work from this laboratory will utilise this methodology for the synthesis of target molecules and probe the utility of isothioureas and other Lewis bases in enantioselective catalysis.
