# Supplementary material for: Isothiourea-catalysed enantioselective pyrrolizine synthesis: synthetic and computational studies
Source: Org Biomol Chem. 2016 Jul 21;14(38):8957–65. doi: 10.1039/c6ob01557c (PMC5314687; doi:10.1039/c6ob01557c)
Supplement: Supplementary file 2 [file OB-014-C6OB01557C-s002.pdf]

Electronic Supplementary Material (ESI) for Organic & Biomolecular Chemistry.
This journal is © The Royal Society of Chemistry 2016

Catalytic Enantioselective Synthesis of Pyrrolizine Carboxylates using Isothiourea Catalysis: A Synthetic and Computational Study

Daniel G. Stark,[a] Patrick Williamson,[a] Emma R. Gayner,[b] Stefania F. Musolino,[a] Ryan W. F. Kerr,[a] Alexandra M. Z. Slawin,[a] Timothy J. C. O'Riordan,[c] Stuart A. Macgregor*[b] and A. D. Smith*[a]

Table of Contents

1.1 General Information **2**

1.2 General Procedures **3**

1.3 Experimental Information **5**

1.3.1 Synthesis of Pyrrole Aldehydes 5

1.3.2 Synthesis of Pyrrolyl Enones 6

1.3.3 *N*-Alkylation of Pyrrolyl Enones 10

1.3.4 Hydrolysis of Pyrrolyl Enone-Esters 15

1.3.5 Intramolecular Isothiourea-Catalysed Michael Addition-Lactonisation 20

1.3.6 Intramolecular Isothiourea-Catalysed Michael Addition-Lactonisation/Ring Opening . 21

1.3.7 Derivatisations 30

1.4 Computational Details **33**

1.5 Computed Reaction Profiles and Labelling Schemes **34**

1.6 Computed Cartesian Coordinates (Å) and Energies (au) for all stationary points **36**

1.7 References and Notes **53**

1.8 ^{1}H NMR, ^{13}C{^{1}H} NMR and HPLC Data for Novel Compounds **53**

1.1 General Information

Reactions involving moisture sensitive reagents were carried out under a nitrogen atmosphere using standard vacuum line techniques in addition to dry solvents. All glassware used was flame dried and cooled under vacuum. For moisture sensitive reactions, solvents (THF, CH_2Cl_2, toluene, hexane and Et_2O) were obtained anhydrous and purified by an alumina column (Mbraun SPS-800). Petrol is defined as petroleum ether 40-60 °C. All other solvents and commercial reagents were used as supplied without further purification unless stated otherwise.

Room temperature (rt) refers to 20-25 °C. Temperatures of 0 °C and −78 °C were obtained using ice/water and CO_2(s)/acetone baths respectively. Temperatures of 0 °C to -50 °C for overnight reactions were obtained using an immersion cooler (HAAKE EK 90). Reflux conditions were obtained using an oil bath equipped with a contact thermometer. *Under reduced pressure* refers to the use of a Büchi Rotavapor R-2000 rotary evaporator with a Vacubrand CVC_2 vacuum controller or a Heidolph Laborota 4001 rotary evaporator with a vacuum controller.

Analytical thin layer chromatography was performed on pre-coated aluminium plates (Kieselgel 60 F_{254} silica). Plates were visualised under UV light (254 nm) or by staining with either phosphomolybdic acid or $KMnO_4$ followed by heating. Flash column chromatography was performed on Kieselgel 60 silica in the solvent system stated under a positive pressure of compressed air or on a Biotage® IsoleraTM 4, using Biotage® Snap Ultra or Biotage® KP Sil columns under the solvent system stated.

^{1}H, ^{13}C and ^{19}F nuclear magnetic resonance (NMR) spectra were acquired on either a Bruker Avance 300 (300 MHz, ^{1}H, 75 MHz ^{13}C, 282 MHz ^{19}F), Bruker Avance II 400 (400 MHz, ^{1}H, 100 MHz ^{13}C, 376 MHz ^{19}F) or a Bruker Avance II 400 (500 MHz, ^{1}H, 125 MHz ^{13}C, 470 MHz ^{19}F) spectrometer at ambient temperature in the deuterated solvent stated. All chemical shifts are quoted in parts per million (ppm) relative to the residual solvent as the internal standard. All coupling constants, *J*, are quoted in Hz. Multiplicities are indicated by: s (singlet), d (doublet), t (triplet), q (quartet), sept (septet), ABq (AB quartet), sept (septet), oct (octet), m (multiplet), dd (doublet of doublets), ddd (doublet of doublet of doublets, dt (doublet of triplets), dq (doublet of quartets) and td (triplet of doublets). The abbreviation Ar is used to denote aromatic, Ph to denote phenyl, Bn to denote benzyl, py to denote pyridyl and br to denote broad.

Infrared spectra (ν_{max}/cm^{-1}) were recorded on either a Perkin-Elmer Spectrum GX FT-IR spectrometer using a Shimadzu IRAffinity-1 using a Pike attenuated total reflectance (ATR) accessory. Only the characteristic peaks are quoted.

Melting points were recorded on an Electrothermal 9100 melting point apparatus and are uncorrected.

HPLC analyses were obtained on two separate machines; a Gilson HPLC consisting of a Gilson 305 pump, Gilson 306 pump, Gilson 811C dynamic mixer, Gilson 805 manometric module, Gilson 401C dilutor, Gilson 213XL sample injector and sample detection was performed with a Gilson 118 UV/vis detector while the temperature was assumed to be 20 °C; a Shimadzu HPLC consisting of a DGU-20A5 degasser, LC-20AT liquid chromatograph, SIL-20AHT autosampler, CMB-20A communications bus module, SPD-M20A diode array detector and a CTO-20A column oven which allowed the temperature to be set from 25-40 °C. Separation was achieved using DAICEL CHIRALCEL OD-H and OJ-H columns or DAICEL CHIRALPAK AD-H, AS-H, IA, IB, IC and ID columns. All chiral HPLC traces were compared to the authentic racemic spectrum prepared in analogous fashion.

Mass spectrometry (*m/z*) data were acquired by electrospray ionisation (ESI), electron impact (EI), atmospheric solids analysis probe (ASAP) or nanospray ionisation (NSI) either at the University of St Andrews or the EPSRC National Mass Spectrometry Service Centre, Swansea. At the University of St Andrews, low and high resolution ESI MS were carried out on a Micromass LCT spectrometer. At the EPSRC National Mass Spectrometry Service Centre, low resolution NSI MS was carried out on a Micromass Quattro II spectrometer and high resolution NSI MS on a Thermofisher LTQ Orbitrap XL spectrometer.

Optical rotations were measured on a Perkin Elmer Precisely/Model-341 polarimeter operating at the sodium D line with a 100 mm path cell at rt.

1.2 General Procedures

General procedure A: *Preparation of Pyrrole 2-Carboxaldehydes*

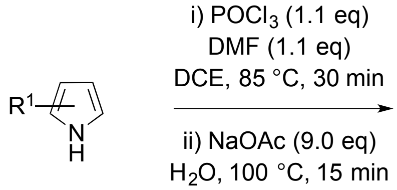

Following literature procedure,[1] $POCl_3$ (1.1 eq) was added dropwise to DMF (1.1 eq) and the reaction was stirred for 15 min at rt. DCE (2.2 M in DMF) was added and the mixture cooled to 0 °C. A solution of requisite pyrrole (1.0 eq) in DCE (2.0 M in pyrrole) was added dropwise and the reaction was heated to 85 °C for 15 min before cooling to rt. A solution of aq. NaOAc (9.0 M, 9.0 eq) was added and the biphasic mixture stirred at 100 °C for 15 min. The reaction was cooled to rt and the phases separated. The aqueous phase was extracted with Et_2O (×3). The combined organics were washed with $NaHCO_3$, dried over $MgSO_4$ and concentrated under reduced pressure to provide the desired pyrrole 2-carboxaldehyde.

General Procedure B: *Preparation of Pyrrolyl Enones*

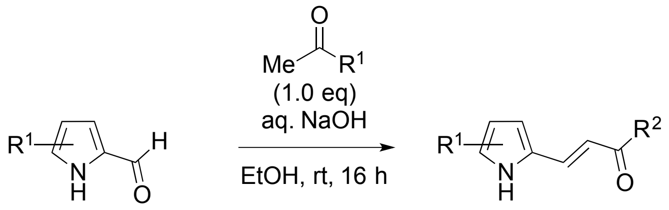

To an aqueous solution of NaOH (10% w/v in H_2O, 2.0 eq) was added dropwise to a solution of pyrrole-2-carboxaldehyde (1.0 eq) and the requisite ketone (1.0 eq) in ethanol (2.5 M in ketone). The reaction was stirred at rt for 16 h then acidified to pH 3 with aqueous HCl (2 M). The resultant precipitate was filtered, washed with cold ethanol to provide the desired enone product. Products were purified as described.

General Procedure C: *N-Alkylation of Pyrrolyl Enones*

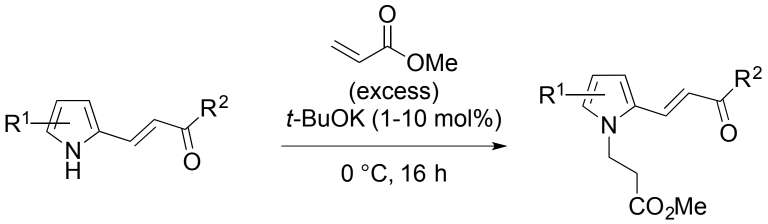

To a solution of pyrrole enone (1.0 eq) in methyl acrylate (0.2 M in pyrrolyl enone) was added potassium *tert*-butoxide (1-10 mol%) and the reaction stirred at 70 °C for 16 h. The reaction was concentrated under reduced pressure, diluted with EtOAc, washed with brine (×3), dried over $MgSO_4$ and concentrated under reduced pressure to provide the crude product. Products were isolated by column chromatography in the solvent system stated.

General Procedure D: *Hydrolysis of Pyrrolyl Enone-Esters*

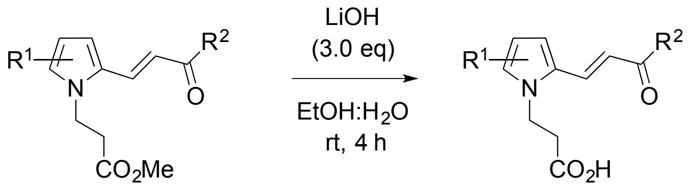

To a solution of pyrrolyl enone ester (1.0 eq) in H_2O/ethanol (1:1) was added LiOH.H_2O (4.0 eq) and reaction stirred at rt for 4 h. The reaction was then basified to pH 8 with aq. NaOH (2 M) and washed with Et_2O (×3). The aqueous layer was carefully acidified to pH 3 (caution: acidifying beyond pH 3 can lead to decomposition of product) with aq. HCl (2 M) and extracted with EtOAc (×3). Combined organic layers were washed with brine, dried over $MgSO_4$ and concentrated under reduced pressure to provide enone-acid products.

General Procedure E: *Michael Addition-Lactonisation in situ Ring Opening*

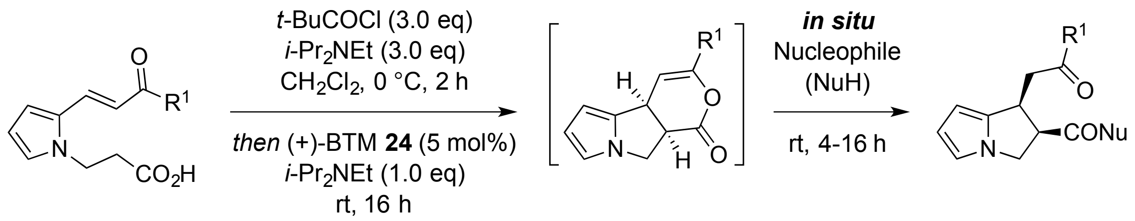

To a solution of pyrrolyl enone-acid (1.0 eq) in CH_2Cl_2 (0.1 M) was added pivaloyl chloride (3.0 eq) and *i*-Pr_2NEt (3.0 eq) at 0 °C and the reaction was allowed to warm to rt over 2 h. (+)-BTM **24** (5 mol%) and *i*-Pr_2NEt (1.5 eq) was added and reaction stirred at rt overnight. The requisite nucleophile was then added and the reaction stirred until complete by TLC analysis. Reaction was quenched with aq. HCl (1 M) and extracted with CH_2Cl_2 (×3). Combined organic layers were dried over $MgSO_4$ and concentrated in vacuo to provide crude products. Products were isolated by column chromatography in the solvent system stated.

1.3 Experimental Information

1.3.1 Synthesis of Pyrrole Aldehydes

1*H*-Pyrrole 2-carboxaldehyde

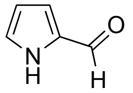

48

Following general procedure A, $POCl_3$ (2.0 mL, 21.5 mmol), DMF (1.7 mL, 21.5 mmol) and DCE (10 mL), pyrrole (1.4 mL, 20.2 mmol) in DCE (10 mL) and NaOAc (15 g, 182.9 mmol) in H_2O (20 mL) gave the title compound as a red oil (1.83 g, 96%); ^{1}H NMR (500 MHz, d_6-DMSO) 6.33 (1H, m, pyrrolyl(4)*H*), 7.01 (1H, m, pyrrolyl(3)*H*), 7.18 (1H, m, pyrrolyl(5)*H*), 9.49 (1H, s, C*H*O), 11.13 (1H, br. s, NH). All data in accordance with literature.[1]

4,5,6,7-Tetrahydro-1*H*-indole

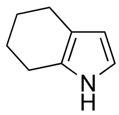

49

Following the literature procedure,[2] $NH_2OH.HCl$ (3.54 g, 50.9 mmol), cyclohexanone (5.28 mL, 50.9 mmol) and KOH (2.86 g, 50.9 mmol) were stirred in DMSO (42 mL) at 70 °C for 30 min. The temperature was increased to 120 °C and further KOH (7.15 g, 127.4 mmol) was added before the slow addition of a solution of DCE (20.5 mL, 259.5 mmol) in DMSO (65 mL) *via* syringe pump over 4 h. The reaction was cooled and added to aq. NH_4Cl (100 mL, 10% in H_2O) and extracted with Et_2O

(3×30 mL). The combined organics were washed with aq. KOH (1 M) then dried over $MgSO_4$ and concentrated under reduced pressure to give the crude product. Purification by column chromatography provided the title compound as pink solid (2.19 g, 35%); mp 50-51 °C {Lit.[142] 51-52 °C}; ^{1}H NMR (500 MHz, $CDCl_3$) 1.74-1.87 (4H, m, C(5)*H₂* and C(6)*H₂*), 2.57 (4H, dt, *J* 5.8, 20.2, C(4)*H₂* and C(7)*H₂*), 6.00 (1H, br. s, C(3)*H*), 6.64 (1H, t, *J* 2.6, C(2)*H*), 7.70 (1H, N*H*). All data in accordance with literature.[2]

4,5,6,7-tetrahydro-1*H*-indole-2-carbaldehyde

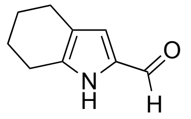

50

Following general procedure A, $POCl_3$ (1.85 mL, 19.9 mmol), DMF (1.54 mL, 19.9 mmol) and DCE (9 mL), 4,5,6,7-tetrahydro-1*H*-indole **49** (1.4 mL, 20.2 mmol) in DCE (9 mL) and NaOAc (13.3 g, 162.3 mmol) in H_2O (18 mL) gave the title compound as a brown oil (2.21 g, 82%); ^{1}H NMR (500 MHz, $CDCl_3$) 1.71-1.83 (4H, m, C(5)H_2 and C(6)H_2), 2.51 (2H, t, *J* 6.1, (C(4)H_2), 2.67 (2H, t, *J* 6.2, C(7)H_2), 6.73 (1H, s, C(3)*H*), 9.17 (1H, s, C*H*O), 10.5 (1H, br. s, N*H*). All data in accordance with literature.[3]

1.3.2 Synthesis of Pyrrolyl Enones

(*E*)-1-phenyl-3-(1*H*-pyrrol-2-yl)prop-2-en-1-one

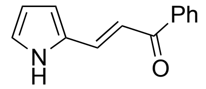

51

Following general procedure B, pyrrole carboxaldehyde (5.00 g, 52.6 mmol), aq. NaOH (5 mL, 10% w/v in H_2O) and acetophenone (6.14 mL, 52.6 mmol) in EtOH (21 mL) gave the title compound as a yellow solid (7.05 g, 68%); mp 135-137 °C; {lit.[4] 136-137 °C}; ^{1}H NMR (500 MHz, $CDCl_3$) 6.34 (1H, s, pyrrolyl(4)*H*), 6.72 (1H, s, pyrrolyl(3)*H*), 7.00 (1H, s, pyrrolyl(5)*H*), 7.20 (1H, d, *J* 15.5, C(2)*H*), 7.47 (2H, m, C(1)Ar(3,5)*H*), 7.33 (1H, m, C(1)Ar(4)*H*), 7.78 (1H, d, *J* 15.5, C(3)*H*), 7.98 (2H, d, *J* 7.3, C(1)Ar(2,6)*H*). All data in accordance with literature.[4]

(*E*)-1-(4-bromophenyl)-3-(1*H*-pyrrol-2-yl)prop-2-en-1-one

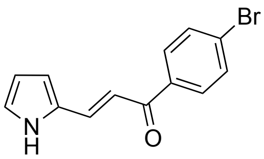

52

Following general procedure B, pyrrole carboxaldehyde (3.00 g, 31.5 mmol), aq. NaOH (3 mL, 10% w/v in H_2O) and 4'-bromoacetophenone (6.27 g, 31.5 mmol) in EtOH (13 mL) gave the title compound as a yellow solid (5.48 g, 63%); mp 182-184 °C; ν_{max} (ATR)/cm^{-1} 975, 1330, 1539 (C-O), 3307 (C-H); ^{1}H NMR (500 MHz, d_6-DMSO) 6.35 (1H, dt, *J* 2.3, 3.6, pyrrolyl(4)*H*), 6.75 (1H, br. s, pyrrolyl(3)*H*), 7.17 (1H, br. s, pyrrolyl(5)*H*), 7.53 (1H, d, *J* 15.4, C(2)*H*), 7.61 (1H, d, *J* 15.5, C(3)*H*), 7.79 (2H, d, *J* 8.7, C(1)Ar(3,5)*H*), 7.96 (2H, d, *J* 8.6, C(1)Ar(2,6)*H*), 11.74 (1H, s, N*H*); ^{13}C NMR (125 MHz d_6-DMSO) 110.8 (pyrrolyl*C*(4)H), 114.1 (*C*(2)H), 116.8 (pyrrolyl*C*(3)H), 124.6 (pyrrolyl*C*(5)H), 126.6 (pyrrolyl*C*(2)), 129.1 (C(1)Ar*C*(4)), 129.9 (C(1)Ar*C*(3,5)H), 131.8 (C(1)Ar*C*(2,6)H), 134.7 (C(1)Ar*C*(1)), 137.3 (*C*(3)H), 187.3 (*C*(1)); HRMS (APCI$^+$), $C_{13}H_{10}BrNO$ $[M+H]^+$, requires 277.9998, found 277.9999 (+0.2 ppm).

(*E*)-1-(3-bromophenyl)-3-(1*H*-pyrrol-2-yl)prop-2-en-1-one

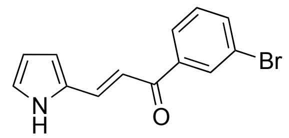

53

Following general procedure B, pyrrole carboxaldehyde (3.00 g, 31.5 mmol), aq. NaOH (3 mL, 10% w/v in H_2O) and 3'-bromoacetophenone (4.16 mL, 31.5 mmol) in EtOH (13 mL) gave the title compound as a crude mixture of E/Z isomers (85:15) (5.48 g, 63%). Data for (*E*)-isomer; ^{1}H NMR (500 MHz, d_6-DMSO) 6.21 (1H, m, pyrrolyl(4)*H*), 6.75 (1H, br. s, pyrrolyl(3)*H*), 7.16 (1H, br. s, pyrrolyl(5)*H*), 7.49-7.54 (2H, m, Ar(5)*H* and C(2)*H*), 7.61 (1H, d, *J* 15.3, C(3)*H*), 7.81-7.82 (1H, m, Ar(6)*H*), 7.99-8.00 (1H, m, Ar(4)*H*), 8.14-8.15 (1H, m, Ar(2)*H*), 11.7 (1H, s, N*H*). Due to instability this compound was used immediately without further characterisation.

(*E*)-3-(1*H*-pyrrol-2-yl)-1-(thiophen-2-yl)prop-2-en-1-one

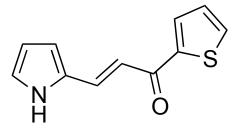

54

Following general procedure B, pyrrole carboxaldehyde (3.00 g, 31.5 mmol), aq. NaOH (3 mL, 10% w/v in H_2O) and 2-acetylthiophene (3.40 mL, 31.5 mmol) in EtOH (13 mL) gave the title compound as a yellow solid (4.55 g, 71%); mp 131-132 °C {Lit.[5] 130 °C}; ^{1}H NMR (500 MHz, $CDCl_3$) 6.34-6.35 (1H, m, pyrrolyl(4)*H*), 6.73 (1H, s, pyrrolyl(3)*H*), 7.00-7.03 (2H, m, C(2)*H* and pyrrolyl(5)*H*), 7.16 (1H, dd, *J* 3.8, 4.9, C(1)Ar(3)*H*), 7.64 (1H, dd, *J* 1.0, 4.9, C(1)Ar(3)*H*), 7.76 (1H, d, *J* 15.4, C(3)*H*), 7.81 (1H, dd, *J* 1.1, 3.8, C(1)Ar(5)*H*). All data in accordance with literature.[5]

(*E*)-1-(4-methoxyphenyl)-3-(1*H*-pyrrol-2-yl)prop-2-en-1-one

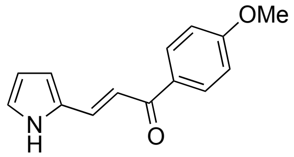

55

Following general procedure B, pyrrole carboxaldehyde (1.50 g, 15.8 mmol), aq. NaOH (1.5 mL, 10% w/v in H_2O) and 4'-methoxyacetophenone (2.37 g, 15.8 mmol) in EtOH (6.3 mL) gave the title compound as a yellow solid (2.30 g, 64%); mp 164-166 °C; ν_{max} (ATR)/cm^{-1} 1134, 1539 (C-O), 3224 (C-H); ^{1}H NMR (500 MHz, $CDCl_3$) 3.88 (3H, s, C(1)ArOC*H*$_3$), 6.33 (1H, br. s, pyrrolyl(4)*H*), 6.71 (1H, s, pyrrolyl(3)*H*), 6.95-6.98 (3H, m, C(1)Ar(3,5)*H* and pyrrolyl(5)*H*), 7.16 (1H, d, *J* 15.5, C(2)*H*), 7.74 (1H, d, *J* 15.5, C(3)*H*), 8.00 (2H, d, *J* 8.8, C(1)Ar(2,6)*H*), 8.85 (1H, s, N*H*); ^{13}C NMR (125 MHz) 55.6 (C(1)ArO*C*H$_3$), 111.6 (pyrrolyl*C*(4)H), 113.9 (C(1)Ar*C*(3,5)H), 114.9 (pyrrolyl*C*(3)H), 115.7 (*C*(2)H), 122.9 (pyrrolyl*C*(5)H), 129.5 (pyrrolyl*C*(2)), 130.7 (C(1)Ar*C*(2,6)H), 131.6 (C(1)Ar*C*(1)), 133.8 (*C*(3)H), 163.3 (C(1)Ar*C*(4)), 188.7 (*C*(1)); HRMS (APCI$^+$), $C_{14}H_{13}NO_2$ $[M+H]^+$, requires 226.0863, found 226.0862 (−0.2 ppm).

(*E*)-1-(Naphthalen-2-yl)-3-(1*H*-pyrrol-2-yl)prop-2-en-1-one

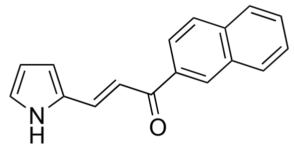

56

Following general procedure B, pyrrole carboxaldehyde (3.00 g, 31.5 mmol), aq. NaOH (3 mL, 10% w/v in H_2O) and 2-acetylnaphthalene (5.36 g, 31.5 mmol) in EtOH (20 mL) gave the title compound as a yellow solid (5.92 g, 76%); mp 158-160 °C; ν_{max} (ATR)/cm^{-1} 820, 974, 1541 (C-O), 3284 (C-H); ^{1}H NMR (400 MHz, $CDCl_3$) 6.36 (1H, dd, *J* 1.1, 2.5, pyrrolyl(4)*H*), 6.75-6.76 (1H, m, pyrrolyl(3)*H*), 7.02 (1H, td, 1.4, 2.7, pyrrolyl(5)*H*), 7.30 (1H, d, *J* 15.5, C(2)*H*), 7.57 (2H, dddd, *J* 1.4, 6.9, 8.1, 19.5, Ar*H*), 7.80 (1H, d, *J* 15.5, C(3)*H*), 7.88-7.97 (3H, m, Ar*H*), 8.07 (1H, dd, *J* 1.8, 8.6, Ar*H*), 8.49 (1H, s, Ar*H*), 8.91 (1H, br. s, N*H*); ^{13}C NMR (100 MHz) 111.7 (pyrrolyl*C*(4)H), 115.6 (pyrrolyl*C*(3)H), 116.0 (*C*(2)H), 123.2 (pyrrolyl*C*(5)H), 124.7 (Ar*C*H), 126.9 (Ar*C*H), 128.0 (Ar*C*H), 128.3 (Ar*C*H), 128.6 (Ar*C*H), 129.5 (pyrrolyl*C*(2)), 129.6 (Ar*C*H), 129.6 (Ar*C*H), 132.7 (Ar*C*), 134.5 (Ar*C*), 135.5 (Ar*C*), 136.1 (*C*(3)H), 190.2 (*C*(1)); HRMS (NSI$^+$), $C_{17}H_{14}NO_2$ $[M+H]^+$, requires 248.1068, found 248.1070 (−0.8 ppm).

(*E*)-1-(Naphthalen-1-yl)-3-(1*H*-pyrrol-2-yl)prop-2-en-1-one

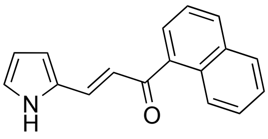

57

Following general procedure B, pyrrole carboxaldehyde (2.00 g, 21.0 mmol), aq. NaOH (2 mL, 10% w/v in H_2O) and 1-acetylnaphthalene (6.14 mL, 52.6 mmol) in EtOH (8.4 mL) gave the title compound as a yellow oil (1.06 g, 20%); ^{1}H NMR (300 MHz, $CDCl_3$) 6.30-6.33 (1H, m, pyrrolyl(4)*H*), 6.62 (1H, br. s, pyrrolyl(3)*H*), 6.89 (1H, d, *J* 15.9, C(2)*H*), 6.98 (1H, br. s, pyrrolyl(5)*H*), 7.45 (1H, d, *J* 16.0, C(3)*H*), 7.50-7.56 (3H, m, Ar*H*), 7.69 (1H, dd, *J* 1.2, 7.1, Ar*H*), 7.88-7.92 (1H, m, Ar*H*), 7.96-7.99 (1H, m, Ar*H*), 8.23-8.26 (1H, m, Ar*H*), 8.84 (1H, br. s, N*H*); ^{13}C NMR (100 MHz) 111.7 (pyrrolyl*C*(4)H), 116.4 (pyrrolyl*C*(3)H), 121.0 (*C*(2)H), 124.2 (pyrrolyl*C*(5)H), 124.8 (Ar*C*H), 125.8 (Ar*C*H), 126.5 (Ar*C*H), 126.6 (Ar*C*H), 127.3 (Ar*C*H), 128.5 (Ar*C*H), 129.0 (pyrrolyl*C*(2)), 130.6 (Ar*C*), 131.1 (Ar*C*H), 133.9 (Ar*C*), 136.9 (*C*(3)H), 137.7 (Ar*C*), 196.7 (*C*(1)); HRMS (NSI$^+$), $C_{17}H_{14}NO$ $[M+H]^+$, requires 248.1070, found 248.1071 (+0.4 ppm).

(*E*)-4-(1*H*-Pyrrol-2-yl)but-3-en-2-one

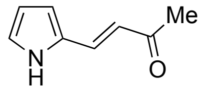

58

Following the procedure from Chimni and Mahajan,[5] to a solution of pyrrole aldehyde (3.00 g, 31.5 mmol) and acetone (23.2 mL, 315.0 mmol) in H_2O (157.5 mL) was added pyrrolidine (0.77 mL, 9.45 mmol) and the reaction stirred at rt for 16 h. The reaction was quenched with HCl (1M in H_2O), extracted with CH_2Cl_2 (×3), dried over $MgSO_4$ and concentrated under reduced pressure to the title compound as a yellow solid (2.97 g, 70%); mp 115-117 °C {Lit.[6] 117-120 °C}; ^{1}H NMR (300 MHz, $CDCl_3$) 2.33 (3H, s, C(1)H_3), 6.30-6.36 (2H, m, C(3)*H* and pyrrolyl(4)*H*), 6.60-6.62 (1H, m, pyrrolyl(3)*H*), 6.98-6.99 (1H, m, pyrrolyl(5)*H*), 7.41 (1H, d, *J* 16.2, C(4)*H*), 8.94 (1H, br. s, N*H*). All data in accordance with literature.[6]

(*E*)-1-Phenyl-3-(4,5,6,7-tetrahydro-1*H*-indol-2-yl)prop-2-en-1-one

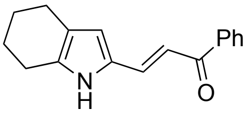

59

Following general procedure B, 4,5,6,7-tetrahydro-1*H*-indole-2-carbaldehyde **50** (2.21 g, 14.6 mmol), aq. NaOH (3 mL, 10% w/v in H_2O) and acetophenone (1.70 mL, 14.6 mmol) in EtOH (10 mL) gave crude product. The reaction mixture was neutralised to pH 7 with aq. HCl (1 M) and extracted with EtOAc (×3), dried over $MgSO_4$ and concentrated under reduced pressure. Purification by column

chromatography (EtOAc:hexane 7.5:92.5) gave the title compound as a brown oil (1.26 g, 34%); ^{1}H NMR (500 MHz, $CDCl_3$) 1.69-1.87 (4H, m, C(3)C(5)H_2 and C(3)C(6)H_2), 2.52 (2H, t, *J* 6.0, (C(3)C(4)H_2), 2.64 (2H, t, *J* 6.1, C(3)C(7)H_2), 6.47 (1H, br. s, C(3)C(3)*H*), 7.01 (1H, d, *J* 15.4, C(3)*H*), 7.43-7.48 (2H, m, C(1)Ar(3,5)*H*), 7.50-7.58 (1H, m, C(1)Ar(4)*H*), 7.68 (1H, d, *J* 15.4, C(2)*H*), 7.95-7.97 (2H, m, C(1)Ar(2,6)*H*), 8.48 (1H, br. s, N*H*). All data in accordance with literature.[7]

1.3.3 *N*-Alkylation of Pyrrolyl Enones

Methyl (*E*)-3-(2-(3-oxo-3-phenylprop-1-en-1-yl)-1*H*-pyrrol-1-yl)propanoate

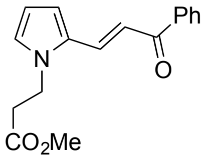

60

Following general procedure C, pyrrolyl enone **51** (5.00 g, 25.6 mmol), potassium *tert*-butoxide (29 mg, 0.26 mmol) in methyl acrylate (135 mL) gave the title compound as a yellow oil (6.74 g, 93%); ν_{max} (ATR)/cm^{-1} 1213, 1566, 1583, 1732 (ester C=O), 2951 (C-H); ^{1}H NMR (500 MHz, $CDCl_3$) 2.78 (2H, t, *J* 6.9, C(2)H_2), 3.69 (3H, s, CO_2CH_3), 4.39 (2H, t, *J* 6.9, C(3)H_2), 6.23-6.24 (1H, m, pyrrolyl(4)*H*), 6.85-6.86 (1H, m, pyrrolyl(3)*H*), 6.91-6.92 (1H, m, pyrrolyl(5)*H*), 7.33 (1H, d, *J* 15.1, pyrrolyl(2)C(2)*H*), 7.48-7.51 (2H, m, C(O)Ar(3,5)*H*), 7.55-7.58 (1H, C(O)Ar(4)*H*), 7.79 (1H, d, *J* 15.1, pyrrolyl(2)C(1)*H*), 8.01-8.02 (2H, C(O)Ar(2,6)*H*); ^{13}C NMR (75 MHz, $CDCl_3$) 36.3 (*C*(2)H_2), 42.7 (*C*(3)H_2), 52.2 (CO_2CH_3), 110.4 (pyrrolyl*C*(4)H), 112.8 (pyrrolyl*C*(3)H), 117.2 (pyrrolyl(2)*C*(2)H), 127.2 (pyrrolyl*C*(5)H), 128.4 (C(O)Ar*C*(2,6)H), 128.7 (C(O)Ar*C*(3,5)H), 129.4 (pyrrolyl*C*(2)), 131.7 (pyrrolyl(2)*C*(1)H), 132.6 (C(O)Ar*C*(4)H), 138.8 (C(O)Ar*C*(1)), 171.2 (*C*O_2Me), 189.8 (pyrrolyl(2)*C*(3)); HRMS (NSI$^+$), $C_{17}H_{17}NO_3Na$ [M+Na]$^+$, requires 306.1101, found 306.1094 (−2.2 ppm).

Methyl (*E*)-3-(2-(3-(4-bromophenyl)-3-oxoprop-1-en-1-yl)-1*H*-pyrrol-1-yl)propanoate

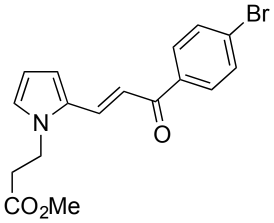

61

Following general procedure C, pyrrolyl enone **52** (3.00 g, 10.9 mmol), potassium *tert*-butoxide (57 mg, 0.26 mmol) in methyl acrylate (57 mL) gave the title compound as a yellow oil (3.51 g, 89%); ν_{max} (ATR)/cm^{-1} 1170, 1678, 1732 (ester C=O), 2951 (C-H); ^{1}H NMR (400 MHz, $CDCl_3$) 2.77 (2H, t, *J* 6.9, C(2)H_2), 3.68 (3H, s, CO_2CH_3), 4.38 (2H, t, *J* 6.9, C(3)H_2), 6.23 (1H, m, pyrrolyl(4)*H*), 6.86 (1H, dd, *J* 1.1, 4.0, pyrrolyl(3)*H*), 6.92 (1H, dd, *J* 1.6, 2.5, pyrrolyl(5)*H*), 7.26 (1H, d, *J* 15.1, C

pyrrolyl(2)C(2)*H*), 7.62 (2H, d, *J* 8.7 C(O)Ar(3,5)*H*), 7.79 (1H, d, *J* 15.1, pyrrolyl(2)C(1)*H*), 7.88 (2H, d, *J* 8.7 C(O)Ar(2,6)*H*); ^{13}C NMR (100 MHz, $CDCl_3$) 36.3 (*C*(2)H_2), 42.6 (CO_2CH_3), 52.2 (*C*(3)H_2), 110.5 (pyrrolyl*C*(4)H), 113.2 (pyrrolyl*C*(3)H), 116.4 (pyrrolyl(2)*C*(2)H), 127.5 (pyrrolyl*C*(5)H), 127.5 (C(O)Ar*C*(4)), 129.3 (pyrrolyl*C*(2)), 129.9 (C(O)Ar*C*(2,6)H), 131.9 (C(O)Ar*C*(3,5)H), 132.1 (pyrrolyl(2)*C*(1)H), 137.5 (C(O)Ar*C*(1)), 171.1 (*C*O_2Me), 188.5 (pyrrolyl(2)*C*(3)); HRMS (NSI$^+$), $C_{17}H_{16}Br^{79}NO_3Na$ [M+Na]$^+$, requires 384.0206, found 384.0207 (+0.3 ppm).

Methyl (*E*)-3-(2-(3-(3-bromophenyl)-3-oxoprop-1-en-1-yl)-1*H*-pyrrol-1-yl)propanoate

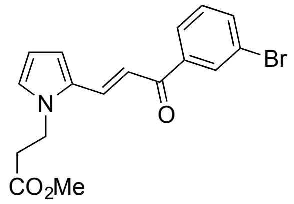

62

Following general procedure C, pyrrolyl enone **53** (3.00 g, 10.9 mmol), potassium *tert*-butoxide (122 mg, 1.09 mmol) in methyl acrylate (57 mL) gave the title compound as a yellow oil (1.62 g, 41%); ν_{max} (ATR)/cm^{-1} 1560, 1578, 1734 (ester C=O), 2953 (C-H); ^{1}H NMR (500 MHz, $CDCl_3$) 2.78 (2H, t, *J* 6.9, C(2)*H₂*), 3.69 (3H, s, CO_2CH_3), 4.40 (2H, t, *J* 6.9, C(3)H_2), 6.24-6.25 (1H, m, pyrrolyl(4)*H*), 6.89 (1H, dd, *J* 1.3, 3.9, pyrrolyl(3)*H*), 6.93-6.94 (1H, m, pyrrolyl(5)*H*), 7.24 (1H, d, *J* 15.1, pyrrolyl(2)C(2)*H*), 7.37 (1H, t, *J* 7.9, C(O)Ar(5)*H*), 7.68 (1H, ddd, *J* 1.0, 1.9, 7.9, C(O)Ar(6)*H*), 7.79 (1H, d, *J* 15.1, pyrrolyl(2)C(1)*H*), 7.93 (1H, dt, *J* 1.2, 7.7, C(O)Ar(4)*H*), 8.13 (1H, m, C(O)Ar(2)*H*); ^{13}C NMR (125 MHz, $CDCl_3$) 36.3 (*C*(2)H_2), 42.6 (CO_2CH_3), 52.2 (*C*(3)H_2), 110.6 (pyrrolyl*C*(4)H), 113.4 (pyrrolyl*C*(3)H), 116.3 (pyrrolyl(2)*C*(2)H), 123.0 (C(O)Ar*C*(3)), 126.8 (C(O)Ar*C*(4)H), 127.7 (pyrrolyl*C*(5)H), 129.2 (C(O)Ar*C*(2)), 130.3 (C(O)Ar*C*(5)H), 131.4 (C(O)Ar*C*(2)H), 132.4 (pyrrolyl(2)*C*(1)H), 135.4 (C(O)Ar*C*(6)H), 140.6 (C(O)Ar*C*(1)), 171.1 (*C*O_2Me), 188.3 (pyrrolyl(2)*C*(3)); HRMS (NSI$^+$), $C_{17}H_{16}Br^{79}NO_3Na$ [M+Na]$^+$, requires 384.0206, found 384.0201 (−1.2 ppm).

Methyl (*E*)-3-(2-(3-oxo-3-(thiophen-2-yl)prop-1-en-1-yl)-1*H*-pyrrol-1-yl)propanoate

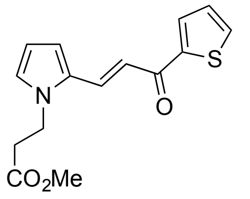

63

Following general procedure C, pyrrolyl enone **54** (3.00 g, 14.8 mmol), potassium *tert*-butoxide (166 mg, 1.48 mmol) in methyl acrylate (78 mL) gave the title compound as a yellow oil (1.28 g, 30%); ν_{max} (ATR)/cm^{-1} 1410, 1570, 1635, 1734 (ester C=O), 2951, 3103 (C-H); ^{1}H NMR (300 MHz, $CDCl_3$)

2.77 (2H, t, *J* 6.9, C(2)*H₂*), 3.69 (3H, s, CO_2CH_3), 4.39 (2H, t, *J* 6.9, C(3)*H₂*), 6.22-6.24 (1H, m, pyrrolyl(4)*H*), 6.85 (1H, dd, *J* 1.1, 4.0, pyrrolyl(3)*H*), 6.91 (1H, dd, *J* 1.6, 2.5, pyrrolyl(5)*H*), 7.15-7.21 (2H, m, C(O)thienyl(4)*H* and pyrrolyl(2)C(2)*H*), 7.64 (1H, dd, *J* 1.1, 5.0, C(O)thienyl(3)*H*), 7.77 (1H, d, *J* 15.0, pyrrolyl(2)C(1)*H*), 7.82 (1H, dd, *J* 1.1, 3.8, C(O)thienyl(5)*H*); ^{13}C NMR (75 MHz, $CDCl_3$) 36.3 (*C*(2)H_2), 42.7 (CO_2CH_3), 52.2 (*C*(3)H_2), 110.4 (pyrrolyl*C*(4)H), 112.9 (pyrrolyl*C*(3)H), 117.0 (pyrrolyl(2)*C*(2)H), 127.3 (pyrrolyl*C*(5)H), 128.3 (C(O)thienyl*C*(5)H), 129.2 (pyrrolyl*C*(2)), 131.0 (pyrrolyl(2)*C*(1)H), 131.2 (C(O)thienyl*C*(4)H), 133.3 (C(O)thienyl*C*(3)H), 146.2 (C(O)thienyl*C*(2)), 171.2 (*C*O_2Me), 181.8 (pyrrolyl(2)*C*(3)); HRMS (NSI$^+$), $C_{15}H_{15}NO_3SNa$ $[M+Na]^+$, requires 312.0665, found 312.0665 (0.0 ppm).

Methyl (*E*)-3-(2-(3-(4-methoxyphenyl)-3-oxoprop-1-en-1-yl)-1*H*-pyrrol-1-yl)propanoate

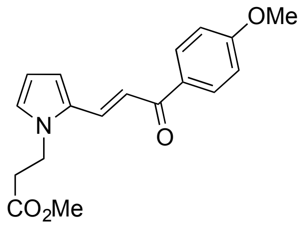

64

Following general procedure C, pyrrolyl enone **55** (2.00 g, 8.80 mmol), potassium *tert*-butoxide (10 mg, 0.088 mmol) in methyl acrylate (46 mL) gave the title compound as a yellow oil (2.56 g, 97%); ν_{max} (ATR)/cm^{-1} 1168, 1581, 1589, 1732 (ester C=O), 2951 (C-H); ^{1}H NMR (300 MHz, $CDCl_3$) 2.78 (2H, t, *J* 6.9, C(2)*H₂*), 3.68 (3H, s, CO_2CH_3), 3.88 (3H, s, ArOC*H₃*), 4.39 (2H, t, *J* 6.9, C(3)*H₂*), 6.21 (1H, m, pyrrolyl(4)*H*), 6.83 (1H, dd, *J* 1.3, 3.9, pyrrolyl(3)*H*), 6.90 (1H, dd, *J* 1.6, 2.5, pyrrolyl(5)*H*), 6.97 (1H, d, *J* 8.9, C(O)Ar(3,5)*H*), 7.34 (1H, d, *J* 15.1, pyrrolyl(2)C(2)*H*), 7.76 (1H, d, *J* 15.1, pyrrolyl(2)C(1)*H*), 8.03 (2H, d, *J* 8.9, C(O)Ar(2,6)*H*); ^{13}C NMR (75 MHz) 36.3 (*C*(2)H_2), 42.6 (*C*(3)H_2), 52.1 (CO_2CH_3), 55.6 (ArO*C*H_3), 110.2 (pyrrolyl*C*(4)H), 112.4 (pyrrolyl*C*(3)H), 113.9 (C(O)Ar*C*(3,5)H), 117.2 (pyrrolyl(2)*C*(2)H), 126.9 (pyrrolyl*C*(5)H), 129.5 (pyrrolyl*C*(2)), 130.6 (C(O)Ar*C*(2,6)H), 131.0 (pyrrolyl(2)*C*(1)H), 131.6 (C(O)Ar*C*(1)), 163.3 (C(O)Ar*C*(4)), 171.2 (*C*O_2Me), 188.1 (pyrrolyl(2)*C*(3)); HRMS (NSI$^+$), $C_{18}H_{20}NO_4$ $[M+H]^+$, requires 313.1387, found 313.1379 (−2.6 ppm).

Methyl (*E*)-3-(2-(3-(naphthalen-2-yl)-3-oxoprop-1-en-1-yl)-1*H*-pyrrol-1-yl)propanoate

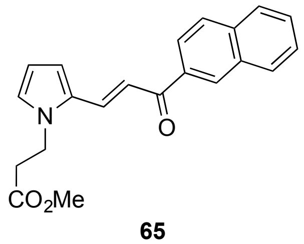

65

Following general procedure C, pyrrolyl enone **56** (3.00 g, 12.1 mmol), potassium *tert*-butoxide (27 mg, 0.24 mmol) in methyl acrylate (64 mL) gave the title compound as a yellow oil (2.53 g, 63%); ν_{max} (ATR)/cm^{-1} 1170, 1570, 1730 (ester C=O), 2951 (C-H); ^{1}H NMR (300 MHz, $CDCl_3$) 2.80 (2H, t, *J* 6.9, C(2)H_2), 3.69 (3H, s, CO_2CH_3), 4.42 (2H, t, *J* 6.9, C(3)H_2), 6.25-6.27 (1H, m, pyrrolyl(4)*H*), 6.92-6.94 (2H, m, pyrrolyl(3)*H* and pyrrolyl(5)*H*), 7.49 (1H, d, *J* 15.1, pyrrolyl(2)C(2)*H*), 7.52-7.63 (2H, m, 2×C(O)Ar*H*), 7.82-8.02 (4H, m, pyrrolyl(2)C(1)*H* and 3×C(O)Ar*H*), 8.11 (1H, dd, *J* 1.7, 8.6, C(O)Ar*H*), 8.53 (1H, br. s, C(O)Ar*H*); ^{13}C NMR (75 MHz, $CDCl_3$) 36.3 (*C*(2)H_2), 42.7 (*C*(3)H_2), 52.2 (CO_2CH_3), 110.4 (pyrrolyl*C*(4)H), 112.9 (pyrrolyl*C*(3)H), 117.3 (pyrrolyl(2)*C*(2)H), 124.6 (C(O)Ar*C*H), 126.8 (C(O)Ar*C*H), 127.2 (C(O)Ar*C*H), 127.9 (C(O)Ar*C*H), 128.3 (C(O)Ar*C*H), 128.6 (C(O)Ar*C*H), 129.5 (pyrrolyl*C*(2)), 129.6 (C(O)Ar*C*H), 129.6 (C(O)Ar*C*H), 131.7 (pyrrolyl(2)*C*(1)H), 132.8 (C(O)Ar*C*), 135.5 (C(O)Ar*C*), 136.1 (C(O)Ar*C*(1)), 171.2 (*C*O_2Me), 189.6 (pyrrolyl(2)*C*(3)); HRMS (NSI^{+}), $C_{21}H_{19}NO_3Na$ [M+Na]$^{+}$, requires 356.1257, found 356.1255 (−0.6 ppm).

Methyl (*E*)-3-(2-(3-(naphthalen-1-yl)-3-oxoprop-1-en-1-yl)-1*H*-pyrrol-1-yl)propanoate

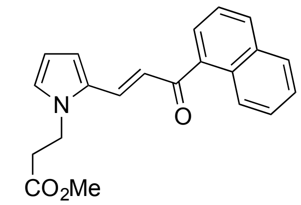

66

Following general procedure C pyrrolyl enone **57** (1.06 g, 4.47 mmol), potassium *tert*-butoxide (5 mg, 0.043 mmol) in methyl acrylate (22.5 mL) gave the title compound as a yellow oil (922 mg, 62%); ν_{max} (ATR)/cm^{-1} 1280, 1570, 1732 (ester C=O), 2961 (C-H); ^{1}H NMR (500 MHz, $CDCl_3$) 2.70 (2H, t, *J* 6.8, C(2)H_2), 3.65 (3H, s, CO_2CH_3), 4.28 (2H, t, *J* 6.8, C(3)H_2), 6.21-6.22 (1H, m, pyrrolyl(4)*H*), 6.80 (1H, dd, *J* 1.2, 3.9, pyrrolyl(3)*H*), 6.90 (1H, br. s, pyrrolyl(5)*H*), 7.08 (1H, d, *J* 15.4, pyrrolyl(2)C(2)*H*), 7.50-7.57 (3H, m, Ar*H*), 7.63 (1H, d, *J* 15.4, 7.76-7.77 (1H, m, Ar*H*), 7.89 (1H, d, *J* 8.3, Ar*H*), 7.96 (1H, d, *J* 8.2, Ar*H*), 8.37 (1H, d, *J* 8.2, Ar*H*); ^{13}C NMR (125 MHz) 36.0 (*C*(2)H_2), 42.5 (*C*(3)H_2), 52.0 (CO_2CH_3), 110.4 (pyrrolyl*C*(4)H), 113.3 (pyrrolyl*C*(3)H), 122.0 (pyrrolyl(2)*C*(2)H), 124.6 (C(O)Ar*C*H), 125.8 (C(O)Ar*C*H), 126.4 (C(O)Ar*C*H), 126.8 (C(O)Ar*C*H), 127.2 (C(O)Ar*C*H), 127.5 (C(O)Ar*C*H), 128.4 (C(O)Ar*C*H), 128.8 (pyrrolyl*C*(2)), 130.5 (C(O)Ar*C*), 131.3 (C(O)Ar*C*H), 132.4 (pyrrolyl(2)*C*(1)H), 133.8 (C(O)Ar*C*), 137.9 (C(O)Ar*C*(1)), 170.9 (*C*O_2Me), 194.8 (pyrrolyl(2)*C*(3)); HRMS (NSI^{+}), $C_{21}H_{20}NO_3$ [M+H]$^{+}$, requires 334.1438, found 334.1439 (+0.3 ppm).

Methyl (*E*)-3-(2-(3-oxo-3-phenylprop-1-en-1-yl)-4,5,6,7-tetrahydro-1*H*-indol-1-yl)propanoate

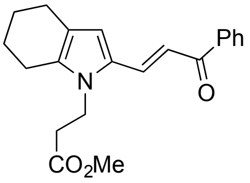

67

Following general procedure C, pyrrolyl enone **58** (1.26 g, 5.01 mmol), potassium *tert*-butoxide (6 mg, 0.05 mmol) in methyl acrylate (26 mL) gave the title compound as a red oil (1.90 g, 95%); ν_{max} (ATR)/cm^{-1} 1549, 1581, 1732 (ester C=O), 2926 (C-H); ^{1}H NMR (500 MHz, $CDCl_3$) 1.71-1.76 (2H, m, C(3)C(6)H_2), 1.83-1.88 (2H, m, C(3)C(5)H_2), 2.52 (2H, m, C(3)C(4)H_2), 2.61 (2H, t, *J* 6.1, C(3)C(7)H_2), 2.70 (2H, t, *J* 6.5, C(2)H_2), 3.70 (3H, s, CO_2CH_3), 4.27 (2H, t, *J* 6.1, C(3)H_2), 6.69 (1H, s, C(3)pyrrolyl(3)*H*), 7.24 (1H, d, *J* 15.1, C(3)Ar(2)C(2)*H*), 7.46-7.49 (2H, m, C(O)Ar(3,5)*H*), 7.51-7.56 (1H, m, C(O)Ar(4)*H*), 7.79 (1H, d, *J* 15.0, C(3)Ar(2)C(1)*H*), 7.99-8.01 (2H, m, C(O)Ar(2,6)*H*); ^{13}C NMR (125 MHz, $CDCl_3$) 22.6 (C(3)*C*(7)H_2), 23.1 (C(3)*C*(4)H_2), 23.1 (C(3)*C*(5)H_2), 23.4 (C(3)*C*(6)H_2), 36.0 (*C*(2)H_2), 36.9 (*C*(3)H_2), 52.2 (CO_2CH_3), 111.8 (C(3)Ar*C*(3)H), 114.8 (C(3)Ar(2)*C*(2)H), 121.1 (C(3)*C*(4*a*)), 128.3 (C(O)Ar*C*(2,6)H), 128.3 (C(3)Ar*C*(2)), 128.6 (C(O)Ar*C*(3,5)H), 132.0 (C(3)Ar(2)*C*(1)H), 132.3 (C(O)Ar*C*(4)H), 135.6 (C(3)Ar(2)*C*(2)), 139.2 (C(O)*C*(1)), 171.1 (*C*O_2Me), 189.7 (C(3)Ar(2)*C*(3)); HRMS (NSI$^+$), $C_{21}H_{23}NO_3Na$ $[M+Na]^+$, requires 360.1576, found 360.1541 (−9.7 ppm).

Methyl (*E*)-3-(2-(3-(4-bromophenyl)-3-oxoprop-1-en-1-yl)-5-chloro-1*H*-pyrrol-1-yl)propanoate

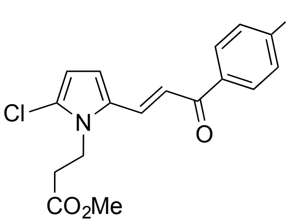

68

To a solution of pyrrolyl enone-ester **61** (2.20 g, 6.08 mmol) in CH_2Cl_2 (87 mL) at 0 °C was added *N*-chlorosuccinimide (975 mg, 7.30 mmol) portionwise. The reaction was allowed to warm to rt and stirred for 4 h. Reaction was quenched with saturated aq. $NaCO_3$, extracted with CH_2Cl_2 (×3), dried over $MgSO_4$ and concentrated under reduced pressure to crude reaction mixture. Column chromatography (EtOAc:hexane 15:85) gave the title compound as a brown oil (600 mg, 25%) ; ν_{max} (ATR)/cm^{-1} 1555, 1587, 1739 (ester C=O), 2998 (C-H); ^{1}H NMR (300 MHz, $CDCl_3$) 2.74 (2H, t, *J* 6.9, C(2)H_2), 3.71 (3H, s, CO_2CH_3), 4.42 (2H, t, *J* 6.9, C(3)H_2), 6.21 (1H, d, *J* 4.2, pyrrolyl(4)*H*), 6.83 (1H, d, *J* 4.2, pyrrolyl(3)*H*), 7.26 (1H, d, *J* 15.1, pyrrolyl(2)C(2)*H*), 7.61-7.65 (2H, m, C(O)Ar(3,5)*H*), 7.76 (1H, d, *J* 15.1, pyrrolyl(2)C(1)*H*), 7.86-7.90 (2H, m, C(O)Ar(3,5)*H*); ^{13}C NMR (75 MHz, $CDCl_3$) 35.2 (*C*(2)H_2), 39.8 (CO_2CH_3), 52.2 (*C*(3)H_2), 109.7 (pyrrolyl*C*(4)H), 112.6 (pyrrolyl*C*(3)H), 116.5 (pyrrolyl(2)*C*(2)H), 127.8 (), 129.3 (pyrrolyl*C*(2)), 129.9 (C(O)Ar*C*(2,6)H), 132.0 (pyrrolyl(2)*C*(1)H),

132.0 (C(O)Ar*C*(3,5)H), 137.4 (pyrrolyl*C*(5)), 170.6 (*C*O_2Me), 188.4 (pyrrolyl(2)*C*(3)); $C_{17}H_{18}{}^{77}BrClNO_3$ $[M+H]^+$, requires 397.9974, found 397.9970 (−1.1 ppm).

Methyl (*E*)-3-(2-(3-oxobut-1-en-1-yl)-1*H*-pyrrol-1-yl)propanoate

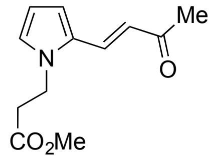

69

Following general procedure C pyrrolyl enone **58** (2.97 g, 22.0 mmol), potassium *tert*-butoxide (25 mg, 0.22 mmol) in methyl acrylate (116 mL) gave the title compound as a yellow oil (4.18 g, 90%); ν_{max} (ATR)/cm^{-1} 1174, 1627, 1740 (ester C=O), 2964 (C-H); ^{1}H NMR (300 MHz, $CDCl_3$) 2.30 (3H, s, pyrrolyl(2)C(4)*H₃*), 2.73 (2H, t, *J* 6.8, C(2)*H₂*), 3.66 (3H, s, CO_2CH_3), 4.32 (2H, t, *J* 6.8, C(3)*H₂*), 6.18 (1H, ddd, *J* 0.5, 2.7, 3.9, pyrrolyl(4)*H*), 6.50 (1H, d, *J* 15.6, pyrrolyl(2)C(2)*H*), 6.69-6.71 (1H, m, pyrrolyl(3)*H*), 6.86 (1H, dd, *J* 1.7, 2.5, pyrrolyl(5)*H*), 7.44 (1H, d, *J* 15.6, pyrrolyl(2)C(1)*H*); ^{13}C NMR (75 MHz, $CDCl_3$) 28.3 (pyrrolyl(2)*C*(4)H_3) 36.2 (*C*(2)H_2), 42.5 (CO_2CH_3), 52.1 (*C*(3)H_2), 110.3 (pyrrolyl*C*(4)H), 112.7 (pyrrolyl*C*(3)H), 122.0 (pyrrolyl(2)*C*(2)H), 126.9 (pyrrolyl*C*(5)H), 130.2 (pyrrolyl*C*(2)), 171.1 (*C*O_2Me), 197.7 (pyrrolyl(2)*C*(3)); $C_{12}H_{16}NO_3$ $[M+H]^+$, requires 222.1125, found 222.1123 (−0.9 ppm).

1.3.4 Hydrolysis of Pyrrolyl Enone-Esters

(*E*)-3-(2-(3-Oxo-3-phenylprop-1-en-1-yl)-1*H*-pyrrol-1-yl)propanoic acid

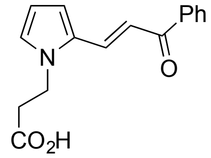

11

Following general procedure D, pyrrole enone ester **60** (8.00 g, 28.4 mmol) and LiOH (3.57 g, 85.2 mmol) in H_2O/ethanol (105 mL:105 mL) gave the title compound as a brown solid (4.96 g, 65%); mp 103-104 °C; ν_{max} (ATR)/cm^{-1} 1641 (C=O), 1732 (C=O), 3157 (C-H); ^{1}H NMR (500 MHz, $CDCl_3$) 2.85 (2H, t, *J* 7.1, C(2)*H₂*), 4.41 (2H, t, *J* 7.1, C(3)*H₂*), 6.25-6.26 (1H, m, pyrrolyl(4)*H*), 6.88 (1H, dd, *J* 1.3, 3.9, pyrrolyl(3)*H*), 6.94 (1H, m, pyrrolyl(5)*H*), 7.35 (1H, d, *J* 15.1, pyrrolyl(2)C(2)*H*), 7.48-7.51 (2H, m, C(O)Ar(3,5)*H*), 7.56-7.59 (1H, C(O)Ar(4)*H*), 7.86 (1H, d, *J* 15.1, pyrrolyl(2)C(1)*H*), 8.01-8.03 (2H, C(O)Ar(2,6)*H*); ^{13}C NMR (100 MHz, $CDCl_3$) 36.1 (*C*(2)H_2), 42.5 (*C*(3)H_2), 52.2 (CO_2CH_3), 110.6 (pyrrolyl*C*(4)H), 113.1 (pyrrolyl*C*(3)H), 116.9 (pyrrolyl(2)*C*(2)H), 127.4 (pyrrolyl*C*(5)H), 128.5 (C(O)Ar*C*(2,6)H), 128.7 (C(O)Ar*C*(3,5)H), 129.5 (pyrrolyl*C*(2)), 132.4 (pyrrolyl(2)*C*(1)H), 132.8 (C(O)Ar*C*(4)H), 138.7 (C(O)Ar*C*(1)), 174.5 (*C*O_2H), 190.3 (pyrrolyl(2)*C*(3)); HRMS (NSI+), $C_{16}H_{15}NO_3Na$ $[M+Na]^+$, requires 292.0944, found 292.0947 (+1.0 ppm).

(*E*)-3-(2-(3-(4-Bromophenyl)-3-oxoprop-1-en-1-yl)-1*H*-pyrrol-1-yl)propanoic acid

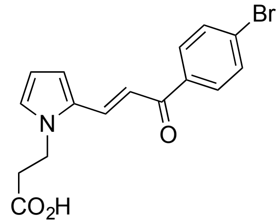

12

Following general procedure D, pyrrole enone ester **61** (4.11 g, 11.4 mmol) and LiOH (1.44 g, 34.2 mmol) in H_2O/ethanol (48 mL:48 mL) gave the title compound as a yellow solid (3.57 g, 90%); mp 110-112 °C; ν_{max} (ATR)/cm^{-1} 1636 (C=O), 1726 (C=O), 3059 (C-H); ^{1}H NMR (500 MHz, d_6-DMSO) 2.69 (2H, t, *J* 6.9, C(2)*H*), 4.33 (2H, t, *J* 6.9, C(3)*H*), 6.20-6.22 (1H, m, pyrrolyl(4)*H*), 7.10 (1H, dd, *J* 4.0, 1.5, pyrrolyl(3)*H*), 7.14-7.16 (1H, m, pyrrolyl(5)*H*), 7.54 (1H, d, *J* 15.1, pyrrolyl(2)C(2)*H*), 7.73-7.78 (3H, m, pyrrolyl(2)C(1)*H* and C(O)Ar(3,5)*H*), 8.02-8.06 (2H, m, C(O)Ar(2,6)*H*), 12.4 (1H, br. s, O*H*); ^{13}C NMR (125 MHz, d_6-DMSO) 36.0 (*C*(2)H_2), 42.0 (*C*(3)H_2), 110.0 (pyrrolyl*C*(4)H), 113.6 (pyrrolyl*C*(3)H), 115.7 (pyrrolyl(2)*C*(2)H), 128.0 (C(O)Ar*C*(4)), 130.0 (pyrrolyl*C*(2)), 130.2 (C(O)Ar*C*(2,6)H), 131.7 (C(O)Ar*C*(3,5)H), 132.40 (pyrrolyl(2)*C*(1)H), 137.2 (C(O)Ar*C*(1)), 172.0 (*C*O_2H), 187.3 (pyrrolyl(2)*C*(3)); HRMS (NSI$^+$), $C_{16}H_{15}{}^{79}BrNO_3$ $[M+H]^+$, requires 348.0230, found 348.0233 (+0.9 ppm).

(*E*)-3-(2-(3-(3-Bromophenyl)-3-oxoprop-1-en-1-yl)-1*H*-pyrrol-1-yl)propanoic acid

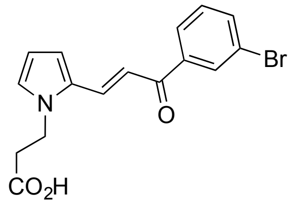

13

Following general procedure D, pyrrole enone ester **62** (1.40 g, 3.87 mmol) and LiOH (487 mg, 11.6 mmol) in H_2O/ethanol (14 mL:14 mL) gave the title compound as a yellow solid (1.07 g, 81%); mp 138-140 °C; ν_{max} (ATR)/cm^{-1} 1584, 1728 (C=O), 3096 (C-H); ^{1}H NMR (400 MHz, d_6-DMSO) 2.69 (2H, t, *J* 6.9, C(2)*H_2*), 4.33 (2H, t, *J* 6.9, C(3)*H_2*), 6.21-6.22 (1H, m, pyrrolyl(4)*H*), 7.14-7.16 (2H, m, pyrrolyl(3)*H* and pyrrolyl(5)*H*), 7.50-7.57 (2H, m, pyrroly(2)C(2)*H* and C(O)Ar(5)*H*), 7.77 (1H, d, *J* 15.0, pyrrolyl(2)C(2)*H*), 7.83 (1H, dd, *J* 1.1, 8.0, C(O)Ar(5)*H*), 8.09 (1H, dt, *J* 1.1, 7.8, C(O)Ar(4)*H*), 8.23 (1H, t, *J* 1.8, C(O)Ar(2)*H*); ^{13}C NMR (125 MHz, d_6-DMSO) 36.0 (*C*(2)H), 42.1 (*C*(3)H), 110.0 (pyrrolyl(4)*H*), 113.9 (pyrrolyl(3)*H*), 115.6 (pyrrolyl(2)C(2)*H*), 122.3 (C(O)Ar*C*(3)), 127.2 (C(O)Ar*C*(6)H), 128.2 (C(O)Ar*C*(6)H), 128.9 (C(O)Ar*C*(5)H), 130.6 (CO)Ar*C*(2)H), 130.9 (pyrrolyl*C*(2)), 132.7 (C(O)Ar*C*(4)H), 135.2 (pyrrolyl(2)*C*(1)H), 140.3 (C(O)Ar*C*(1)), 172.0 (*C*O_2H), 186.9 (pyrrolyl(2)*C*(3)); HRMS (NSI$^+$), $C_{16}H_{15}{}^{79}BrNO_3$ $[M+H]^+$, requires 276.0501, found 276.9886 (+0.9 ppm).

(*E*)-3-(2-(3-oxo-3-(Thiophen-2-yl)prop-1-en-1-yl)-1*H*-pyrrol-1-yl)propanoic acid

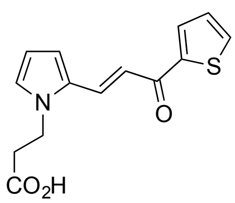

14

Following general procedure D, pyrrole enone ester **63** (1.28 g, 4.42 mmol) and LiOH (319 mg, 13.3 mmol) in H_2O/ethanol (16 mL:16 mL) gave the title compound as a yellow solid (1.12 g, 92%); mp 122-124 °C; ν_{max} (ATR)/cm^{-1} 1558, 1627 (C=O), 1726 (C=O), 2910 (C-H); ^{1}H NMR (400 MHz, $CDCl_3$) 2.84 (2H, t, *J* 7.2, C(2)*H₂*), 4.33 (2H, t, *J* 6.9, C(3)*H₂*), 6.24-6.26 (1H, dd, *J* 2.7, 3.5, pyrrolyl(4)*H*), 6.88 (1H, dd, *J* 1.2, 4.0, pyrrolyl(3)*H*), 6.94 (1H, dd, 1.6, 2.5, pyrrolyl(5)*H*), 7.16-7.22 (2H, m, pyrrolyl(2)C(2)*H* and C(O)thienyl(4)*H*), 7.66 (1H, dd, *J* 1.1, 4.9, C(O)thienyl(3)*H*), 7.84-7.88 (2H, m, C(O)thienyl(5)*H* and pyrrolyl(2)C(1)*H*); ^{13}C NMR (100 MHz, $CDCl_3$) 36.1 (*C*(2)H_2), 42.5 (*C*(3)H_2), 110.7 (pyrrolyl*C*(4)H), 113.1 (pyrrolyl*C*(3)H), 116.8 (pyrrolyl(2)*C*(2)H), 127.5 (pyrrolyl*C*(5)H), 128.4 (C(O)thienyl*C*(4)H), 129.3 (pyrrolyl*C*(2)), 131.6 (C(O)thienyl*C*(5)H), 131.7 (pyrrolyl(2)*C*(1)H), 133.7 (C(O)thienyl*C*(3)H), 146.0 (C(O)thienyl*C*(2)), 174.6 (*C*O_2H), 182.3 (pyrrolyl(2)*C*(3)); HRMS (NSI$^-$), $C_{14}H_{12}NO_3S$ [M−H]$^-$, requires 274.0543, found 274.0536 (−2.7 ppm).

(*E*)-3-(2-(3-(4-Methoxyphenyl)-3-oxoprop-1-en-1-yl)-1*H*-pyrrol-1-yl)propanoic acid

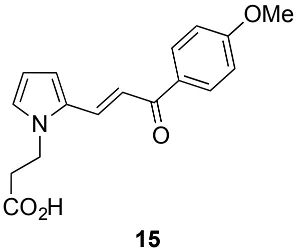

15

Following general procedure D, pyrrole enone ester **64** (2.43 g, 7.75 mmol) and LiOH (978 mg, 23.3 mmol) in H_2O/ethanol (29 mL:29 mL) gave the title compound as a brown solid (1.90 g, 82%); mp 147-148 °C; ν_{max} (ATR)/cm^{-1} 1634 (C=O), 1730, (C=O), 3084 C-H); ^{1}H NMR (500 MHz, d_6-DMSO) 2.69 (2H, t, *J* 6.9, C(2)*H₂*), 3.86 (3H, s, OC*H₃*), 4.33 (2H, t, *J* 6.9, C(3)*H₂*), 6.18-6.20 (1H, m, pyrrolyl(4)*H*), 7.04-7.08 (3H, m, pyrrolyl(3)*H* and C(O)Ar(3,5)*H*), 7.11-7.12 (1H, m, pyrrolyl(5)*H*), 7.56 (1H, d, *J* 15.1, pyrrolyl(2)C(2)*H*), 7.70 (1H, d, *J* 15.1, pyrrolyl(2)C(1)*H*), 8.07-8.12 (m, 2H, C(O)ArC(2,6)*H*), 12.38 (1H, br. s., O*H*); ^{13}C NMR (125 MHz, *d_6*-DMSO) 36.0 (*C*(2)H_2), 42.1 (*C*(3)H_2), 55.5 (O*C*H_3), 109.6 (pyrrolyl*C*(4)H), 112.9 (pyrrolyl*C*(3)H), 113.9 (C(O)Ar*C*(3,5)H), 116.3 (pyrrolyl(2)*C*(2)H), 127.3 (pyrrolyl*C*(5)H), 128.94 (pyrrolyl*C*(2)), 130.5 (C(O)Ar*C*(2,6)H) 131.0 (C(O)Ar*C*(1)), 131.1 (pyrrolyl(2)*C*(1)H), 162.8 (C(O)Ar*C*(1)), 172.0 (*C*(1)), 186.7

(pyrrolylC(2)*C*(3)); HRMS (NSI^+), $C_{17}H_{16}NO_4$ $[M-H]^-$, found 298.1085, requires 298.1081 (−1.3 ppm).

(*E*)-3-(2-(3-(Naphthalen-2-yl)-3-oxoprop-1-en-1-yl)-1*H*-pyrrol-1-yl)propanoic acid

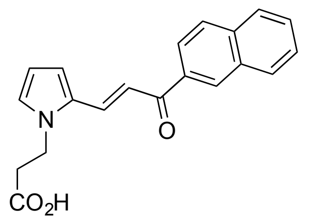

16

Following general procedure D, pyrrolyl enone ester **65** (2.53 g, 7.57 mmol) and LiOH (952 mg, 22.7 mmol) in H_2O/ethanol (28 mL:28 mL) gave the title compound as a brown solid (1.35 g, 56%); mp 120-122 °C; ν_{max} (ATR)/cm^{-1} 1556, 1728 (C=O), 3047 (C-H); ^{1}H NMR (400 MHz, d_6-DMSO) 2.72 (2H, t, *J* 6.9, C(2)H_2), 4.36 (2H, t, *J* 6.9, C(3)H_2), 6.23-6.24 (1H, m, pyrrolyl(4)*H*), 7.12-7.16 (2H, m, pyrrolyl(3)*H* and pyrrolyl(5)*H*), 7.61-7.69 (2H, m, Ar*H*), 7.74 (1H, d, *J* 15.1, pyrrolyl(2)C(2)*H*), 7.81 (1H, d, *J* 15.1, pyrrolyl(2)C(1)*H*), 7.96-8.05 (2H, m, Ar*H*), 8.10-8.16 (2H, m, Ar*H*), 8.83 (1H, s, Ar*H*), 12.4 (1H, br. s, CO_2H); ^{13}C NMR (100 MHz, d_6-DMSO) 36.0 (*C*(2)H_2), 42.1 (*C*(3)H_2), 109.8 (pyrrolyl*C*(4)H), 113.3 (pyrrolyl*C*(3)H), 116.3 (pyrrolyl(2)*C*(2)H), 124.2 (C(O)Ar*C*H), 126.9 (C(O)Ar*C*H), 127.7 (C(O)Ar*C*H), 127.7 (C(O)Ar*C*H), 128.3 (C(O)Ar*C*H), 128.4 (pyrrolyl*C*(5)H), 129.0 (C(O)Ar*C*H), 129.5 (C(O)Ar*C*H), 129.6 (pyrrolyl*C*(2)), 131.8 (C(O)Ar*C*), 132.4 (pyrrolyl(2)*C*(1)H), 134.9 (C(O)Ar*C*), 135.5 (C(O)Ar*C*), 172.0 (*C*O_2H), 188.2 (pyrrolyl(2)*C*(3)); HRMS (NSI-), $C_{20}H_{16}NO_3$ $[M-H]^-$, requires 318.1136, found 318.1131 (−1.5 ppm).

(*E*)-3-(2-(3-(Naphthalen-1-yl)-3-oxoprop-1-en-1-yl)-1*H*-pyrrol-1-yl)propanoic acid

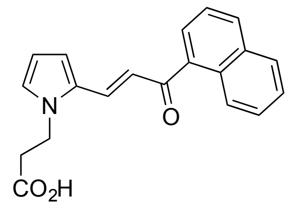

17

Following general procedure D, pyrrolyl enone ester **66** (1.68 g, 5.04 mmol) and LiOH (634 mg, 15.1 mmol) in H_2O/ethanol (19 mL:19 mL) gave the title compound as a crude residue of 80% purity; ^{1}H NMR (400 MHz, d^6-DMSO) 2.65 (2H, t, *J* 6.9, C(2)H_2), 4.24 (2H, t, *J* 6.9, C(3)H_2), 6.18-6.19 (1H, m, pyrrolyl(4)*H*), 6.98-6.98 (1H, m, pyrrolyl(3)*H*), 7.13-7.14 (1H, m, pyrrolyl(5)*H*), 7.17 (1H, d, *J* pyrrolyl(2)C2)*H*), 7.55-7.63 (5H, m, C(O)Ar*H* ×3 and pyrrolyl(2)C(1)*H*), 7.88-7.89 (1H, m, C(O)Ar*H*), 8.02-8.03 (1H, m, C(O)Ar*H*), 8.10-8.11 (1H, m, C(O)Ar*H*), 8.29-8.30 (1H, m, C(O)Ar*H*) 12.3 (1H, br. s, CO_2H). The crude residue was carried forward immediately into the next step without further characterisation.

(*E*)-3-(2-(3-oxo-3-phenylprop-1-en-1-yl)-4,5,6,7-tetrahydro-1*H*-indol-1-yl)propanoic acid

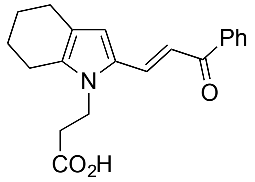

18

Following general procedure D, pyrrolyl enone ester **67** (1.90 g, 5.63 mmol) and LiOH (709 mg, 16.9 mmol) in H_2O/ethanol (21 mL:21 mL) gave the title compound as a crude residue of 80% purity; ^1H NMR (400 MHz, d^6-DMSO) 1.74-1.77 (2H, m, indolyl(6)H_2), 1.81-1.87 (2H, m, indolyl(5)H_2), 2.50-2.53 (2H, m, indolyl(4)H_2), 2.59-2.62 (2H, m, indolyl(7)H_2), 2.70 (2H, t, *J* 6.8, C(2)H_2), 4.26 (2H, t, *J* 6.8, C(3)H_2), 6.69 (1H, s, indolyl(3)*H*), 7.23 (1H, d, *J* 14.9, indolyl(2)C(2)*H*), 7.44-7.54 (3H, m, C(O)Ar(3,5)*H* and C(O)Ar(4)*H*), 7.79 (1H, d, *J* 14.9, indolyl(2)C(1)*H*), 7.98-8.00 (2H, m, C(O)Ar(2,6)*H*). The crude residue was carried forward immediately into the next step without further characterisation.

(*E*)-3-(2-(3-(4-bromophenyl)-3-oxoprop-1-en-1-yl)-5-chloro-1*H*-pyrrol-1-yl)propanoic acid

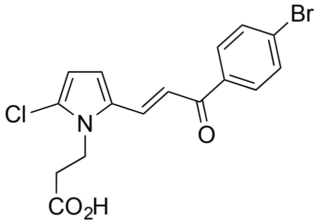

19

Following general procedure D, pyrrolyl enone ester **68** (496 mg, 1.24 mmol) and LiOH (156 mg, 3.72 mmol) in H_2O/ethanol (4.6 mL:4.6 mL) gave the title compound as a brown solid (327 mg, 69%); mp 160-162 °C; ν_{max} (ATR)/cm^{-1} 1558, 1575, 1641 (C=O), 1741 (C=O), 3086 (C-H); ^1H NMR (300 MHz, $CDCl_3$) 2.79 (2H, t, *J* 6.9, C(2)H_2), 4.46 (2H, t, *J* 6.9, C(3)H_2), 6.23 (1H, d, *J* 4.2, pyrrolyl(4)*H*), 6.87 (1H, d, *J* 4.1, pyrrolyl(3)*H*), 7.28 (1H, d, *J* 15.1, pyrrolyl(2)C(2)*H*), 7.63 (2H, d, *J* 8.5, C(O)Ar(3,5)*H*), 7.85-7.90 (3H, m, pyrrolyl(2)C(1)*H* and C(O)Ar(2,6)*H*); ^{13}C NMR (125 MHz, $CDCl_3$) 34.8 (*C*(2)H_2), 39.5 (*C*(3)H_2), 109.8 (pyrrolyl*C*(4)H), 112.8 (pyrrolyl*C*(3)H), 116.3 (pyrrolyl(2)*C*(2)H), 122.5 (C(O)Ar*C*(4)), 128.0 (C(O)Ar*C*(1)), 129.4 (), 130.0 (C(O)Ar*C*(3,5)H), 132.0 (C(O)Ar*C*(2,6)H), 132.7 (pyrrolyl(2)*C*(1)H), 137.2 (pyrrolyl*C*(5)), 173.1 (*C*O_2H), 188.9 (pyrrolyl(2)*C*(3)O); HRMS (NSI$^-$), $C_{16}H_{12}Br^{79}Cl^{35}NO_3$ $[M-H]^-$, requires 379.9694, found 379.9695 (0.0 ppm).

(*E*)-3-(2-(3-Oxobut-1-en-1-yl)-1*H*-pyrrol-1-yl)propanoic acid

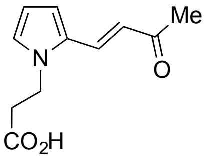

20

Following general procedure D, pyrrolyl enone ester **69** (4.41 g, 19.9 mmol) and LiOH (2.51 g, 59.7 mmol) in H_2O/ethanol (74 mL:74 mL) gave the title compound as a brown solid (1.12 g, 92%); mp 122-124 °C; ν_{max} (ATR)/cm^{-1} 1591, 1714 (C=O), 2906 (C-H); ^{1}H NMR (500 MHz, $CDCl_3$) 2.27 (3H, s, C(O)C*H₃*), 2.65 (2H, t, *J* 6.8, C(2)*H₂*), 4.30 (2H, d, *J* 6.8 C(3)*H₂*), 6.13-6.15 (1H, m, pyrrolyl(4)*H*), 6.45 (1H, d, *J* 15.6, pyrrolyl(2)C(2)*H*), 6.77 (1H, dd, *J* 1.4, 3.9, pyrrolyl(3)*H*), 7.06-7.07 (1H, m, pyrrolyl(5)*H*), 7.53 (1H, d, *J* 15.9, pyrrolyl(2)C(1)*H*); ^{13}C NMR (125 MHz, $CDCl_3$) 27.1 (C(O)*C*H_3), 36.2 (*C*(2)H_2), 42.1 (*C*(3)H_2), 109.8 (pyrrolyl*C*(4)H), 112.1 (pyrrolyl*C*(3)H), 122.2 (pyrrolyl(2)*C*(2)H), 127.2 (pyrrolyl*C*(5)H), 128.2 (pyrrolyl*C*(2)), 131.5 (pyrrolyl(2)*C*(1)H), 172.3 (*C*O_2H), 197.5 (pyrrolyl(2)*C*(3)); $C_{11}H_{12}NO_3$ $[M-H]^-$, requires 206.0823, found 206.0823 (0.0 ppm).

1.3.5 Intramolecular Isothiourea-Catalysed Michael Addition-Lactonisation

(4a*S*,9a*R*)-3-Phenyl-4a,9a-dihydro-1*H*,9*H*-pyrano[4,3-*a*]pyrrolizin-1-one

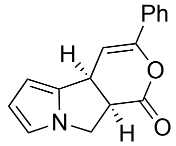

21

Pyrrolyl enone-acid **11** (50.0 mg, 0.19 mmol), *i*-Pr_2NEt (97 µL, 0.56 mmol), pivaloyl chloride (69 µL, 0.56 mmol) in CH_2Cl_2 (2 mL), (+)-BTM **24** (4.7 mg, 0.019 mmol) and *i*-Pr_2NEt (49 µL, 0.28 mmol) gave crude product (>95:5 dr). Purification by column chromatography (EtOAc:petrol ether 10:90) afforded the title compound (>95:5 dr) as a yellow oil (39 mg, 84%); $[\alpha]_D^{20}$ +52.2 (*c* 0.5 $CHCl_3$); Chiral HPLC analysis; ChiralPak AD-H (90:10 hexane:IPA, flow rate 1.0 mlmin^{-1}, 270 nm, 30 °C) t_R 14.3 (major) and t_R 16.4 (minor), >99% ee; ν_{max} (ATR)/cm^{-1} 3028 (C-H), 1728 (C=O), ^{1}H NMR (500 MHz, $CDCl_3$) 3.91 (1H, td, *J* 8.0, 5.0, C(9a)*H*), 4.27 (1H, dd, *J* 4.6, 4.3, C(4a)*H*), 4.37 (1H, dd, *J* 10.8, 8.0, C(9)*Ha*), 4.55 (1H, dd, *J* 10.8, 5.0, C(9)*Hb*), 5.90 (1H, d, *J* 4.6, C(4)*H*), 5.95 (1H, dt, *J* 3.3, 1.1, C(5)*H*), 6.27 (1H, t, *J* 3.1, C(6)*H*), 6.67 (1H, dd, *J* 2.8, 1.1, C(7)*H*), 7.36-7.42 (3H, m, C(3)ArC(3,5)*H* and C(3)ArC(4)*H*), 7.63-7.66 (2H, m, C(3)ArC(2,6)*H*); ^{13}C NMR (126 MHz, $CDCl_3$) 35.8 (*C*(4a)H), 45.2 (*C*(9a)H), 49.0 (*C*(9)H^aH^b), 99.6 (*C*(4)H), 100.0 (*C*(5)H), 113.8 (*C*(6)H), 115.2 (*C*(7)H), 125.2 (C(3)Ar*C*(2,6)H), 128.9 (C(3)Ar*C*(4), 129.7 (C(3)Ar*C*(3,5)H), 132.4 (*C*(4b)), 136.6 (C(3)Ar*C*(1)), 148.7 (*C*(3)), 168.3 (*C*(1)); HRMS (NSI$^+$), $C_{16}H_{13}NO_2Na$ $[M+Na]^+$, requires 274.0838, found 274.0843 (+1.8 ppm).

1.3.6 Intramolecular Isothiourea-Catalysed Michael Addition-Lactonisation/Ring Opening

Methyl (1*S*,2*R*)-1-(2-oxo-2-phenylethyl)-2,3-dihydro-1*H*-pyrrolizine-2-carboxylate

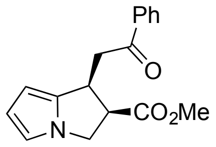

27

Following general procedure E, pyrrolyl enone-acid **11** (50 mg, 0.19 mmol), *i*-Pr_2NEt (97 µL, 0.56 mmol), pivaloyl chloride (69 µL, 0.56 mmol) in CH_2Cl_2 (2 mL), (+)-BTM **24** (2.4 mg, 0.0095 mmol) and *i*-Pr_2NEt (49 µL, 0.28 mmol) gave the crude product (>95:5 dr). Purification by column chromatography (EtOAc:petrol 10:90) gave the title compound (>95:5 dr) as a yellow oil (45 mg, 86%); $[\alpha]_D^{20}$ –54.3 (*c* 1.0 in $CHCl_3$); Chiral HPLC analysis ChiralPak AD-H (90:10 hexane:IPA, flow rate 1.0 mlmin^{-1}, 220 nm), t_R 10.6 (minor) and t_R 12.2 (major), >99% ee; ν_{max} (ATR)/cm^{-1} 2953 (C-H), 1730 (C=O), 1684 (C=O); ^{1}H NMR (400 MHz, $CDCl_3$) 3.19-3.34 (2H, m, pyrrolizine(1)C*H*^{a}H^b and pyrrolizine(1)CH^a*H*^{b}), 3.58 (3H, s, CO_2CH_3), 4.00 (1H, dd, *J* 16.2, 7.6, pyrrolizine(2)*H*), 4.15 (1H, dd, *J* 11.7, 7.6, pyrrolizine(3)*H*^{a}H^b), 4.19-4.26 (1H, m, pyrrolizine(1)*H*), 4.33 (1H, dd, *J* 11.4, 7.2, pyrrolizine(3)H^a*H*^{b}), 5.80-5.82 (1H, dt, *J* 3.4, 1.0, pyrrolizine(7)*H*), 6.18-6.22 (1H, m, pyrrolizine(6)*H*), 6.61 (1H, dd, *J* 2.6, 1.2, pyrrolizine(5)*H*), 7.43-7.49 (2H, m, C(O)ArC(3,5)*H*), 7.53-7.58 (1H, m, C(O)ArC(4)*H*), 7.94-7.90 (2H, m, C(O)ArC(2,6)*H*); ^{13}C NMR (125 MHz, $CDCl_3$) 35.3 (pyrrolizine*C*(1)H), 39.8 (pyrrolizine(1)*C*H^{a}H^b), 47.5 (pyrrolizine*C*(3)H^{a}H^b), 49.6 (pyrrolizine*C*(2)H), 51.8 (CO_2CH_3), 99.7 (pyrrolizine*C*(7)H), 112.3 (pyrrolizine*C*(6)H), 113.9 (pyrrolizine*C*(5)H), 127.8 (C(O)Ar*C*(2,6)H), 128.5 (C(O)Ar*C*(3,5)H), 133.1 (C(O)Ar*C*(4)H), 136.7 (pyrrolizine*C*(7a)), 137.6 (C(O)Ar*C*(1)), 172.1 (*C*O_2Me), 197.7 (*C*(O)Ar); HRMS (NSI$^+$), $C_{17}H_{18}NO_3$ $[M+H]^+$, requires 284.1281, found 284.1284 (+1.1 ppm).

(1*S*,2*R*)-*N*-Allyl-1-(2-oxo-2-phenylethyl)-2,3-dihydro-1*H*-pyrrolizine-2-carboxamide

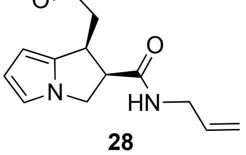

28

Following general procedure E, pyrrolyl enone-acid **11** (150 mg, 0.56 mmol), *i*-Pr_2NEt (293 µL, 1.68 mmol), pivaloyl chloride (207 µL, 1.68 mmol) in CH_2Cl_2 (5.6 mL), (+)-BTM **24** (7 mg, 0.028 mmol), *i*-Pr_2NEt (293 µL, 1.68 mmol) and allyl amine (168 µL, 2.24 mmol) gave, after purification by column chromatography (EtOAc:petrol 15:85) the title compound (>95:5 dr) as a brown oil (140 mg, 81%); $[\alpha]_D^{20}$ –41.0 (*c* 0.5 in $CHCl_3$); Chiral HPLC analysis: ChiralPak AD-H (80:20 hexane:IPA, 1.0 mlmin^{-1}, 254 nm), t_R 9.1 (minor) and t_R 11.7 (major), 98.5:1.5 er; ν_{max} (ATR)/cm^{-1} 1637(C=O), 1683 (C=O),

3290 (C-H); ^{1}H NMR (500 MHz, $CDCl_3$) 3.33 (1H, dd, *J* 5.3, pyrrolizine(1)C*H*^{a}H^{b}COAr), 3.42 (1H, dd, *J* 8.6, 18.5, pyrrolizine(1)CH^{a}*H*^{b}COAr), 3.59-3.64 (1H, m, C(O)NHC*H*^{a}H^{b}), 3.72-3.80 (2H, m, pyrrolizine(2)*H* and C(O)NHCH^{a}*H*^{b}), 4.09-4.18 (2H, m, pyrrolizine(3)*H*^{a}H^{b} and pyrrolizine(1)*H*), 4.37 (1H, dd, *J* 6.0, 10.6, pyrrolizine(3)H^{a}*H*^{b}), 4.96-5.06 (2H, m, =C*H*^{a}H^{b} and =CH^{a}*H*^{b}), 5.61 (1H, ddt, *J* 6.0, 10.2, 16.3, C*H*=CH_2), 5.79-5.80 (2H, m, pyrrolizine(7)*H* and C(O)N*H*R), 6.20 (1H, t, *J* 3.0, pyrrolizine(6)*H*), 6.61 (1H, br. s, pyrrolizine(5)*H*), 7.44-7.47 (2H, m, C(O)Ar(3,5)*H*), 7.55-7.58 (1H, m, C(O)Ar(4)*H*), 7.93-7.94 (2H, m, C(O)Ar(2,6)*H*); ^{13}C NMR (125 MHz, $CDCl_3$) 35.6 (pyrrolizine*C*(1)H), 40.3 (pyrrolizine(1)*C*H^{a}H^{b}C(O)Ar), 42.1 (C(O)NH*C*H^{a}H^{b}), 48.2 (pyrrolizine*C*(3)H^{a}H^{b}), 51.4 (pyrrolizine*C*(2)H), 99.4 (pyrrolizine*C*(7)H), 112.6 (pyrrolizine*C*(6)H), 114.1 (pyrrolizine*C*(5)H), 117.1 (=*C*H^{a}H^{b}), 128.2 (C(O)Ar*C*(2,6)H), 128.7 (C(O)Ar*C*(3,5)H), 133.5 (C(O)Ar*C*(4)H), 133.7 (*C*H=CH_2), 136.7 (pyrrolizine*C*(8)), 138.1 (C(O)Ar*C*(1)), 170.8 (*C*(O)NHR), 199.4 (*C*(O)Ar); HRMS (NSI^{+}), $C_{20}H_{21}N_2O_2$ $[M+H]^{+}$, requires 321.1598, found 321.1589 (−0.1 ppm).

1-Phenyl-2-((1*S*,2*R*)-2-(pyrrolidine-1-carbonyl)-2,3-dihydro-1*H*-pyrrolizin-1-yl)ethan-1-one

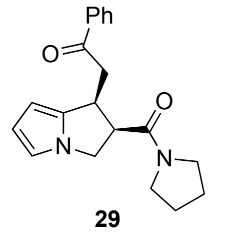

29

Following general procedure E, pyrrolyl enone-acid **11** (100 mg, 0.37 mmol), *i*-Pr_2NEt (195 μL, 1.12 mmol), pivaloyl chloride (138 μL, 1.12 mmol) in CH_2Cl_2 (3.7 mL), (+)-BTM **24** (4 mg, 0.019 mmol), *i*-Pr_2NEt (65 μL, 0.37 mmol) and pyrrolidine (126 μL, 1.51 mmol) gave, after purification by column chromatography (EtOAc:petrol 15:85) to afford the title compound as a yellow oil (119 mg, quant.,); $[\alpha]_D^{20}$ −83.0 (*c* 1.0 in $CHCl_3$); Chiral HPLC analysis ChiralPak IB (90:10 hexane:IPA, 1.0 mlmin^{-1}, 211 nm), t_R 13.0 (major) and t_R 15.6 (minor), >99% ee; ν_{max} (ATR)/cm^{-1} 1610 (C=O), 1682 (C=O), 2951 (C-H); ^{1}H NMR (500 MHz, $CDCl_3$) 1.75 (2H, p, *J* 7.0, C(O)NC(3)*H*$_2$), 1.90-2.04 (2H, m, C(O)NC(4)*H*$_2$), 2.97-3.04 (2H, m, ArC(O)C*H*^{a} and C(O)NC(2)*H*^{a}), 3.29-3.36 (1H, m, C(O)NC(2)*H*^{b}), 3.46-3.52 (1H, m, C(O)NC(5)*H*^{a}), 3.56 (1H, dd, *J* 9.8, 8.6, ArC(O)C*H*^{b}), 3.69-3.74 (1H, m, C(O)NC(5)*H*^{b}), 4.01-4.10 (2H, m, C(1)*H* and C(3)*H*^{a}), 4.24-4.29 (1H, m, C(2)*H*), 4.49-4.56 (1H, m, C(3)*H*^{b}), 5.80-5.82 (1H, m, C(7)*H*), 6.21 (1H, t, *J* 3.0, C(6)*H*), 6.62 (1H, dd, *J* 2.7, 1.3, C(5)*H*), 7.41-7.45 (2H, m, C(O)ArC(3,5)*H*), 7.51-7.55 (1H, m, C(O)ArC(4)*H*), 7.89-7.91 (2H, m, C(O)ArC(2,6)*H*); ^{13}C NMR (125 MHz, $CDCl_3$) 24.3 (C(O)N*C*(3)H_2), 26.1 (C(O)N*C*(4)H_2), 34.2 (*C*(2)H), 40.6 (ArC(O)*C*H^{a}H^{b}), 46.0 (C(O)N*C*(2)H^{a}H^{b}), 46.7 (C(O)N*C*(5)H^{a}H^{b}), 48.2 (*C*(3)H^{a}H^{b}), 49.4 (*C*(1)H), 99.3 (*C*(7)H), 112.4 (*C*(6)H), 114.1 (*C*(5)H), 128.2 (C(O)Ar*C*(2,6)H), 128.7 (C(O)Ar*C*(3,5)H), 133.2 (C(O)Ar*C*(4)H), 136.9 (*C*(7a)), 138.6 (C(O)Ar*C*(1)), 168.6 (*C*O$_2$NR), 198.3 (*C*(O)Ar); HRMS (NSI^{+}), $C_{20}H_{23}N_2O_2$ $[M+H]^{+}$, requires 323.1754, found 323.1754 (+0.0 ppm).

2-((1*S*,2*R*)-2-(Morpholine-4-carbonyl)-2,3-dihydro-1*H*-pyrrolizin-1-yl)-1-phenylethan-1-one

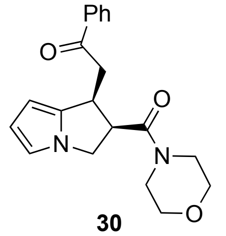

30

Following general procedure E, pyrrolyl enone-acid **11** (150 mg, 0.56 mmol), *i*-Pr_2NEt (293 μL, 1.68 mmol), pivaloyl chloride (207 μL, 1.68 mmol) in CH_2Cl_2 (5.6 mL), (+)-BTM **24** (7 mg, 0.028 mmol) and *i*-Pr_2NEt (98 μL, 0.56 mmol) gave, after purification by column chromatography (EtOAc:petrol 10:90) the title compound (>95:5 dr) as a brown oil (29 mg, 66%); $[\alpha]_D^{20}$ −26.2 (*c* 0.5 in $CHCl_3$); Chiral HPLC analysis: ChiralPak IA (80:20 hexane:IPA, 1.0 mlmin^{-1}, 220 nm), t_R 13.3 (major) and t_R 16.6 (minor), >99:1 er; ν_{max} (ATR)/cm^{-1} 1633 (C=O), 1681 (C=O), 2974, 3064 (C-H); ^{1}H NMR (500 MHz, $CDCl_3$) 3.04 (1H, dd, *J* 4.8, 18.1, pyrrolizine(2)*H*), 3.32-3.39 (1H, dd, *J* 6.8, 10.3, pyrrolizine(1)CH^aH^b), 3.41-3.48 (3H, m, pyrrolizine(1)CH^aH^b and 2×C(O)NCH^aH^b), 3.55-3.59 (2H, m, 2×C(O)NCH^aH^b), 3.63-3.73 (2H, 2×C(O)CH_2CH^aH^bO), 3.79-3.82 (1H, m, pyrrolizine(1)*H*), 4.07 (1H, dd, *J* 7.4, 10.1, pyrrolizine(3)H^aH^b), 4.13-4.19 (2H, m, 2×C(O)CH_2CH^aH^bO), 4.52-4.55 (1H, m, pyrrolizine(3)H^aH^b), 5.80 (1H, d, *J* 2.8, pyrrolizine(7)*H*), 6.20 (1H, t, *J* 2.8, pyrrolizine(6)*H*), 6.61 (1H, br. s, pyrrolizine(5)*H*), 7.42-7.45 (2H, m, C(O)Ar(3,5)*H*), 7.53-7.56 (1H, m, C(O)Ar(4)*H*), 7.91 (2H, d, *J* 7.5, C(O)Ar(2,6)*H*); ^{13}C NMR (125 MHz, $CDCl_3$) 34.9 (pyrrolizine*C*(1)H), 40.3 (pyrrolizine(1)*C*H^{a}H^{b}COAr), 42.1 (pyrrolizine*C*(2)H), 46.2 (C(O)N$C^a$$H_2$), 47.2 (C(O)N$C^b$$H_2$), 48.4 (pyrrolizine*C*(3)H^{a}H^b), 66.4 (C(O)N$CH_2$$C^a$$H_2$O), 66.6 (C(O)N$CH_2$$C^b$$H_2$O), 99.5 (pyrrolizine*C*(7)H), 112.4 (pyrrolizine*C*(6)H), 114.2 (pyrrolizine*C*(5)H), 128.2 (C(O)Ar*C*(2,6)H), 128.7 (C(O)Ar*C*(3,5)H), 133.4 (C(O)Ar*C*(4)H), 136.7 (pyrrolizine*C*(8)), 138.2 (C(O)Ar*C*(1)), 169.0 (*C*(O)NR), 198.0 (*C*(O)Ar); HRMS (NSI$^+$), $C_{20}H_{22}N_2O_3Na$ $[M+H]^+$, requires 361.1523, found 361.1522 (−0.2 ppm).

***Tert*-butyl 4-((1*S*,2*R*)-1-(2-oxo-2-phenylethyl)-2,3-dihydro-1*H*-pyrrolizine-2-carbonyl)piperazine-1-carboxylate**

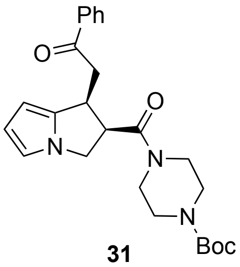

31

Following general procedure E, pyrrolyl enone-acid **11** (100 mg, 0.37 mmol), *i*-Pr_2NEt (193 μL, 1.11 mmol), pivaloyl chloride (137 μL, 1.11 mmol) in CH_2Cl_2 (3 mL), (+)-BTM **24** (4 mg, 0.019 mmol), *i*-

Pr_2NEt (293 μL, 1.68 mmol) and *N*-Boc piperazine (276 mg, 1.48 mmol) gave, after purification by column chromatography (EtOAc:petrol 20:80) the title compound (>95:5 dr) as a brown oil (121 mg, 75%); $[\alpha]_D^{20}$ –31.6 (*c* 0.5 in $CHCl_3$); Chiral HPLC analysis: ChiralPak AD-H (90:10 hexane:IPA, 1.0 mlmin^{-1}, 211 nm), t_R 23.0 (major) and t_R 30.3 (minor), >99:1 er; ν_{max} (ATR)/cm^{-1} 1163, 1633 (C=O), 1681 (C=O), 2927, 2974 (C-H); ^{1}H NMR (300 MHz, $CDCl_3$) 1.47 (9H, s, C(C*H₃*)₃), 2.99-3.06 (1H, m, pyrrolizine(2)*H*), 3.25-3.31 (3H, m, pyrrolizine(1)CH^aH^bCOAr and BocNC*H₂*), 3.40-3.70 (6H, m, 2×NC*H₂* and BocNC*H₂*), 4.04-4.20 (3H, m, pyrrolizine(3)H^aH^b and pyrrolizine(1)*H* and pyrrolizine(1)CH^aH^bCOAr), 4.50-4.56 (1H, m, pyrrolizine(3)H^aH^b); ^{13}C NMR (75 MHz, $CDCl_3$) 28.5 (C(*C*H₃)₃), 35.0 (pyrrolizine*C*(1)H), 40.4 (pyrrolizine*C*(2)H), 41.6 (N*C*H₂), 45.7 (BocN*C*H₂), 47.5 (pyrrolizine(1)*C*H^aH^bCOAr), 48.4 (pyrrolizine*C*(3)H₂), 80.4 (*C*(CH₃)₃), 99.5 (pyrrolizine*C*(7)H), 112.4 (pyrrolizine*C*(6)H), 114.3 (pyrrolizine*C*(5)H), 128.2 (C(O)Ar*C*(2,6)H), 128.2 (C(O)Ar*C*(3,5)H), 133.4 (C(O)Ar*C*(4)H), 136.8 (pyrrolizine*C*(7a)), 138.2 (C(O)Ar*C*(1)), 154.6 (N*C*(O)C(CH₃)₃), 168.9 (*C*O₂Me), 198.1 (*C*(O)Ar); HRMS (NSI$^+$), $C_{25}H_{32}N_3O_4$ [M+CH₂+H]$^+$, requires 438.2387, found 438.2394 (1.5 ppm).

1-Phenyl-2-((1*S*,2*R*)-2-(4,5,6,7-tetrahydrothieno[2,3-*c*]pyridine-6-carbonyl)-2,3-dihydro-1*H*-pyrrolizin-1-yl)ethan-1-one

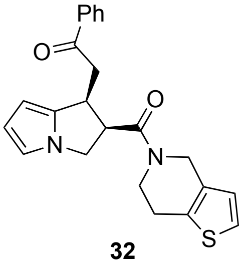

32

Following general procedure E, pyrrolyl enone-acid **11** (100 mg, 0.37 mmol), *i*-Pr_2NEt (193 μL, 1.11 mmol), pivaloyl chloride (137 μL, 1.11 mmol) in CH_2Cl_2 (3 mL), (+)-BTM **24** (4 mg, 0.019 mmol), *i*-Pr_2NEt (293 μL, 1.68 mmol) and 4,5,6,7-tetrahydrothieno[3,2-c] pyridine (260 mg, 1.48 mmol) gave, after purification by column chromatography (EtOAc:petrol 15:85) the title compound (90:10 dr, 50:50 rotameric mixture) as a brown oil (87 mg, 60%); $[\alpha]_D^{20}$ –50.6 (*c* 0.5 in $CHCl_3$); Chiral HPLC analysis: ChiralPak AD-H (90:10 hexane:IPA, 1.0 mlmin^{-1}, 220 nm), t_R 35.3 (major) and t_R 46.5 (minor), >99:1 er; ν_{max} (ATR)/cm^{-1} 1660 (C=O), 1734 (C=O), 2951, 3089 (C-H); data for single rotamer; ^{1}H NMR (500 MHz, $CDCl_3$) 2.64 (1H, dt, *J* 5.2, 16.0, C*H*), 2.95-2.99 (1H, m, C*H*), 3.03-3.12 (2H, m, 2×C*H*), 3.57 (1H, ddd, *J* 4.7, 7.4, 12.6, C*H*), 3.90 (1H, dt, *J* 5.1, 12.8, C*H*), 4.05-4.13 (4H, m, 4×C*H*), 4.57-4.64 (2H, m, 4×C*H*), 5.83-5.85 (1H, m, pyrrolizine(7)*H*), 6.22 (1H, t, *J* 3.0, pyrrolizine(6)*H*), 6.64-6.65 (1H, m, pyrrolizine(5)*H*), 6.74 (1H, d, *J* 5.2, thienyl(3)*H*), 7.14 (1H, d, *J* 5.2, thienyl(2)*H*), 7.40-7.44 (2H, m , Ar(3,5)*H*), 7.51-7.56 (1H, m, Ar(4)*H*), 7.82-7.83 (2H, m, Ar(2,6)*H*); ^{13}C NMR (125 MHz, $CDCl_3$) 24.7 (*C*H), 34.9 (*C*H), 40.2 (*C*H), 43.0 (*C*H), 46.0 (*C*H),

48.1 (*C*H), 48.4 (*C*H), 99.6 (pyrrolizine*C*(7)H), 112.4 (pyrrolizine*C*(6)H), 114.2 (pyrrolizine*C*(5)H), 123.7 (thienyl*C*(2)H), 124.7 (thienyl*C*(3)H), 128.1 (Ar*C*H), 128.7 (Ar*C*H), 132.4 (Ar*C*), 133.3 (Ar*C*), 134.3 (Ar*C*H), 136.7 (Ar*C*), 138.3 (Ar*C*), 169.1 (*C*(O)NR), 197.9 (*C*(O)Ar); HRMS (NSI$^+$), $C_{22}H_{21}N_2O_2S$ $[M+H]^+$, requires 377.1318, found 377.1295 (−6.1 ppm).

Methyl (1*S*,2*R*)-1-(2-(4-bromophenyl)-2-oxoethyl)-2,3-dihydro-1*H*-pyrrolizine-2-carboxylate

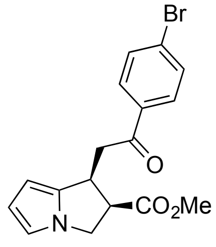

33

Following general procedure E pyrrolyl enone-acid **12** (60 mg, 0.17 mmol), *i*-Pr_2NEt (90 μL, 0.52 mmol), pivaloyl chloride (64 μL, 0.52 mmol) in CH_2Cl_2 (2 mL), (+)-BTM **24** (2.2 mg, 0.009 mmol) and *i*-Pr_2NEt (45 μL, 0.24 mmol) gave the crude product (>95:5 dr). Purification by column chromatography (EtOAc:petrol 10:90) gave the title compound (>95:5 dr) as a yellow oil (49 mg, 78%); $[\alpha]_D^{20}$ −50.8 (*c* 0.5 in $CHCl_3$); Chiral HPLC analysis: ChiralPak AD-H (90:10 hexane:IPA, 1.0 mlmin^{-1}, 254 nm), t_R 14.5 (minor) and t_R 17.4 (major), >99% ee; ν_{max} (ATR)/cm^{-1} 1689 (C=O), 1732 (C=O), 2889, 2951 (C-H); ^{1}H NMR (400 MHz, $CDCl_3$) 3.14-3.30 (2H, m, pyrrolizine(1)CH^aH^{b}C(O)Ar and pyrrolizine(1)CH^aH^bC(O)Ar), 3.59 (3H, s, CO_2CH_3), 3.98 (1H, dd, *J* 8.1, 7.8, pyrrolizine(2)*H*), 4.12-4.23 (2H, m, pyrrolizine(3)H^aH^b and pyrrolizine(1)*H*)), 4.32 (1H, dd, *J* 10.7, 7.4, pyrrolizine(3)H^aH^b), 5.78-5.81 (1H, m, pyrrolizine(7)*H*), 6.19 (1H, t, *J* 3.0, pyrrolizine(6)*H*), 6.60 (1H, dd, *J* 2.8, 1.2, pyrrolizine(5)*H*), 6.57-6.61 (2H, m, C(O)ArC(3,5)*H*), 7.76-7.81 (2H, m, C(O)ArC(2,6)*H*); ^{13}C NMR (125 MHz, $CDCl_3$) 35.7 (pyrrolizine*C*(1)H), 40.3 (pyrrolizine(1)*C*H^{a}H^{b}C(O)Ar), 48.0 (pyrrolizine*C*(3)H^{a}H^b), 50.0 (pyrrolizine*C*(2)H), 52.4 (CO_2CH_3), 100.3 (pyrrolizine*C*(7)H), 112.8 (pyrrolizine*C*(6)H), 114.2 (pyrrolizine*C*(5)H), 128.7 (C(O)Ar*C*(4)), 129.9 (C(O)Ar*C*(2,6)H), 132.3 (C(O)Ar*C*(3,5)H), 135.9 (pyrrolizine*C*(7a)), 137.9 (C(O)Ar*C*(1)), 172.47 (*C*O$_2$Me), 197.2 (*C*(O)Ar); HRMS (NSI$^+$), $C_{17}H_{17}{}^{77}BrNO_4$ $[M+H]^+$, requires 362.0386, found 362.0378 (−2.3 ppm).

Methyl (1*S*,2*R*)-1-(2-(3-bromophenyl)-2-oxoethyl)-2,3-dihydro-1*H*-pyrrolizine-2-carboxylate

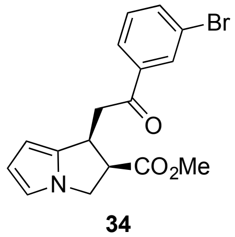

34

Following general procedure E, pyrrolyl enone-acid **13** (100 mg, 0.29 mmol), *i*-Pr_2NEt (151 µL, 0.87 mmol), pivaloyl chloride (107 µL, 0.87 mmol) in CH_2Cl_2 (2 mL), (+)-BTM **24** (4 mg, 0.016 mmol) and *i*-Pr_2NEt (76 µL, 0.44 mmol) gave the crude product (>95:5 dr). Purification by column chromatography (EtOAc:petrol 10:90) gave the title compound (>95:5 dr) as a brown oil (84 mg, 80%); $[\alpha]_D^{20}$ −53.4 (*c* 0.5 in $CHCl_3$); Chiral HPLC analysis: ChiralPak OD-H (90:10 hexane:IPA, 1.0 mlmin^{-1}, 220 nm), t_R 15.6 (minor) and t_R 17.6 (major), >99% ee; ν_{max} (ATR)/cm^{-1} 1695 (C=O), 1771 (C=O), 2980 (C-H); ^{1}H NMR (500 MHz, $CDCl_3$) 3.19 (1H, dd, *J* 6.6, 17.8, pyrrolizine(1)C*H*^{a}COAr), 3.28 (1H, dd, *J* 7.6, 17.8, pyrrolizine(1)C*H*^{b}COAr), 3.61 (3H, s, CO_2CH_3), 3.99 (1H, q, *J* 7.8, pyrrolizine(2)*H*), 4.14-4.21 (2H, m, pyrrolizine(3)*H*^{a} and pyrrolizine(1)*H*), 4.32 (1H, dd, *J* 7.4, 10.6, pyrrolizine(3)*H*^{b}), 5.80 (1H, d, *J* 3.4, pyrrolizine(7)*H*), 6.19 (1H, t, *J* 3.0, pyrrolizine(6)*H*), 6.61 (1H, dd, *J* 1.2, 2.6, pyrrolizine(5)*H*), 7.33 (1H, t, *J* 7.8, C(O)Ar(5)*H*), 7.68 (1H, ddd, 0.9, 1.8, 7.9, C(O)Ar(4)*H*), 7.83 (1H, dt, *J* 1.1, 7.8, C(O)Ar(6)*H*), 8.04 (1H, t, *J* 1.7, C(O)Ar(2)*H*); ^{13}C NMR (125 MHz, $CDCl_3$) 35.4 (pyrrolizine*C*(1)H), 40.2 (pyrrolizine(1)*C*H^{a}H^{b}COAr), 47.8 (pyrrolizine*C*(3)H^{a}H^b), 49.8 (pyrrolizine*C*(2)H), 52.2 (CO_2CH_3), 100.1 (pyrrolizine*C*(7)H), 112.6 (pyrrolizine*C*(6)H), 114.2 (pyrrolizine*C*(5)H), 123.2 (C(O)Ar*C*(3)), 126.6 (C(O)Ar*C*(6)H), 130.4 (C(O)Ar*C*(5)H), 131.2 (C(O)Ar*C*(2)), 136.2 (C(O)Ar*C*(4)H), 137.6 (pyrrolizine*C*(8)), 138.6 (C(O)Ar*C*(1)), 172.2 (*C*O_2Me), 196.7 (*C*(O)Ar); HRMS (NSI$^+$), $C_{17}H_{17}Br^{79}NO_3$ [M+H]$^+$, requires 362.0386, found 362.0386 (−0.1 ppm).

Methyl (1*S*,2*R*)-1-(2-oxo-2-(thiophen-2-yl)ethyl)-2,3-dihydro-1*H*-pyrrolizine-2-carboxylate

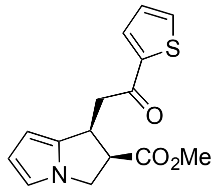

35

Following general procedure E, pyrrolyl enone-acid **14** (100 mg, 0.29 mmol), *i*-Pr_2NEt (151 µL, 0.87 mmol), pivaloyl chloride (107 µL, 0.87 mmol) in CH_2Cl_2 (2 mL), (+)-BTM **24** (4 mg, 0.016 mmol) and *i*-Pr_2NEt (76 µL, 0.44 mmol) gave, after purification by column chromatography (EtOAc:petrol ether 10:90) the title compound (>95:5 dr) as a brown oil (84 mg, 80%); $[\alpha]_D^{20}$ −75.2 (*c* 0.5 in $CHCl_3$); Chiral HPLC analysis: ChiralPak OD-H (90:10 hexane:IPA, 1.0 mlmin^{-1}, 270 nm), t_R 18.3 (minor) and t_R 30.9 (major), 99:1 er; ν_{max} (ATR)/cm^{-1} 1661 (C=O), 1724 (C=O), 2895, 2951 (C-H); ^{1}H NMR (400 MHz, $CDCl_3$) 3.20 (2H, dq, *J* 7.2, 17.2, pyrrolizine(1)C*H*^{a}*H*^{b}), 3.60 (3H, s, CO_2CH_3), 3.97 (1H, q, *J* 7.8, pyrrolizine(2)*H*), 4.14 (1H, dd, *J* 7.8, 10.7, pyrrolizine(3)*H*^{a}), 4.20 (1H, q, *J* 7.5, pyrrolizine(1)*H*), 4.33 (1H, dd, *J* 7.3, 10.7, pyrrolizine(3)*H*^{b}), 5.80 (1H, d, *J* 3.4, pyrrolizine(7)*H*), 6.19 (1H, t, *J* 3.0, pyrrolizine(6)*H*), 6.60 (1H, dd, *J* 1.2, 2.6, pyrrolizine(5)*H*), 7.11 (1H, dd, *J* 3.9, 4.9, C(O)thienyl(4)*H*), 7.62-7.65 (2H, m, C(O)thienyl(3)*H* and C(O)thienyl(5)*H*); ^{13}C NMR (100 MHz,

$CDCl_3$) 35.5 (pyrrolizine*C*(1)H), 40.7 (pyrrolizine(1)*C*H^{a}H^{b}CO), 47.7 (pyrrolizine*C*(3)H^{a}H^b), 49.8 (pyrrolizine*C*(2)H), 52.1 (CO_2CH_3), 100.0 (pyrrolizine*C*(7)H), 112.6 (pyrrolizine*C*(6)H), 114.2 (pyrrolizine*C*(5)H), 128.3 (C(O)thienyl*C*(4)H), 132.1 (C(O)thienyl*C*(3)H), 133.8 (C(O)thienyl*C*(5)H), 137.6 (pyrrolizine*C*(8)), 144.2 (C(O)thienyl*C*(2)), 172.2 (CO_2Me), 190.7 (*C*(O)thienyl); HRMS (NSI^+), $C_{15}H_{15}NO_3SNa$ $[M+Na]^+$, requires 312.0665, found 312.0660 (−1.6 ppm).

Methyl (1*S*,2*R*)-1-(2-(4-methoxyphenyl)-2-oxoethyl)-2,3-dihydro-1*H*-pyrrolizine-2-carboxylate

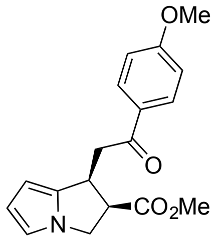

36

Following general procedure E, pyrrolyl enone-acid **15** (42 mg, 0.13 mmol), *i*-Pr_2NEt (70 µL, 0.40 mmol), pivaloyl chloride (50 µL, 0.40 mmol) in CH_2Cl_2 (2 mL), (+)-BTM **24** (1.7 mg, 0.007 mmol) and *i*-Pr_2NEt (35 µL, 0.20 mmol) gave the crude product (>95:5 dr). Purification by column chromatography (EtOAc:petrol 10:90) gave the title compound (>95:5 dr) as a yellow oil (41 mg, 98%); %); $[\alpha]_D^{20}$ −61.3 (*c* 0.5 in $CHCl_3$); Chiral HPLC analysis: ChiralPak AD-H (90:10 hexane:IPA, flow rate 1.0 mlmin^{-1}, 270 nm) t_R 20.0 (minor) and t_R 25.1 (major) >99:1 er; ν_{max} (ATR)/cm^{-1} 2953 (C-H), 1734 (C=O), 1676 (C=O); ^1H NMR (500 MHz, $CDCl_3$) 3.12-3.29 (2H, m, pyrrolizine(1)C*H^a*H^{b}C(O)Ar and pyrrolizine(1)CH^a*H^b*C(O)Ar), 3.57 (3H, s, CO_2CH_3), 3.86 (3H, s, C(O)Ar(4)OCH_3), 3.94-4.02 (1H, m, pyrrolizine(2)*H*), 4.11-4.25 (2H, m, pyrrolizine(3)*H^a*H^b and pyrrolizine(1)*H*), 4.33 (1H, dd, *J* 7.1, 10.6, pyrrolizine(3)H^a*H^b*), 5.80 (1H, dt, *J* 1.0, 3.4, pyrrolizine(7)*H*), 6.19 (1H, m, pyrrolizine(6)*H*), 6.60 (1H, dd, *J* 1.3, 2.6, pyrrolizine(5)*H*), 6.92 (2H, d, *J* 8.9, C(O)ArC(3,5)*H*), 7.90 (2H, d, *J* 9.0, C(O)ArC(2,6)*H*); ^{13}C NMR (125 MHz, $CDCl_3$) 35.7 (pyrrolizine*C*(1)H), 39.7 (pyrrolizine(1)*C*H^{a}H^b), 47.8 (pyrrolizine*C*(3)H^{a}H^b), 49.9 (pyrrolizine*C*(2)H), 52.1 (CO_2CH_3), 55.6 (C(O)Ar(4)OCH_3), 99.9 (pyrrolizine*C*(7)H), 112.5 (pyrrolizine*C*(6)H), 113.8 (C(O)Ar*C*(2,6)H), 114.0 (pyrrolizine*C*(5)H), 130.1 (C(O)Ar*C*(1)), 130.4 (C(O)Ar*C*(3,5)H, 138.0 (C(O)Ar*C*(4)), 163.6 (pyrrolizine*C*(7a)), 172.3 (CO_2Me), 196.4 (*C*(O)Ar); HRMS (NSI^+), $C_{18}H_{20}NO_4$ $[M+H]^+$, requires 314.1389, found 314.1388 (+0.3 ppm).

Methyl (1*S*,2*R*)-1-(2-(naphthalen-2-yl)-2-oxoethyl)-2,3-dihydro-1*H*-pyrrolizine-2-carboxylate

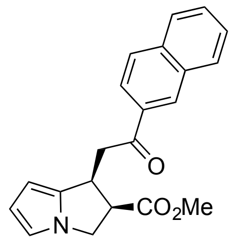

37

Following general procedure E, pyrrolyl enone-acid **16** (100 mg, 0.31 mmol), *i*-Pr_2NEt (162 µL, 0.93 mmol), pivaloyl chloride (115 µL, 0.93 mmol) in CH_2Cl_2 (3 mL), (+)-BTM **24** (4 mg, 0.016 mmol) and *i*-Pr_2NEt (82 µL, 0.47 mmol) gave the crude product (>95:5 dr). Purification by column chromatography (EtOAc:petrol 10:90) gave the title compound (>95:5 dr) as a white solid (84 mg, 81%); mp 108-110 °C; $[\alpha]_D^{20}$ –64.1 (*c* 0.5 in $CHCl_3$); Chiral HPLC analysis ChiralPak OD-H (90:10 hexane:IPA, flow rate 1.0 mlmin^{-1}, 220 nm, 30 °C), t_R 19.3 (minor) and t_R 26.5 (major), >99:1 er; ν_{max} (ATR)/cm^{-1} 1674 (C=O), 1720 (C=O), 3021 (C-H) ; ^{1}H NMR (400 MHz, $CDCl_3$) 3.35 (1H, dd, *J* 7.8, 17.6, pyrrolizine(1)CH^aH^{b}C(O)Ar), 3.46 (1H, dd, *J* 7.8, 17.6, pyrrolizine(1)CH^aH^bC(O)Ar), 3.58 (3H, s, CO_2CH_3), 4.03 (1H, q, *J* pyrrolizine(2)*H*), 4.18 (1H, dd, *J* 7.8, 10.5, pyrrolizine(3)H^aH^b), 4.29 (1H, q, *J* 7.3, pyrrolizine(1)*H*), 4.37 (1H, dd, *J* 7.2, 10.5, pyrrolizine(3)H^aH^b), 5.85 (1H, br. s, pyrrolizine(7)*H*), 6.21 (1H, br. s, pyrrolizine(6)*H*), 6.63 (1H, br. s, pyrrolizine(5)*H*), 7.54-7.62 (2H, m, Ar*H*), 7.86-8.03 (4H, m, Ar*H*), 8.42 (1H, br. s, Ar*H*); ^{13}C NMR (125 MHz, $CDCl_3$) 35.7 (pyrrolizine*C*(1)H), 40.2 (pyrrolizine(2)*C*H^{a}H^{b}C(O)Ar), 47.8 (pyrrolizine*C*(3)H^{a}H^b), 49.9 (pyrrolizine*C*(2)H), 51.8 (CO_2CH_3), 100.0 (pyrrolizine*C*(7)H), 112.6 (pyrrolizine*C*(6)H), 114.1 (pyrrolizine*C*(5)H), 123.8 (Ar*C*H), 127.0 (Ar*C*H), 127.9 (Ar*C*H), 128.6 (Ar*C*H), 128.6 (Ar*C*H), 129.7 (Ar*C*H), 129.8 (Ar*C*H), 132.6 (Ar*C*), 134.2 (Ar*C*), 135.8 (pyrrolizine*C*(7a)), 137.9 (C(O)Ar*C*(1)), 172.4 (*C*O_2Me), 197.9 (*C*(O)Ar); HRMS (NSI$^+$), $C_{21}H_{19}NO_3Na$ [M+Na]$^+$, requires 356.1257, found 356.1250 (−1.1 ppm).

Methyl (1*S*,2*R*)-1-(2-(naphthalen-1-yl)-2-oxoethyl)-2,3-dihydro-1*H*-pyrrolizine-2-carboxylate

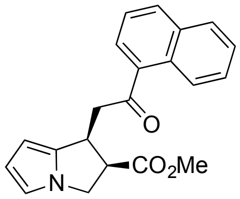

38

Following general procedure E, pyrrolyl enone-acid **17** (100 mg, 0.31 mmol), *i*-Pr_2NEt (162 µL, 0.93 mmol), pivaloyl chloride (115 µL, 0.93 mmol) in CH_2Cl_2 (3 mL), (+)-BTM **24** (4 mg, 0.016 mmol) and *i*-Pr_2NEt (82 µL, 0.47 mmol) gave the crude product (>95:5 dr). Purification by column chromatography (EtOAc:petrol 10:90) gave the title compound (>95:5 dr) as a white solid (74 mg,

72%); $[\alpha]_D^{20}$ –55.2 (*c* 0.5 in $CHCl_3$); Chiral HPLC analysis ChiralPak AD-H (90:10 hexane:IPA, flow rate 1.0 mlmin^{-1}, 211 nm, 30 °C), t_R 12.5 (minor) and t_R 14.2 (major), >99:1 er; ν_{max} (ATR)/cm^{-1} 1165, 1670 (C=O), 1732 (C=O), 2951 (C-H); ^{1}H NMR (400 MHz, $CDCl_3$) 3.36 (2H, d, *J* 7.2, pyrrolizine(1)CH^aH^{b}C(O)Ar and pyrrolizine(1)CH^aH^bC(O)Ar), 3.57 (3H, s, CO_2CH_3), 4.05 (1H, q, *J* pyrrolizine(2)*H*), 4.18 (1H, dd, *J* 7.8, 10.6, pyrrolizine(3)H^aH^b), 4.30 (1H, q, *J* 7.5, pyrrolizine(1)*H*), 4.35 (1H, dd, *J* 7.1, 10.6, pyrrolizine(3)H^aH^b), 5.86 (1H, d, *J* 3.4, pyrrolizine(7)*H*), 6.20 (1H, *J* 3.0, pyrrolizine(6)*H*), 6.62 (1H, dd, *J* 1.2, 2.5, pyrrolizine(5)*H*), 7.46-7.49 (1H, m, Ar*H*), 7.52-7.56 (1H, m, Ar*H*), 7.61 (1H, ddd, *J* 1.3, 6.9, 8.5, Ar*H*), 7.81-7.83 (1H, m, Ar*H*), 7.87-7.88 (1H, m, Ar*H*), 7.98 (1H, br. d, *J* 8.2, Ar*H*), 8.61 (1H, br. d, *J* 8.6, Ar*H*); HRMS (NSI$^+$), $C_{11}H_{12}NO_3$ [M+H]$^+$, requires 206.0823, found 206.0823 (0.0 ppm).

Methyl (1*S*,2*R*)-1-(2-(4-bromophenyl)-2-oxoethyl)-5-chloro-2,3-dihydro-1*H*-pyrrolizine-2-carboxylate

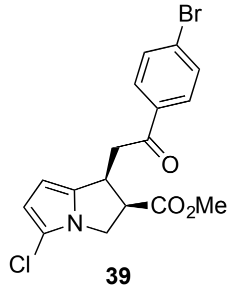

39

Following general procedure E, pyrrolyl enone-acid **19** (40 mg, 0.11 mmol), *i*-Pr_2NEt (55 μL, 0.32 mmol), pivaloyl chloride (39 μL, 0.32 mmol) in CH_2Cl_2 (1 mL), (+)-BTM **24** (1 mg, 0.005 mmol) and *i*-Pr_2NEt (18 μL, 0.11 mmol) gave, after purification by column chromatography (EtOAc:petrol 10:90) the title compound (94:6 dr) as a brown oil (29 mg, 66%); $[\alpha]_D^{20}$ –40.1 (*c* 0.5 in $CHCl_3$); Chiral HPLC analysis: ChiralPak OD-H (90:10 hexane:IPA, 1.0 mlmin^{-1}, 211 nm), t_R 18.0 (minor) and t_R 21.4 (major), >99:1 er; ν_{max} (ATR)/cm^{-1} 1584 (C=O), 1688 (C=O), 2926, 2951 (C-H); ^{1}H NMR (500 MHz, $CDCl_3$) 3.18 (1H, dd, *J* 7.1, 17.7, pyrrolizine(1)CH^aH^{b}COAr), 3.24 (1H, dd, *J* 7.2, 17.7, pyrrolizine(1)CH^aH^bCOAr), 3.62 (3H, s, CO_2CH_3), 3.97 (1H, q, *J* 7.8, pyrrolizine(2)*H*), 4.12 (1H, dd, *J* 7.9, 10.9, pyrrolizine(3)H^aH^b), 4.17-4.27 (2H, m, pyrrolizine(1)*H* and pyrrolizine(3)H^aH^b) 5.74 (1H, dd, *J* 0.8, 3.6, pyrrolizine(7)*H*), 5.97 (1H, d, *J* 3.6, pyrrolizine(6)*H*), 7.60 (2H, d, *J* 8.6, C(O)Ar(3,5)*H*), 7.77 (1H, d, *J* 8.6, C(O)Ar(2,6)*H*); ^{13}C NMR (125 MHz, $CDCl_3$) 36.4 (pyrrolizine*C*(1)H), 39.9 (pyrrolizine(1)*C*H^{a}H^{b}COAr), 46.4 (pyrrolizine*C*(3)H^{a}H^b), 49.3 (pyrrolizine*C*(2)H), 52.3 (CO_2CH_3), 100.9 (pyrrolizine*C*(7)H), 109.6 (pyrrolizine*C*(6)H), 127.5 (C(O)Ar*C*(4)Br), 128.7 (pyrrolizine*C*(8)), 129.6 (C(O)Ar*C*(3,5)H), 132.1 (C(O)Ar*C*(2,6)H), 135.5 (C(O)Ar*C*(1)), 136.4 (pyrrolizine*C*(5)Cl), 171.8 (*C*O_2Me), 196.8 (*C*(O)Ar); HRMS (NSI$^+$), $C_{17}H_{16}Br^{79}ClNO_3$ [M+Na]$^+$, requires 397.9974, found 397.9974 (0.0 ppm).

Methyl (1*S*,2*R*)-1-(2-oxo-2-phenylethyl)-2,3,5,6,7,8-hexahydro-1*H*-pyrrolo[1,2-*a*]indole-2-carboxylate

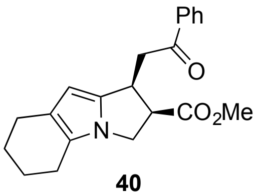

40

Following general procedure E, pyrrolyl enone-acid **18** (100 mg, 0.31 mmol), *i*-Pr_2NEt (162 μL, 0.93 mmol), pivaloyl chloride (115 μL, 0.93 mmol) in CH_2Cl_2 (3 mL), (+)-BTM **24** (4 mg, 0.016 mmol) and *i*-Pr_2NEt (54 μL, 0.31 mmol) gave, after purification by column chromatography (EtOAc:petrol 10:90) the title compound (>95:5 dr) as a brown oil (55 mg, 53%); $[\alpha]_D^{20}$ – 33.1 (*c* 0.5 in $CHCl_3$); Chiral HPLC analysis: ChiralPak IC (90:10 hexane:IPA, 1.0 $mlmin^{-1}$, 211 nm), t_R 14.7 (major) and t_R 17.4 (minor), >99% ee; ν_{max} (ATR)/cm^{-1} 1665 (C=O), 1730 (C=O), 2841, 2927, 2953 (C-H); ^1H NMR (500 MHz, $CDCl_3$) 1.68-1.74 (2H, m, pyrroloindolyl(7)H_2), 1.76-1.84 (2H, m, pyrroloindolyl(6)H_2), 2.47-2.55 (4H, m, pyrroloindolyl(8)H_2 and pyrroloindolyl(5)H_2), 3.18 (1H, dd, *J* 6.5, 17.8, pyrroloindolyl(1)C$H^a$$H^b$COAr), 3.57 (3H, s, CO_2CH_3), 3.92-4.01 (2H, m, pyrroloindolyl(2)*H* and pyrroloindolyl(3)H^a), 4.14 (1H, dd, *J* 7.0, 9.9, pyrroloindolyl(3)H^b), 4.19 (1H, q, *J* 7.4, pyrroloindolyl(1)*H*), 5.59 (1H, s, pyrroloindolyl(9)*H*), 7.43-7.46 (2H, m, C(O)Ar(3,5)*H*), 7.53-7.57 (1H, m, C(O)Ar(4)*H*), 7.91-7.93 (C(O)Ar(2,6)*H*); ^{13}C NMR (125 MHz, $CDCl_3$) 21.9 (pyrroloindolyl*C*(9)H_2), 23.4 (pyrroloindolyl*C*(8)H_2), 23.7 (pyrroloindolyl*C*(5)H_2), 23.8 (pyrroloindolyl*C*(6)H_2), 35.6 (pyrroloindolyl*C*(1)H), 40.5 (pyrroloindolyl(1)*C*$H^a$$H^b$COAr), 45.4 (pyrroloindolylC(3)$H^a$$H^b$), 49.6 (pyrroloindolyl*C*(2)H), 52.1 (CO_2CH_3), 98.4 (pyrroloindolyl*C*(9)H), 121.3 (pyrroloindolyl*C*(8a)), 122.9 (pyrroloindolyl*C*(4a)), 128.1 (C(O)Ar*C*(3,5)H), 128.7 (C(O)Ar*C*(2,6)H), 133.3 (C(O)Ar*C*(4)H), 135.5 (pyrroloindolyl*C*(9a)), 137.0 (C(O)Ar*C*(1)), 172.5 (*C*O_2Me), 198.1 (*C*(O)Ar); In our hands this compound proved to be unstable to mass spectrometry analysis.

1.3.7 Derivatisations

***Tert*-butyl 5-methoxy-2-(4-(2-((1*S*,2*R*)-2-(methoxycarbonyl)-2,3-dihydro-1*H*-pyrrolizin-1-yl)acetyl)phenyl)-1*H*-indole-1-carboxylate**

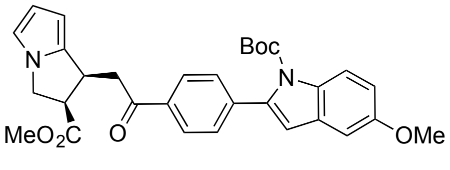

40

A flame-dried schlenk flask, under inert atmosphere, was charged with $Pd(PPh_3)_4$ (16 mg, 0.014 mmol), pyrrolizine **33** (50 mg, 0.14), Na_2CO_3 (45 mg, 0.42 mmol) and *N*-Boc-5-methoxy-2-indolylboronic acid (49 mg, 0.17 mmol). Degassed DME (2.2 mL) was added and reaction stirred at

80 °C for 16 h. Reaction cooled to rt and dilute with EtOAc, washed with brine (×3), dried over $MgSO_4$ and concentrated under reduced pressure to give crude reaction mixture that, following column chromatography (EtOAc:Petrol 20:80), gave the title compound (>95:5 dr) as a brown oil (45 mg, 60%); $[\alpha]_D^{20}$ –22.0 (c 0.5 in $CHCl_3$); Chiral HPLC analysis ChiralPak AD-H (90:10 hexane:IPA, 1.0 mlmin^{-1}, 254 nm), t_R 42.6 (major) and t_R 52.0 (minor), >99:1 er; ν_{max} (ATR)/cm^{-1} 1607, 1681 (C=O), 1726 (C=O), 2929, 2951 (C-H); ^{1}H NMR (300 MHz, $CDCl_3$) 1.35 (9H, s, $NCO_2C(C\mathit{H_3})_3$), 3.20-3.38 (2H, m, pyrrolizine(1)CH^aH^{b}C(O)indole), 3.62 (3H, s, $CO_2C\mathit{H_3}$), 3.87 (3H, s, ArOC$\mathit{H_3}$), 3.97-4.05 (1H, m, pyrrolizine(2)*H*), 4.14-4.27 (2H, m, pyrrolizine(1)*H* and pyrrolizine(3)H^aH^b), 4.35 (1H, dd, *J* 7.2, 10.6, pyrrolizine(3)H^aH^b), 5.81 (1H, d, *J* 3.3, pyrrolizine(7)*H*), 6.19-6.21 (1H, m, pyrrolizine(6)*H*), 6.55 (1H, s, indolyl(3)*H*), 6.61 (1H, dd, *J* 1.2, 2.6, pyrrolizine(5)*H*), 6.95-7.03 (2H, m, indolyl(6)*H* and indolyl(7)*H*), 7.50 (2H, d, *J* 8.4, C(O)Ar(3,5)*H*), 7.95 (2H, d, *J* 8.5, C(O)Ar(2,6)*H*), 8.08 (1H, d, *J* 9.0, indolyl(4)*H*); ^{13}C NMR (100 MHz, $CDCl_3$) 27.9 ($C(C\mathit{H_3})_3$), 35.6 (pyrrolizine*C*(1)H), 40.2 (pyrrolizine(1)*C*H^{a}H^{b}C(O)Ar), 47.8 (pyrrolizine*C*(3)H^{a}H^b), 49.9 (pyrrolizine*C*(2)H), 52.1 ($CO_2\mathit{C}H_3$), 55.9 (ArO*C*H_3), 84.0 ($NCO_2\mathit{C}(CH_3)_3$), 100.1 (pyrrolizine*C*(7)H), 103.2 (indolyl*C*(3)H), 111.2 (pyrrolizine*C*(7)H), 112.2 (pyrrolizine*C*(6)H), 113.9 (indolyl*C*(4)H), 114.1 (indolyl*C*(6)H), 116.3 (pyrrolizine*C*(5)H), 127.7 (C(O)Ar*C*(3,5)*H*), 128.9 (C(O)Ar(2,6)*H*), 130.0 (indolyl*C*(2)), 132.6 (indolyl*C*(7a)), 135.8 (indolyl*C*(3a)), 137.9 (pyrrolizine*C*(8)), 139.8 (C(O)Ar*C*(1)), 140.0 (C(O)Ar*C*(4)), 150.1 (N*C*O_2*t*-Bu), 156.3 (indolyl*C*(5)), 172.3 (*C*O_2Me), 197.5 (*C*(O)Ar); HRMS (NSI$^+$), $C_{31}H_{32}N_2O_6Na$ [M+Na]$^+$, requires 551.2158, found 551.2138 (−3.6 ppm).

Methyl (1*S*,2*R*)-5-chloro-1-(2-oxo-2-phenylethyl)-2,3-dihydro-1*H*-pyrrolizine-2-carboxylate

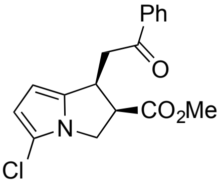

41

To a solution of pyrrolizine **27** in CH_2Cl_2 (5 mL) at 0 °C, under inert atmosphere was added *N*-chlorosucinimide (47 mg, 0.35 mmol) and reaction stirred for 2 h. Reaction was dilute with CH_2Cl_2 (5 mL), washed with Na_2CO_3 (×3), dried over $MgSO_4$ and concentrated under reduced pressure to give crude reaction mixture that, following column chromatography (EtOAc:Petrol 20:80), gave the title compound (>95:5 dr) as a brown oil (86 mg, 79%); $[\alpha]_D^{20}$ –31.2 (c 0.5 in $CHCl_3$); Chiral HPLC analysis ChiralPak OD-H (90:10 hexane:IPA, 1.0 mlmin^{-1}, 211 nm), t_R 24.2 (minor) and t_R 39.7 (major), >99:1 er. ν_{max} (ATR)/cm^{-1} 1681 (C=O), 1730 (C=O), 3001 (C-H); ^{1}H NMR (500 MHz, $CDCl_3$) 3.18-3.30 (2H, m, pyrrolizine(1)CH^aH^{b}C(O)Ar and pyrrolizine(1)CH^aH^bC(O)Ar), 3.61 (3H, s, $CO_2C\mathit{H_3}$), 3.98 (1H, q, *J* 7.8, pyrrolizine(2)*H*), 4.12 (1H, dd, *J* 7.8, 10.9, pyrrolizine(3)H^aH^b), 4.19-4.27 (2H, m, pyrrolizine(3)H^aH^b and pyrrolizine(1)*H*), 5.75 (1H, d, *J* 3.5, pyrrolizine(7)*H*), 5.97 (1H,

d, *J* 3.5, pyrrolizine(6)*H*), 7.44-7.47 (2H, m, C(O)Ar(3,5)*H*), 7.55-7.58 (1H, m, C(O)Ar(4)*H*), 7.90-7.92 (2H, m, C(O)Ar(2,6)*H*); ^{13}C NMR (125 MHz, $CDCl_3$) 36.5 (pyrrolizine*C*(1)H), 39.9 (pyrrolizine*C*(2)H), 46.5 (pyrrolizine(1)*C*H^aH^bC(O)Ar), 49.3 (pyrrolizine*C*(3)H), 52.2 (CO_2*C*H_3), 100.9 (pyrrolizine*C*(7)H), 109.5 (pyrrolizine*C*(6)H), 110.1 (), 128.1 (C(O)Ar*C*(3,5)H), 128.8 (C(O)Ar*C*(2,6)H), 133.4 (pyrrolizine*C*(8)). 136.6 (C(O)Ar*C*(1)), 136.8 (pyrrolizine*C*(5)Cl), 171.9 (*C*O_2Me), 197.7 (*C*(O)Ar); HRMS (NSI^+), $C_{17}H_{17}ClNO_3$ $[M+H]^+$, requires 318.0891, found 318.0893 (+0.6 ppm).

1.4 Computational Details

Calculations were run with Gaussian 09 Revision D.01.[8] Geometry optimisations were performed using the M06-2X functional[9] using 6-31G** basis sets[10] on all atoms (called BS1). All stationary points were fully characterized via analytical frequency calculations as either minima (all positive eigenvalues) or transition states (one negative eigenvalue) and IRC calculations and subsequent geometry optimizations were used to confirm the minima linked by each transition state. Frequency calculations also provided a free energy in the gas-phase, computed at 298.15 K and 1 atm. SCF energies were recomputed with the larger 6-311++G** basis set[11] (BS2) and incorporate a correction for CH_2Cl_2 solvent (CH_2Cl_2, PCM approach[12]). An equivalent set of results were also generated with the B3LYP functional.[13] Reaction profiles computed with the M06-2X functional, including the reaction of **(*E*)-45** to give $\mathbf{21_{trans}}$ are given in Figure S1 while those computed with the B3LYP functional are given in Figure S2. With this latter functional the reactions of **(*Z*)-45** yielded no intermediate corresponding to **47** and the lactonisation step leads directly to products.

1.5 Computed Reaction Profiles and Labelling Schemes

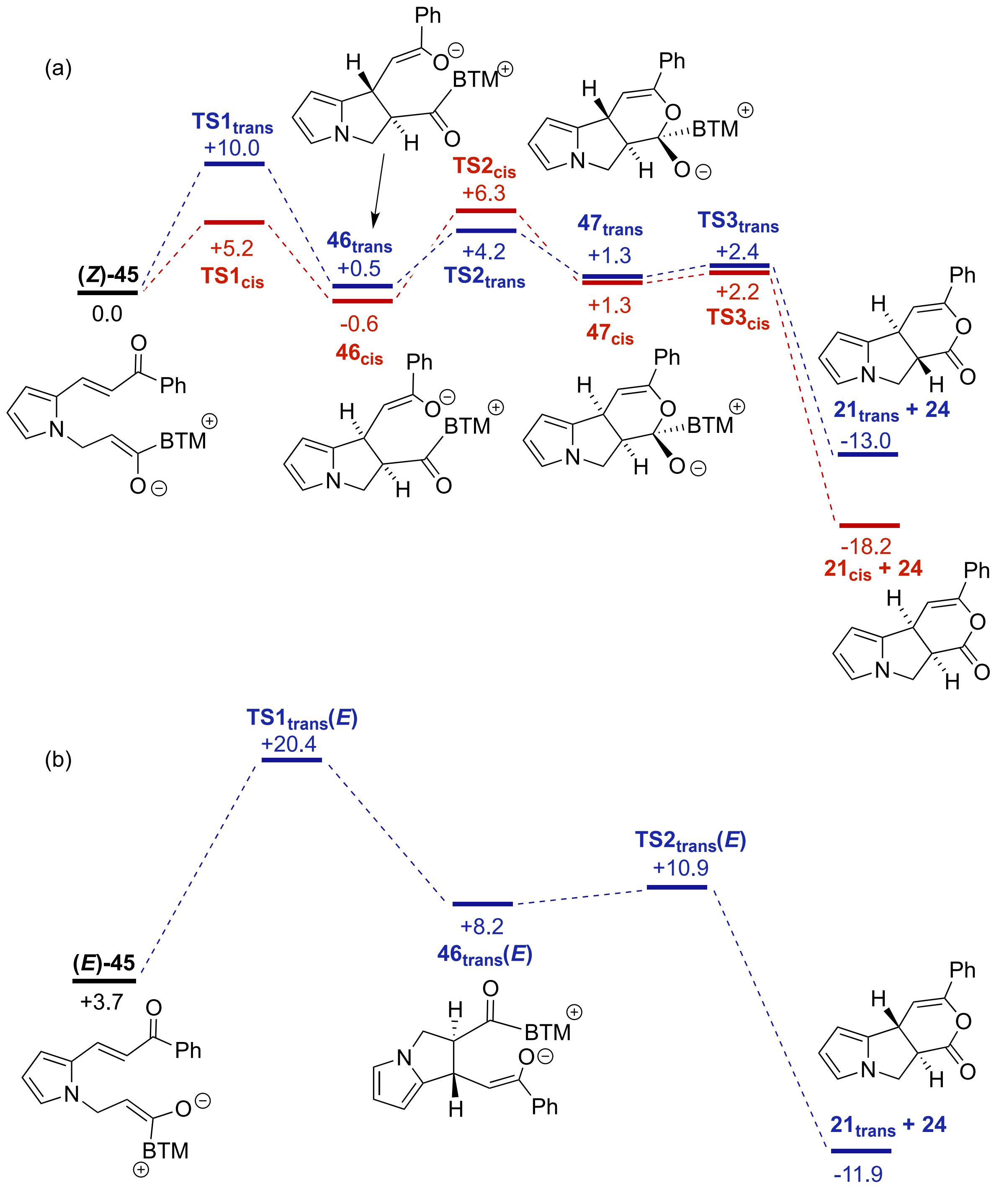

Figure S1. Reaction profiles (free energies, kcal/mol) computed with the M06-2X functional for the formation of cis- and trans-isomers of **21** from (a) **(*Z*)-45** (as shown in the main text) and (b) **(*E*)-45**. Free energies include corrections from dichloromethane solvent.

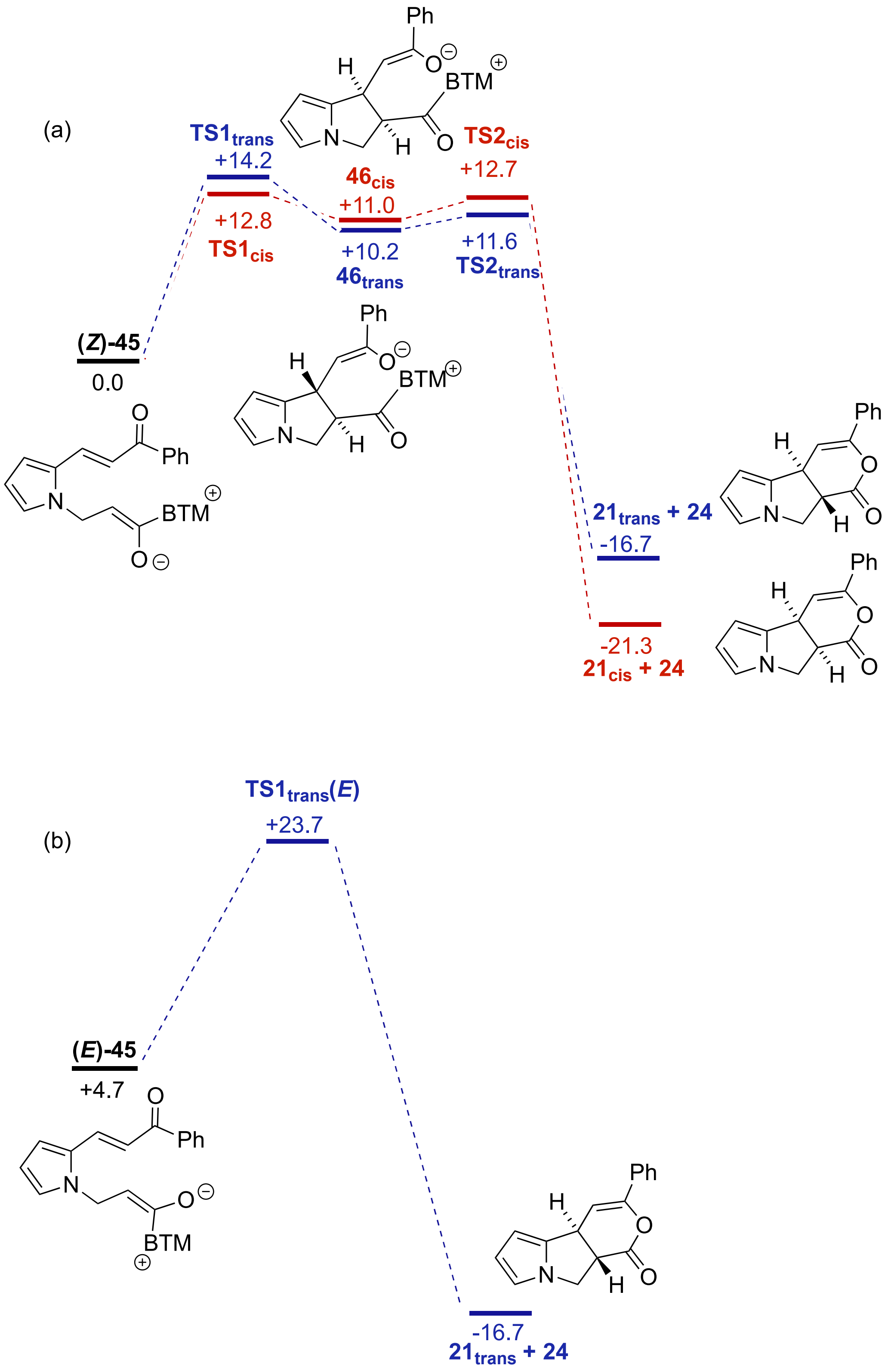

Figure S2. Reaction profiles (free energies, kcal/mol) computed with the B3LYP functional for the formation of cis- and trans-isomers of **21** from (a) **(*Z*)-45** and (b) **(*E*)-45**. Free energies include corrections from dichloromethane solvent.

1.6 Computed Cartesian Coordinates (Å) and Energies (au) for all stationary points.

1.6.1 M06-2X

(a) Formation of 21_{cis} from (Z)-45

```
62
(Z)-45
H   3.33461  4.23186 -0.35319
C   5.17620  3.09701 -0.90928
C   3.80625  3.31606 -0.68156
H   5.98590  3.79508 -0.75865
H  -1.85300  5.65736  2.71902
H  -4.11972  4.69893  3.00990
C  -3.39179  4.19127  2.38488
C  -2.11733  4.72860  2.22394
H  -0.19757  4.52049  1.29608
C  -1.17986  4.07947  1.42534
C  -1.51091  2.88504  0.78016
C  -2.79952  2.36052  0.93539
C  -3.73299  3.00572  1.73554
H  -4.72904  2.58917  1.84929
H  -3.04812  1.44648  0.40363
H   6.17476  1.22621 -1.63641
O  -0.98089  1.14463 -0.71252
H   1.24019  3.21147  0.59892
C   0.85936  2.49645 -0.12196
C  -0.55365  2.11758 -0.09101
C   1.70907  1.87861 -0.97844
H   1.28803  1.16140 -1.68033
C   3.12612  2.14996 -1.02540
C   5.29661  1.79778 -1.37181
N   4.06562  1.23353 -1.45217
C   3.81794 -0.18037 -1.83093
C   2.83052 -0.84818 -0.94019
H   3.03738 -0.94657  0.11717
H   3.43607 -0.21098 -2.85614
O   1.21225 -1.18286 -2.66550
C   1.62147 -1.18400 -1.48255
S  -1.38281 -1.65418 -2.51599
C  -2.99908 -1.55641 -1.76948
C  -2.92213 -1.42143 -0.37679
C  -4.05874 -1.26228  0.40735
N  -1.60471 -1.48236  0.05019
C  -0.70036 -1.56036 -0.93643
N   0.54385 -1.49395 -0.51209
C   0.55506 -1.15017  0.92585
H  -1.19261 -1.85661  2.08699
C  -0.96342 -1.12370  1.30941
H  -1.29730 -0.12761  1.61715
H   4.80942 -0.64760 -1.82207
H   0.98918 -0.14778  1.01060
C   1.34333 -2.13371  1.75870
C   2.11534 -1.67565  2.82445
C   2.79643 -2.57909  3.63646
C   2.71053 -3.94447  3.38246
C   1.94459 -4.40524  2.31314
C   1.26223 -3.50344  1.50434
H   2.19239 -0.60740  3.01085
H   3.40086 -2.21526  4.46098
H   3.24629 -4.64886  4.01040
H   1.88517 -5.46862  2.10513
H   0.67915 -3.85646  0.65746
H  -3.97928 -1.15347  1.48429
C  -5.29148 -1.24118 -0.23871
H  -6.19609 -1.11447  0.34614
C  -5.37993 -1.38501 -1.62508
H  -6.35176 -1.36838 -2.10583
C  -4.23470 -1.54213 -2.40305
H  -4.30520 -1.63980 -3.48145

SCF  =             -1520.57793395
H(0 K)=            -1520.081400
H(298 K)=          -1520.051438
G(298 K)=          -1520.144988
SCF (BS2) =        -1520.94865112
SCF(DCM) =         -1520.59758708
Lowest Frequency = 15.8198cm-1

62
TS1cis
H  -5.68136 -2.50848 -0.42980
C  -4.62616 -2.31515 -0.59872
C  -4.22568 -1.43810 -1.60380
H  -4.96892 -0.94267 -2.22154
C  -2.31130 -2.69702 -0.02296
H  -2.53428 -0.50973 -2.58549
C  -2.87275 -1.19070 -1.81080
H  -1.58040 -3.18540  0.61216
C  -1.89727 -1.81132 -1.02456
C  -3.66437 -2.94584  0.18846
H  -3.96870 -3.63564  0.97008
C  -0.45261 -1.43661 -1.27872
O  -0.24318 -0.50988 -2.09986
H   0.34025 -2.88799  0.14873
C  -0.61259  0.79819  0.78368
C  -2.72024  1.43880  0.14876
C  -3.00428  0.62392  1.25262
S  -1.52311 -0.02816  1.98961
C   0.57037 -2.11159 -0.56840
C   1.60225 -0.02445  1.18638
O   1.15508 -0.57943  2.18523
C   1.92159 -1.78077 -0.77443
C   2.77934 -0.32300  0.46031
H   2.10553 -1.19921 -1.67821
N   0.68950  0.88399  0.53361
C  -3.72647  2.05585 -0.58417
N  -1.35125  1.50135 -0.07204
C  -0.52311  2.15691 -1.08412
H  -0.62531  3.24303 -1.00261
H  -0.80438  1.79355 -2.07304
C   0.91363  1.67237 -0.70688
C   3.83628 -1.12933  1.16475
H   3.54517 -1.30482  2.20564
H   4.80785 -0.62506  1.14916
```

C 4.80285 -3.44971 0.67088
H 5.63659 -3.36213 1.35168
C 4.36401 -4.50511 -0.09962
N 3.94636 -2.40525 0.46665
C 2.95966 -2.77129 -0.40092
C 3.18767 -4.08003 -0.77418
H 2.58664 -4.64398 -1.47205
H 4.83847 -5.47317 -0.17295
H 3.12304 0.41129 -0.25971
C 1.90110 2.79558 -0.50872
H 1.24363 0.97602 -1.48099
C 1.84159 3.62668 0.61202
C 2.74222 4.67696 0.75120
C 3.70606 4.90709 -0.22958
C 3.76885 4.08149 -1.34762
C 2.86933 3.02731 -1.48456
H 1.09827 3.44062 1.38389
H 2.69707 5.31420 1.62833
H 4.41056 5.72454 -0.11700
H 4.52176 4.25098 -2.11023
H 2.92083 2.37377 -2.35167
H -3.48803 2.67727 -1.44057
C -5.03966 1.82958 -0.18705
H -5.84846 2.29495 -0.73980
C -5.33365 1.00834 0.90505
H -6.36718 0.83977 1.18650
C -4.31955 0.39383 1.63435
H -4.54920 -0.25445 2.47320

SCF = -1520.57045438
H(0 K)= -1520.074169
H(298 K)= -1520.045209
G(298 K)= -1520.135459
SCF (BS2) = -1520.94239287
SCF(DCM) = -1520.59017197
Lowest Frequency = -325.4725cm-1

62
46cis
H -5.63593 -2.74271 -0.19301
C -4.58950 -2.50721 -0.36456
C -4.21707 -1.67603 -1.41803
H -4.97514 -1.25856 -2.07522
C -2.26315 -2.73250 0.25443
H -2.55643 -0.71803 -2.43203
C -2.87453 -1.37381 -1.62792
H -1.51263 -3.12920 0.93093
C -1.87554 -1.89876 -0.80155
C -3.60479 -3.03346 0.47163
H -3.88349 -3.68261 1.29709
C -0.44985 -1.45208 -1.05343
O -0.31332 -0.46185 -1.85928
H 0.43307 -2.94430 0.23553
C -0.68204 0.95616 0.79222
C -2.72099 1.53880 -0.04487
C -3.09275 0.76120 1.05985
S -1.67748 0.18292 1.95663
C 0.58338 -2.06907 -0.38386
C 1.43158 -0.02193 1.26000
O 1.04893 -0.40063 2.34268
C 1.98992 -1.60441 -0.61399
C 2.58427 -0.57270 0.48355
H 2.00752 -1.09467 -1.58460
N 0.64269 0.94929 0.58825
C -3.66700 2.08270 -0.90554
N -1.33883 1.63912 -0.13097
C -0.43857 2.17597 -1.15425
H -0.49462 3.26830 -1.15924
H -0.70024 1.73162 -2.11440
C 0.95852 1.65997 -0.68503
C 3.55289 -1.37942 1.36606
H 3.05834 -1.70951 2.28507
H 4.44611 -0.80668 1.63015
C 4.78141 -3.52892 0.58606
H 5.53529 -3.56905 1.35753
C 4.50382 -4.37912 -0.46354
N 3.88073 -2.50244 0.51309
C 3.03165 -2.68442 -0.53983
C 3.39020 -3.84978 -1.17795
H 2.91357 -4.26959 -2.05080
H 5.05009 -5.28104 -0.70001
H 3.11666 0.23342 -0.03077
C 1.98348 2.74688 -0.47750
H 1.28005 0.90980 -1.40895
C 1.85465 3.67584 0.55810
C 2.79558 4.68748 0.71194
C 3.87159 4.78021 -0.17015
C 4.00526 3.85723 -1.20228
C 3.06398 2.84114 -1.35312
H 1.02013 3.59910 1.25193
H 2.69261 5.40338 1.52078
H 4.60686 5.56859 -0.04751
H 4.84430 3.92167 -1.88707
H 3.16804 2.11230 -2.15296
H -3.36417 2.67022 -1.76521
C -5.00414 1.82273 -0.63150
H -5.76682 2.22499 -1.28913
C -5.38202 1.04441 0.46775
H -6.43277 0.84733 0.64861
C -4.43126 0.50142 1.32496
H -4.72177 -0.12441 2.16136

SCF = -1520.58086231
H(0 K)= -1520.082168
H(298 K)= -1520.053440
G(298 K)= -1520.141603
SCF (BS2) = -1520.95375291
SCF(DCM) = -1520.60266181
Lowest Frequency = 25.0511cm-1

62
TS2cis
H 4.31902 5.25267 -0.39454
C 3.43422 4.62797 -0.46979
C 3.55491 3.25952 -0.69522
H 4.53875 2.80988 -0.80171
C 1.02913 4.39461 -0.44424
H 2.48856 1.39632 -0.96949
C 2.41677 2.46457 -0.79098
H 0.04892 4.84852 -0.33954
C 1.13808 3.01677 -0.66318
C 2.16427 5.19099 -0.34759

```
H   2.05887  6.25763 -0.17299
C  -0.04033  2.10110 -0.74884
O   0.21400  0.82293 -0.84937
H  -1.64887  3.53084 -0.48411
C   1.20586 -1.13487  0.65524
C   3.22575 -1.85383 -0.15609
C   3.63213 -0.97074  0.85289
S   2.24620 -0.21658  1.66835
C  -1.33327  2.50870 -0.64333
C  -0.77738  0.09173  0.91845
O  -0.28038  0.68191  1.85803
C  -2.35948  1.41718 -0.70125
C  -2.18623  0.31967  0.43771
H  -2.28168  0.88741 -1.66077
N  -0.12149 -1.16705  0.53767
C   4.14446 -2.53455 -0.94753
N   1.83954 -1.91328 -0.21947
C   0.90125 -2.51516 -1.16181
H   0.89397 -3.60297 -1.05099
H   1.17358 -2.24425 -2.18416
C  -0.45793 -1.88031 -0.72458
C  -3.08232  0.82826  1.58506
H  -2.52194  1.51820  2.22318
H  -3.48858  0.01696  2.19532
C  -5.36714  2.00629  1.14777
H  -5.83396  1.82838  2.10460
C  -5.80884  2.68437  0.03087
N  -4.12705  1.51540  0.85049
C  -3.76061  1.87642 -0.41445
C  -4.78902  2.60816 -0.96281
H  -4.81478  3.03517 -1.95431
H  -6.76869  3.17091 -0.06828
H  -2.60607 -0.61813  0.06083
C  -1.54317 -2.91233 -0.54070
H  -0.74368 -1.10286 -1.43903
C  -1.49603 -3.80108  0.53559
C  -2.47260 -4.77964  0.67884
C  -3.50366 -4.87819 -0.25435
C  -3.55775 -3.99182 -1.32533
C  -2.58009 -3.00898 -1.46718
H  -0.70004 -3.71123  1.27106
H  -2.43433 -5.46231  1.52137
H  -4.26781 -5.63992 -0.14023
H  -4.36466 -4.05759 -2.04756
H  -2.62832 -2.30517 -2.29440
H   3.81377 -3.21408 -1.72571
C   5.49380 -2.30662 -0.70450
H   6.23535 -2.82030 -1.30613
C   5.90939 -1.42580  0.29937
H   6.96853 -1.26556  0.46683
C   4.98442 -0.74739  1.08660
H   5.30742 -0.05764  1.85907

SCF  =          -1520.56751370
H(0 K)=         -1520.069736
H(298 K)=       -1520.041394
G(298 K)=       -1520.130949
SCF (BS2) =     -1520.93799971
SCF(DCM) =      -1520.59134609
Lowest Frequency = -133.2045cm-1
```

```
62
47cis
H  -0.52480  7.09221 -1.24750
C  -0.73179  6.03194 -1.14070
C   0.14634  5.21490 -0.43127
H   1.03549  5.64107  0.02330
C  -2.13907  4.12382 -1.58629
H   0.54521  3.20100  0.26512
C  -0.11344  3.85551 -0.29842
H  -3.01718  3.68819 -2.05402
C  -1.26439  3.30285 -0.86955
C  -1.87482  5.48284 -1.71770
H  -2.55617  6.11317 -2.28073
C  -1.53351  1.86379 -0.67660
O  -0.40932  1.09166 -0.56649
H  -3.64805  1.87247 -0.59194
C   1.99782 -0.39229  0.39753
C   4.24556 -0.60538 -0.08814
C   4.28181  0.23777  1.03170
S   2.64471  0.63276  1.63431
C  -2.73592  1.28777 -0.56740
C  -0.37986  0.24396  0.65643
O   0.04976  0.82236  1.68923
C  -2.79490 -0.19300 -0.27810
C  -1.69466 -0.56544  0.76362
H  -2.65971 -0.77516 -1.19952
N   0.77373 -0.75006  0.10757
C   5.40653 -1.02760 -0.72392
N   2.93199 -0.90650 -0.42419
C   2.30492 -1.75796 -1.42835
H   2.52895 -2.81130 -1.22900
H   2.64424 -1.49790 -2.43330
C   0.79027 -1.44223 -1.20349
C  -2.37981 -0.34785  2.12680
H  -2.26701  0.68907  2.45462
H  -1.98879 -1.00781  2.90376
C  -4.90224 -0.92583  2.50521
H  -4.89370 -1.03082  3.57945
C  -5.92978 -1.03177  1.58967
N  -3.76355 -0.66132  1.80191
C  -4.03960 -0.58274  0.46724
C  -5.38610 -0.80902  0.29090
H  -5.92083 -0.82240 -0.64739
H  -6.95936 -1.25984  1.82590
H  -1.47878 -1.63235  0.66229
C  -0.06989 -2.68248 -1.23070
H   0.43130 -0.71906 -1.94440
C   0.08536 -3.66270 -0.24618
C  -0.70455 -4.80512 -0.26387
C  -1.65442 -4.98115 -1.27020
C  -1.80880 -4.01217 -2.25536
C  -1.01743 -2.86424 -2.23490
H   0.80859 -3.51028  0.55192
H  -0.58711 -5.55598  0.51050
H  -2.27660 -5.86983 -1.27947
H  -2.55153 -4.14172 -3.03540
H  -1.14587 -2.09915 -2.99624
H   5.36258 -1.68033 -1.58934
C   6.62210 -0.58271 -0.21122
H   7.54476 -0.89747 -0.68644
C   6.67009  0.25994  0.90107
```

H   7.62896 0.59355 1.28181
C   5.49985 0.67912 1.53102
H   5.53736 1.33421 2.39531

SCF =   -1520.58316728
H(0 K)=   -1520.084567
H(298 K)=   -1520.055876
G(298 K)=   -1520.146536
SCF (BS2) =   -1520.94938367
SCF(DCM) =   -1520.60363005
Lowest Frequency = 14.3213cm-1

62
TS3cis
H -0.73537 7.09518 -1.34020
C -0.90534 6.03176 -1.20385
C -0.02028 5.27367 -0.44030
H   0.83704 5.74820 0.02703
C -2.22336 4.05638 -1.62928
H   0.43670 3.30204 0.33164
C -0.23246 3.91018 -0.26950
H -3.06706 3.57240 -2.11225
C -1.34249 3.29408 -0.85665
C -2.00705 5.41948 -1.79790
H -2.69287 6.00406 -2.40313
C -1.56329 1.85224 -0.62638
O -0.40791 1.14209 -0.41912
H -3.67476 1.76402 -0.68671
C   2.02590 -0.39204 0.42236
C   4.27407 -0.52456 -0.12404
C   4.32076 0.26259 1.03633
S   2.69460 0.59029 1.69496
C -2.74236 1.22227 -0.57924
C -0.42539 0.29893 0.78002
O   0.02285 0.81457 1.81528
C -2.76358 -0.25268 -0.25476
C -1.70158 -0.55943 0.84429
H -2.56789 -0.85215 -1.15380
N   0.81048 -0.76713 0.15473
C   5.43141 -0.88838 -0.80155
N   2.96223 -0.83448 -0.44937
C   2.33490 -1.68107 -1.45627
H   2.59350 -2.73244 -1.28705
H   2.63720 -1.39013 -2.46455
C   0.81927 -1.41198 -1.17636
C -2.45407 -0.33803 2.17168
H -2.39493 0.70847 2.48479
H -2.07487 -0.96793 2.97857
C -4.96990 -0.99027 2.45091
H -5.00656 -1.07018 3.52669
C -5.95083 -1.15143 1.49349
N -3.80950 -0.70483 1.79231
C -4.02761 -0.66620 0.44489
C -5.35653 -0.94127 0.21485
H -5.84720 -0.99606 -0.74576
H -6.98222 -1.40731 1.68956
H -1.43050 -1.61605 0.77916
C -0.01232 -2.67120 -1.22532
H   0.42450 -0.67588 -1.88725
C   0.13747 -3.64626 -0.23494
C -0.61636 -4.81257 -0.27919
C -1.52510 -5.01770 -1.31695
C -1.67550 -4.05293 -2.30716
C -0.92044 -2.88182 -2.26032
H   0.82969 -3.47148 0.58552
H -0.50238 -5.55979 0.49930
H -2.11954 -5.92479 -1.34671
H -2.38766 -4.20419 -3.11153
H -1.04595 -2.12118 -3.02684
H   5.38286 -1.49771 -1.69778
C   6.64903 -0.44438 -0.29154
H   7.56700 -0.71577 -0.80129
C   6.70457 0.34194 0.86015
H   7.66411 0.67626 1.23846
C   5.53872 0.70449 1.53315
H   5.58006 1.31662 2.42816

SCF =   -1520.58280508
H(0 K)=   -1520.085090
H(298 K)=   -1520.056779
G(298 K)=   -1520.147213
SCF (BS2) =   -1520.94886926
SCF(DCM) =   -1520.60151674
Lowest Frequency = -143.6195cm-1

30
24
C -0.37710 -0.92916 -1.22428
C   0.42125 1.11372 -0.57912
H -0.78329 -1.77933 -0.67111
H   0.00492 -1.27039 -2.19472
N -0.68409 1.48845 -1.08559
N   0.66646 -0.24585 -0.48043
S   1.79485 2.03818 0.04329
C -2.58860 0.04644 -0.41591
C -3.67420 -0.73814 -0.81046
C -2.57127 0.58426 0.87025
C -4.72459 -0.98792 0.06700
H -3.69850 -1.15162 -1.81640
C -3.62164 0.33476 1.74969
H -1.74040 1.21418 1.17148
C -4.69899 -0.45219 1.35228
H -5.56576 -1.59431 -0.25395
H -3.59950 0.76077 2.74787
H -5.51815 -0.64229 2.03836
C -1.42130 0.23987 -1.36656
H -1.80673 0.27321 -2.38999
C   2.49263 -1.87590 -0.04280
C   3.81148 -2.01141 0.39000
C   4.56750 -0.89996 0.75485
C   4.01971 0.38310 0.68683
C   2.71328 0.52460 0.25135
C   1.94623 -0.59939 -0.10644
H   1.89875 -2.74007 -0.32123
H   4.25110 -3.00166 0.44469
H   5.59005 -1.02637 1.09230
H   4.60705 1.25288 0.96213

SCF =   -698.207579658
H(0 K)=   -697.969189
H(298 K)=   -697.955637
G(298 K)=   -698.011487

```
SCF (BS2) =     -698.364477812
SCF(DCM) =  -698.21608794
Lowest Frequency = 27.1554cm-1

32
21cis
H   5.82010 -1.62107 -0.56137
C   4.82388 -1.23321 -0.37496
C   4.61851  0.13713 -0.24517
H   5.45506  0.82333 -0.32807
C   2.46499 -1.61106 -0.04260
H   3.17920  1.70397  0.09099
C   3.34213  0.63684 -0.00818
H   1.62197 -2.29338 -0.00276
C   2.25522 -0.23566  0.10771
C   3.74187 -2.10545 -0.27565
H   3.89086 -3.17380 -0.39429
C   0.90501  0.29316  0.38949
O   0.80268  1.63013  0.02786
H   0.07190 -1.39883  1.28310
C  -0.09546 -0.37673  0.96562
C  -0.38094  2.29821  0.08410
O  -0.38719  3.48366 -0.08943
C  -1.46291  0.21640  1.17205
C  -1.62853  1.47109  0.27970
H  -1.62779  0.45280  2.22913
C  -2.14386  0.91731 -1.07622
H  -1.31576  0.63176 -1.73703
H  -2.78126  1.63583 -1.59501
C  -3.78812 -1.10370 -1.19668
H  -4.19470 -0.93481 -2.18200
C  -4.03543 -2.09371 -0.26922
N  -2.88106 -0.24985 -0.63603
C  -2.53740 -0.67977  0.61484
C  -3.23862 -1.83111  0.88374
H  -3.20167 -2.41288  1.79268
H  -4.73056 -2.91061 -0.39670
H  -2.38602  2.13782  0.69454

SCF  =          -822.386011564
H(0 K)=         -822.127591
H(298 K)=       -822.113487
G(298 K)=       -822.170084
SCF (BS2) =     -822.597470569
SCF(DCM) =      -822.395396679
Lowest Frequency = 36.2883cm-1
```

(b) Formation of 21_{trans} from (Z)-45

```
62
TS1trans
C   1.40854  1.89966 -0.13653
C  -0.00731  1.98635 -0.12701
C   1.31016 -1.48706 -1.20996
H   2.00960  2.43886  0.58554
C   5.58142  0.72554 -1.85918
H   6.37809  0.02108 -2.04762
C   4.26095  2.51512 -1.47011
H   3.89065  3.52364 -1.35851
C   5.58146  2.10373 -1.79657
H   6.43848  2.74185 -1.95628
N   4.31262  0.30046 -1.59193
C   3.49981  1.36907 -1.35682
C   3.74725 -1.03986 -1.47274
H   3.51584 -1.44957 -2.46134
H   4.48527 -1.68839 -0.98793
O   1.08889 -1.84143 -2.36580
O  -0.75010  1.24957 -0.81571
C  -0.68585  2.97435  0.79773
C  -2.08243  2.92921  0.87654
C  -0.00208  3.91622  1.57283
C  -2.77831  3.78387  1.72413
H  -2.59932  2.21442  0.24224
C  -0.69681  4.77327  2.42107
H   1.07713  3.99825  1.50514
C  -2.08563  4.70678  2.50447
H  -3.86230  3.73701  1.77179
H  -0.15171  5.50067  3.01458
H  -2.62497  5.37708  3.16656
C  -1.40980 -0.73135  1.30274
C  -1.05999 -1.43599 -0.86511
H  -1.81595 -1.36605  2.09487
H  -1.60530  0.32340  1.51252
N   0.15305 -1.49945 -0.33469
N  -1.99663 -1.08774  0.01370
S  -1.63553 -1.63521 -2.47469
C   2.06771  1.11218 -1.09734
C   2.48122 -0.90216 -0.67018
H   2.59283 -0.88333  0.40862
H   1.45966  0.84085 -1.96275
C   0.69933 -1.99777  2.04078
C   1.55969 -1.54623  3.03971
C   0.36453 -3.35177  1.97904
C   2.07892 -2.43806  3.97557
H   1.82885 -0.49334  3.07728
C   0.88577 -4.24289  2.91047
H  -0.29439 -3.70830  1.19063
C   1.74218 -3.78639  3.91138
H   2.75172 -2.07925  4.74744
H   0.62796 -5.29548  2.85423
H   2.15000 -4.48396  4.63555
C   0.11014 -1.00666  1.06590
H   0.66341 -0.06473  1.10096
C  -4.39269 -0.41674  0.11708
C  -5.54850 -0.26685 -0.64078
C  -5.55971 -0.55995 -2.00770
C  -4.40970 -1.00210 -2.65544
C  -3.24818 -1.14674 -1.90731
C  -3.24886 -0.86098 -0.53631
H  -4.37291 -0.18776  1.17742
H  -6.45552  0.08485 -0.16160
H  -6.47520 -0.43511 -2.57505
H  -4.41612 -1.21613 -3.71887

SCF  =          -1520.56334604
H(0 K)=         -1520.067232
H(298 K)=       -1520.038106
G(298 K)=       -1520.129413
SCF (BS2) =     -1520.93417690
SCF(DCM) =      -1520.58264494
Lowest Frequency = -353.9720cm-1
```

```
62
46trans
C   1.56637  1.86015 -0.12101
C   0.19120  1.91209 -0.15865
C   1.13549 -1.24710 -1.30712
H   2.15963  2.51504  0.50552
C   5.76995  0.45226 -1.74054
H   6.49855 -0.32133 -1.92991
C   4.61369  2.36757 -1.35031
H   4.36093  3.40752 -1.20710
C   5.90099  1.82052 -1.63199
H   6.82672  2.36959 -1.72842
N   4.45129  0.16252 -1.52313
C   3.74114  1.30613 -1.29728
C   3.63723 -1.03619 -1.55685
H   3.39695 -1.32877 -2.58455
H   4.13349 -1.86982 -1.05255
O   0.95896 -1.62248 -2.44424
O  -0.53412  1.18290 -0.92548
C  -0.55768  2.80389  0.80746
C  -1.91229  3.04725  0.54908
C   0.00273  3.33920  1.97295
C  -2.67847  3.82159  1.41501
H  -2.33436  2.60547 -0.34844
C  -0.76208  4.11292  2.84103
H   1.04136  3.13208  2.21203
C  -2.10568  4.36001  2.56529
H  -3.72500  4.00991  1.19137
H  -0.31062  4.51834  3.74178
H  -2.70112  4.96321  3.24379
C  -1.44963 -0.57535  1.27605
C  -1.18297 -1.34369 -0.87946
H  -1.85843 -1.15047  2.11072
H  -1.60552  0.49772  1.41552
N   0.06252 -1.41204 -0.38635
N  -2.08142 -0.98139  0.02159
S  -1.80656 -1.55174 -2.46457
C   2.28055  1.00777 -1.11977
C   2.37241 -0.56401 -0.81508
H   2.50717 -0.69610  0.26117
H   1.75950  1.08337 -2.08759
C   0.62996 -1.91989  1.97700
C   1.62758 -1.52218  2.86520
C   0.16823 -3.23793  2.00286
C   2.15808 -2.43202  3.77799
H   1.98828 -0.49639  2.83475
C   0.69783 -4.14625  2.91188
H  -0.60133 -3.55418  1.30143
C   1.69320 -3.74275  3.80178
H   2.93588 -2.11557  4.46506
H   0.33838 -5.17000  2.92644
H   2.10674 -4.45331  4.50994
C   0.05104 -0.90756  1.02046
H   0.62754  0.02134  1.05023
C  -4.45607 -0.24836  0.18553
C  -5.62803 -0.08235 -0.54036
C  -5.68768 -0.39610 -1.90336
C  -4.57031 -0.87164 -2.58025
C  -3.39076 -1.03209 -1.86171
C  -3.34406 -0.73210 -0.49549
H  -4.39616  0.00341  1.23883
H  -6.51121  0.30107 -0.04166
H  -6.61721 -0.25709 -2.44385
H  -4.61229 -1.09699 -3.64034

SCF  =           -1520.57674162
H(0 K)=          -1520.078237
H(298 K)=        -1520.049155
G(298 K)=        -1520.140220
SCF (BS2) =      -1520.94925455
SCF(DCM) =         -1520.59859437
Lowest Frequency = 19.4021cm-1

62
TS2trans
C   1.95842  2.01022 -0.70946
C   0.59788  1.99724 -0.65784
C   0.73481 -0.90157 -1.29774
H   2.58700  2.71027 -0.17441
C   5.71589 -0.81226 -1.39655
H   6.16278 -1.79253 -1.32904
C   5.22659  1.40017 -1.54209
H   5.32419  2.47200 -1.63174
C   6.28146  0.43988 -1.51265
H   7.34116  0.64638 -1.55825
N   4.36158 -0.63352 -1.34228
C   4.05075  0.69489 -1.43830
C   3.19985 -1.50486 -1.28696
H   2.96417 -1.92183 -2.27251
H   3.34542 -2.31917 -0.57122
O   0.47226 -1.33599 -2.40148
O  -0.08774  1.05501 -1.24659
C  -0.19449  2.95627  0.18135
C  -1.55709  2.69564  0.37176
C   0.36050  4.07547  0.81265
C  -2.33672  3.50884  1.19064
H  -1.98588  1.85050 -0.15990
C  -0.41730  4.89106  1.62698
H   1.40705  4.31683  0.65612
C  -1.76839  4.60867  1.82736
H  -3.39398  3.29163  1.31992
H   0.03182  5.75592  2.10597
H  -2.37260  5.24832  2.46301
C  -1.69756 -0.60066  1.56989
C  -1.52163 -1.18244 -0.65271
H  -1.91835 -1.38524  2.30004
H  -1.99057  0.37787  1.95920
N  -0.25589 -1.20280 -0.24545
N  -2.38701 -0.87423  0.31213
S  -2.24524 -1.43685 -2.19236
C   2.56125  0.86702 -1.45663
C   2.11113 -0.50953 -0.84323
H   2.16195 -0.40094  0.24132
H   2.19227  0.86295 -2.49537
C   0.68085 -1.39925  2.06616
C   1.58165 -0.71426  2.88040
C   0.59482 -2.79032  2.14710
C   2.38409 -1.41289  3.77889
H   1.66520  0.36649  2.79092
C   1.39947 -3.48781  3.04153
H  -0.09172 -3.32604  1.49573
C   2.29253 -2.79905  3.86075
```

```
H   3.08760 -0.87401  4.40470
H   1.33462 -4.56965  3.09654
H   2.92213 -3.34542  4.55537
C  -0.20385 -0.62087  1.12485
H   0.15391  0.40736  1.02442
C  -4.82638 -0.52448  0.69565
C  -6.06895 -0.53051  0.07292
C  -6.19464 -0.83156 -1.28694
C  -5.07827 -1.13070 -2.06208
C  -3.83105 -1.12881 -1.44853
C  -3.71659 -0.82924 -0.08460
H  -4.71813 -0.28906  1.74901
H  -6.95408 -0.29437  0.65296
H  -7.17605 -0.82844 -1.74782
H  -5.17713 -1.35441 -3.11891

SCF  =           -1520.57135201
H(0 K)=          -1520.073917
H(298 K)=        -1520.045621
G(298 K)=        -1520.134353
SCF (BS2) =      -1520.94210845
SCF(DCM) =       -1520.59488561
Lowest Frequency = -68.3535cm-1

62
47trans
C  -2.94071  0.35191 -1.27278
C  -2.19497 -0.76335 -1.21612
C  -0.05254  0.50499 -1.08025
H  -4.01977  0.29193 -1.33976
C  -2.20353  4.84800  0.27776
H  -1.54984  5.54794  0.77594
C  -3.91405  3.71057 -0.69056
H  -4.88995  3.45263 -1.07453
C  -3.54299  4.92587 -0.04119
H  -4.19184  5.76147  0.17894
N  -1.76117  3.62645 -0.14333
C  -2.78371  2.92975 -0.73515
C  -0.48239  2.91876 -0.15610
H   0.14960  3.24452 -0.99035
H   0.05051  3.05444  0.78970
O   0.56991  0.88764 -2.09994
O  -0.82735 -0.77190 -1.20658
C  -2.74712 -2.13699 -1.15618
C  -1.98731 -3.21917 -1.61410
C  -4.00726 -2.38410 -0.59778
C  -2.48863 -4.51541 -1.53687
H  -1.00743 -3.02614 -2.03724
C  -4.50647 -3.67939 -0.52499
H  -4.58322 -1.55554 -0.19683
C  -3.74939 -4.75094 -0.99520
H  -1.89240 -5.34532 -1.90366
H  -5.48432 -3.85439 -0.08694
H  -4.13774 -5.76239 -0.93193
C   2.25160 -1.28012  1.60642
C   2.28167 -0.00290 -0.30298
H   2.38962 -0.69521  2.52338
H   2.50145 -2.32431  1.80140
N   1.01536 -0.04828 -0.00110
N   3.06602 -0.72527  0.52668
S   3.15566  0.81697 -1.55419
C  -2.20982  1.65593 -1.28264
C  -0.97435  1.48904 -0.37620
H  -1.32837  1.08398  0.57717
H  -1.83642  1.85340 -2.30138
C  -0.26271 -0.79526  2.00444
C  -1.44746 -1.52790  1.97127
C  -0.13829  0.27469  2.89399
C  -2.50129 -1.19685  2.81894
H  -1.55773 -2.33834  1.25530
C  -1.18891  0.60378  3.74271
H   0.76868  0.87517  2.90042
C  -2.37313 -0.13201  3.70506
H  -3.42413 -1.76603  2.77238
H  -1.09156  1.44183  4.42501
H  -3.19657  0.13387  4.35970
C   0.82426 -1.11628  1.01263
H   0.54324 -2.02013  0.46050
C   5.47154 -1.11995  1.05872
C   6.77352 -0.83592  0.65509
C   7.02291 -0.02671 -0.45445
C   5.97320  0.52331 -1.18822
C   4.67138  0.25499 -0.78869
C   4.43147 -0.56639  0.32283
H   5.26958 -1.75202  1.91707
H   7.60454 -1.25365  1.21290
H   8.04541  0.17978 -0.75039
H   6.16899  1.15326 -2.04985

SCF  =           -1520.58188595
H(0 K)=          -1520.083323
H(298 K)=        -1520.054704
G(298 K)=        -1520.144561
SCF (BS2) =      -1520.94910779
SCF(DCM) =       -1520.60334642
Lowest Frequency = 13.1829cm-1

62
TS3trans
C  -2.97992  0.33970 -1.24492
C  -2.17993 -0.73778 -1.27927
C  -0.09737  0.61011 -1.17959
H  -4.05707  0.23333 -1.22473
C  -2.35552  4.80932  0.41820
H  -1.70937  5.51726  0.91486
C  -4.05962  3.63873 -0.52156
H  -5.03985  3.35491 -0.87488
C  -3.70927  4.84422  0.15655
H  -4.38006  5.64504  0.43262
N  -1.88382  3.62225 -0.06456
C  -2.90258  2.90619 -0.63982
C  -0.57907  2.96934 -0.15622
H   0.00508  3.35107 -1.00134
H  -0.01183  3.09145  0.77108
O   0.56227  1.04261 -2.13948
O  -0.81514 -0.66623 -1.38464
C  -2.64844 -2.14111 -1.21891
C  -1.84563 -3.17215 -1.71862
C  -3.86650 -2.46774 -0.61003
C  -2.26133 -4.49772 -1.62582
H  -0.89921 -2.91807 -2.18352
C  -4.28040 -3.79148 -0.52239
```

```
H  -4.47402 -1.67992 -0.17530
C  -3.47900 -4.81308 -1.02964
H  -1.63130 -5.28694 -2.02436
H  -5.22554 -4.02822 -0.04358
H  -3.80026 -5.84701 -0.95481
C   2.22427 -1.36790  1.49260
C   2.26980  0.06695 -0.29913
H   2.34673 -0.86432  2.45911
H   2.47120 -2.42552  1.60072
N   1.00214 -0.00938 -0.03331
N   3.05236 -0.72306  0.47663
S   3.17132  1.00335 -1.45377
C  -2.30581  1.67452 -1.25449
C  -1.02363  1.53055 -0.40901
H  -1.32038  1.07436  0.54026
H  -1.99433  1.91472 -2.28512
C  -0.27936 -0.89201  1.91289
C  -1.41936 -1.69243  1.91895
C  -0.18102  0.17453  2.81046
C  -2.45653 -1.42964  2.81113
H  -1.50809 -2.50731  1.20513
C  -1.21447  0.43639  3.70177
H   0.69408  0.82072  2.78878
C  -2.35532 -0.36670  3.70236
H  -3.34572 -2.05173  2.79587
H  -1.13844  1.27267  4.38893
H  -3.16563 -0.15515  4.39234
C   0.80318 -1.14752  0.89658
H   0.51454 -2.00860  0.28198
C   5.44994 -1.12950  1.03194
C   6.75709 -0.79340  0.68782
C   7.02110  0.11370 -0.33901
C   5.98013  0.71293 -1.04717
C   4.67413  0.39258 -0.70510
C   4.41802 -0.52764  0.32268
H   5.23886 -1.83868  1.82536
H   7.58048 -1.24859  1.22734
H   8.04702  0.35902 -0.59001
H   6.18463  1.42043 -1.84408

SCF  =           -1520.58188254
H(0 K)=          -1520.084034
H(298 K)=        -1520.055973
G(298 K)=        -1520.144178
SCF (BS2) =      -1520.94908942
SCF(DCM) =       -1520.60204523
Lowest Frequency = -91.6091cm-1

32
21trans
C   0.08494 -0.81464 -0.33381
C   1.09250  0.06517 -0.26337
C  -0.35892  2.00743 -0.20634
H   0.26181 -1.87545 -0.21299
C  -4.64993 -0.50955  0.52529
H  -5.47660  0.08564  0.88203
C  -3.21971 -2.13200 -0.17064
H  -2.81418 -3.09173 -0.45428
C  -4.54581 -1.86217  0.27950
H  -5.33408 -2.58707  0.42039
N  -3.43091  0.04318  0.25409
C  -2.55741 -0.92892 -0.17123
C  -2.86809  1.39153  0.25983
H  -3.13984  1.94731 -0.64491
H  -3.18834  1.95772  1.13597
O  -0.50910  3.17632 -0.41253
O   0.87264  1.43523 -0.40823
C   2.52304 -0.23892 -0.06545
C   3.39780  0.74898  0.39814
C   3.02067 -1.51962 -0.33157
C   4.73950  0.45101  0.61344
H   3.01986  1.74784  0.58390
C   4.36001 -1.81357 -0.11218
H   2.36215 -2.28245 -0.73412
C   5.22398 -0.82937  0.36423
H   5.40850  1.22452  0.97629
H   4.73375 -2.80972 -0.32557
H   6.27160 -1.05802  0.53069
C  -1.28149 -0.25153 -0.56963
C  -1.38359  1.03095  0.27784
H  -1.10343  0.74481  1.30062
H  -1.35812  0.03316 -1.63489

SCF  =           -822.378729697
H(0 K)=          -822.120657
H(298 K)=        -822.106600
G(298 K)=        -822.162684
SCF (BS2) =      -822.589593582
SCF(DCM) =       -822.388190986
Lowest Frequency = 35.9039cm-1
```

(c) 21_{trans} formation from (*E*)-45

```
62
(E)-45
C   1.24291  2.47282 -0.53495
C   1.90380  1.24404 -0.98315
C  -2.15343  0.68838  1.30496
H   1.81439  3.23388 -0.01547
C  -2.80689  4.84985 -0.12026
H  -3.87525  4.97292 -0.01064
C  -0.57762  5.06571 -0.18114
H   0.42510  5.46946 -0.18463
C  -1.77626  5.76760  0.02150
H  -1.89113  6.81970  0.23452
N  -2.28503  3.63887 -0.42414
C  -0.90660  3.73797 -0.44956
C  -3.05306  2.37112 -0.40978
H  -4.07379  2.65738 -0.68558
H  -2.66696  1.76239 -1.22945
O  -1.80600  0.27985  2.42968
O   1.34967  0.43594 -1.72928
C   3.29321  0.94768 -0.50520
C   4.06960  0.06032 -1.25438
C   3.79882  1.46894  0.68925
C   5.34868 -0.27921 -0.83235
H   3.64305 -0.35846 -2.16008
C   5.07101  1.10765  1.12426
H   3.18841  2.13144  1.29539
C   5.85015  0.24162  0.35961
H   5.95125 -0.96141 -1.42409
H   5.45587  1.50513  2.05800
```

```
H   6.84673 -0.02944  0.69426
C  -0.90492 -1.55193 -1.59709
C  -0.37679 -0.76395  0.47690
H  -1.16351 -2.53218 -2.00089
H  -0.42155 -0.91908 -2.34787
N  -1.49676 -0.12885  0.19236
N  -0.01090 -1.66351 -0.44690
S   0.73041 -0.60974  1.78940
C  -0.08193  2.61241 -0.76530
C  -2.99768  1.68592  0.91074
H  -3.48242  2.19872  1.73459
H  -0.56053  1.76224 -1.24661
C  -3.17367 -1.85417 -0.46282
C  -3.98116 -2.45983 -1.42908
C  -3.31268 -2.21378  0.87739
C  -4.91178 -3.42577 -1.06629
H  -3.88205 -2.16967 -2.47348
C  -4.25058 -3.18091  1.23617
H  -2.71938 -1.72379  1.64606
C  -5.04598 -3.79045  0.27197
H  -5.53587 -3.88784 -1.82453
H  -4.36016 -3.45102  2.28143
H  -5.77431 -4.54148  0.56073
C  -2.13098 -0.85518 -0.93333
H  -2.58319 -0.15345 -1.63625
C   1.97289 -3.02256 -1.11995
C   3.25148 -3.41060 -0.73104
C   3.81494 -2.95289  0.46266
C   3.11479 -2.08602  1.29672
C   1.84135 -1.69062  0.91175
C   1.27646 -2.16131 -0.28147
H   1.53146 -3.37203 -2.04699
H   3.81976 -4.07815 -1.36988
H   4.81838 -3.25954  0.73693
H   3.56008 -1.70890  2.21141

SCF  =          -1520.57325400
H(0 K)=         -1520.076605
H(298 K)=       -1520.047010
G(298 K)=       -1520.137365
SCF (BS2) =     -1520.94469465
SCF(DCM) =      -1520.59386756
Lowest Frequency = 24.2393cm-1

62
TS1trans (E)
C   0.12782  2.70550 -0.31183
C   1.15672  1.77236 -0.62630
C  -1.02459  0.46146  1.51133
H   0.34886  3.66185  0.14396
C  -4.62962  3.29287 -0.47953
H  -5.62662  3.02104 -0.16548
C  -2.74607  4.27350 -1.25768
H  -2.05812  4.94902 -1.74416
C  -4.16083  4.39945 -1.15422
H  -4.76608  5.21650 -1.51907
N  -3.55368  2.50396 -0.18367
C  -2.40264  3.08928 -0.64384
C  -3.42875  1.28216  0.61542
H  -4.20625  1.29751  1.38816
H  -3.60496  0.39029  0.00319
O  -0.10666  0.69283  2.30039
O   0.95120  0.67749 -1.20924
C   2.57877  2.06084 -0.22609
C   3.59366  1.31196 -0.83021
C   2.91512  2.96477  0.78488
C   4.92056  1.47947 -0.45097
H   3.30972  0.59238 -1.59226
C   4.24287  3.13029  1.16952
H   2.13252  3.51248  1.29893
C   5.24937  2.39156  0.55096
H   5.69947  0.89668 -0.93475
H   4.49130  3.82991  1.96160
H   6.28434  2.52387  0.85159
C  -0.46112 -1.90629 -1.34446
C   0.45378 -1.19714  0.63553
H  -0.77697 -2.89162 -1.69180
H  -0.15451 -1.25967 -2.17082
N  -0.79184 -0.72849  0.69926
N   0.65728 -2.00073 -0.40993
S   1.86432 -0.84846  1.55228
C  -1.18419  2.27190 -0.49622
C  -2.04361  1.38307  1.19606
H  -1.96326  2.21559  1.89055
H  -1.21074  1.36610 -1.10157
C  -2.67538 -2.16381 -0.06891
C  -3.74460 -2.32646 -0.95251
C  -2.64788 -2.89408  1.11789
C  -4.77273 -3.21510 -0.65821
H  -3.77416 -1.74573 -1.87202
C  -3.67987 -3.78212  1.41333
H  -1.82876 -2.75599  1.81751
C  -4.74085 -3.94575  0.52740
H  -5.60158 -3.32984 -1.34891
H  -3.65562 -4.34388  2.34137
H  -5.54496 -4.63511  0.76287
C  -1.55397 -1.22726 -0.46318
H  -1.96158 -0.37561 -1.00824
C   2.52788 -3.02061 -1.70371
C   3.90588 -3.20254 -1.73020
C   4.72198 -2.69441 -0.71434
C   4.17984 -1.98484  0.35262
C   2.80307 -1.79805  0.38405
C   1.99408 -2.30947 -0.63534
H   1.88885 -3.40641 -2.49060
H   4.35426 -3.74421 -2.55579
H   5.79457 -2.84661 -0.76225
H   4.81429 -1.56406  1.12543

SCF  =          -1520.54661810
H(0 K)=         -1520.050310
H(298 K)=       -1520.021473
G(298 K)=       -1520.111245
SCF (BS2) =     -1520.91858389
SCF(DCM) =      -1520.56635786
Lowest Frequency = -356.3296cm-1

62
46trans(E)
C   0.18534  2.55335 -0.02914
C   1.15529  1.67951 -0.46540
C  -1.11950  0.35232  1.56872
```

```
H   0.41504  3.49342  0.45658
C  -4.54497  3.48247 -0.47597
H  -5.56438  3.31041 -0.16510
C  -2.57774  4.24080 -1.32560
H  -1.84336  4.82594 -1.85811
C  -3.98550  4.47070 -1.25610
H  -4.52677  5.28454 -1.71713
N  -3.51954  2.67178 -0.06767
C  -2.32571  3.11655 -0.57947
C  -3.41029  1.46104  0.73114
H  -4.05023  1.50608  1.61592
H  -3.68682  0.56941  0.15168
O  -0.53548  0.34335  2.62619
O   0.91892  0.56958 -1.08009
C   2.61250  1.96863 -0.19421
C   3.56613  1.20291 -0.87272
C   3.06093  2.90405  0.74570
C   4.92704  1.37584 -0.63585
H   3.20162  0.46505 -1.58094
C   4.42022  3.07889  0.98569
H   2.34050  3.48405  1.31333
C   5.36142  2.31585  0.29554
H   5.65006  0.77221 -1.17861
H   4.74815  3.80772  1.72123
H   6.42175  2.45345  0.48533
C  -0.44456 -1.83703 -1.38391
C   0.40265 -1.22462  0.65220
H  -0.76446 -2.79840 -1.78906
H  -0.07481 -1.15003 -2.14810
N  -0.84396 -0.73177  0.68261
N   0.62687 -2.00725 -0.39856
S   1.76137 -0.94906  1.66172
C  -1.23557  2.14790 -0.21493
C  -1.90929  1.52862  1.07069
H  -1.79024  2.26317  1.87214
H  -1.19647  1.37935 -0.99704
C  -2.70279 -2.11581 -0.19011
C  -3.84168 -2.11976 -0.99710
C  -2.61128 -3.01550  0.87289
C  -4.87595 -3.01803 -0.75025
H  -3.92017 -1.41235 -1.81955
C  -3.64709 -3.91083  1.12112
H  -1.73536 -3.00852  1.51577
C  -4.77898 -3.91584  0.30918
H  -5.75932 -3.01046 -1.38008
H  -3.57102 -4.60426  1.95218
H  -5.58603 -4.61367  0.50631
C  -1.56820 -1.17437 -0.53055
H  -1.95360 -0.30310 -1.05968
C   2.55769 -2.90180 -1.69666
C   3.94099 -3.03708 -1.70340
C   4.72115 -2.55487 -0.64737
C   4.13782 -1.91750  0.44272
C   2.75638 -1.77563  0.44937
C   1.98033 -2.26142 -0.60719
H   1.94702 -3.26360 -2.51664
H   4.42244 -3.52022 -2.54635
H   5.79922 -2.66522 -0.68333
H   4.74354 -1.50874  1.24403

SCF  =          -1520.56352307
H(0 K)=          -1520.065397
H(298 K)=        -1520.036365
G(298 K)=        -1520.127014
SCF (BS2) =      -1520.93664723
SCF(DCM) =       -1520.58564372
Lowest Frequency = 16.2203cm-1

62
TS2trans(E)
C  -1.45129  2.62062 -0.22329
C  -0.10765  2.44183 -0.41786
C  -0.57183 -0.04491  1.45960
H  -1.91523  3.58419 -0.05740
C  -5.55928  0.44656  0.93596
H  -6.10349 -0.26865  1.53381
C  -4.84387  2.14500 -0.39225
H  -4.82831  3.00533 -1.04445
C  -5.99132  1.50218  0.16198
H  -7.02232  1.79369  0.02158
N  -4.19286  0.43912  0.87000
C  -3.74730  1.45724  0.06915
C  -3.13424 -0.38895  1.42071
H  -3.31588 -0.62420  2.47281
H  -3.03090 -1.32764  0.86191
O   0.04865  0.16048  2.48154
O   0.42954  1.26224 -0.46332
C   0.82004  3.61644 -0.53003
C   2.18409  3.40674 -0.30986
C   0.38728  4.90521 -0.85861
C   3.09058  4.45879 -0.38942
H   2.50266  2.39547 -0.07861
C   1.29147  5.95885 -0.94116
H  -0.66329  5.07705 -1.07191
C   2.64753  5.74156 -0.70305
H   4.14684  4.27956 -0.20831
H   0.93916  6.95264 -1.20178
H   3.35293  6.56430 -0.77016
C   0.94521 -1.56270 -1.54958
C   1.31805 -1.21794  0.68855
H   0.94690 -2.46917 -2.15709
H   1.21238 -0.67566 -2.13086
N   0.00277 -1.02799  0.57912
N   1.87980 -1.67488 -0.42891
S   2.42918 -0.92301  1.96689
C  -2.25489  1.37450 -0.07758
C  -1.92955  0.56056  1.23093
H  -1.98169  1.30068  2.03411
H  -2.06699  0.72948 -0.94637
C  -1.34146 -2.52970 -0.87114
C  -2.55164 -2.42717 -1.55663
C  -1.00420 -3.73198 -0.24457
C  -3.41830 -3.51685 -1.61537
H  -2.82606 -1.48575 -2.02673
C  -1.86778 -4.81924 -0.30379
H  -0.06679 -3.81163  0.30247
C  -3.07700 -4.71199 -0.99023
H  -4.36286 -3.42563 -2.14120
H  -1.60246 -5.74880  0.18916
H  -3.75363 -5.55925 -1.03103
C  -0.40915 -1.34572 -0.81947
H  -0.85284 -0.44481 -1.22772
```

```
C   4.11560 -2.23144 -1.38458
C   5.47130 -2.30695 -1.09049
C   5.95648 -1.97853  0.18001
C   5.09702 -1.55722  1.18903
C   3.73898 -1.47636  0.90194
C   3.26067 -1.81519 -0.36938
H   3.73191 -2.48258 -2.36743
H   6.16385 -2.62587 -1.86149
H   7.01944 -2.04801  0.38174
H   5.47504 -1.29133  2.17037

SCF  =           -1520.55751594
H(0 K)=          -1520.059843
H(298 K)=        -1520.031394
G(298 K)=        -1520.120697
SCF (BS2) =      -1520.92592421
SCF(DCM) =       -1520.58229559
Lowest Frequency = -20.9358cm-1
```

1.6.2 B3LYP

(a) Formation of 21cis from (Z)-45

```
62
(Z)-45
H   5.70560 -0.98093 -0.63739
C   5.32337 -3.02326 -1.43373
C   5.01111 -1.73387 -0.98401
H   6.30283 -3.47355 -1.51263
H   5.30154  5.29129  0.27225
H   3.83866  6.68609  1.71790
C   3.53308  5.68046  1.44273
C   4.35725  4.89515  0.63479
H   4.61023  3.01132 -0.36058
C   3.96922  3.60143  0.28574
C   2.74758  3.07664  0.73581
C   1.92103  3.88305  1.53377
C   2.31109  5.17054  1.89103
H   1.66457  5.77989  2.51656
H   0.97195  3.47304  1.86160
H   3.92092 -4.63020 -2.13038
O   1.06199  1.42395  0.52999
H   4.29579  0.94570 -0.00727
C   3.23931  0.72252 -0.11145
C   2.26597  1.69736  0.37530
C   2.81550 -0.46303 -0.63271
H   1.74489 -0.59397 -0.72537
C   3.61644 -1.57493 -1.03621
C   4.11568 -3.63716 -1.75038
N   3.08723 -2.78036 -1.51453
C   1.67652 -3.14183 -1.85826
C   0.63999 -2.62898 -0.91813
H   0.74886 -2.80700  0.14381
H   1.45087 -2.76366 -2.86098
O  -0.76908 -1.64772 -2.59940
C  -0.39977 -1.88881 -1.42040
S  -2.71552  0.08183 -2.41508
C  -3.72494  1.37404 -1.66723
C  -3.49668  1.48149 -0.28153
C  -4.14446  2.44517  0.48938
N  -2.58444  0.51877  0.14309
C  -2.10385 -0.27426 -0.83464
N  -1.22841 -1.16641 -0.41449
C  -0.97788 -0.97389  1.04317
H  -2.55176  0.07714  2.21586
C  -1.85898  0.28136  1.39610
H  -1.22954  1.14449  1.62773
H   1.69937 -4.23859 -1.92192
H   0.07244 -0.70094  1.16126
C  -1.31254 -2.19557  1.87670
C  -0.53404 -2.48764  3.00296
C  -0.85647 -3.56730  3.82712
C  -1.95859 -4.36934  3.52846
C  -2.73494 -4.08803  2.40175
C  -2.41571 -3.00633  1.58159
H   0.33217 -1.87135  3.23134
H  -0.24139 -3.78532  4.69535
H  -2.20709 -5.21373  4.16473
H  -3.58742 -4.71532  2.15774
H  -3.01266 -2.80451  0.69681
H  -3.96008  2.52299  1.55586
C  -5.03493  3.30610 -0.15553
H  -5.55078  4.06564  0.42341
C  -5.27054  3.20385 -1.53115
H  -5.96850  3.88279 -2.01052
C  -4.61438  2.23704 -2.29928
H  -4.79379  2.16384 -3.36769

SCF  =           -1521.21710428
H(0 K)=          -1520.726982
H(298 K)=        -1520.696072
G(298 K)=        -1520.793768
SCF (BS2) =      -1521.58304195
SCF (DCM) =      -1521.23456151
Lowest Frequency = 9.3588cm-1

62
TS1cis
H  -3.40121 -4.44567  0.83463
C  -5.02655 -3.85477 -0.60690
C  -3.84909 -3.70944  0.18253
H  -5.64960 -4.73602 -0.68217
H   3.45357 -4.41237 -0.69211
H   4.94622 -4.17026  1.28358
C   3.96708 -3.69873  1.27758
C   3.12724 -3.83560  0.16966
H   1.23445 -3.34447 -0.71273
C   1.86719 -3.23562  0.16211
C   1.42148 -2.48353  1.26184
C   2.28097 -2.34660  2.36367
C   3.53671 -2.95120  2.37667
H   4.18116 -2.84269  3.24566
H   1.92740 -1.75917  3.20460
H  -6.00282 -2.36109 -1.97276
O  -0.05587 -0.87637  2.20205
H  -0.76494 -2.95883 -0.30166
C  -0.93972 -2.18308  0.43350
C   0.07899 -1.78755  1.32176
C  -2.25255 -1.61366  0.51318
H  -2.42552 -1.15128  1.48739
C  -3.38861 -2.41879 -0.01129
C  -5.24146 -2.65816 -1.26615
```

```
N  -4.24373 -1.79578 -0.88304
C  -3.86683 -0.47584 -1.37322
C  -2.69184 -0.02142 -0.51948
H  -2.95283  0.68491  0.26311
H  -3.57548 -0.53253 -2.42761
O  -1.14053 -0.09556 -2.34844
C  -1.48769  0.30237 -1.23428
S   1.54028  0.34997 -2.32626
C   3.12879  0.81863 -1.64112
C   2.99367  1.38409 -0.35940
C   4.10437  1.80881  0.36828
N   1.65361  1.43874  0.01674
C   0.79905  0.93885 -0.88163
N  -0.47397  1.00008 -0.48790
C  -0.55530  1.62678  0.87299
H   1.18589  2.86420  1.50977
C   0.95441  1.82782  1.25279
H   1.22318  1.13791  2.05543
H  -4.71507  0.21319 -1.28506
H  -0.94521  0.86755  1.55694
C  -1.37431  2.90003  0.88736
C  -2.19082  3.16389  1.99426
C  -2.91702  4.35374  2.07190
C  -2.83978  5.28834  1.03866
C  -2.03296  5.02913 -0.07207
C  -1.30213  3.84380 -0.14652
H  -2.25799  2.43263  2.79571
H  -3.54820  4.54436  2.93483
H  -3.40932  6.21131  1.09434
H  -1.97479  5.74977 -0.88259
H  -0.68631  3.64704 -1.02015
H   3.99233  2.23478  1.35952
C   5.36016  1.65657 -0.21843
H   6.24203  1.97534  0.32773
C   5.50157  1.09648 -1.49493
H   6.49065  0.98774 -1.92777
C   4.38643  0.66958 -2.21895
H   4.49883  0.22867 -3.20437

SCF  =          -1521.19590092
H(0 K)=         -1520.705475
H(298 K)=       -1520.675799
G(298 K)=       -1520.770339
SCF (BS2) =     -1521.56152638
SCF (DCM) =     -1521.21664124
Lowest Frequency = -293.6045cm-1

62
46cis
H  -3.69813 -5.43636 -1.05402
C  -2.87709 -4.72421 -1.06363
C  -2.56834 -4.01611 -2.22732
H  -3.14881 -4.17980 -3.13238
C  -1.06435 -3.59332  0.07923
H  -1.26297 -2.53508 -3.12479
C  -1.51656 -3.10033 -2.23405
H  -0.49678 -3.42553  0.98935
C  -0.73824 -2.87947 -1.08648
C  -2.12046 -4.50518  0.09080
H  -2.35487 -5.04537  1.00498
C   0.38199 -1.86544 -1.17436
O   0.31803 -1.03536 -2.16188
H   1.43274 -2.66912  0.52428
C  -1.13933  0.87784  0.82780
C  -3.32258  1.08592  0.15135
C  -3.49036  0.64670  1.47821
S  -1.91520  0.38143  2.28645
C   1.38148 -1.87676 -0.21359
C   1.16158  0.39659  1.30705
O   0.84065  0.12843  2.45411
C   2.57370 -0.95617 -0.32047
C   2.54499  0.27046  0.70931
H   2.57700 -0.55257 -1.33964
N   0.14943  0.93769  0.46692
C  -4.41909  1.33391 -0.67413
N  -1.97162  1.22020 -0.16079
C  -1.22768  1.40189 -1.42040
H  -1.55224  2.30629 -1.93728
H  -1.32801  0.50156 -2.03895
C   0.25493  1.48271 -0.93014
C   3.62029 -0.01448  1.78837
H   3.16597 -0.39816  2.70779
H   4.19855  0.88143  2.03870
C   5.63781 -1.63242  1.45887
H   6.22525 -1.33528  2.31514
C   5.85166 -2.60325  0.49630
N   4.44994 -1.01709  1.14504
C   3.90110 -1.57938  0.02068
C   4.75257 -2.57545 -0.41480
H   4.60716 -3.20685 -1.27969
H   6.71205 -3.25747  0.44653
H   2.78711  1.20126  0.18424
C   0.86867  2.86659 -0.96825
H   0.79412  0.74290 -1.53643
C   0.61850  3.83169  0.01727
C   1.16946  5.10905 -0.08800
C   1.97295  5.43922 -1.18152
C   2.22796  4.48402 -2.16672
C   1.68258  3.20397 -2.05827
H   0.00378  3.58538  0.87898
H   0.97271  5.84571  0.68552
H   2.40294  6.43326 -1.26099
H   2.85976  4.72984 -3.01507
H   1.89084  2.45749 -2.82031
H  -4.28200  1.66063 -1.69922
C  -5.69063  1.13799 -0.13843
H  -6.56100  1.31955 -0.76049
C  -5.86340  0.70830  1.18477
H  -6.86491  0.56320  1.57622
C  -4.76426  0.45616  2.00723
H  -4.89992  0.11381  3.02810

SCF  =          -1521.19901372
H(0 K)=         -1520.707279
H(298 K)=       -1520.677552
G(298 K)=       -1520.770951
SCF (BS2) =     -1521.56395238
SCF (DCM) =     -1521.22271082
Lowest Frequency = 15.3108cm-1

62
TS2cis
```

```
H   2.60408  6.55665 -0.84270
C   1.96150  5.68020 -0.82937
C   2.50569  4.40185 -0.96850
H   3.57937  4.27931 -1.09290
C  -0.24791  4.69928 -0.67076
H   2.07747  2.28077 -1.07409
C   1.67579  3.28115 -0.95377
H  -1.32151  4.83073 -0.58135
C   0.28715  3.40602 -0.79433
C   0.57851  5.82180 -0.68388
H   0.14116  6.81254 -0.58817
C  -0.55111  2.15666 -0.78484
O   0.02510  1.04513 -1.11591
H  -2.35189  3.06306  0.01981
C   1.41787 -0.92350  0.69672
C   3.56431 -1.19011 -0.08650
C   3.79959 -0.53317  1.13620
S   2.26823 -0.13567  1.97497
C  -1.87293  2.18272 -0.38809
C  -0.82836 -0.23322  1.12269
O  -0.41556  0.38520  2.09245
C  -2.64176  0.90956 -0.44206
C  -2.24359 -0.24634  0.65471
H  -2.47743  0.43005 -1.41674
N   0.11730 -1.07882  0.45000
C   4.61550 -1.55012 -0.92943
N   2.20181 -1.40192 -0.28153
C   1.39562 -1.72973 -1.46596
H   1.66943 -2.70596 -1.87011
H   1.52645 -0.94120 -2.21227
C  -0.06291 -1.71130 -0.89669
C  -3.24275 -0.02332  1.81282
H  -2.84241  0.69269  2.53801
H  -3.48749 -0.95381  2.33584
C  -5.71983  0.71141  1.42167
H  -6.14768  0.36003  2.34903
C  -6.28448  1.38299  0.35185
N  -4.39709  0.51002  1.11146
C  -4.10430  1.04894 -0.11622
C  -5.26356  1.60231 -0.62294
H  -5.37198  2.10139 -1.57557
H  -7.32364  1.67328  0.27164
H  -2.46548 -1.20672  0.18101
C  -0.71721 -3.07550 -0.82591
H  -0.64001 -0.99831 -1.48579
C  -0.34045 -4.02790  0.13139
C  -0.92987 -5.29155  0.13965
C  -1.90015 -5.61975 -0.81065
C  -2.28195 -4.67707 -1.76570
C  -1.69469 -3.41042 -1.77096
H   0.40460 -3.77723  0.88175
H  -0.63475 -6.01937  0.88984
H  -2.36022 -6.60338 -0.80130
H  -3.04240 -4.92150 -2.50128
H  -2.00071 -2.67367 -2.50924
H   4.42743 -2.05122 -1.87304
C   5.91210 -1.23998 -0.51994
H   6.74748 -1.50609 -1.15945
C   6.15238 -0.59187  0.69876
H   7.17089 -0.36264  0.99454
C   5.09722 -0.22988  1.53904
H   5.28436  0.28267  2.47724

SCF  =           -1521.19600566
H(0 K)=          -1520.704596
H(298 K)=        -1520.675578
G(298 K)=        -1520.767003
SCF (BS2) =      -1521.56234363
SCF (DCM) =      -1521.21960278
Lowest Frequency = -25.1674cm-1

30
24
C  -0.38716 -1.04090 -0.60999
C   0.53487  1.05823 -0.53275
H  -0.63320 -1.46375  0.37309
H  -0.34930 -1.84876 -1.34544
N  -0.68184  1.38547 -0.74544
N   0.86561 -0.29253 -0.55478
S   1.96458  2.04383 -0.16412
C  -2.73018 -0.00609 -0.32939
C  -3.70166 -0.87667 -0.84020
C  -3.00823  0.70780  0.84149
C  -4.92385 -1.04191 -0.18784
H  -3.50316 -1.42435 -1.75921
C  -4.23304  0.54827  1.49159
H  -2.26571  1.40120  1.22261
C  -5.19235 -0.32911  0.98244
H  -5.66808 -1.71925 -0.59737
H  -4.43977  1.11433  2.39570
H  -6.14553 -0.45092  1.48907
C  -1.38688  0.11041 -1.02239
H  -1.55292  0.05110 -2.10741
C   2.69482 -1.87263  0.07446
C   4.02533 -1.96359  0.49527
C   4.79099 -0.81738  0.71978
C   4.23496  0.45495  0.53419
C   2.91248  0.55139  0.12363
C   2.14009 -0.60668 -0.11455
H   2.10306 -2.76443 -0.10553
H   4.46612 -2.94416  0.64716
H   5.82276 -0.90733  1.04406
H   4.82494  1.34800  0.71619

SCF  =           -698.524932235
H(0 K)=          -698.289372
H(298 K)=        -698.275635
G(298 K)=        -698.331954
SCF (DCM) =      -698.532436265
SCF (BS2) =      -698.678554617
Lowest Frequency = 19.5982cm-1

32
21cis
H   5.89536 -1.56048 -0.59037
C   4.89170 -1.19302 -0.39755
C   4.65571  0.17650 -0.27267
H   5.47684  0.88147 -0.36524
C   2.53680 -1.61934 -0.04389
H   3.18802  1.71579  0.06688
C   3.36769  0.65136 -0.02833
H   1.71286 -2.32387  0.00800
```

```
C   2.29163 -0.24270  0.10080
C   3.82525 -2.08867 -0.28420
H   3.99492 -3.15556 -0.39698
C   0.93239  0.25933  0.38638
O   0.81363  1.61420  0.05846
H   0.08974 -1.45999  1.22826
C  -0.07664 -0.43074  0.93433
C  -0.37941  2.28034  0.11081
O  -0.38654  3.47143 -0.07231
C  -1.44944  0.15164  1.15880
C  -1.63171  1.45645  0.32777
H  -1.60174  0.34824  2.22871
C  -2.24947  0.98687 -1.02743
H  -1.47652  0.75104 -1.77032
H  -2.91818  1.74036 -1.45083
C  -3.90359 -1.03609 -1.17524
H  -4.36752 -0.81078 -2.12393
C  -4.09708 -2.08416 -0.29294
N  -2.96097 -0.21034 -0.61530
C  -2.54394 -0.71796  0.59113
C  -3.23439 -1.88826  0.82851
H  -3.14761 -2.52578  1.69717
H  -4.79755 -2.89673 -0.42950
H  -2.35167  2.11453  0.81830

SCF  =           -822.710561318
H(0 K)=          -822.455345
H(298 K)=        -822.441032
G(298 K)=        -822.498116
SCF (BS2) =      -822.921719275
SCF (DCM) =      -822.719298926
Lowest Frequency = 35.4388cm-1
```

(b) 21trans formation from (Z)-45

```
62
TS1trans
C   2.09691  1.39411 -0.48819
C   0.82165  2.00547 -0.46904
C   0.74522 -1.92316 -0.80654
H   2.93138  1.83770  0.04292
C   5.47467 -1.50746 -1.90703
H   5.98228 -2.46093 -1.88293
C   4.84051  0.66280 -2.07281
H   4.83596  1.72603 -2.26636
C   5.91955 -0.24735 -2.26527
H   6.91441 -0.00636 -2.61503
N   4.16568 -1.38332 -1.51463
C   3.76392 -0.07675 -1.61292
C   3.20145 -2.35581 -1.01096
H   2.82132 -2.98327 -1.82405
H   3.69037 -3.00479 -0.27416
O   0.40998 -2.52922 -1.83004
O  -0.21312  1.49010 -0.98785
C   0.65375  3.34276  0.22314
C  -0.50319  4.08479 -0.06614
C   1.56565  3.87123  1.15166
C  -0.73577  5.31940  0.53736
H  -1.20373  3.66262 -0.77921
C   1.32936  5.10167  1.76563
H   2.46082  3.31572  1.41208
C   0.17990  5.83347  1.45880
H  -1.63140  5.88353  0.28915
H   2.04462  5.48971  2.48622
H  -0.00011  6.79388  1.93424
C  -1.68031  0.15500  1.24516
C  -1.54266 -1.22312 -0.60972
H  -2.23614 -0.03773  2.16614
H  -1.55644  1.22447  1.06894
N  -0.35177 -1.37538 -0.03853
N  -2.36916 -0.44160  0.09284
S  -2.20028 -1.81191 -2.09452
C   2.35295  0.22369 -1.26123
C   2.06771 -1.55226 -0.39800
H   2.19431 -1.30642  0.65142
H   1.62916  0.07017 -2.06451
C  -0.05240 -1.45728  2.44818
C   0.77737 -0.97832  3.46912
C  -0.66935 -2.70613  2.59993
C   0.98225 -1.73045  4.62714
H   1.26839 -0.01528  3.35375
C  -0.46009 -3.46014  3.75423
H  -1.30083 -3.09892  1.80773
C   0.36393 -2.97296  4.77169
H   1.63125 -1.34891  5.40978
H  -0.93727 -4.43029  3.85764
H   0.52774 -3.56256  5.66884
C  -0.29557 -0.58626  1.23035
H   0.50125  0.15042  1.12744
C  -4.65147  0.57688 -0.03463
C  -5.80805  0.65324 -0.81053
C  -5.91532 -0.04085 -2.02258
C  -4.86212 -0.82821 -2.49347
C  -3.70336 -0.90798 -1.72645
C  -3.60375 -0.21195 -0.50728
H  -4.56423  1.11775  0.90163
H  -6.63675  1.26407 -0.46713
H  -6.82603  0.03554 -2.60765
H  -4.94370 -1.35873 -3.43693

SCF  =           -1521.19632754
H(0 K)=          -1520.705726
H(298 K)=        -1520.676074
G(298 K)=        -1520.769816
SCF (BS2) =      -1521.56191770
SCF (DCM) =      -1521.21549970
Lowest Frequency = -319.1932cm-1

62
46trans
C   2.03118  1.46033 -0.40469
C   0.73232  1.94902 -0.46554
C   0.81299 -1.69215 -0.95540
H   2.82521  2.00737  0.09003
C   5.66442 -1.20955 -1.79076
H   6.20070 -2.14717 -1.79316
C   4.96218  0.95377 -1.89014
H   4.94311  2.02574 -2.02504
C   6.08048  0.08150 -2.06275
H   7.08546  0.37092 -2.33992
N   4.33473 -1.13576 -1.45228
C   3.89508  0.16126 -1.51774
```

```
C   3.29871 -2.10500 -1.14359
H   2.96110 -2.63253 -2.04251
H   3.64966 -2.84475 -0.41579
O   0.53584 -2.29549 -1.98192
O  -0.23461  1.33879 -1.05298
C   0.39198  3.25255  0.22921
C  -0.80492  3.89291 -0.13190
C   1.18231  3.84819  1.22715
C  -1.19108  5.09316  0.46483
H  -1.41047  3.41743 -0.89680
C   0.79468  5.04408  1.83150
H   2.10099  3.36549  1.54627
C  -0.39291  5.67565  1.45185
H  -2.11558  5.57724  0.15846
H   1.41988  5.48359  2.60495
H  -0.69207  6.60850  1.92242
C  -1.64502  0.09831  1.24164
C  -1.50649 -1.25864 -0.62903
H  -2.15523 -0.11515  2.18418
H  -1.58562  1.16983  1.04566
N  -0.27544 -1.33395 -0.11046
N  -2.34663 -0.54753  0.12124
S  -2.18190 -1.85375 -2.09954
C   2.42608  0.27651 -1.21947
C   2.17146 -1.21462 -0.57290
H   2.27553 -1.11927  0.50869
H   1.84104  0.27942 -2.15139
C   0.09804 -1.45286  2.36232
C   1.00891 -0.99140  3.32046
C  -0.51269 -2.70112  2.54718
C   1.29936 -1.75957  4.44970
H   1.49387 -0.02940  3.17620
C  -0.21879 -3.47058  3.67220
H  -1.21033 -3.08073  1.80507
C   0.68615 -3.00007  4.62735
H   2.00955 -1.39108  5.18390
H  -0.69357 -4.43870  3.80256
H   0.91513 -3.60134  5.50222
C  -0.22678 -0.56456  1.17786
H   0.52841  0.21749  1.06791
C  -4.69006  0.33031  0.10352
C  -5.87802  0.34837 -0.62535
C  -5.99086 -0.32910 -1.84745
C  -4.91230 -1.03934 -2.37705
C  -3.72112 -1.05964 -1.65594
C  -3.61566 -0.38336 -0.42656
H  -4.59794  0.85891  1.04615
H  -6.72849  0.90015 -0.23834
H  -6.92766 -0.29871 -2.39416
H  -4.99842 -1.55501 -3.32811

SCF  =           -1521.20060953
H(0 K)=          -1520.708644
H(298 K)=        -1520.678899
G(298 K)=        -1520.772280
SCF (BS2) =      -1521.56705130
SCF (DCM) =      -1521.22277090
Lowest Frequency = 16.6390cm-1

62
TS2trans
C   2.36726  1.67892 -0.61839
C   1.03859  2.03037 -0.56249
C   0.68337 -1.14668 -1.18576
H   3.15866  2.26041 -0.16194
C   5.63528 -1.67227 -1.39175
H   5.96018 -2.69931 -1.31232
C   5.42069  0.58583 -1.59471
H   5.65024  1.63335 -1.72958
C   6.34728 -0.50272 -1.58780
H   7.42142 -0.43385 -1.69751
N   4.31349 -1.31721 -1.26814
C   4.16771  0.04164 -1.39942
C   3.05581 -2.03557 -1.14620
H   2.74581 -2.46826 -2.10451
H   3.12242 -2.83650 -0.40215
O   0.38637 -1.55624 -2.29801
O   0.11564  1.28673 -1.09556
C   0.57879  3.26487  0.16853
C  -0.73108  3.72149 -0.05663
C   1.37551  3.97437  1.08373
C  -1.22464  4.85101  0.59743
H  -1.33722  3.17077 -0.76878
C   0.88348  5.10195  1.73995
H   2.38430  3.63363  1.29545
C  -0.41984  5.54782  1.50106
H  -2.23743  5.19297  0.39676
H   1.51763  5.63244  2.44599
H  -0.80101  6.42686  2.01381
C  -1.78828  0.08675  1.33543
C  -1.64452 -1.04839 -0.67171
H  -2.22002 -0.22447  2.28870
H  -1.81421  1.17483  1.23396
N  -0.39099 -1.07784 -0.22527
N  -2.50900 -0.52299  0.20848
S  -2.34851 -1.50640 -2.18016
C   2.71661  0.42714 -1.35157
C   2.08549 -0.90407 -0.71488
H   2.12892 -0.79566  0.36966
H   2.30286  0.46882 -2.37292
C   0.09116 -1.46095  2.20419
C   1.00819 -1.05666  3.18182
C  -0.43650 -2.75844  2.25896
C   1.38653 -1.93018  4.20326
H   1.43188 -0.05654  3.13743
C  -0.05535 -3.63238  3.27648
H  -1.13586 -3.09362  1.49766
C   0.85495 -3.21915  4.25264
H   2.10193 -1.60494  4.95261
H  -0.46600 -4.63744  3.30572
H   1.15243 -3.90189  5.04300
C  -0.33570 -0.46746  1.14096
H   0.35793  0.37018  1.09646
C  -4.92943  0.07942  0.42408
C  -6.15879  0.05304 -0.23430
C  -6.27887 -0.47769 -1.52521
C  -5.16667 -0.99156 -2.19594
C  -3.93530 -0.96718 -1.54707
C  -3.82221 -0.43841 -0.24714
H  -4.83378  0.49220  1.42275
H  -7.03561  0.45256  0.26493
H  -7.24711 -0.48753 -2.01495
```

```
H  -5.25988 -1.39341 -3.19989

SCF  =          -1521.19957720
H(0 K)=         -1520.707826
H(298 K)=       -1520.678926
G(298 K)=       -1520.769906
SCF (BS2) =     -1521.56565989
SCF (DCM) =     -1521.22222157
Lowest Frequency = -23.0392cm-1

32
21trans
C   0.08104 -0.81725 -0.32921
C   1.10092  0.05674 -0.25303
C  -0.36009  2.01993 -0.19182
H   0.25177 -1.88130 -0.22863
C  -4.67877 -0.51082  0.51087
H  -5.50879  0.07992  0.86917
C  -3.24158 -2.13330 -0.19302
H  -2.83841 -3.09326 -0.48350
C  -4.57414 -1.86484  0.24769
H  -5.36609 -2.59050  0.37288
N  -3.44981  0.04471  0.25981
C  -2.57016 -0.93004 -0.17071
C  -2.88620  1.39678  0.27254
H  -3.16919  1.96395 -0.62290
H  -3.20235  1.95817  1.15494
O  -0.49471  3.19930 -0.38623
O   0.87823  1.43758 -0.39007
C   2.53206 -0.24749 -0.06302
C   3.42796  0.75769  0.33794
C   3.02751 -1.54699 -0.27167
C   4.77552  0.46409  0.54339
H   3.06205  1.76711  0.48433
C   4.37263 -1.83602 -0.06197
H   2.36303 -2.33183 -0.61844
C   5.25357 -0.83201  0.34895
H   5.45297  1.25359  0.85557
H   4.73684 -2.84522 -0.23100
H   6.30404 -1.05802  0.50678
C  -1.28888 -0.25340 -0.56382
C  -1.39325  1.04113  0.28024
H  -1.11396  0.75738  1.30502
H  -1.36023  0.02686 -1.63259

SCF  =          -822.703471174
H(0 K)=         -822.448741
H(298 K)=       -822.434475
G(298 K)=       -822.491180
SCF (BS2) =     -822.914217548
SCF (DCM) =     -822.712193029
Lowest Frequency = 26.9661cm-1
```

(c)21trans formation from (E)-45

```
62
(E)-45
C  -1.79394  2.61065  0.05195
C  -2.41741  1.34215  0.42295
C   2.05305  0.29393 -1.72917
H  -2.40234  3.50372 -0.02883
C   2.20680  4.94128 -0.96942
H   3.26899  5.09938 -1.09309
C  -0.02504  5.11604 -0.82275
H  -1.03785  5.49495 -0.83077
C   1.14606  5.83615 -1.09518
H   1.22820  6.88201 -1.35513
N   1.72670  3.71558 -0.63945
C   0.33718  3.78815 -0.54289
C   2.59144  2.51042 -0.44398
H   3.59323  2.93282 -0.29662
H   2.31548  2.07042  0.51406
O   1.99380 -0.42286 -2.76197
O  -1.76052  0.29200  0.53157
C  -3.89885  1.29561  0.67876
C  -4.50541  0.03161  0.76549
C  -4.69614  2.43833  0.85104
C  -5.87147 -0.08836  1.00235
H  -3.87667 -0.84325  0.64043
C  -6.06383  2.31851  1.09838
H  -4.25529  3.42844  0.81339
C  -6.65598  1.05652  1.16976
H  -6.32770 -1.07285  1.05733
H  -6.66660  3.21159  1.23626
H  -7.72231  0.96527  1.35671
C   0.57604 -1.42696  1.46301
C   0.71374 -1.51898 -0.83758
H   1.03402 -2.08471  2.20549
H  -0.32653 -0.95494  1.85666
N   1.42120 -0.43458 -0.55697
N   0.24171 -2.16134  0.24421
S   0.28772 -2.25145 -2.34592
C  -0.45586  2.65626 -0.19746
C   2.57835  1.55137 -1.58225
H   3.00500  1.92625 -2.50776
H   0.05628  1.70324 -0.13138
C   3.02679 -0.60539  1.36727
C   3.42517 -0.15696  2.63378
C   3.94025 -1.30773  0.57263
C   4.71049 -0.41964  3.10769
H   2.72723  0.40370  3.25221
C   5.22752 -1.56602  1.04674
H   3.65582 -1.63383 -0.42276
C   5.61496 -1.12784  2.31405
H   5.00664 -0.06454  4.09053
H   5.93023 -2.10563  0.41869
H   6.61825 -1.32904  2.67804
C   1.59410 -0.35634  0.92369
H   1.26395  0.62058  1.27837
C  -1.26635 -4.05485  0.87794
C  -2.04620 -5.09436  0.36737
C  -2.14765 -5.31137 -1.01181
C  -1.47147 -4.48818 -1.91716
C  -0.69734 -3.44419 -1.42002
C  -0.59629 -3.23830 -0.03139
H  -1.18660 -3.88420  1.94652
H  -2.58119 -5.74247  1.05443
H  -2.75983 -6.12616 -1.38525
H  -1.55352 -4.65720 -2.98671

SCF  =          -1521.20776626
H(0 K)=         -1520.717444
```

```
H(298 K)=        -1520.686680
G(298 K)=        -1520.784418
SCF (BS2) =      -1521.57418896
SCF (DCM) =      -1521.22656178
Lowest Frequency = 6.2476cm-1

62
TS1trans(E)
C   0.48206  2.85157 -0.02894
C   1.48589  1.92948 -0.42882
C  -0.97100  0.31060  1.65116
H   0.73562  3.79144  0.44636
C  -4.20635  3.81719  0.00503
H  -5.21964  3.62353  0.32642
C  -2.24663  4.66603 -0.76344
H  -1.51025  5.31020 -1.22250
C  -3.64145  4.92103 -0.60781
H  -4.17034  5.81885 -0.89791
N  -3.20012  2.90700  0.21585
C  -2.00373  3.40797 -0.24296
C  -3.16029  1.60484  0.88087
H  -3.88363  1.60154  1.70576
H  -3.45740  0.80232  0.19453
O  -0.18095  0.18723  2.59654
O   1.22677  0.75324 -0.82542
C   2.93575  2.34273 -0.37971
C   3.91331  1.34187 -0.49098
C   3.35867  3.67535 -0.25198
C   5.27037  1.65646 -0.45649
H   3.57287  0.31900 -0.61131
C   4.71634  3.99374 -0.22180
H   2.62481  4.47265 -0.19701
C   5.67828  2.98568 -0.31941
H   6.01275  0.86646 -0.54011
H   5.02432  5.03182 -0.12791
H   6.73575  3.23516 -0.29512
C  -0.48413 -1.71698 -1.47792
C   0.23030 -1.52176  0.69640
H  -0.90135 -2.53437 -2.06785
H   0.06499 -1.00211 -2.09763
N  -0.87626 -0.77547  0.67858
N   0.42435 -2.21040 -0.43655
S   1.48616 -1.68790  1.86354
C  -0.86754  2.46521 -0.16486
C  -1.72908  1.49151  1.37458
H  -1.54827  2.18633  2.19273
H  -0.95075  1.63802 -0.87011
C  -2.85960 -1.79712 -0.47290
C  -3.82977 -1.69157 -1.47974
C  -3.08710 -2.66371  0.60187
C  -5.00087 -2.44611 -1.41915
H  -3.67083 -1.01030 -2.31271
C  -4.26166 -3.41638  0.66395
H  -2.35619 -2.74005  1.40072
C  -5.21898 -3.31260 -0.34590
H  -5.74537 -2.34983 -2.20387
H  -4.42825 -4.08139  1.50625
H  -6.13286 -3.89680 -0.29372
C  -1.57490 -0.99561 -0.61960
H  -1.79172 -0.03044 -1.07251
C   2.12613 -3.67789 -1.54029
C   3.35473 -4.31200 -1.35698
C   4.06118 -4.18868 -0.15326
C   3.55826 -3.41869  0.89746
C   2.33441 -2.77801  0.72105
C   1.62771 -2.91195 -0.48754
H   1.57811 -3.77161 -2.47186
H   3.76803 -4.91004 -2.16277
H   5.01444 -4.69371 -0.03520
H   4.11248 -3.31503  1.82508

SCF  =           -1521.17910204
H(0 K)=          -1520.688842
H(298 K)=        -1520.659264
G(298 K)=        -1520.752283
SCF (BS2) =      -1521.54548644
SCF (DCM) =      -1521.19981767
Lowest Frequency = -319.2877cm-1
```

1.7 References and Notes

[1] K. R. Law, C. S. P. McErlean, *Chem. Eur. J.* **2013**, *19*, 15852-15855.

[2] B. A. Trofimov, A. b. I. Mikhaleva, A. V. Ivanov, V. S. Shcherbakova, I. A. Ushakov, *Tetrahedron* **2015**, *71*, 124-128.

[3] L. Sun, N. Tran, C. Liang, S. Hubbard, F. Tang, K. Lipson, R. Schreck, Y. Zhou, G. McMahon, C. Tang, *J. Med. Chem.* **2000**, *43*, 2655-2663.

[4] T. P. Robinson, R. B. Hubbard Iv, T. J. Ehlers, J. L. Arbiser, D. J. Goldsmith, J. P. Bowen, *Biorg. Med. Chem.* **2005**, *13*, 4007-4013.

[5] N. R. El-Rayyes, *J. Heterocycl. Chem.* **1982**, *19*, 415-419.

[6] W. Herz, J. A. Y. Brasch, *J. Org. Chem.* **1958**, *23*, 1513-1516.

[7] I. A. Ushakov, A. V. Afonin, V. K. Voronov, Z. V. Stepanova, L. N. Sobenina, A. I. Mikhaleva, *Russ. J. Org. Chem.* **2003**, *39*, 1318-1324.

[8] Gaussian 09, Revision D.01, M. J. Frisch, G. W. Trucks, H. B. Schlegel, G. E. Scuseria, M. A. Robb, J. R. Cheeseman, G. Scalmani, V. Barone, B. Mennucci, G. A. Petersson, H. Nakatsuji, M. Caricato, X. Li, H. P. Hratchian, A. F. Izmaylov, J. Bloino, G. Zheng, J. L. Sonnenberg, M. Hada, M. Ehara, K. Toyota, R. Fukuda, J. Hasegawa, M. Ishida, T. Nakajima, Y. Honda, O. Kitao, H. Nakai, T. Vreven, J. A. Montgomery, Jr., J. E. Peralta, F. Ogliaro, M. Bearpark, J. J. Heyd, E. Brothers, K. N. Kudin, V. N. Staroverov, R. Kobayashi, J. Normand, K. Raghavachari, A. Rendell, J. C. Burant, S. S. Iyengar, J. Tomasi, M. Cossi, N. Rega, J. M. Millam, M. Klene, J. E. Knox, J. B. Cross, V. Bakken, C. Adamo, J. Jaramillo, R. Gomperts, R. E. Stratmann, O. Yazyev, A. J. Austin, R. Cammi, C. Pomelli, J. W. Ochterski, R. L. Martin, K. Morokuma, V. G. Zakrzewski, G. A. Voth, P. Salvador, J. J. Dannenberg, S. Dapprich, A. D. Daniels, Ö. Farkas, J. B. Foresman, J. V. Ortiz, J. Cioslowski, and D. J. Fox, Gaussian, Inc., Wallingford CT, 2009.

[9] Y. Zhao and D. G. Truhlar, *Theor. Chem. Acc.*, 2008, *120* 215

[10] (i) W. J. Hehre, R. Ditchfield and J. A. Pople, *J. Chem. Phys.* **1972**, *56*, 2257. (ii) P. C. Hariharan and J. A. Pople, *Theor. Chim. Acta.* **1973**, *28*, 213.

[11] (i) A. D. McLean and G. S. Chandler, *J. Chem. Phys.*, **1980** *72*, 5639 (ii) K. Raghavachari, J. S. Binkley, R. Seeger, and J. A. Pople, *J. Chem. Phys.*,**1980**, *72*, 650; (iii) T. Clark, J. Chandrasekhar, G. W. Spitznagel, and P. v. R. Schleyer, *J. Comp. Chem.*, **1983**, *4*, 294

[12] J. Tomasi, B. Mennucci, and R. Cammi, *Chem. Rev.*, **2005**, *105,* 2999

[13] A. D. Becke, *J. Chem. Phys.*, **1993**, *98*, 5648

1.8 ^{1}H NMR, ^{13}C{^{1}H} NMR and HPLC Data for Novel Compounds

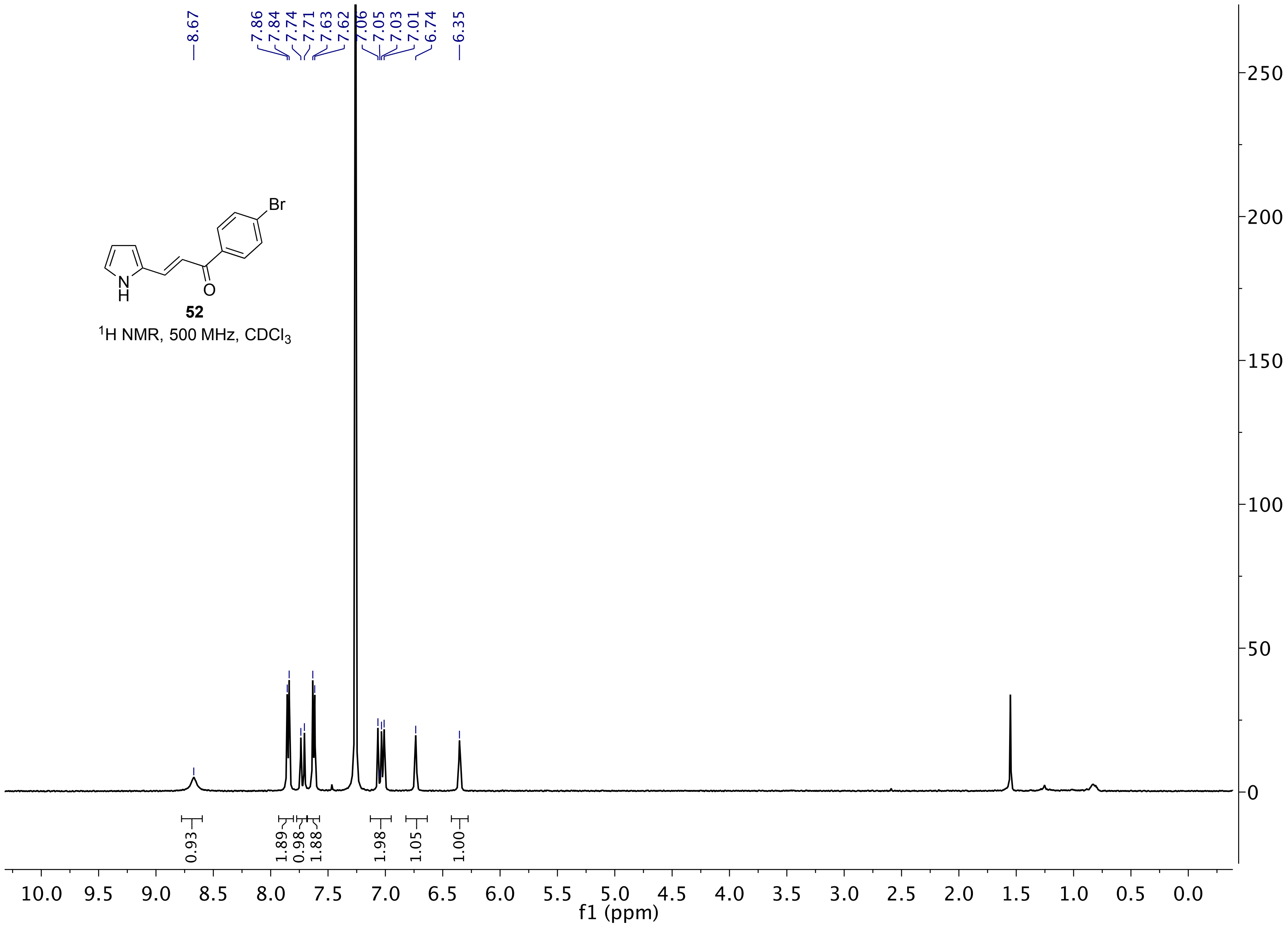
8.67
7.86
7.84
7.74
7.71
7.63
7.62
7.06
7.05
7.03
7.01
6.74
6.35
Br
N
H
O
52
^{1}H NMR, 500 MHz, $CDCl_3$
0.93
1.89
0.98
1.88
1.98
1.05
1.00
10.0 9.5 9.0 8.5 8.0 7.5 7.0 6.5 6.0 5.5 5.0 4.5 4.0 3.5 3.0 2.5 2.0 1.5 1.0 0.5 0.0
f1 (ppm)
250
200
150
100
50
0

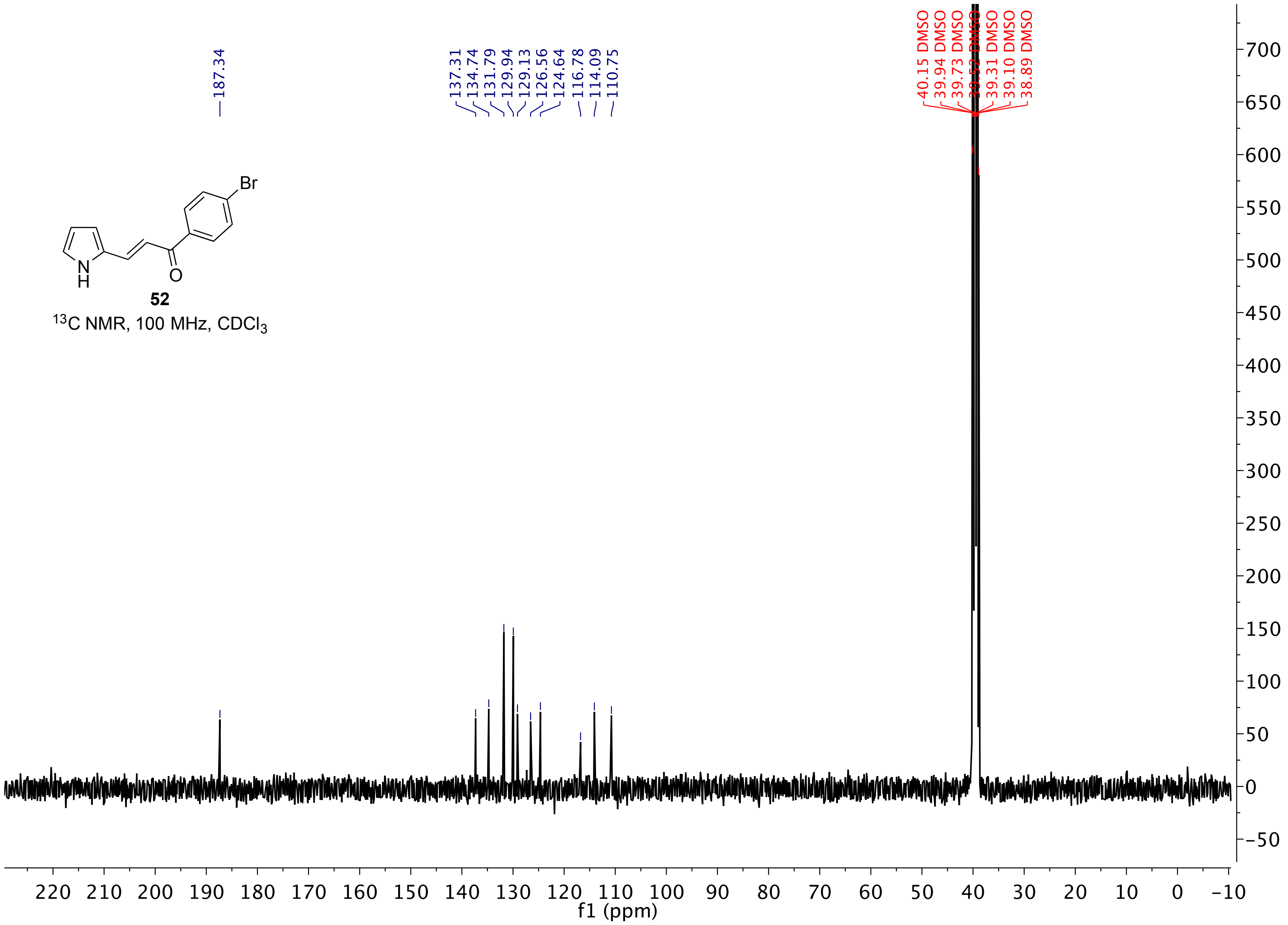
187.34
137.31
134.74
131.79
129.94
129.13
126.56
124.64
116.78
114.09
110.75
40.15 DMSO
39.94 DMSO
39.73 DMSO
39.52 DMSO
39.31 DMSO
39.10 DMSO
38.89 DMSO
Br
N
H
O
52
^{13}C NMR, 100 MHz, $CDCl_3$
f1 (ppm)

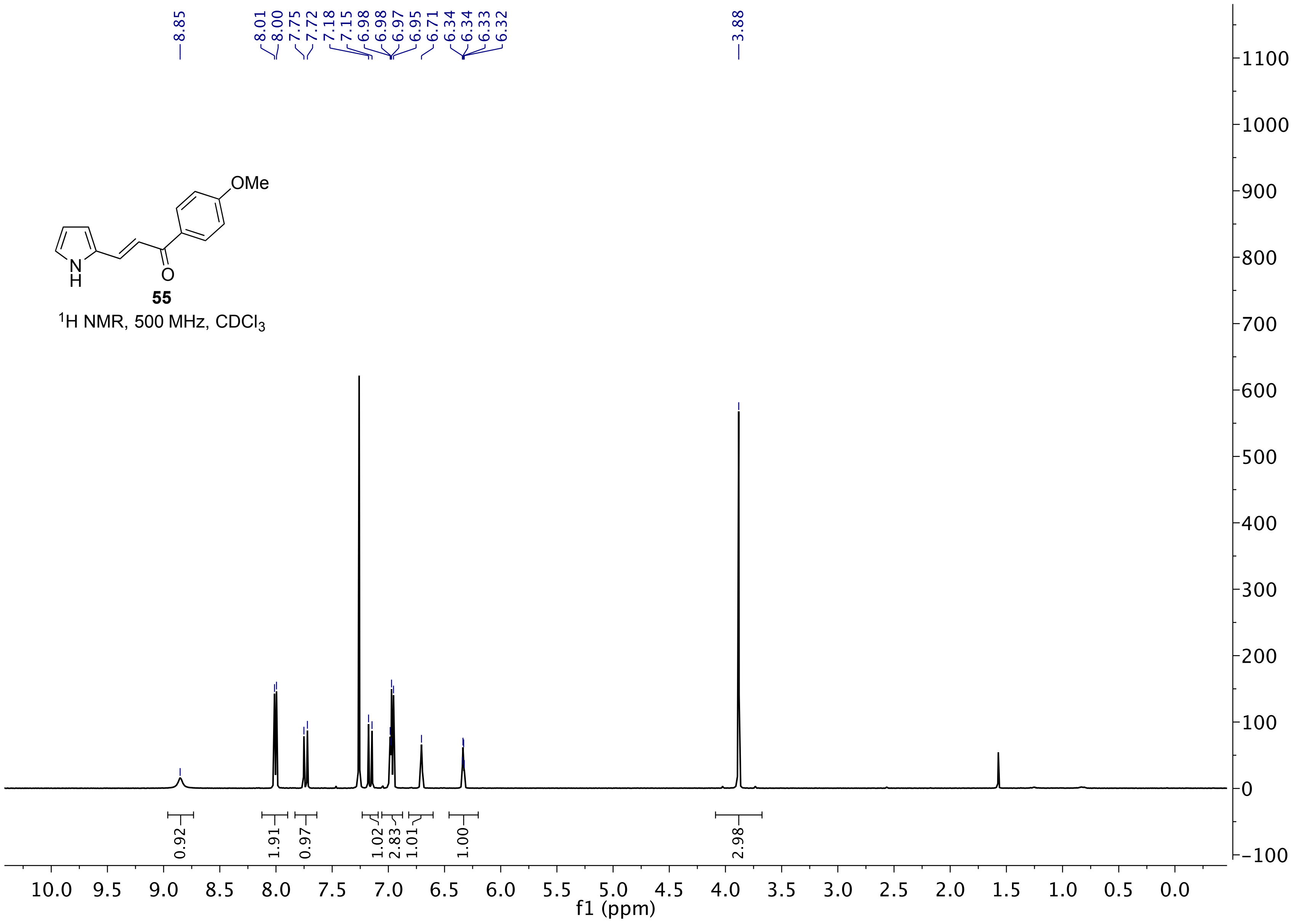
8.85
8.01
8.00
7.75
7.72
7.18
7.15
6.98
6.98
6.97
6.95
6.71
6.34
6.34
6.33
6.32
3.88
OMe
N
H
O
55
^{1}H NMR, 500 MHz, $CDCl_3$
0.92
1.91
0.97
1.02
2.83
1.01
1.00
2.98
10.0 9.5 9.0 8.5 8.0 7.5 7.0 6.5 6.0 5.5 5.0 4.5 4.0 3.5 3.0 2.5 2.0 1.5 1.0 0.5 0.0
f1 (ppm)
1100
1000
900
800
700
600
500
400
300
200
100
0
–100

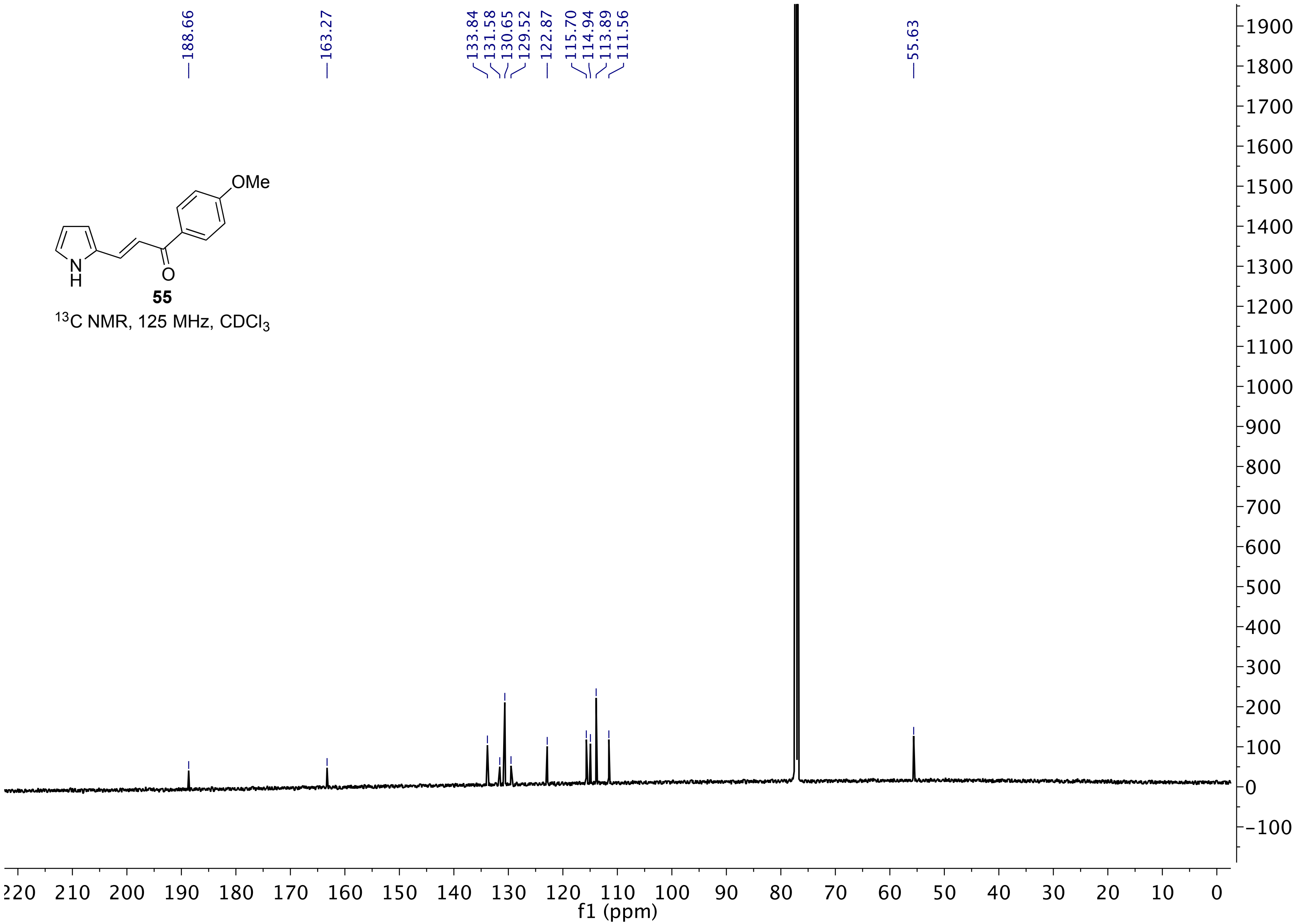
188.66
163.27
133.84
131.58
130.65
129.52
122.87
115.70
114.94
113.89
111.56
55.63
OMe
N
H
O
55
13C NMR, 125 MHz, CDCl3
f1 (ppm)

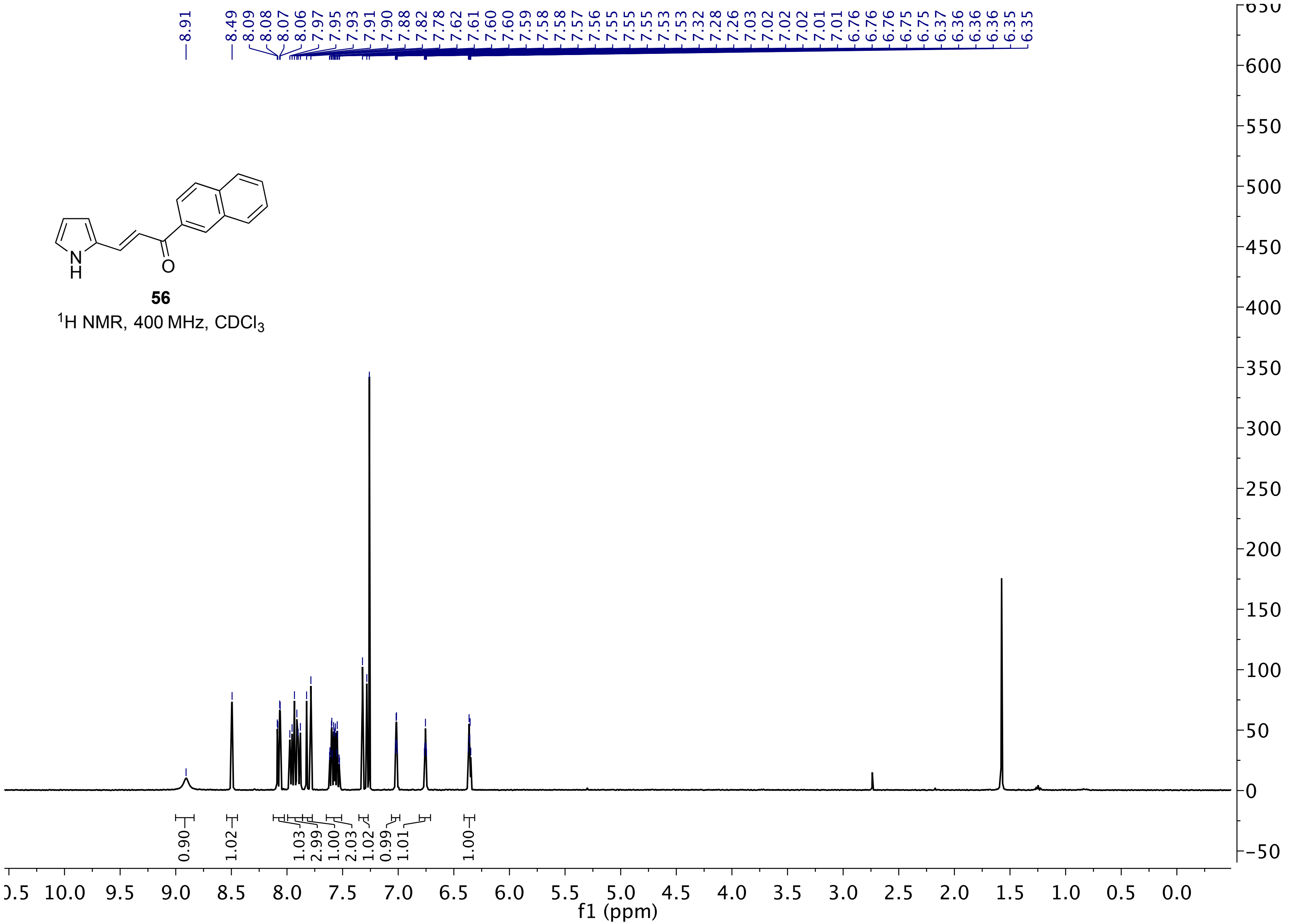
56
^1H NMR, 400 MHz, $CDCl_3$
f1 (ppm)

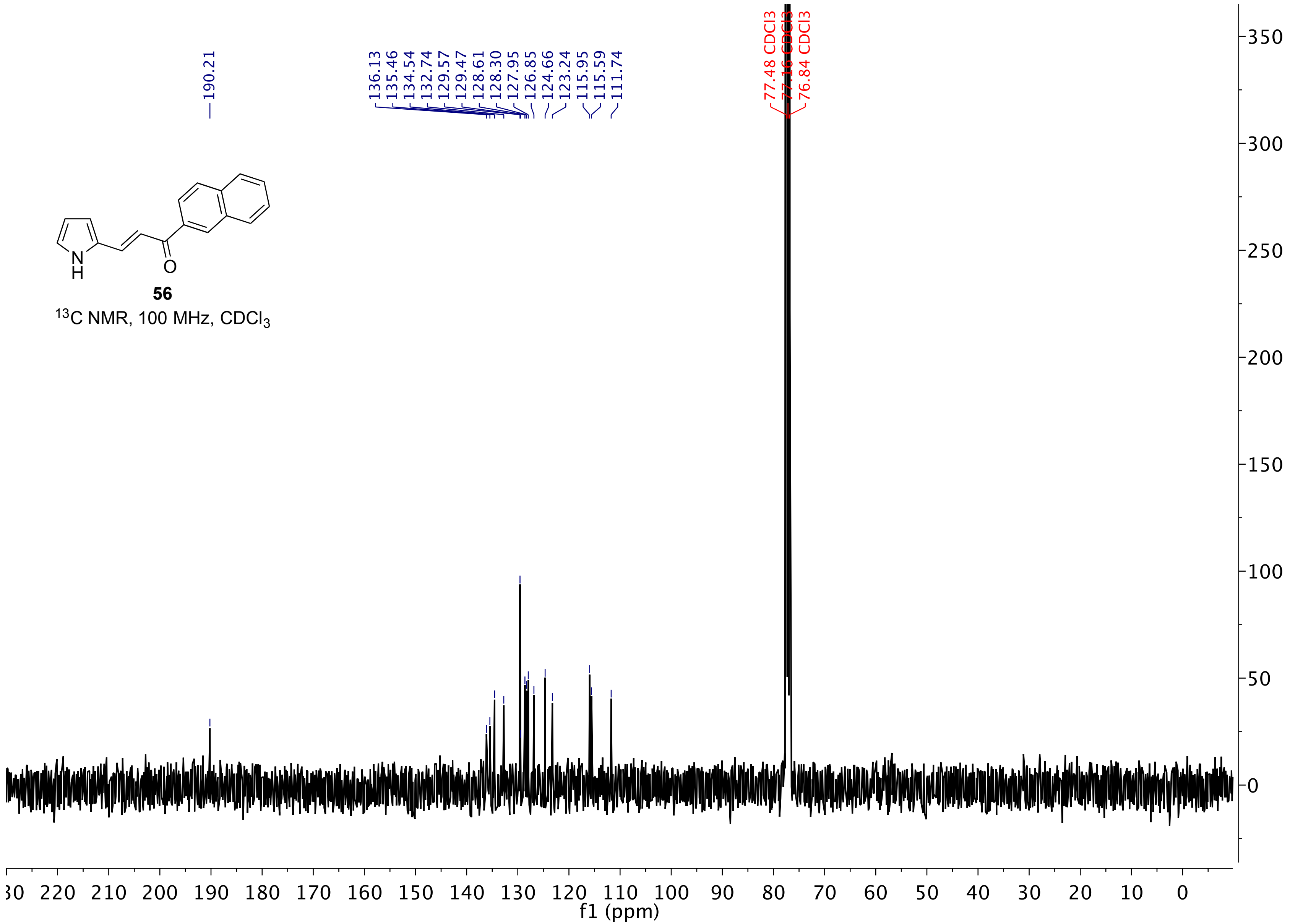

190.21
136.13
135.46
134.54
132.74
129.57
129.47
128.61
128.30
127.95
126.85
124.66
123.24
115.95
115.59
111.74
77.48 CDCl3
77.16 CDCl3
76.84 CDCl3
N
H
O
56
13C NMR, 100 MHz, CDCl3
f1 (ppm)

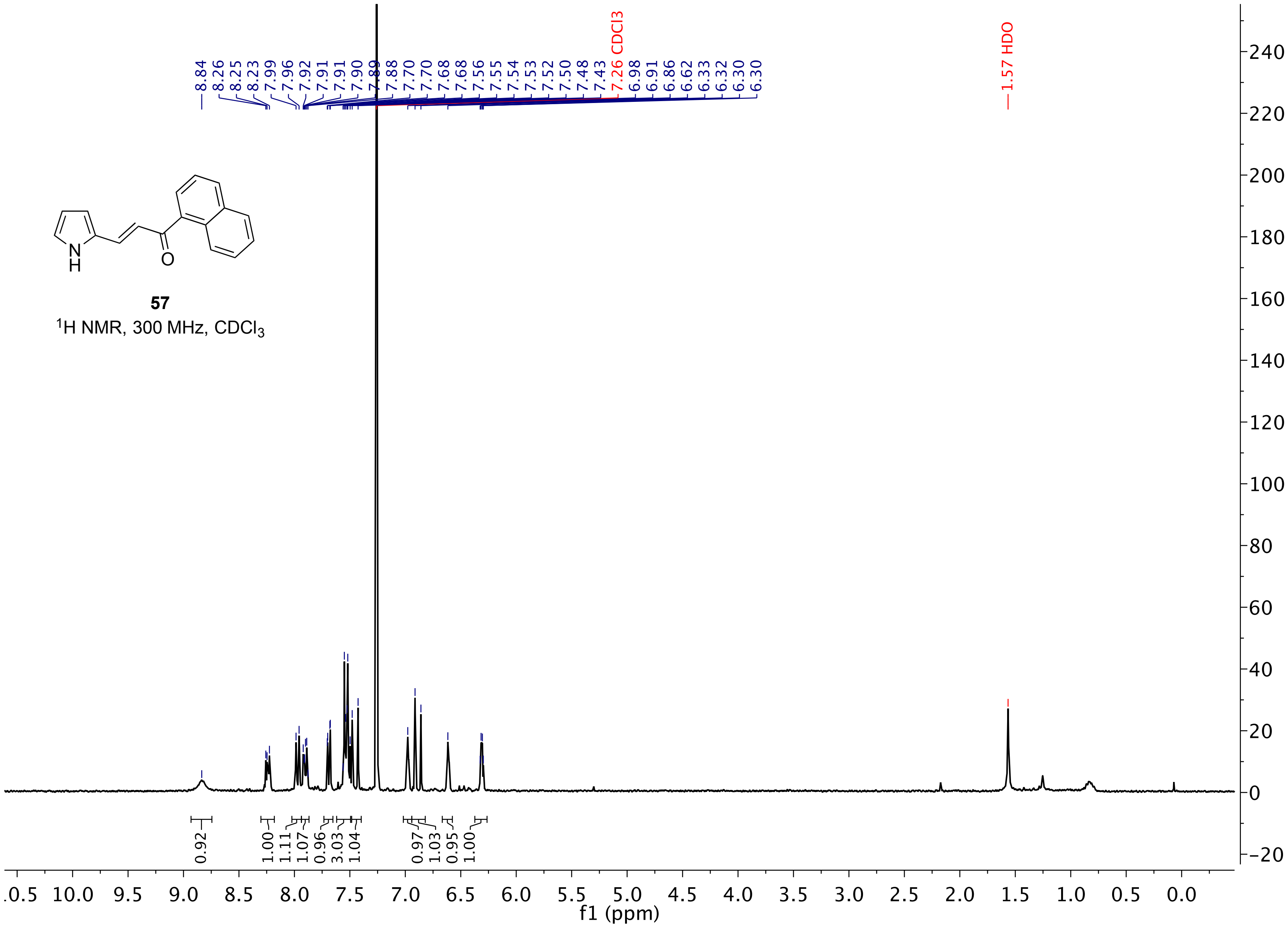
57
¹H NMR, 300 MHz, CDCl3
f1 (ppm)
7.26 CDCl3
1.57 HDO

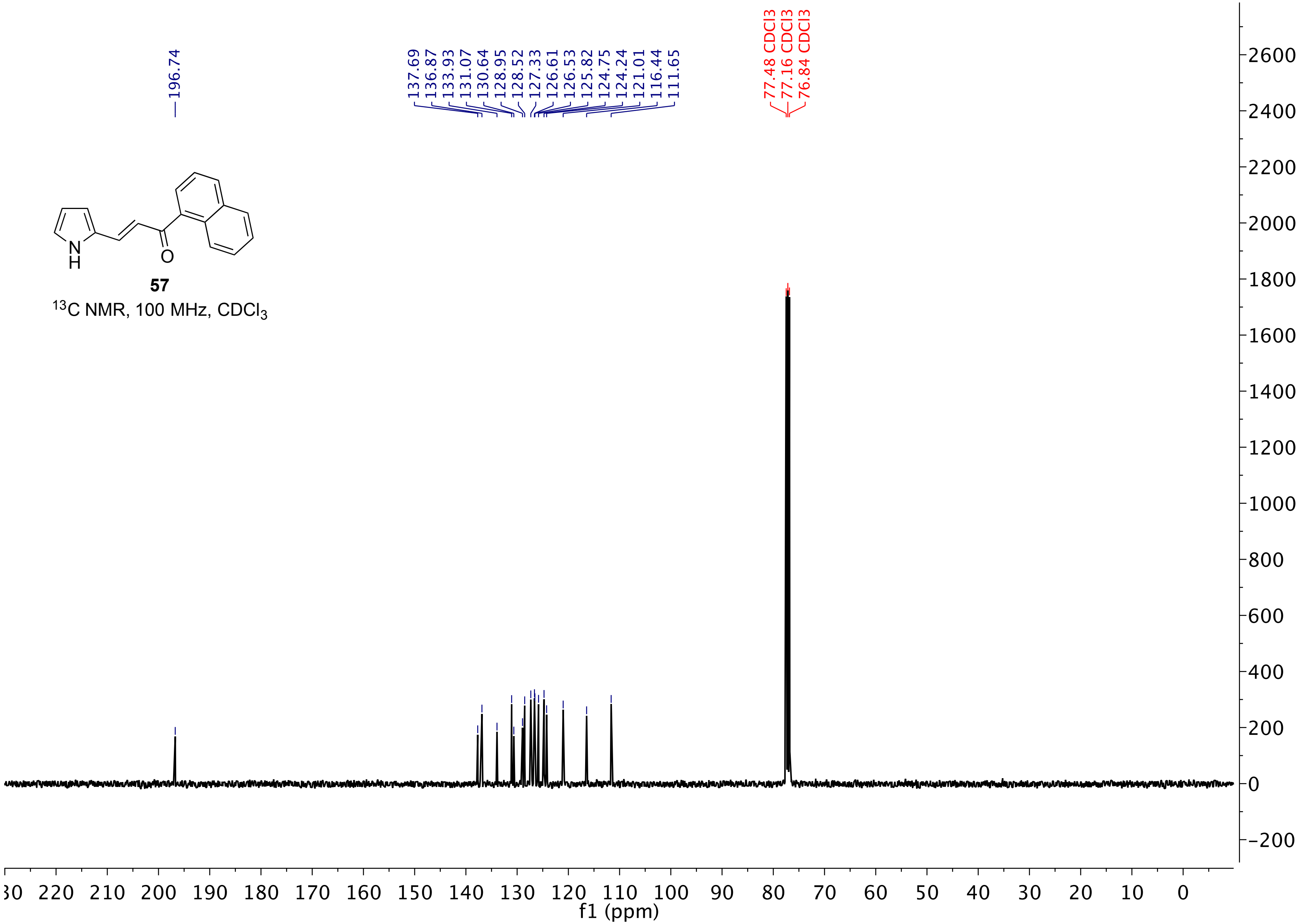

196.74
137.69
136.87
133.93
131.07
130.64
128.95
128.52
127.33
126.61
126.53
125.82
124.75
124.24
121.01
116.44
111.65
77.48 CDCl3
77.16 CDCl3
76.84 CDCl3
N
H
O
57
^{13}C NMR, 100 MHz, CDCl$_3$
f1 (ppm)

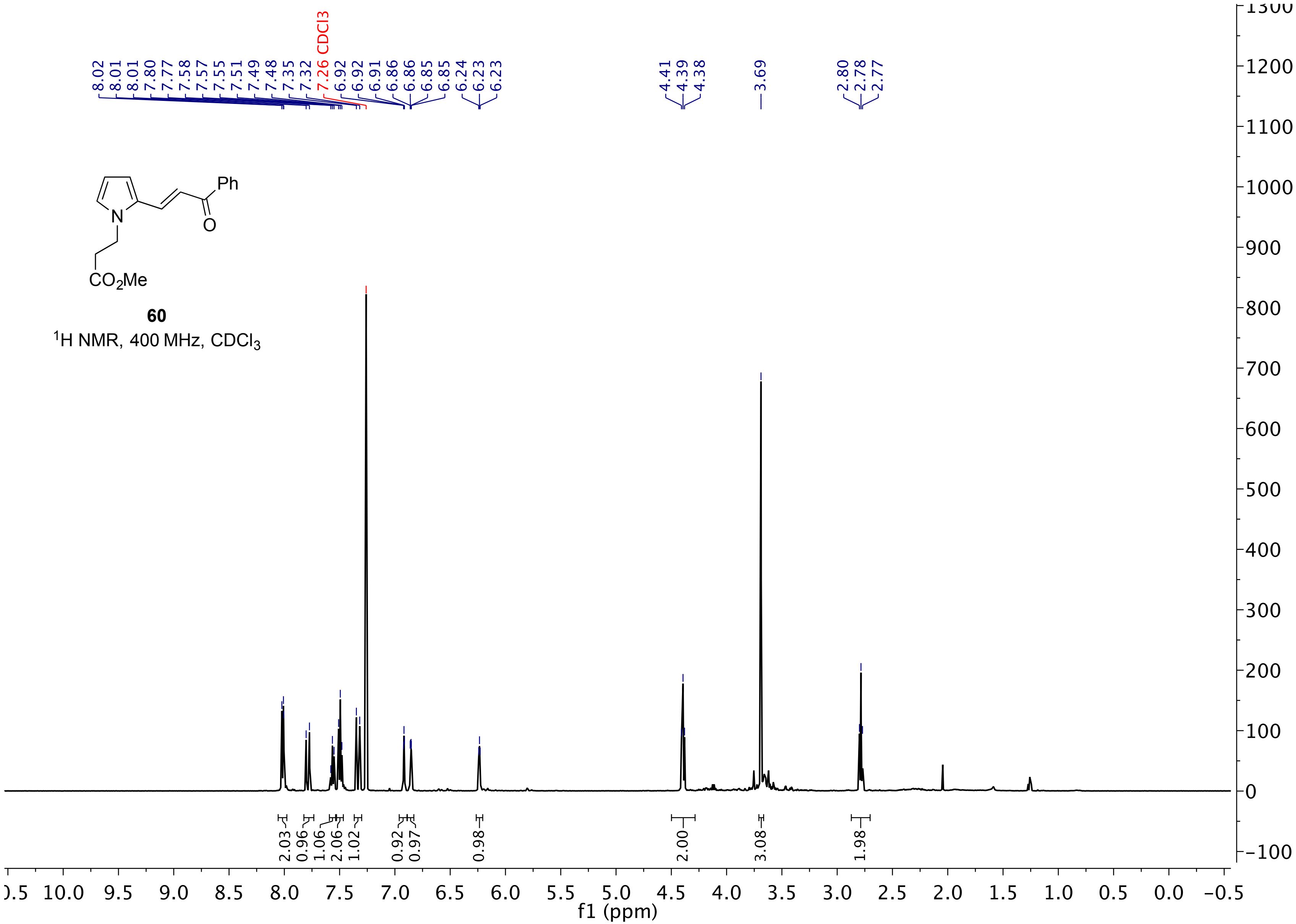

60

^1H NMR, 400 MHz, $CDCl_3$

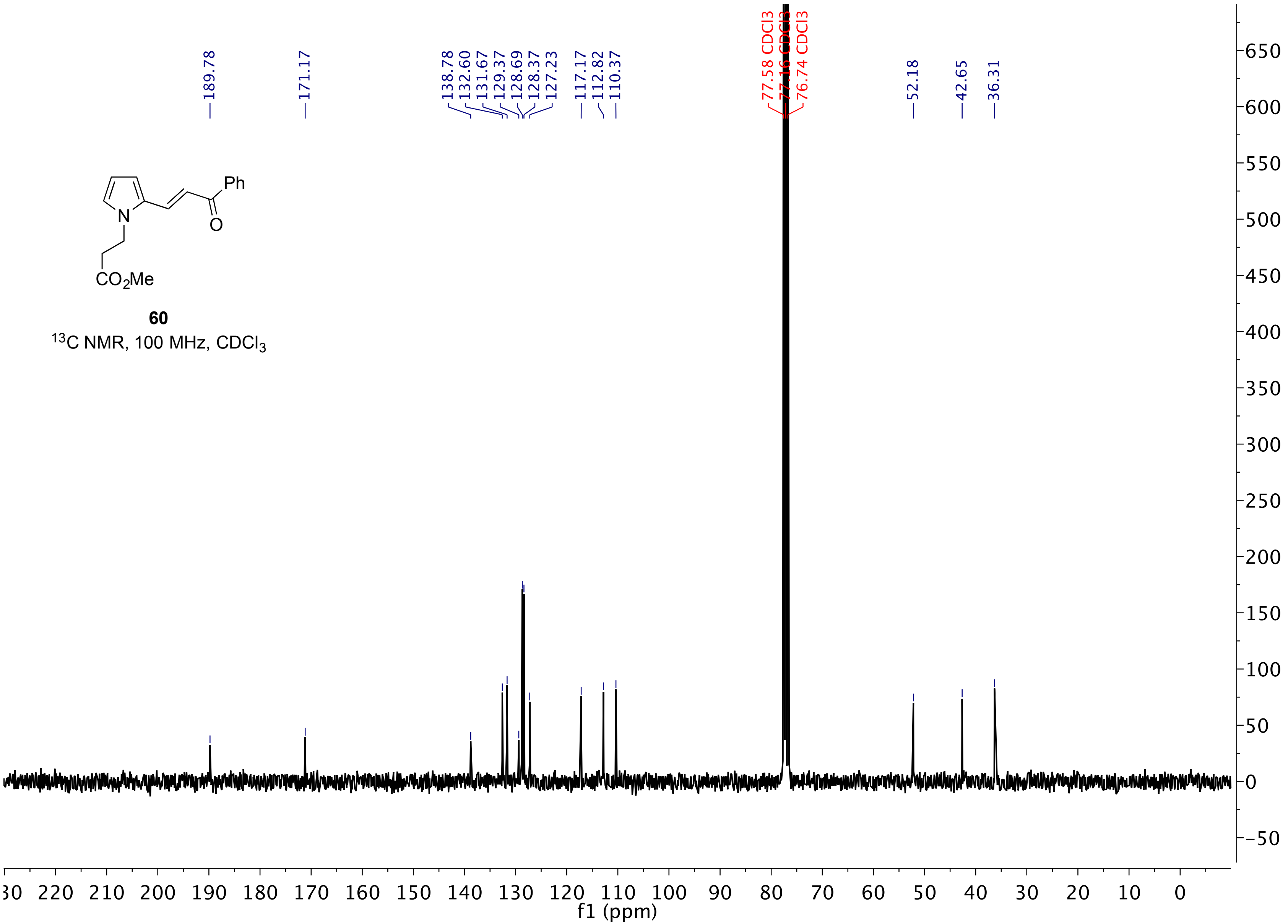

189.78
171.17
138.78
132.60
131.67
129.37
128.69
128.37
127.23
117.17
112.82
110.37
77.58 CDCl3
77.16 CDCl3
76.74 CDCl3
52.18
42.65
36.31
Ph
O
N
CO2Me
60
13C NMR, 100 MHz, CDCl3
f1 (ppm)

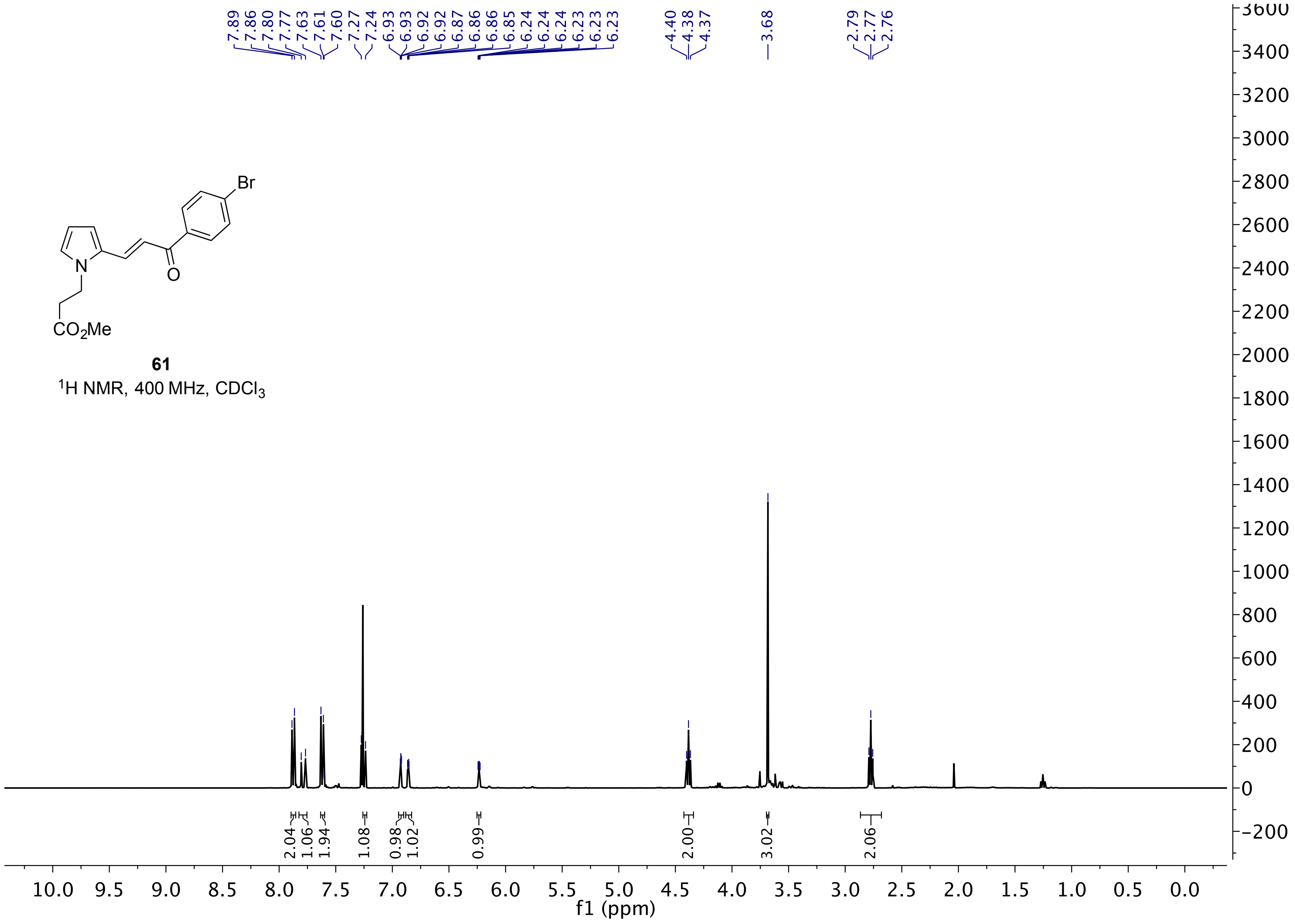
7.89 7.86 7.80 7.77 7.63 7.61 7.60 7.27 7.24 6.93 6.93 6.92 6.92 6.87 6.86 6.86 6.85 6.24 6.24 6.24 6.23 6.23 6.23
4.40 4.38 4.37
3.68
2.79 2.77 2.76
Br
N
O
CO2Me
61
1H NMR, 400 MHz, CDCl3
2.04 1.06 1.94 1.08 0.98 1.02 0.99 2.00 3.02 2.06
f1 (ppm)

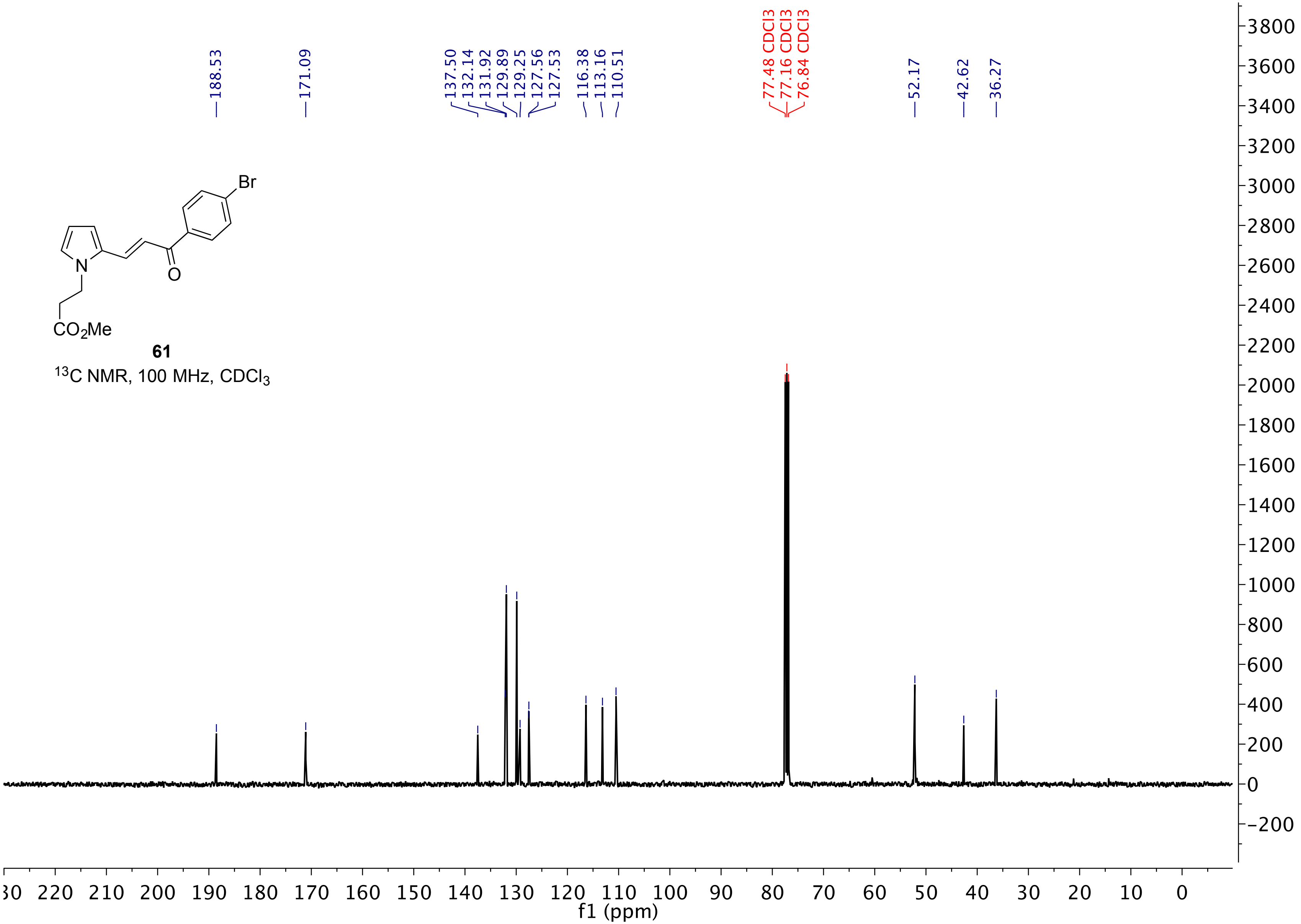

61

^{13}C NMR, 100 MHz, $CDCl_3$

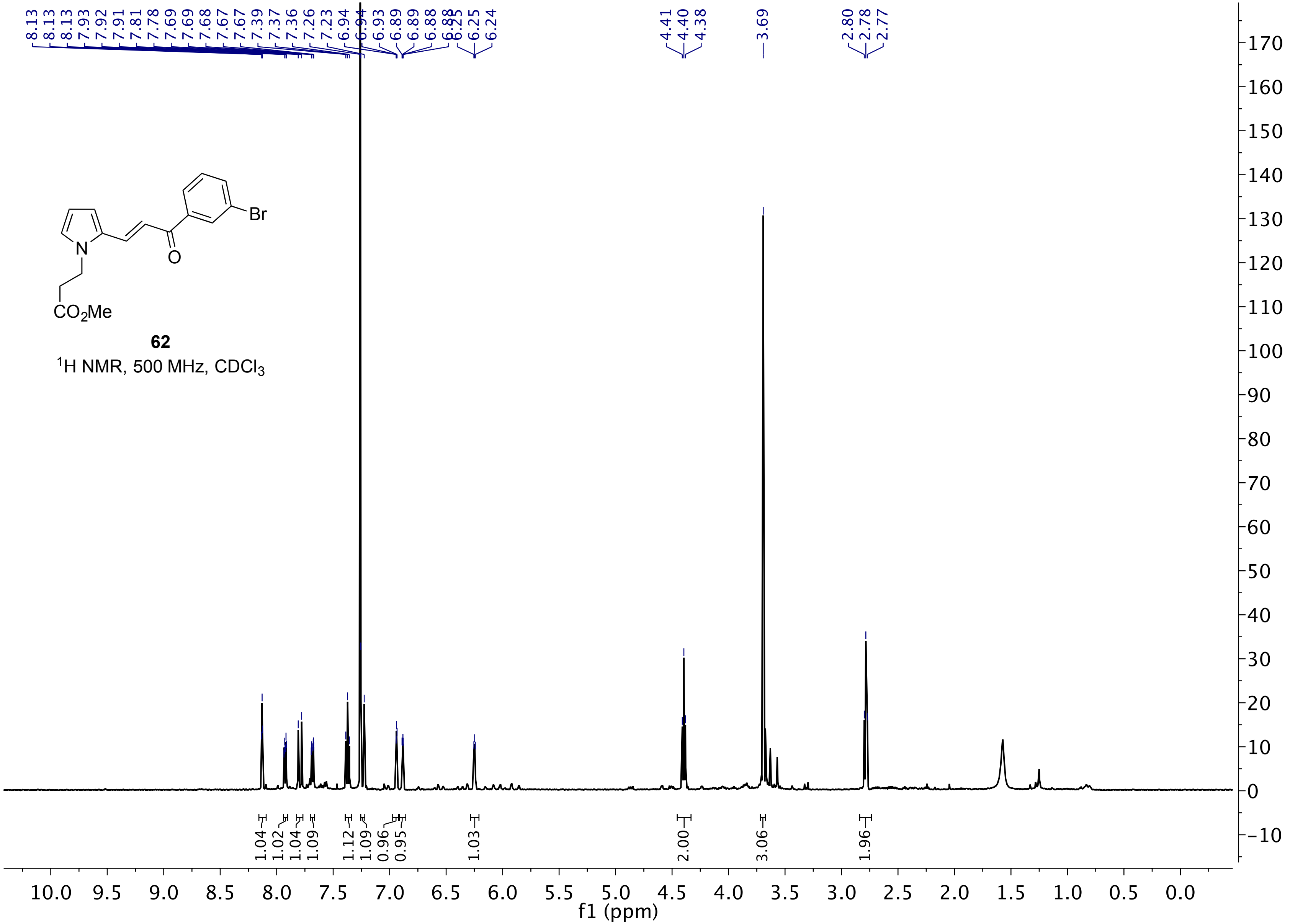

8.13 8.13 8.13 7.93 7.92 7.91 7.81 7.78 7.69 7.69 7.68 7.67 7.67 7.39 7.37 7.36 7.26 7.23 6.94 6.94 6.93 6.89 6.89 6.88 6.88 6.25 6.25 6.24
4.41 4.40 4.38
3.69
2.80 2.78 2.77
Br
N
O
CO_2Me
62
^1H NMR, 500 MHz, $CDCl_3$
1.04 1.02 1.04 1.09 1.12 1.09 0.96 0.95 1.03 2.00 3.06 1.96
f1 (ppm)

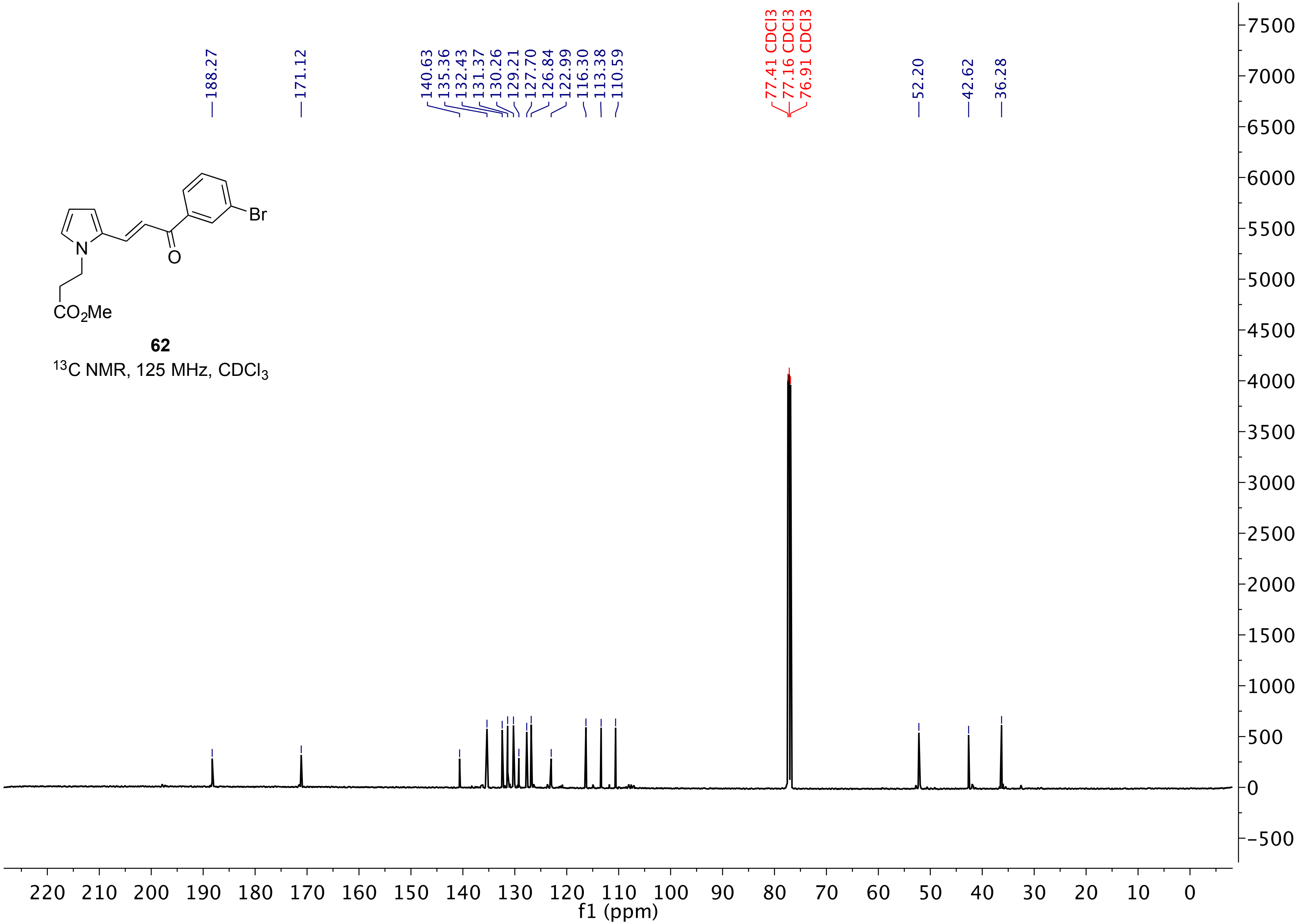
188.27
171.12
140.63
135.36
132.43
131.37
130.26
129.21
127.70
126.84
122.99
116.30
113.38
110.59
77.41 CDCl3
77.16 CDCl3
76.91 CDCl3
52.20
42.62
36.28
Br
N
O
CO_2Me
62
^{13}C NMR, 125 MHz, $CDCl_3$
f1 (ppm)

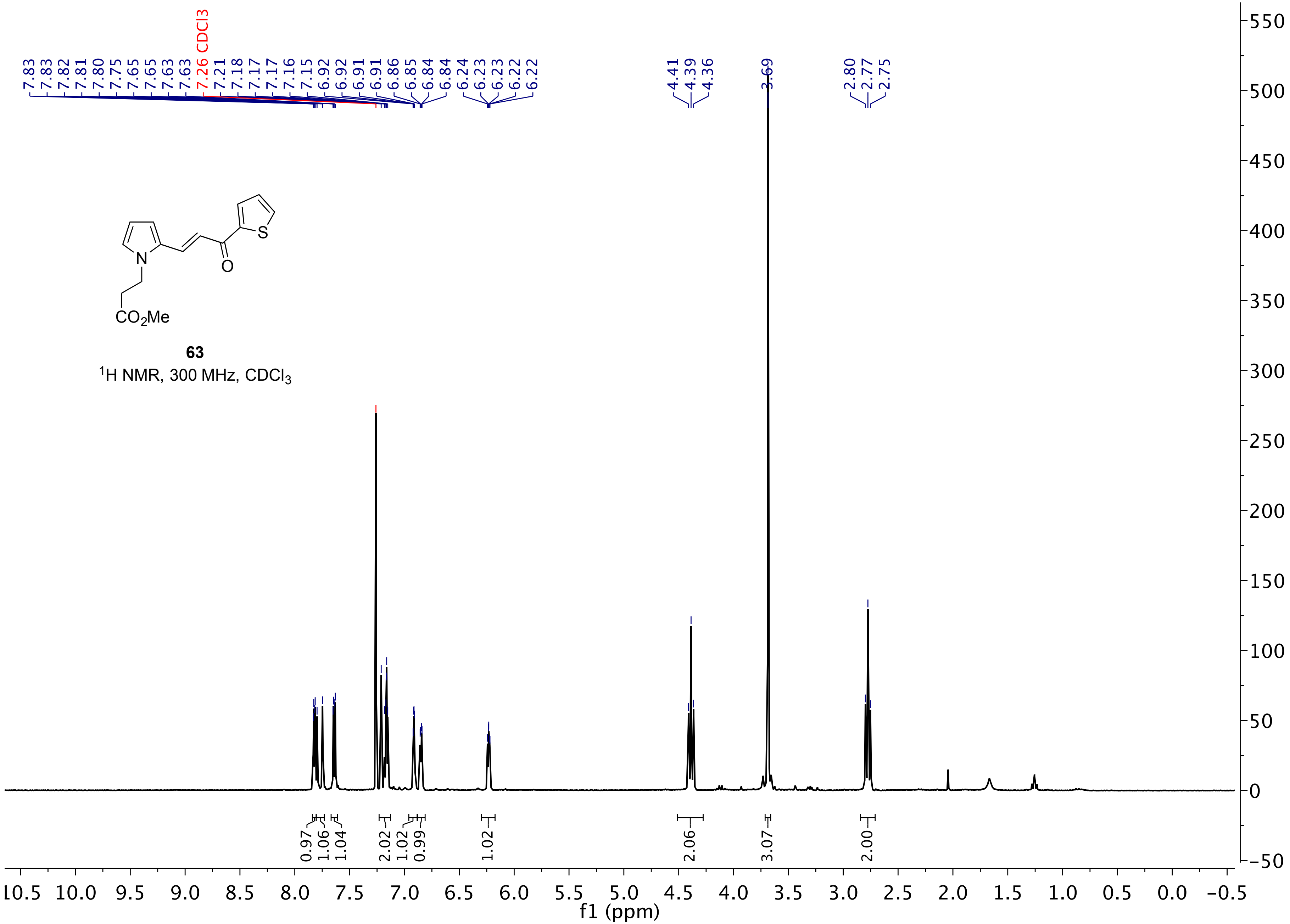
7.83 7.83 7.82 7.81 7.80 7.75 7.65 7.65 7.63 7.63 7.26 CDCl3 7.21 7.18 7.17 7.17 7.16 7.15 6.92 6.92 6.91 6.91 6.86 6.85 6.84 6.84 6.24 6.23 6.23 6.22 6.22
4.41 4.39 4.36
3.69
2.80 2.77 2.75
N
S
O
CO2Me
63
1H NMR, 300 MHz, CDCl3
0.97 1.06 1.04 2.02 1.02 0.99 1.02 2.06 3.07 2.00
f1 (ppm)

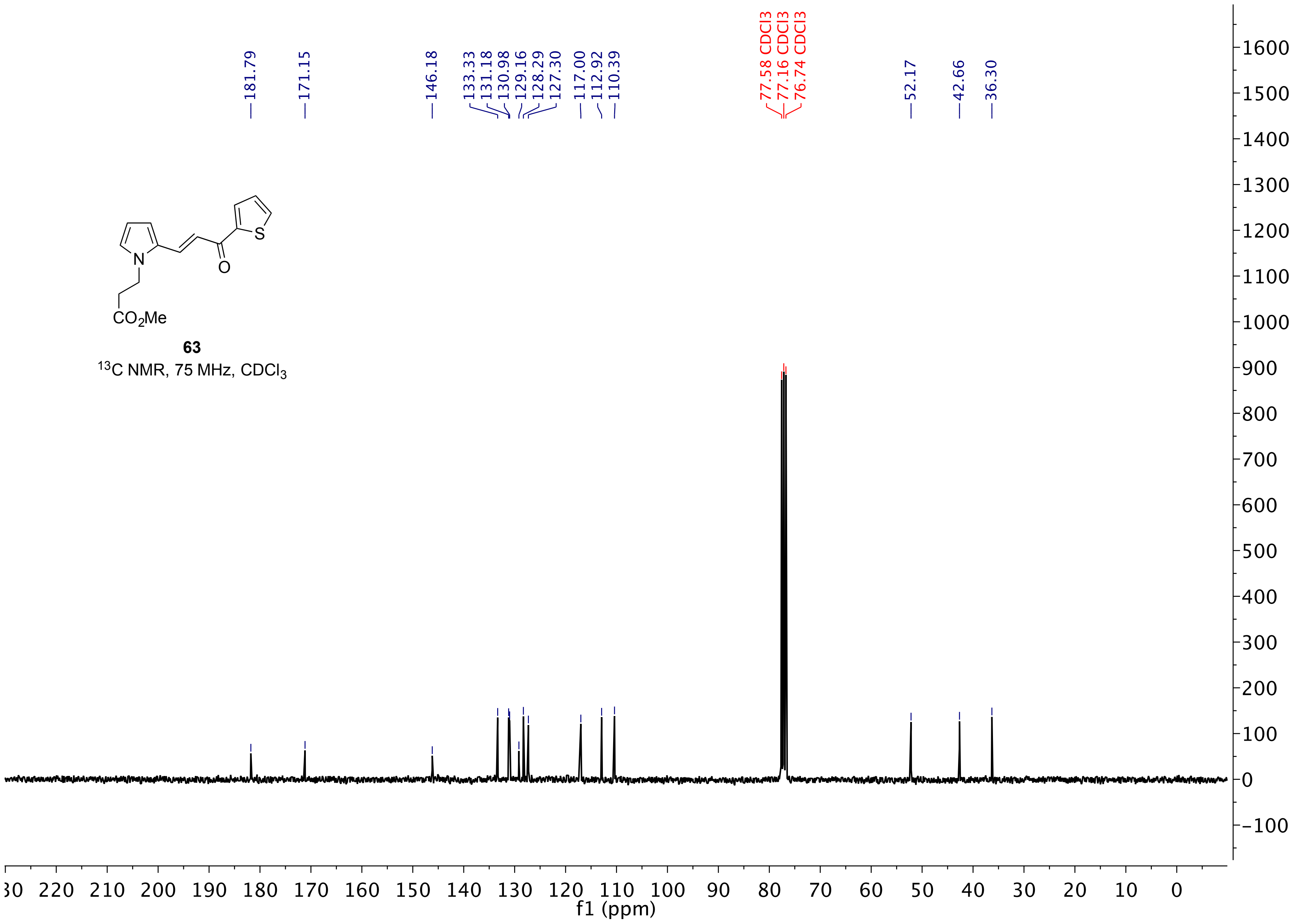

181.79
171.15
146.18
133.33
131.18
130.98
129.16
128.29
127.30
117.00
112.92
110.39
77.58 CDCl3
77.16 CDCl3
76.74 CDCl3
52.17
42.66
36.30
N
S
O
CO2Me
63
13C NMR, 75 MHz, CDCl3
f1 (ppm)

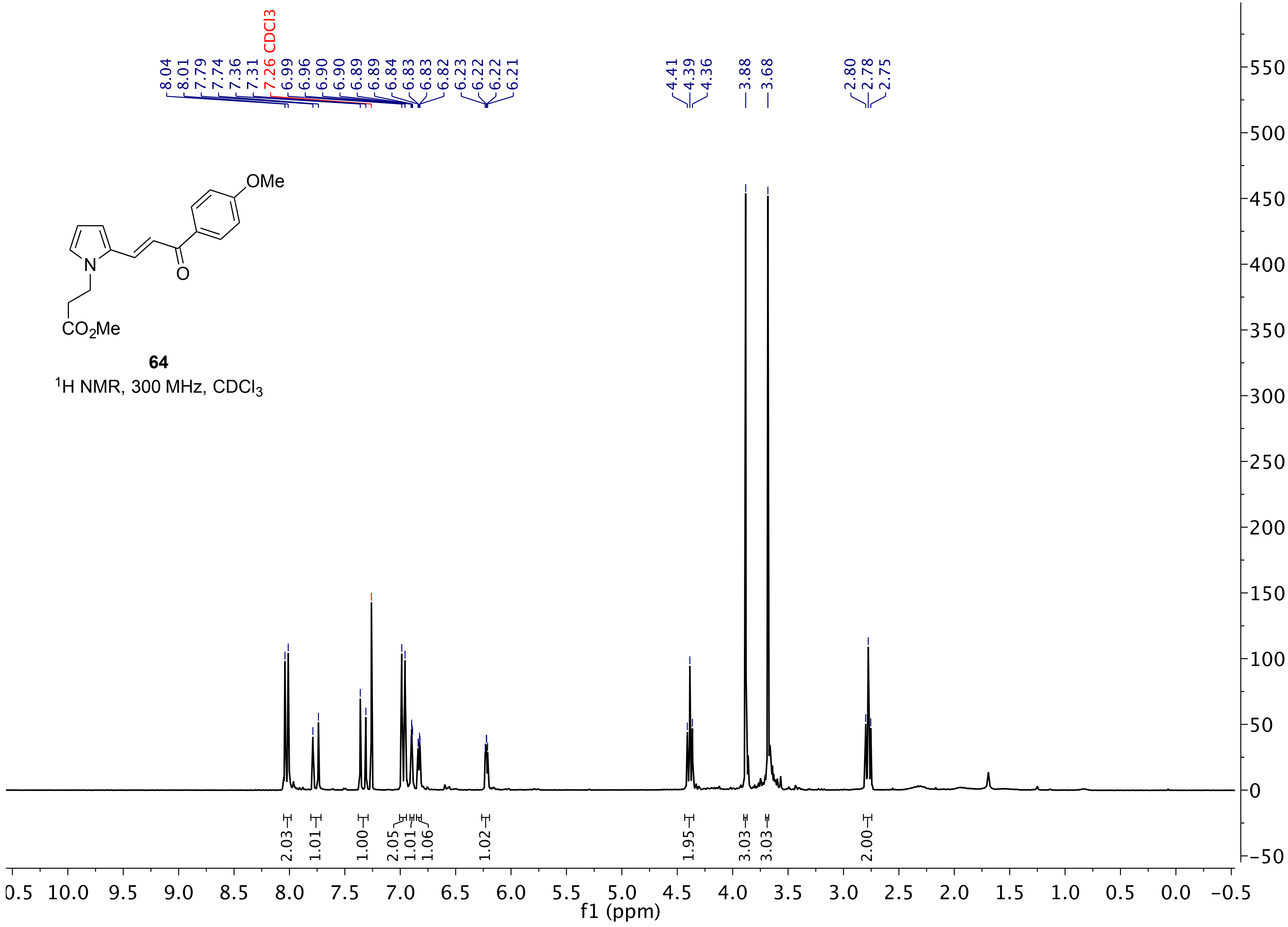
OMe
N
O
CO2Me
64
1H NMR, 300 MHz, CDCl3
8.04 8.01 7.79 7.74 7.36 7.31 7.26 CDCl3 6.99 6.96 6.90 6.90 6.89 6.89 6.84 6.83 6.83 6.82 6.23 6.22 6.22 6.21
4.41 4.39 4.36 3.88 3.68 2.80 2.78 2.75
2.03 1.01 1.00 2.05 1.01 1.06 1.02 1.95 3.03 3.03 2.00
f1 (ppm)

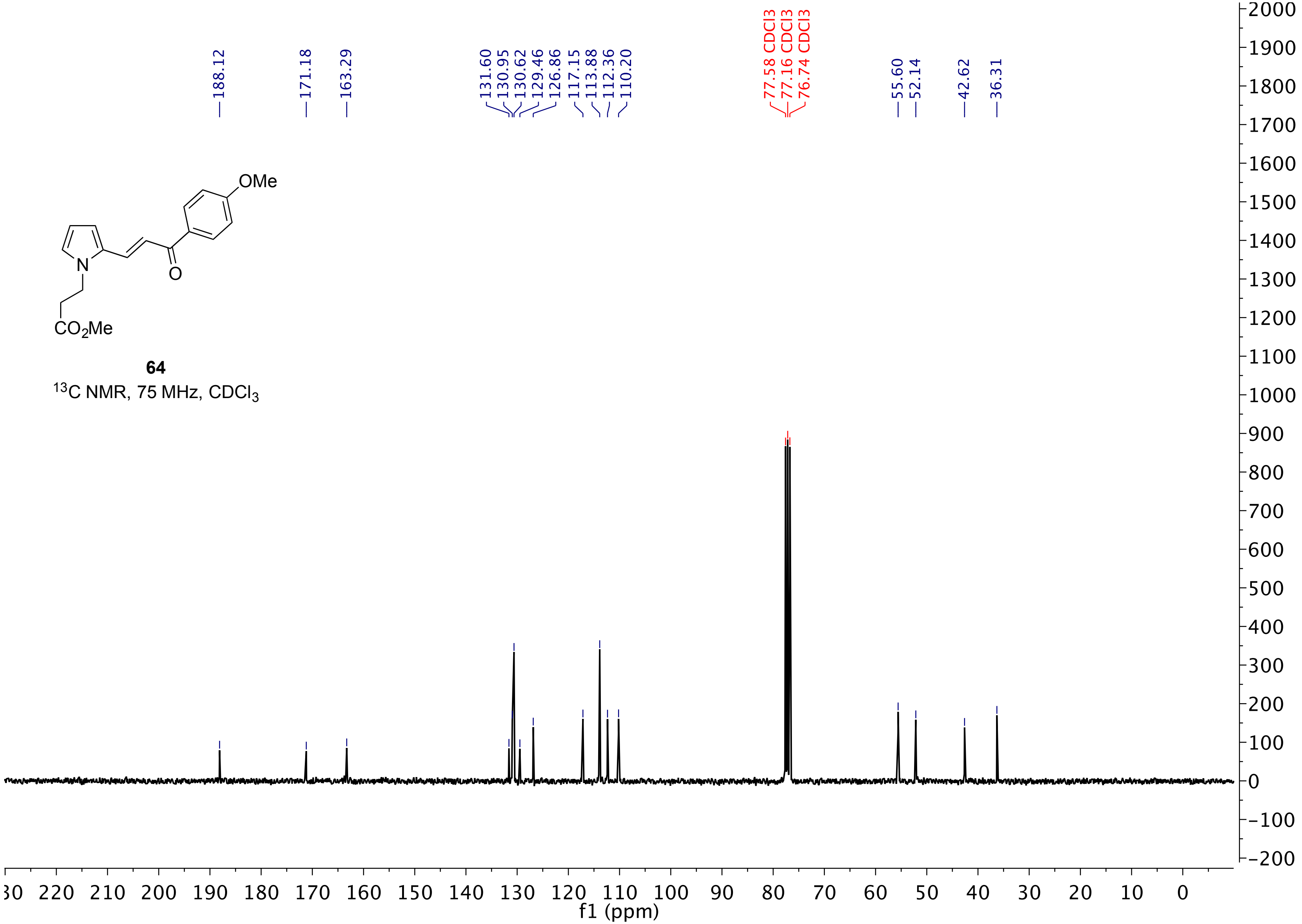
188.12
171.18
163.29
131.60
130.95
130.62
129.46
126.86
117.15
113.88
112.36
110.20
77.58 CDCl3
77.16 CDCl3
76.74 CDCl3
55.60
52.14
42.62
36.31
OMe
N
O
CO2Me
64
13C NMR, 75 MHz, CDCl3
f1 (ppm)

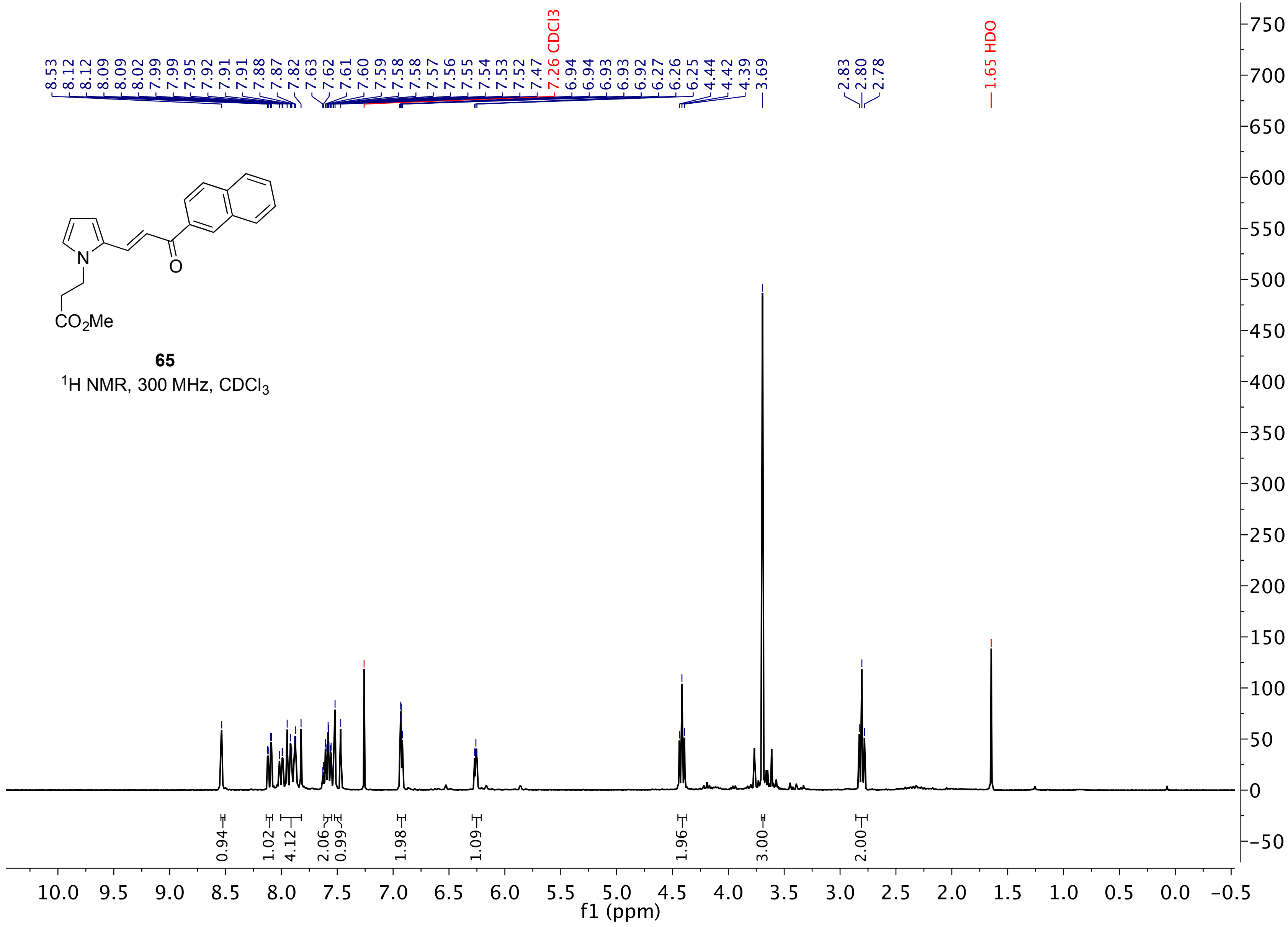

8.53
8.12
8.12
8.09
8.09
8.02
7.99
7.99
7.95
7.92
7.91
7.91
7.88
7.87
7.82
7.63
7.62
7.61
7.60
7.59
7.58
7.58
7.57
7.56
7.55
7.54
7.53
7.52
7.47
7.26 CDCl3
6.94
6.94
6.93
6.93
6.92
6.27
6.26
6.25
4.44
4.42
4.39
3.69
2.83
2.80
2.78
1.65 HDO
N
O
CO2Me
65
1H NMR, 300 MHz, CDCl3
0.94
1.02
4.12
2.06
0.99
1.98
1.09
1.96
3.00
2.00
f1 (ppm)

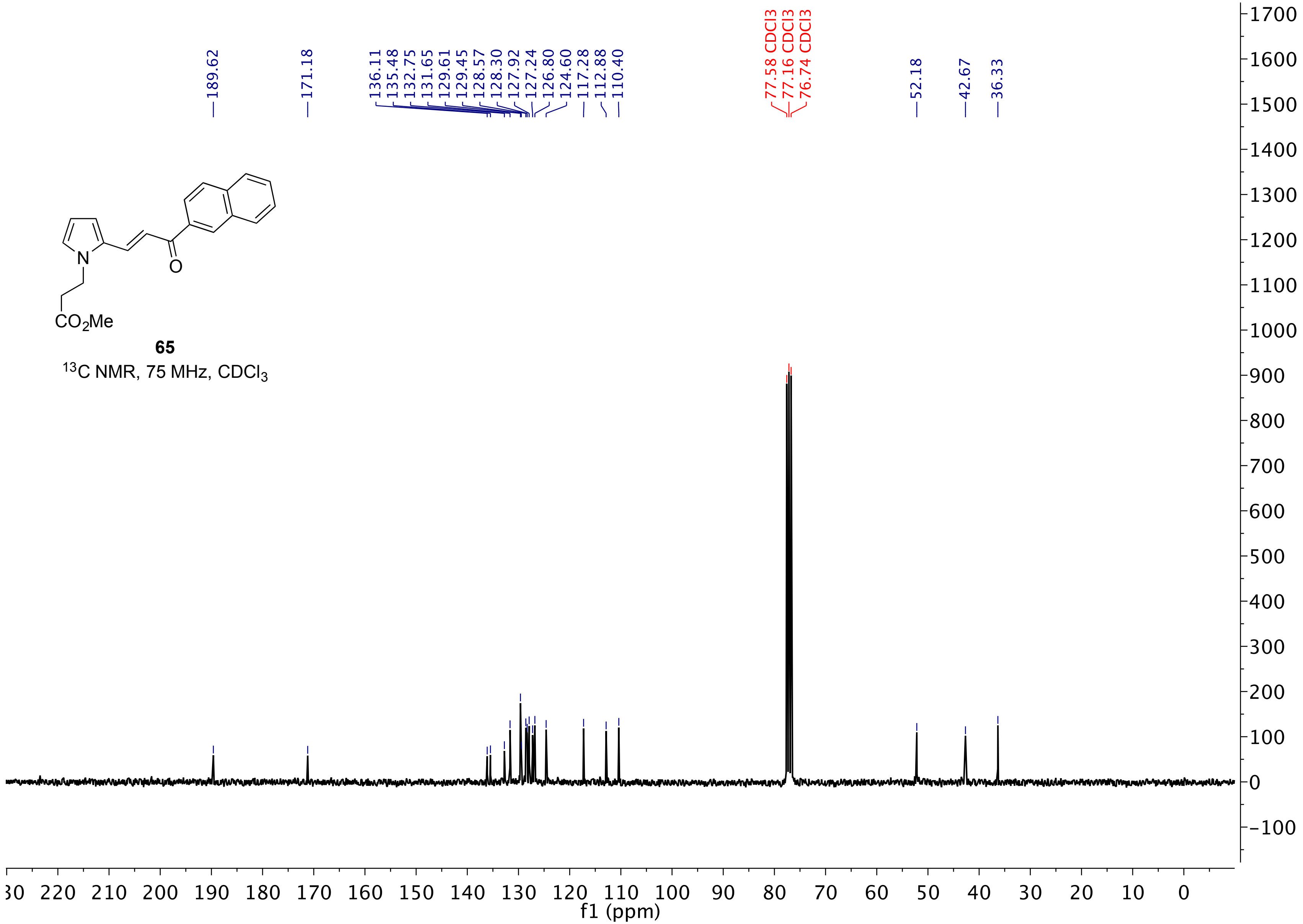
189.62
171.18
136.11
135.48
132.75
131.65
129.61
129.45
128.57
128.30
127.92
127.24
126.80
124.60
117.28
112.88
110.40
77.58 CDCl3
77.16 CDCl3
76.74 CDCl3
52.18
42.67
36.33
O
N
CO2Me
65
13C NMR, 75 MHz, CDCl3
f1 (ppm)

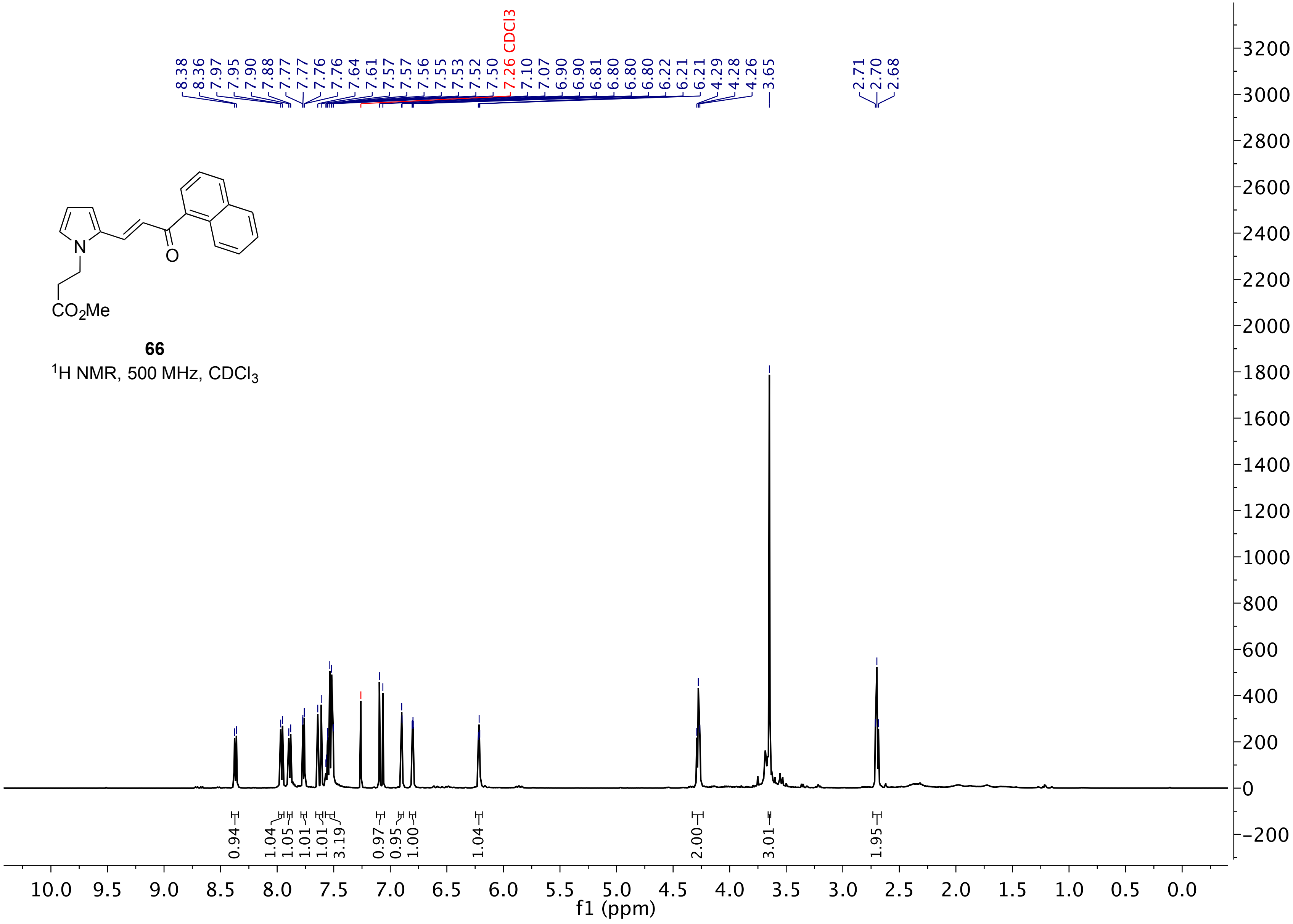

66

^1H NMR, 500 MHz, $CDCl_3$

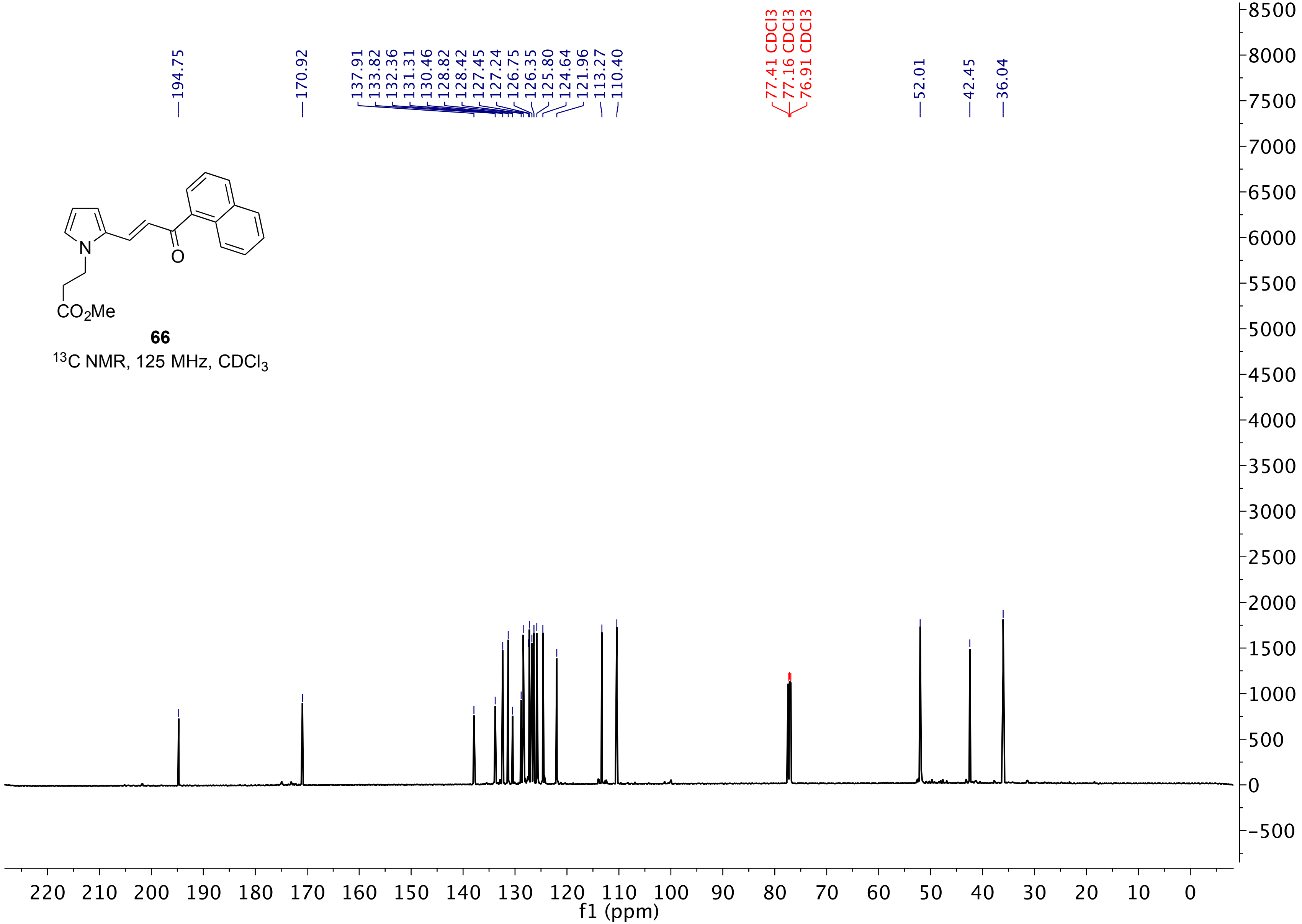
194.75
170.92
137.91
133.82
132.36
131.31
130.46
128.82
128.42
127.45
127.24
126.75
126.35
125.80
124.64
121.96
113.27
110.40
77.41 CDCl3
77.16 CDCl3
76.91 CDCl3
52.01
42.45
36.04
N
O
CO2Me
66
13C NMR, 125 MHz, CDCl3
f1 (ppm)

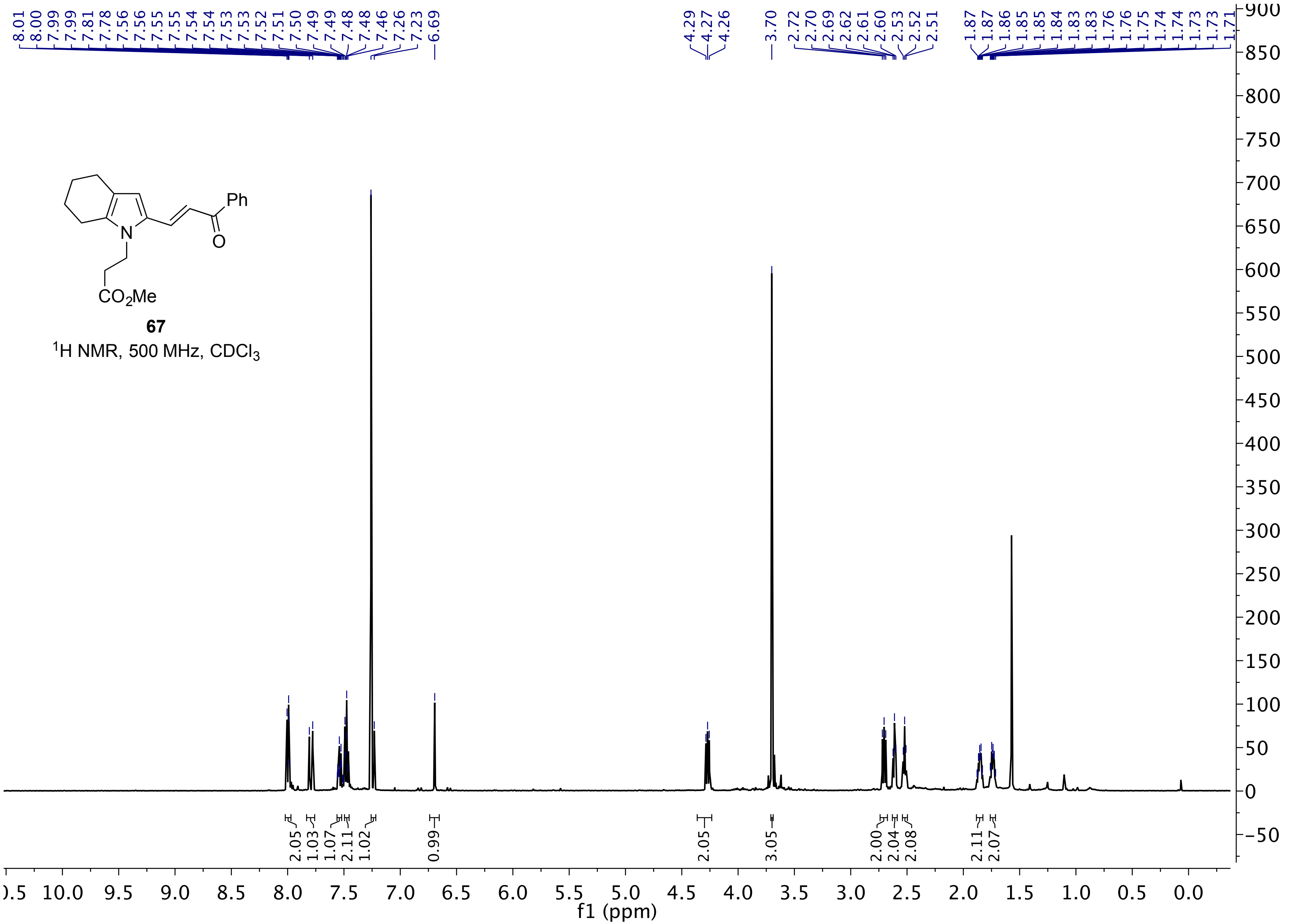
8.01 8.00 7.99 7.81 7.78 7.56 7.56 7.55 7.54 7.54 7.53 7.53 7.52 7.51 7.50 7.49 7.49 7.48 7.48 7.46 7.26 7.23 6.69
4.29 4.27 4.26
3.70
2.72 2.70 2.69 2.62 2.61 2.60 2.53 2.52 2.51
1.87 1.87 1.86 1.85 1.85 1.84 1.83 1.83 1.76 1.76 1.75 1.74 1.74 1.73 1.73 1.71
Ph
N
O
CO2Me
67
1H NMR, 500 MHz, CDCl3
2.05 1.03 1.07 2.11 1.02 0.99 2.05 3.05 2.00 2.04 2.08 2.11 2.07
f1 (ppm)

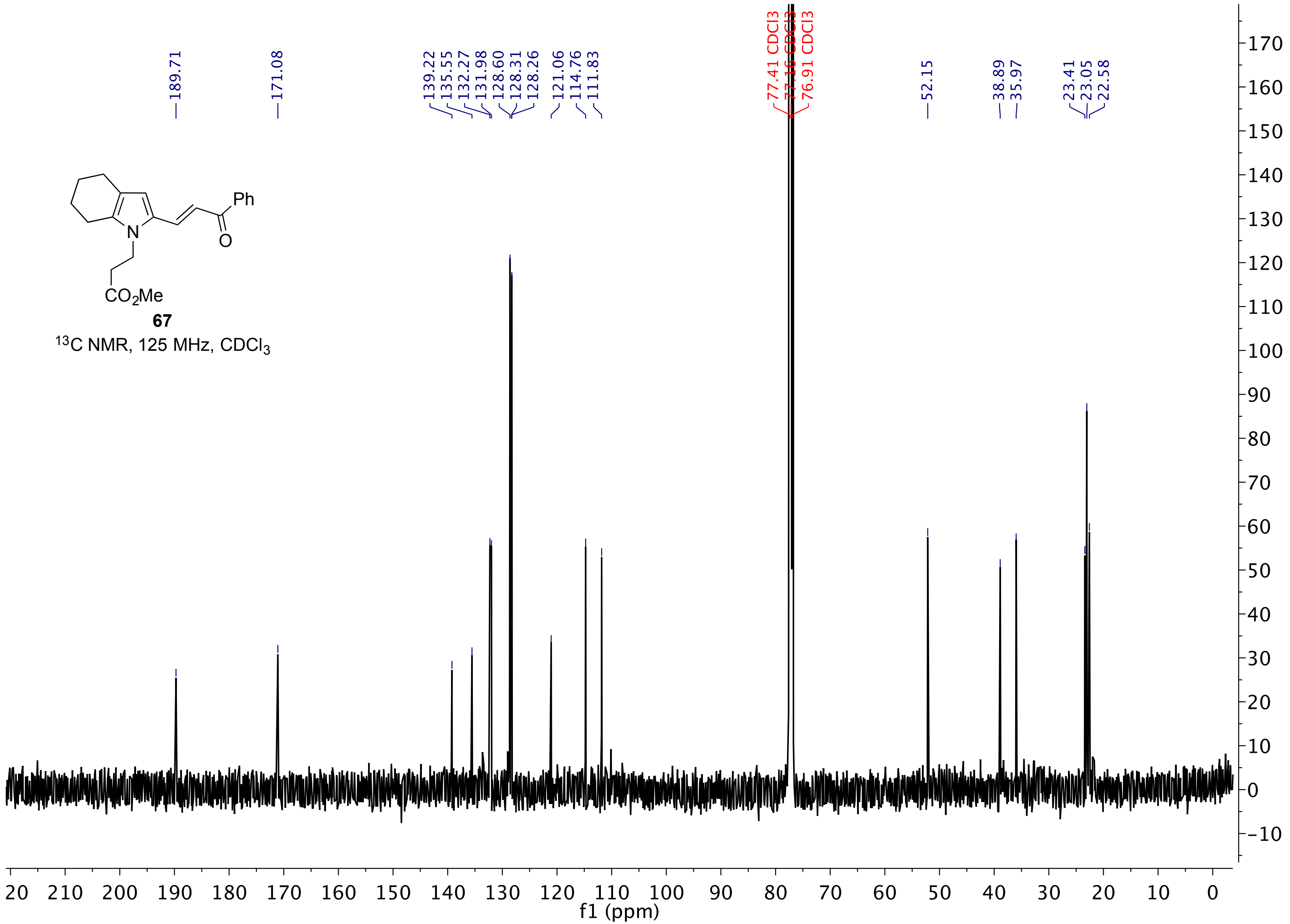

189.71
171.08
139.22
135.55
132.27
131.98
128.60
128.31
128.26
121.06
114.76
111.83
77.41 CDCl3
77.16 CDCl3
76.91 CDCl3
52.15
38.89
35.97
23.41
23.05
22.58
Ph
O
N
CO2Me
67
13C NMR, 125 MHz, CDCl3
f1 (ppm)

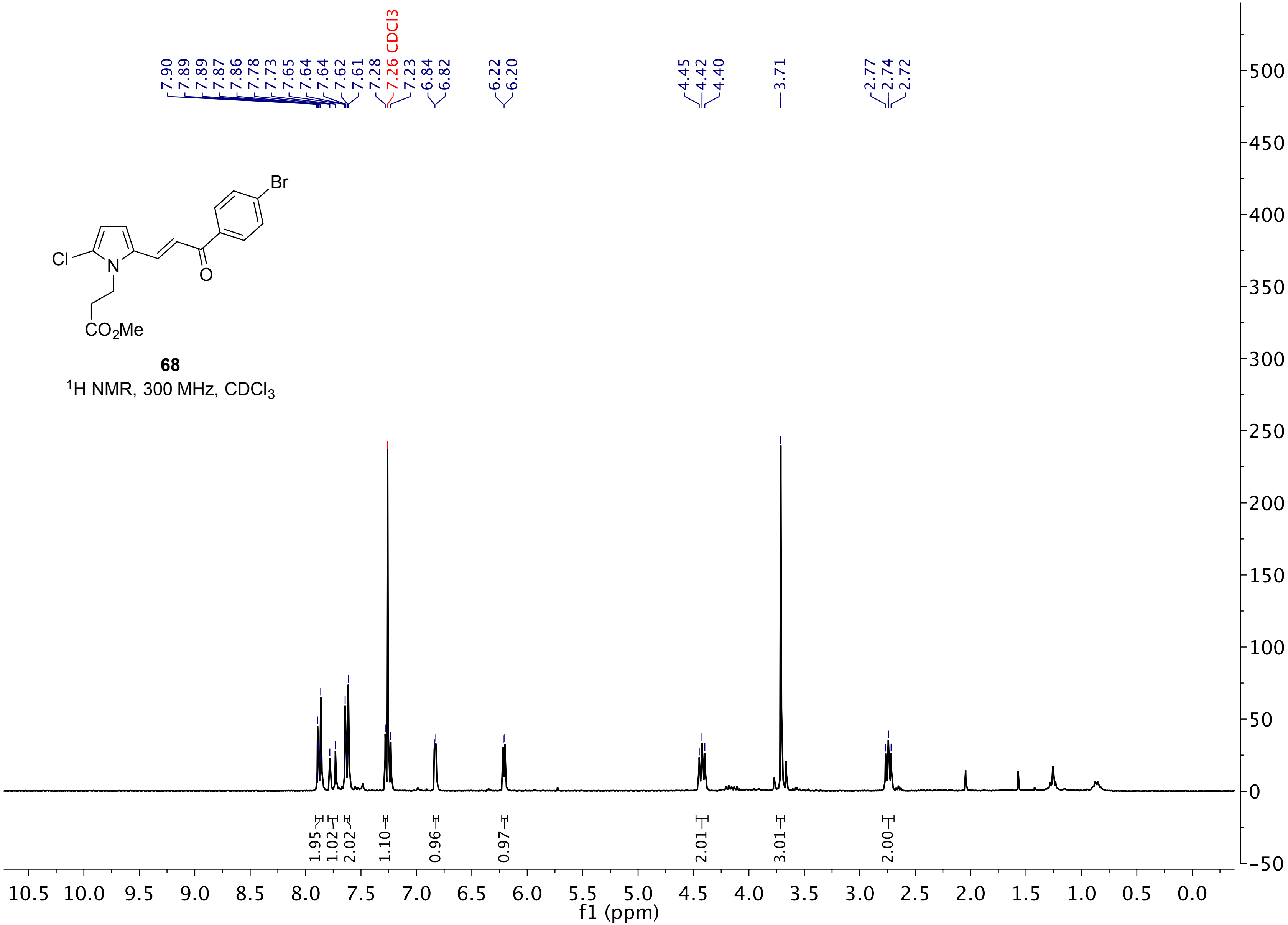
7.90 7.89 7.89 7.87 7.86 7.78 7.73 7.65 7.64 7.64 7.62 7.61 7.28 7.26 CDCl3 7.23 6.84 6.82 6.22 6.20 4.45 4.42 4.40 3.71 2.77 2.74 2.72
Br
Cl
N
O
CO_2Me
68
^1H NMR, 300 MHz, $CDCl_3$
1.95 1.02 2.02 1.10 0.96 0.97 2.01 3.01 2.00
f1 (ppm)

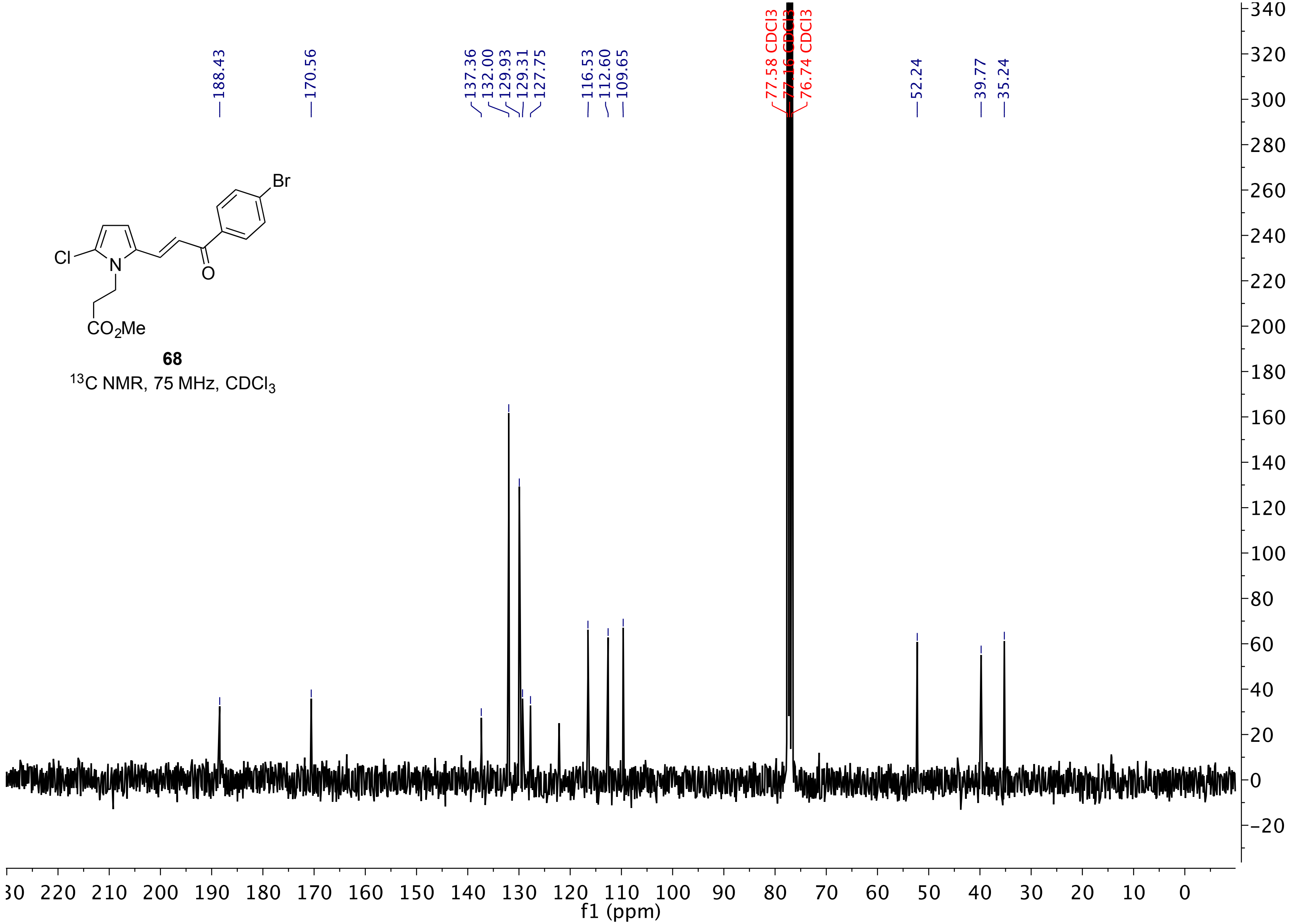
188.43
170.56
137.36
132.00
129.93
129.31
127.75
116.53
112.60
109.65
77.58 CDCl3
77.16 CDCl3
76.74 CDCl3
52.24
39.77
35.24
Br
Cl
N
O
CO2Me
68
13C NMR, 75 MHz, CDCl3
f1 (ppm)

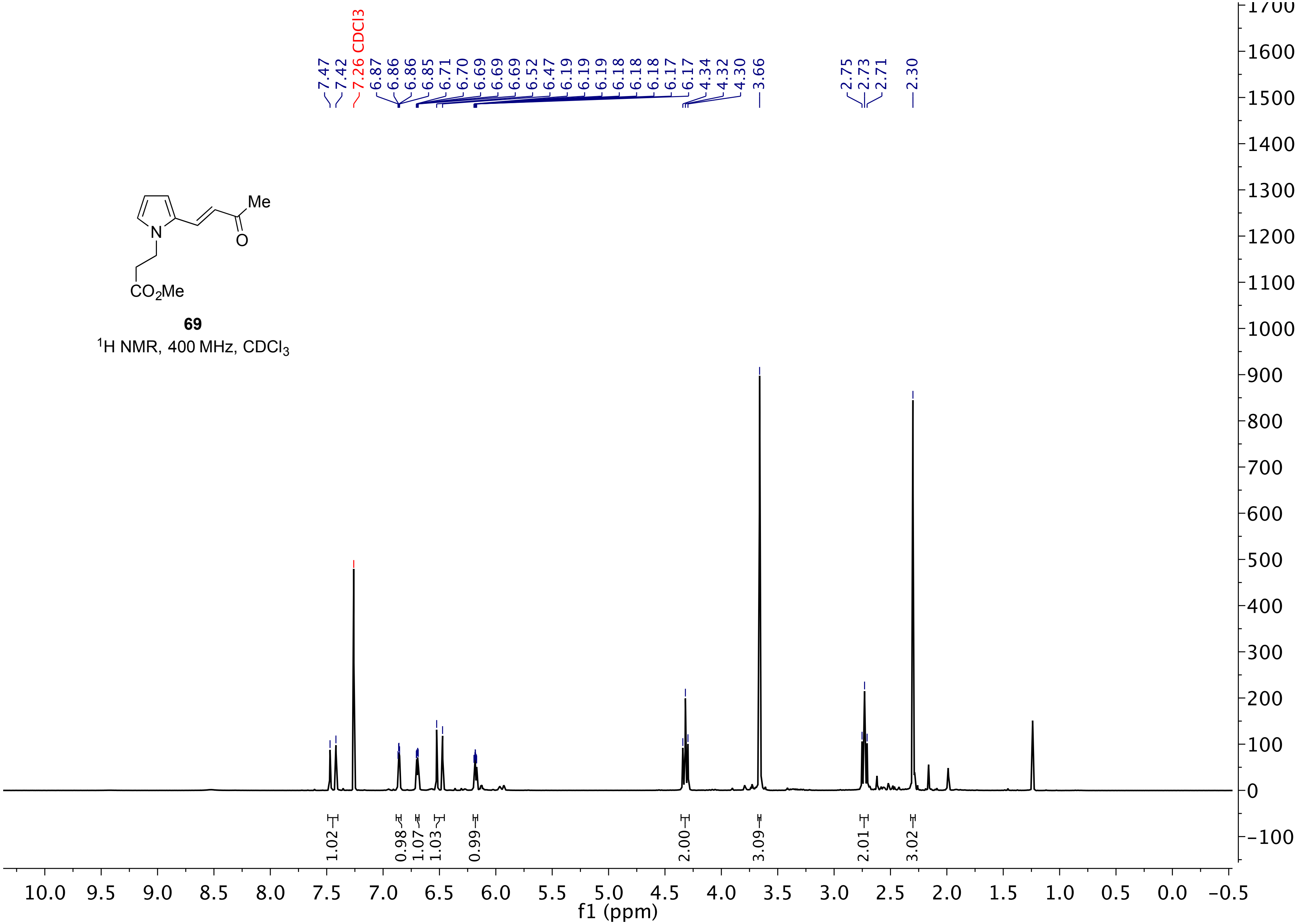

7.47
7.42
7.26 CDCl3
6.87
6.86
6.86
6.85
6.71
6.70
6.69
6.69
6.69
6.52
6.47
6.19
6.19
6.19
6.18
6.18
6.18
6.17
6.17
4.34
4.32
4.30
3.66
2.75
2.73
2.71
2.30
Me
N
O
CO2Me
69
1H NMR, 400 MHz, CDCl3
1.02
0.98
1.07
1.03
0.99
2.00
3.09
2.01
3.02
10.0 9.5 9.0 8.5 8.0 7.5 7.0 6.5 6.0 5.5 5.0 4.5 4.0 3.5 3.0 2.5 2.0 1.5 1.0 0.5 0.0 −0.5
f1 (ppm)
1700 1600 1500 1400 1300 1200 1100 1000 900 800 700 600 500 400 300 200 100 0 −100

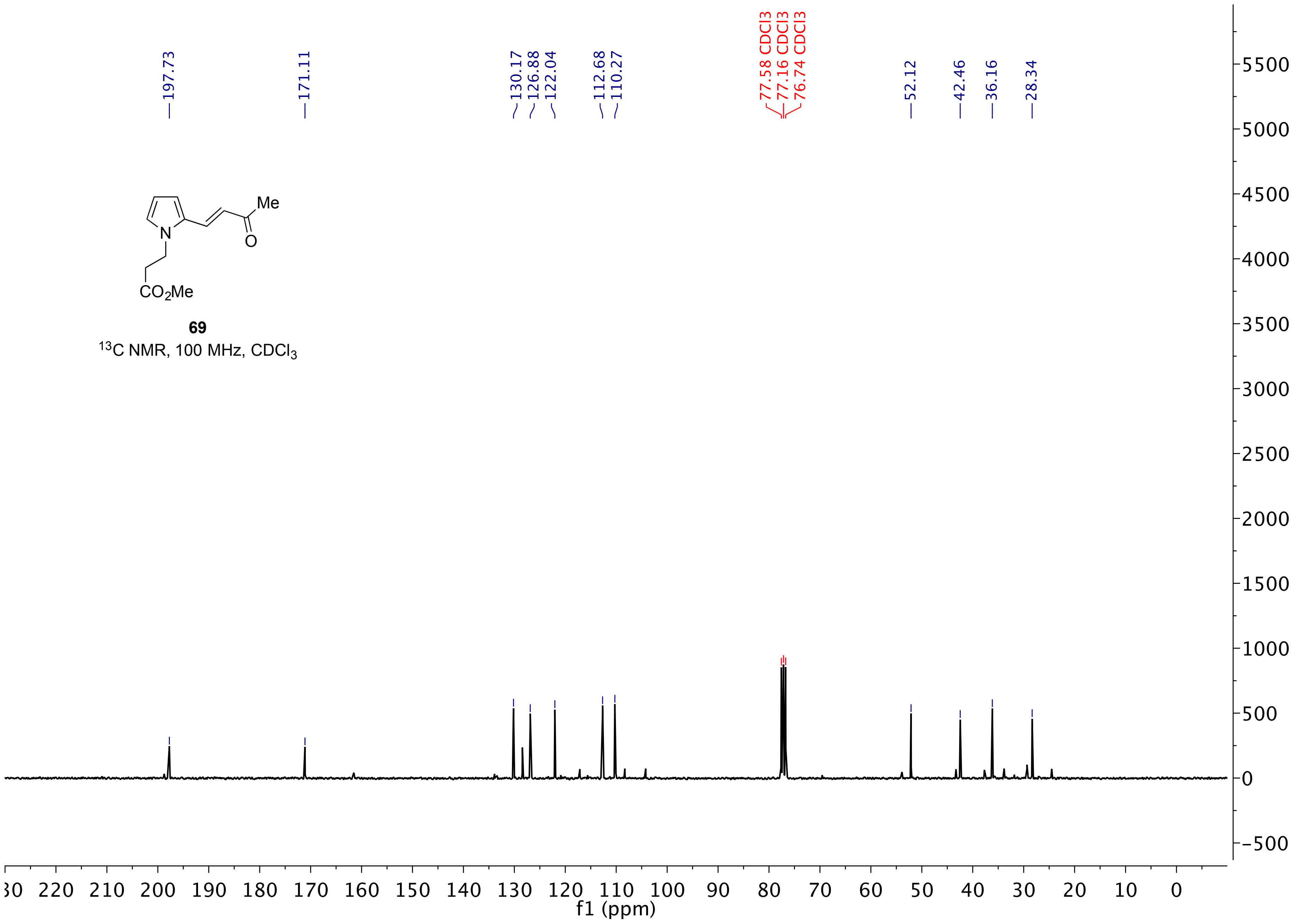

69

^{13}C NMR, 100 MHz, $CDCl_3$

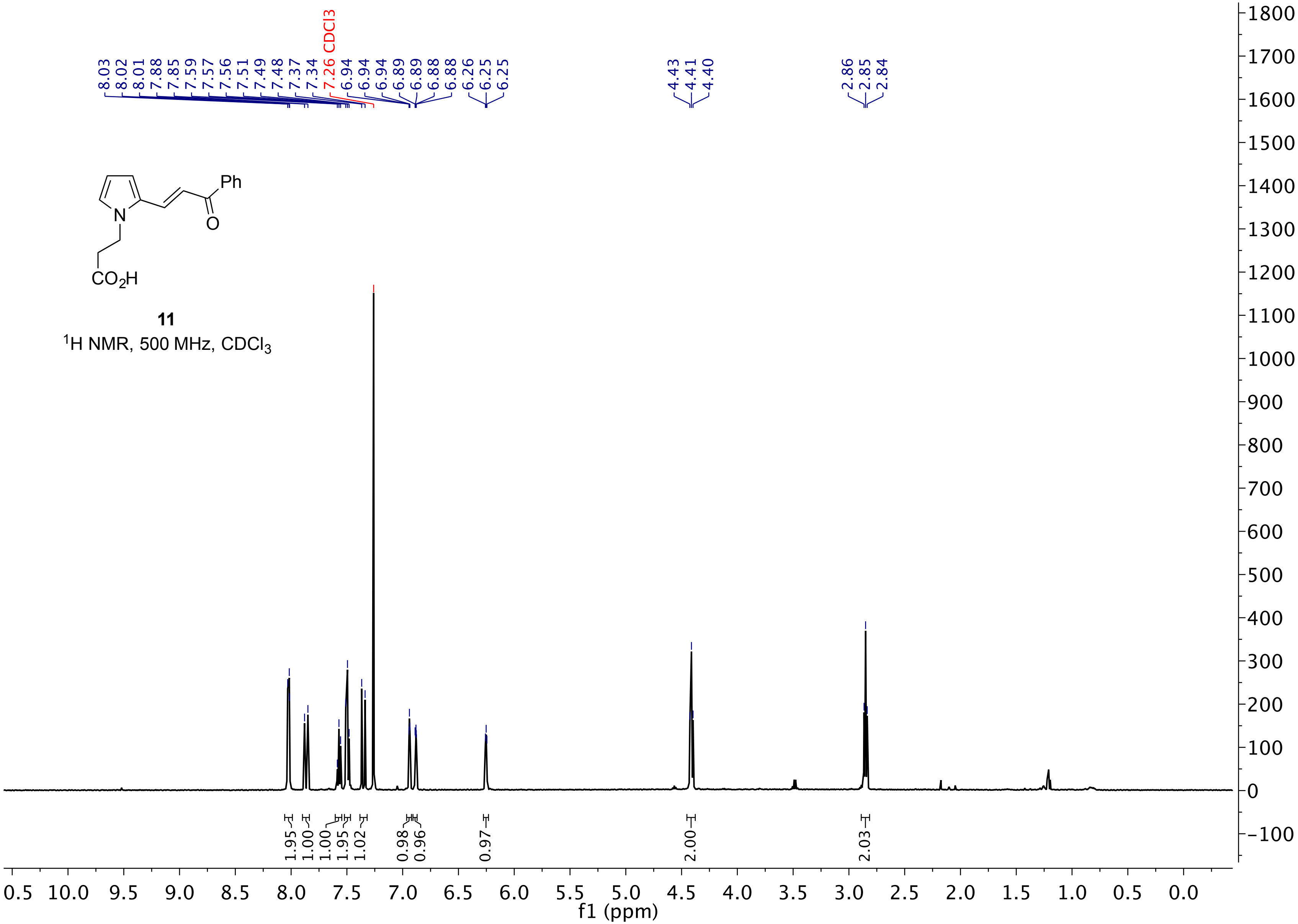
8.03 8.02 8.01 7.88 7.85 7.59 7.57 7.56 7.51 7.49 7.48 7.37 7.34 7.26 CDCl3 6.94 6.94 6.94 6.89 6.89 6.88 6.88 6.26 6.25 6.25
4.43 4.41 4.40
2.86 2.85 2.84
Ph
N
O
CO_2H
11
^{1}H NMR, 500 MHz, $CDCl_3$
1.95 1.00 1.00 1.95 1.02 0.98 0.96 0.97 2.00 2.03
f1 (ppm)

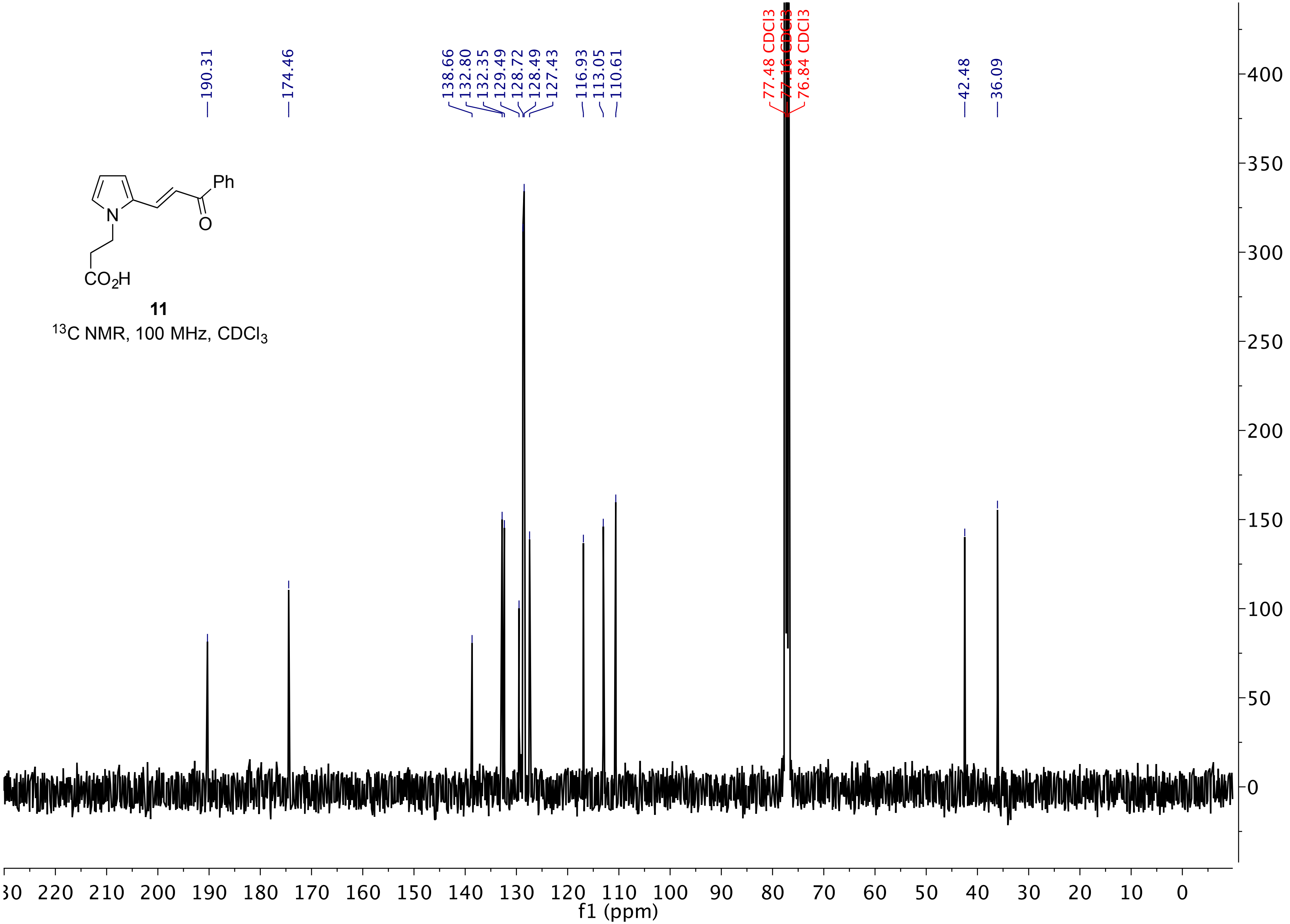
190.31
174.46
138.66
132.80
132.35
129.49
128.72
128.49
127.43
116.93
113.05
110.61
77.48 CDCl3
77.16 CDCl3
76.84 CDCl3
42.48
36.09
Ph
O
N
CO2H
11
13C NMR, 100 MHz, CDCl3
f1 (ppm)

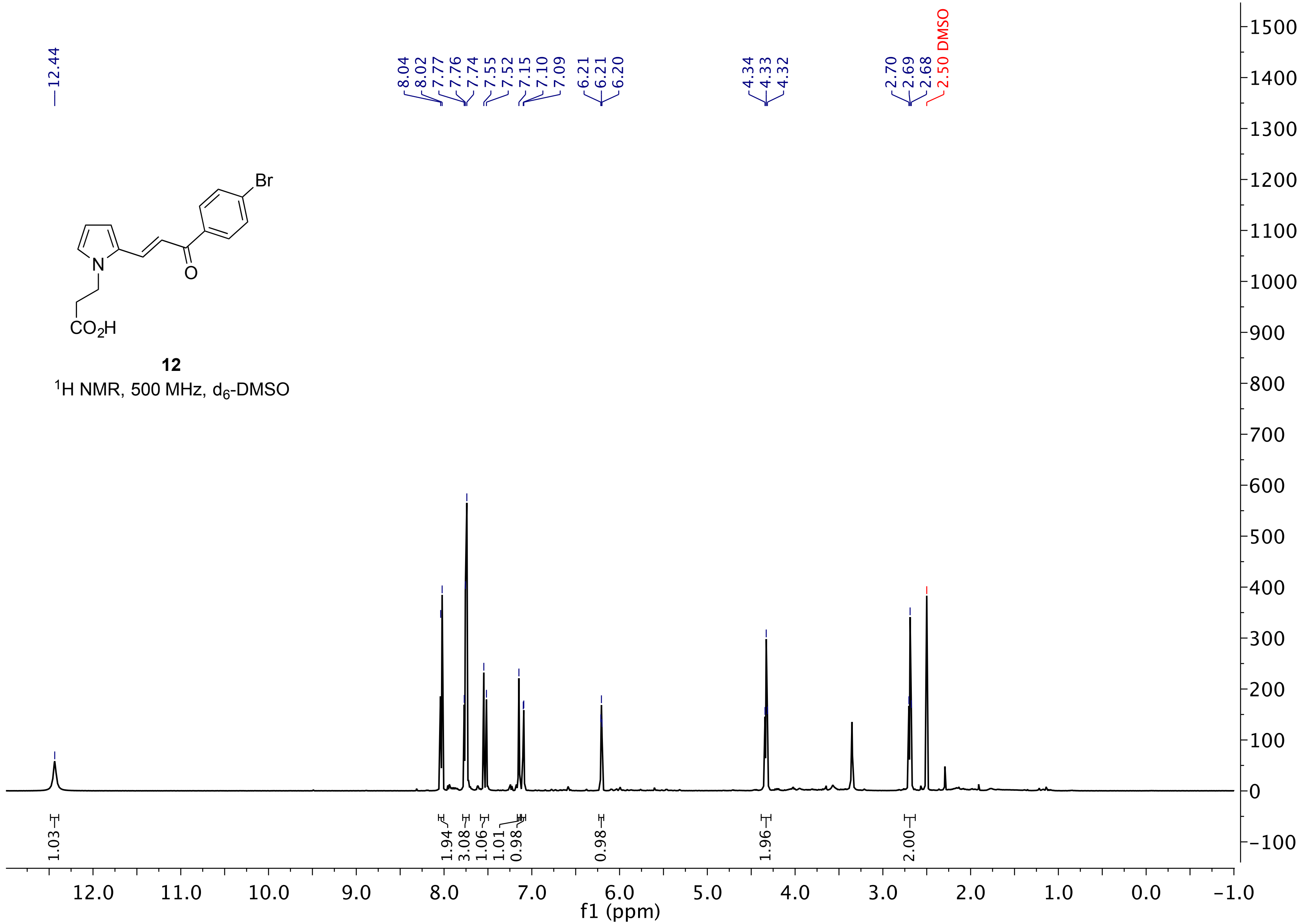
12.44
8.04
8.02
7.77
7.76
7.74
7.55
7.52
7.15
7.10
7.09
6.21
6.21
6.20
4.34
4.33
4.32
2.70
2.69
2.68
2.50 DMSO
Br
N
O
CO2H
12
1H NMR, 500 MHz, d6-DMSO
1.03
1.94
3.08
1.06
1.01
0.98
0.98
1.96
2.00
f1 (ppm)

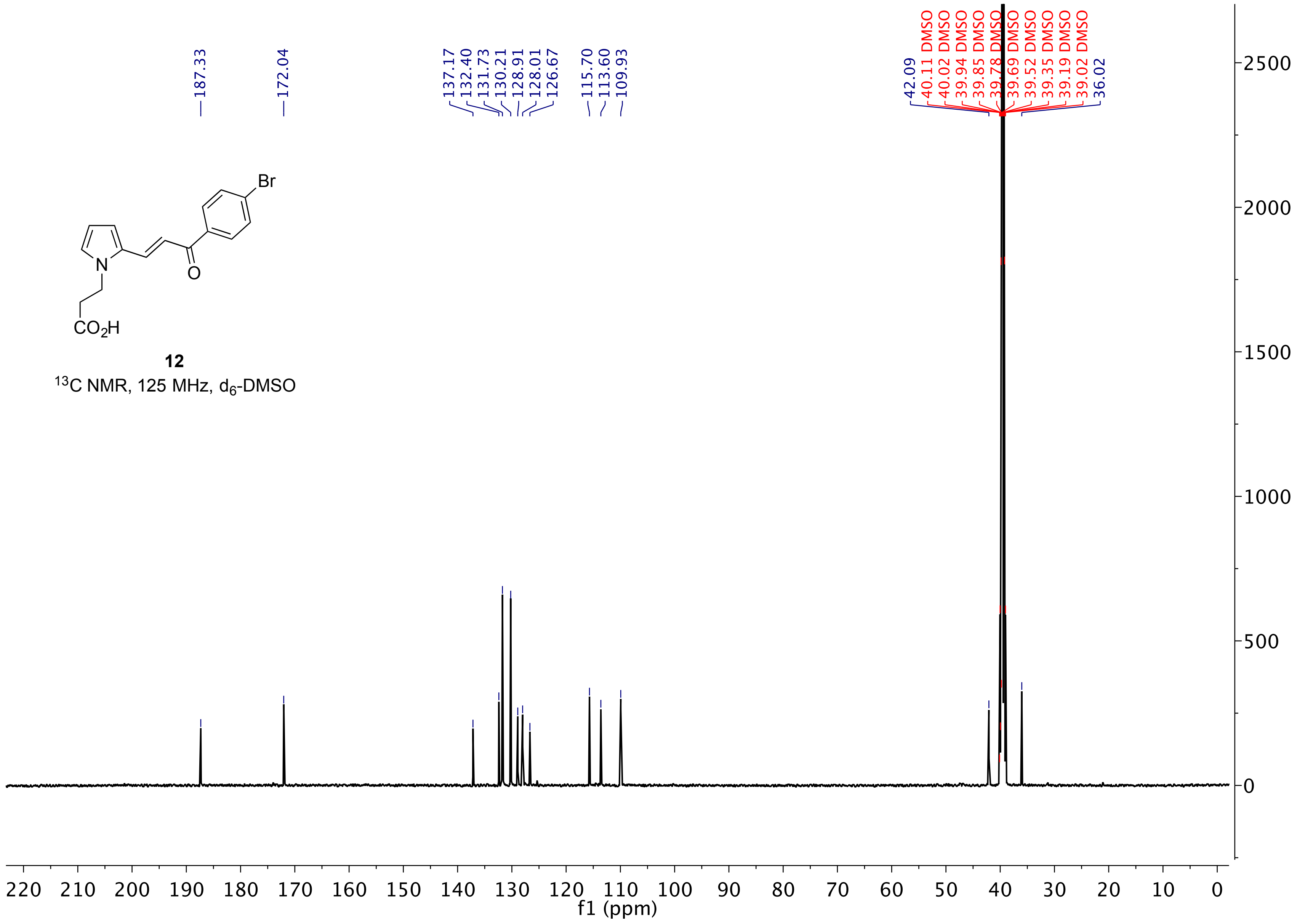
187.33
172.04
137.17
132.40
131.73
130.21
128.91
128.01
126.67
115.70
113.60
109.93
42.09
40.11 DMSO
40.02 DMSO
39.94 DMSO
39.85 DMSO
39.78 DMSO
39.69 DMSO
39.52 DMSO
39.35 DMSO
39.19 DMSO
39.02 DMSO
36.02
Br
N
O
CO_2H
12
^{13}C NMR, 125 MHz, d_6-DMSO
f1 (ppm)

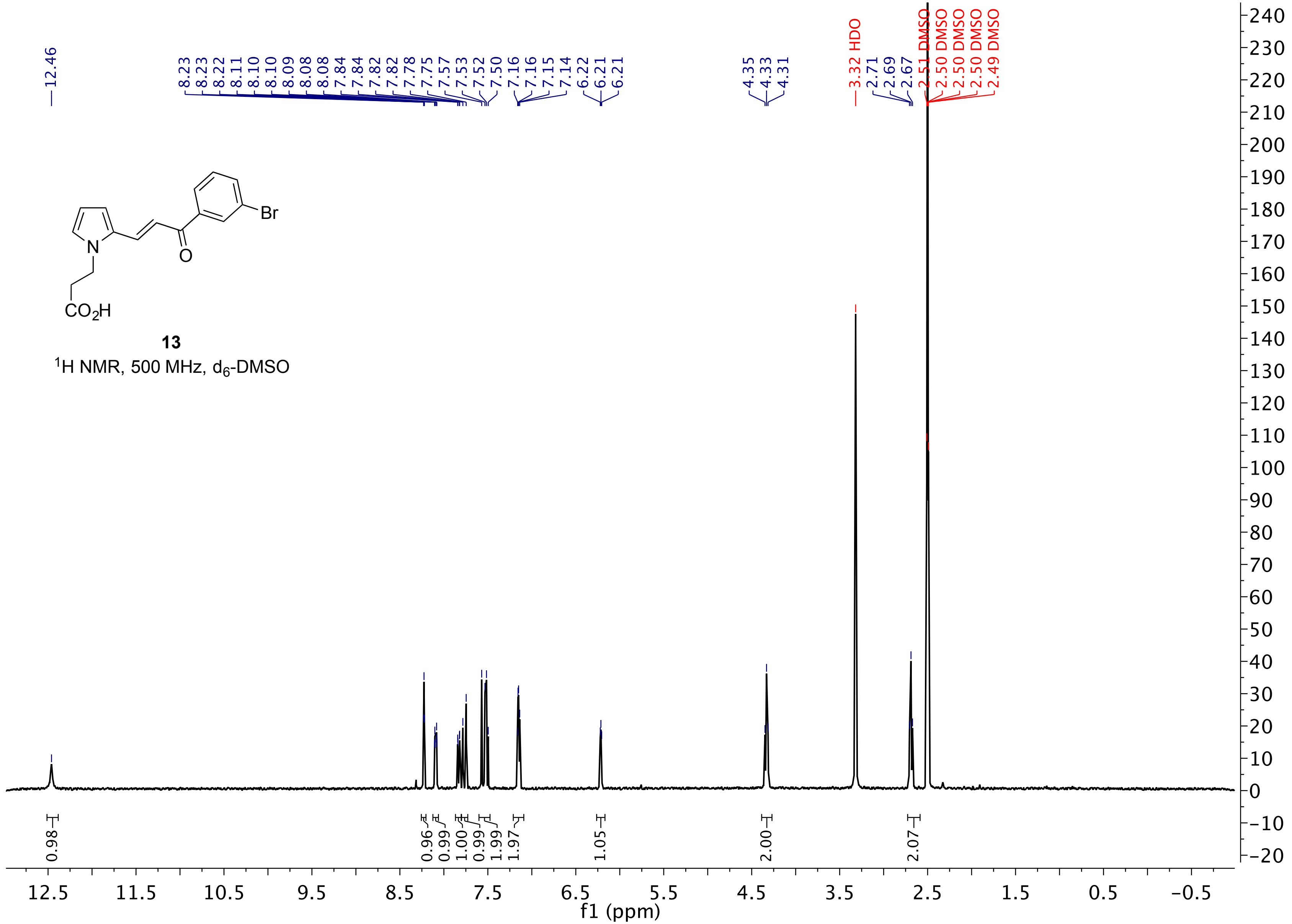

13

^{1}H NMR, 500 MHz, d_6-DMSO

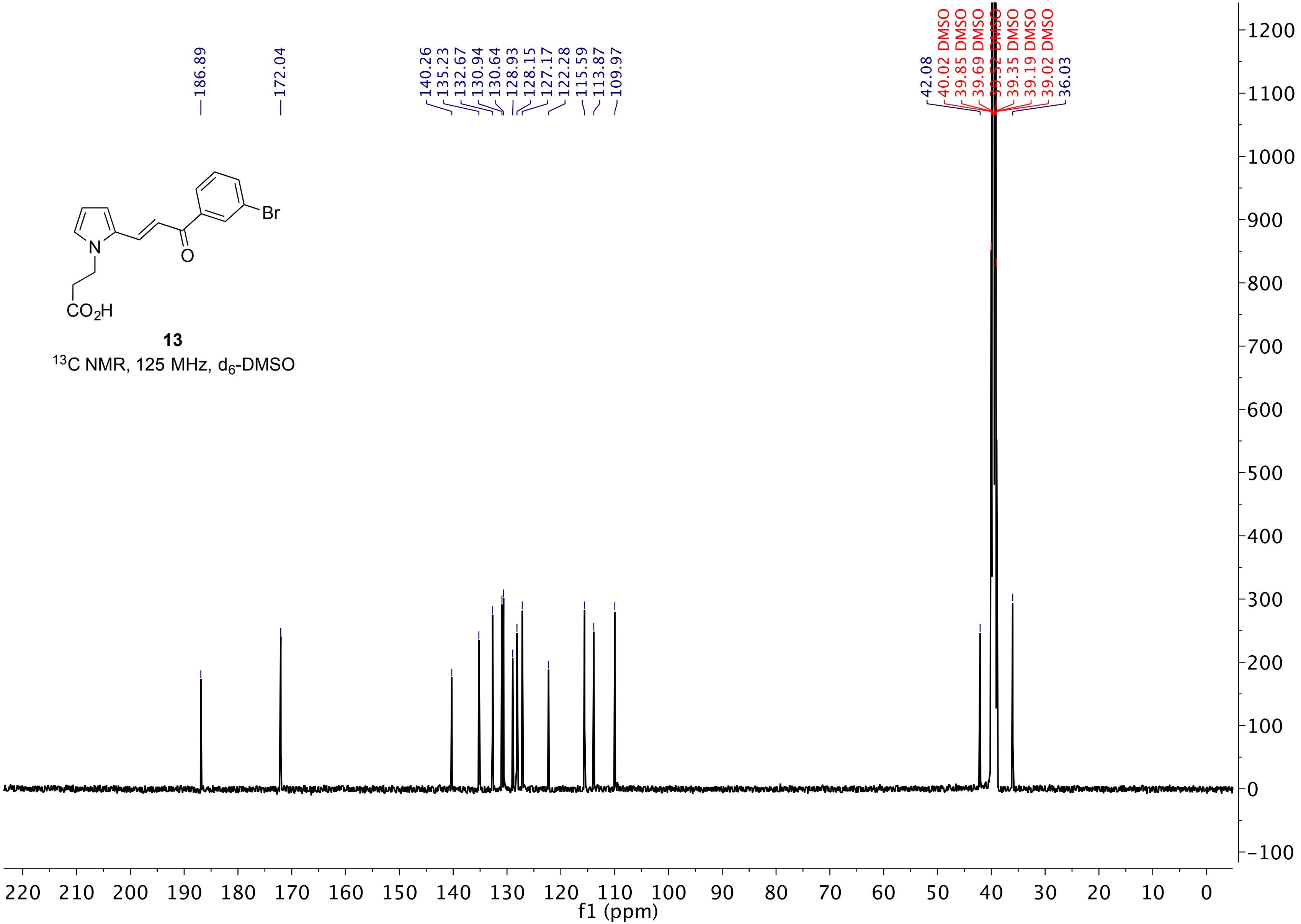
186.89
172.04
140.26
135.23
132.67
130.94
130.64
128.93
128.15
127.17
122.28
115.59
113.87
109.97
42.08
40.02 DMSO
39.85 DMSO
39.69 DMSO
39.52 DMSO
39.35 DMSO
39.19 DMSO
39.02 DMSO
36.03
Br
N
O
CO_2H
13
^{13}C NMR, 125 MHz, d_6-DMSO
f1 (ppm)

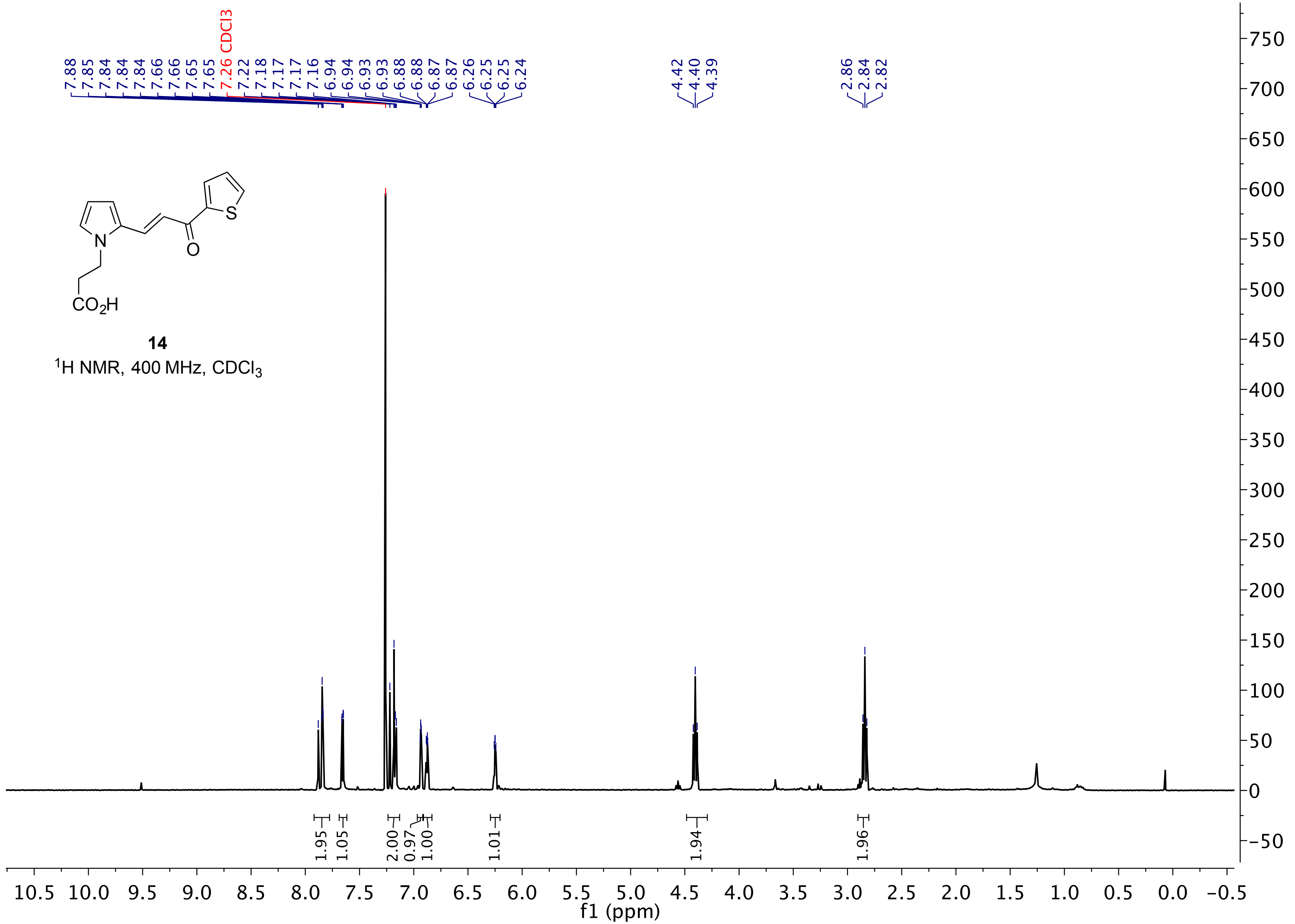

7.88
7.85
7.84
7.84
7.84
7.66
7.66
7.65
7.65
7.26 CDCl3
7.22
7.18
7.17
7.17
7.16
6.94
6.94
6.93
6.93
6.88
6.88
6.87
6.87
6.26
6.25
6.25
6.24
4.42
4.40
4.39
2.86
2.84
2.82
S
N
O
CO2H
14
1H NMR, 400 MHz, CDCl3
1.95
1.05
2.00
0.97
1.00
1.01
1.94
1.96
f1 (ppm)

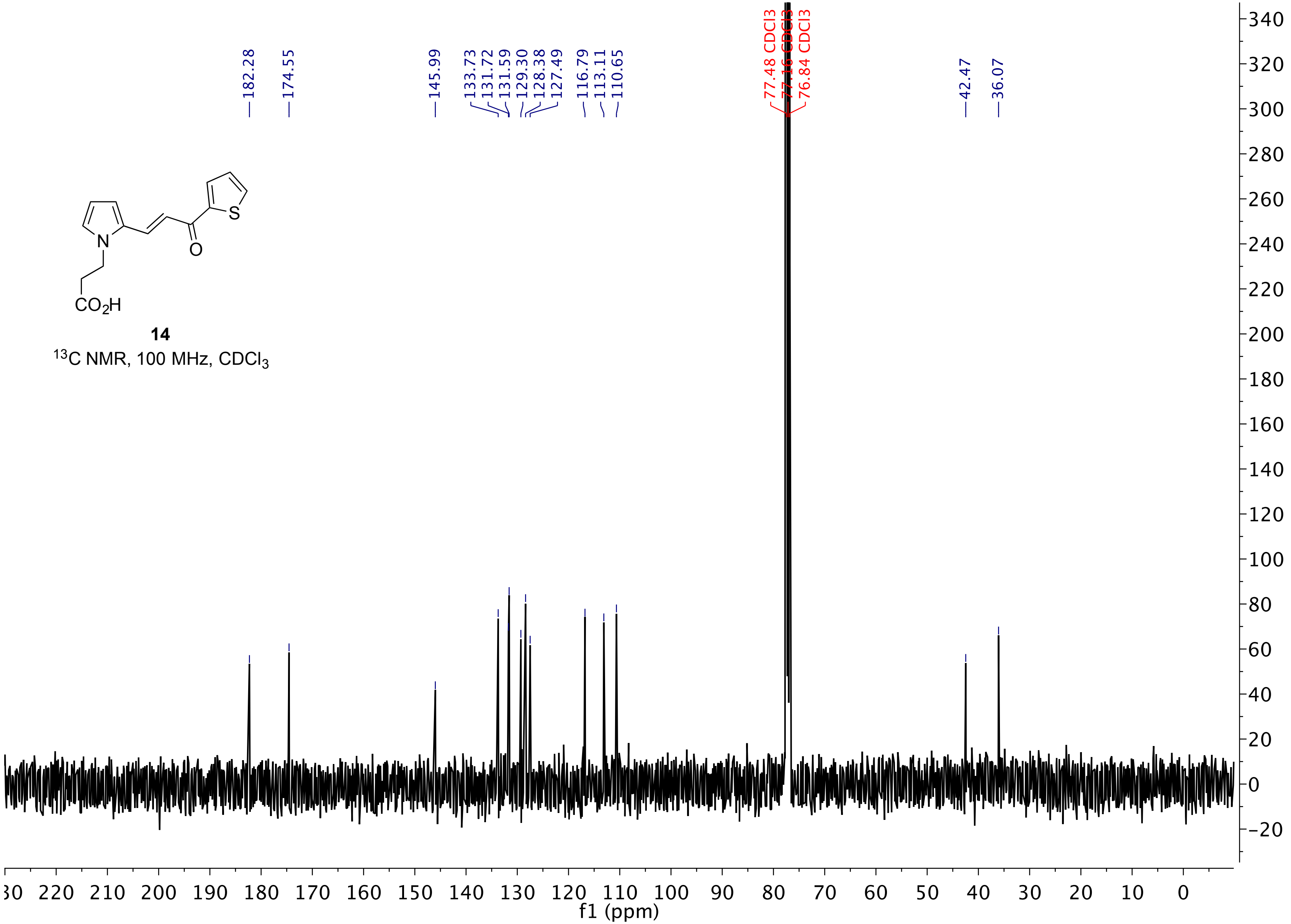

14

^{13}C NMR, 100 MHz, $CDCl_3$

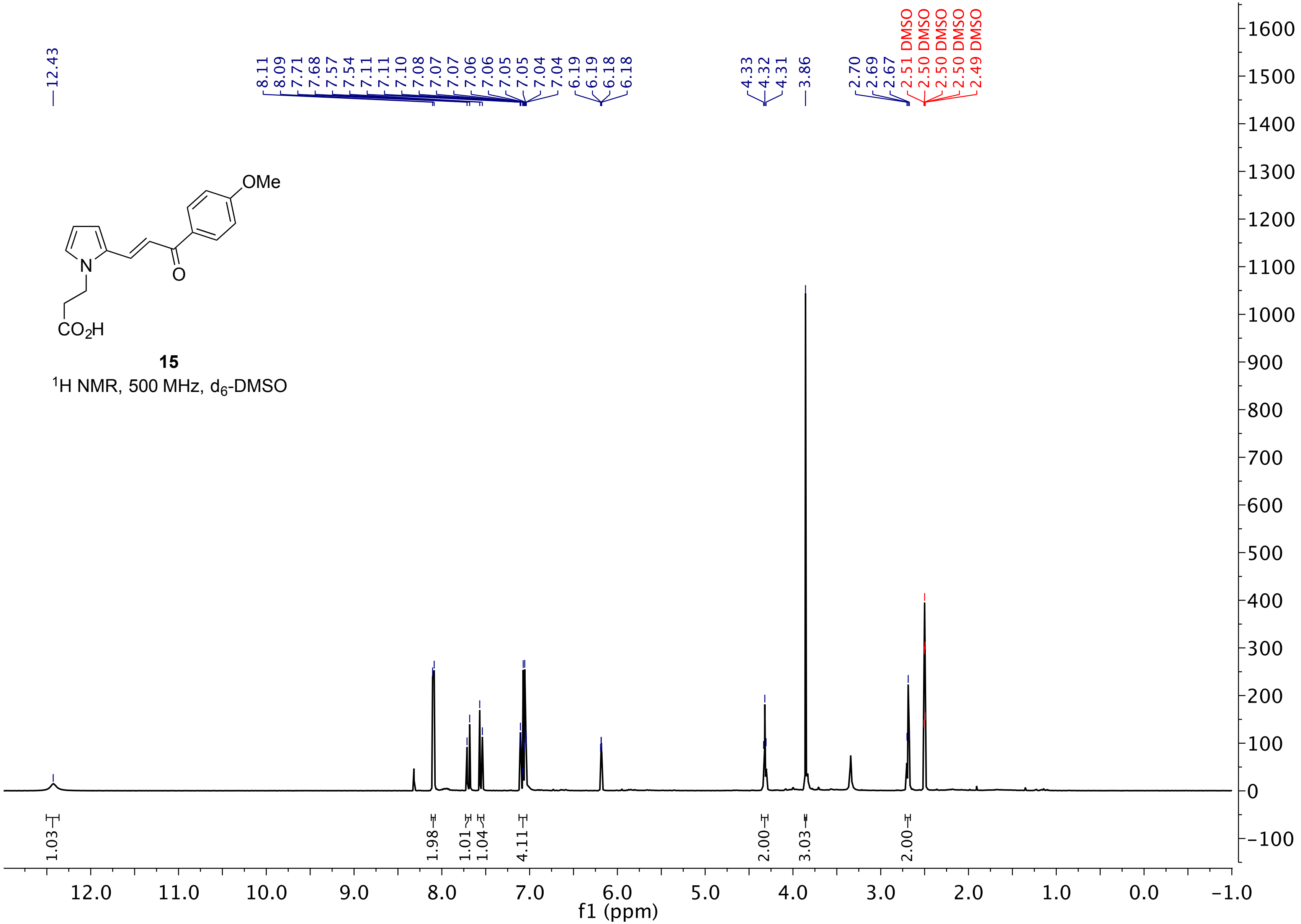
OMe
N
O
CO2H
15
1H NMR, 500 MHz, d6-DMSO
12.43
8.11
8.09
7.71
7.68
7.57
7.54
7.11
7.11
7.10
7.08
7.07
7.07
7.06
7.06
7.05
7.05
7.04
7.04
6.19
6.19
6.18
6.18
4.33
4.32
4.31
3.86
2.70
2.69
2.67
2.51 DMSO
2.50 DMSO
2.50 DMSO
2.50 DMSO
2.49 DMSO
1.03
1.98
1.01
1.04
4.11
2.00
3.03
2.00
f1 (ppm)

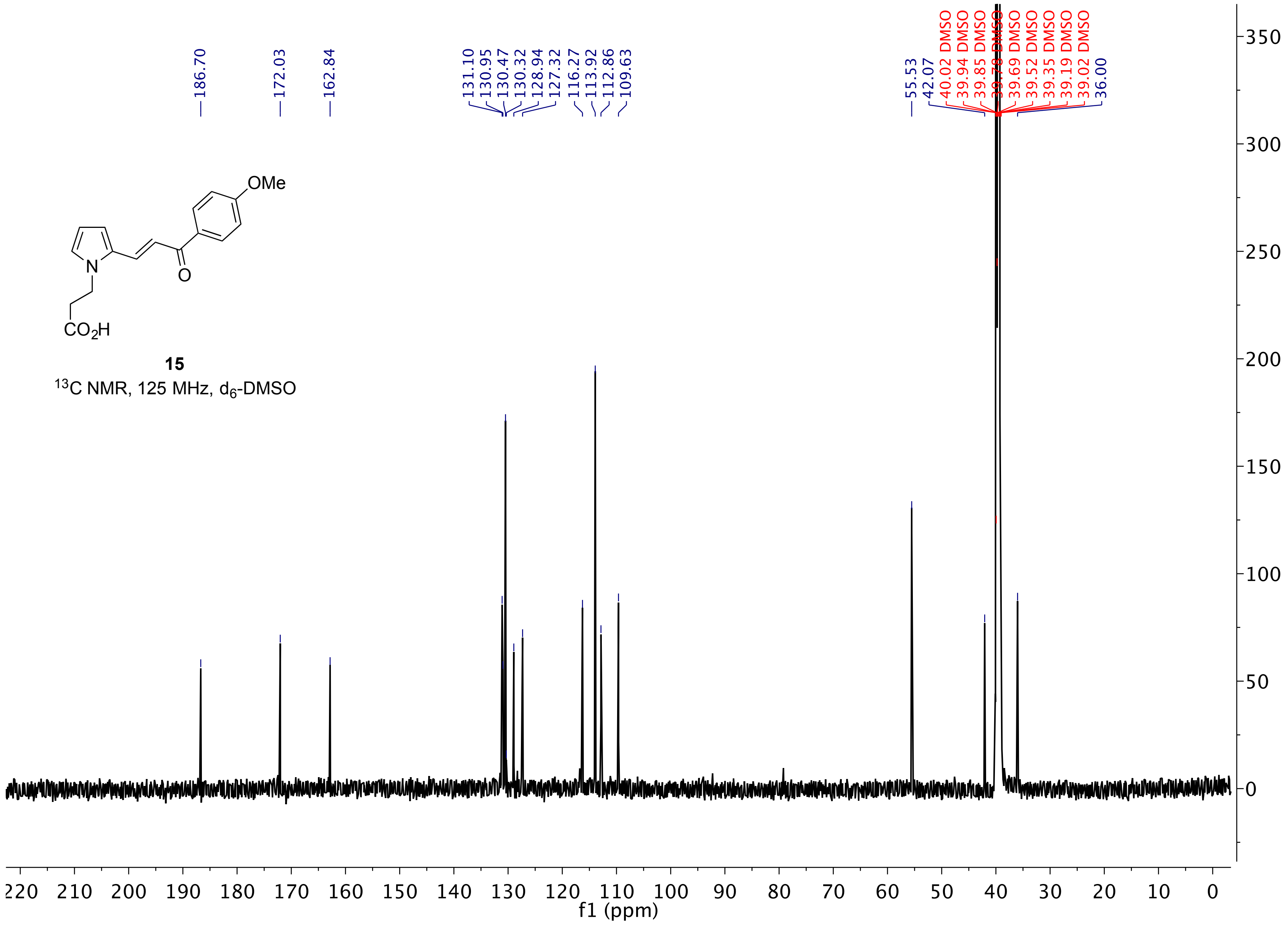

186.70
172.03
162.84
131.10
130.95
130.47
130.32
128.94
127.32
116.27
113.92
112.86
109.63
55.53
42.07
40.02 DMSO
39.94 DMSO
39.85 DMSO
39.78 DMSO
39.69 DMSO
39.52 DMSO
39.35 DMSO
39.19 DMSO
39.02 DMSO
36.00
OMe
N
O
CO_2H
15
^{13}C NMR, 125 MHz, d_6-DMSO
f1 (ppm)

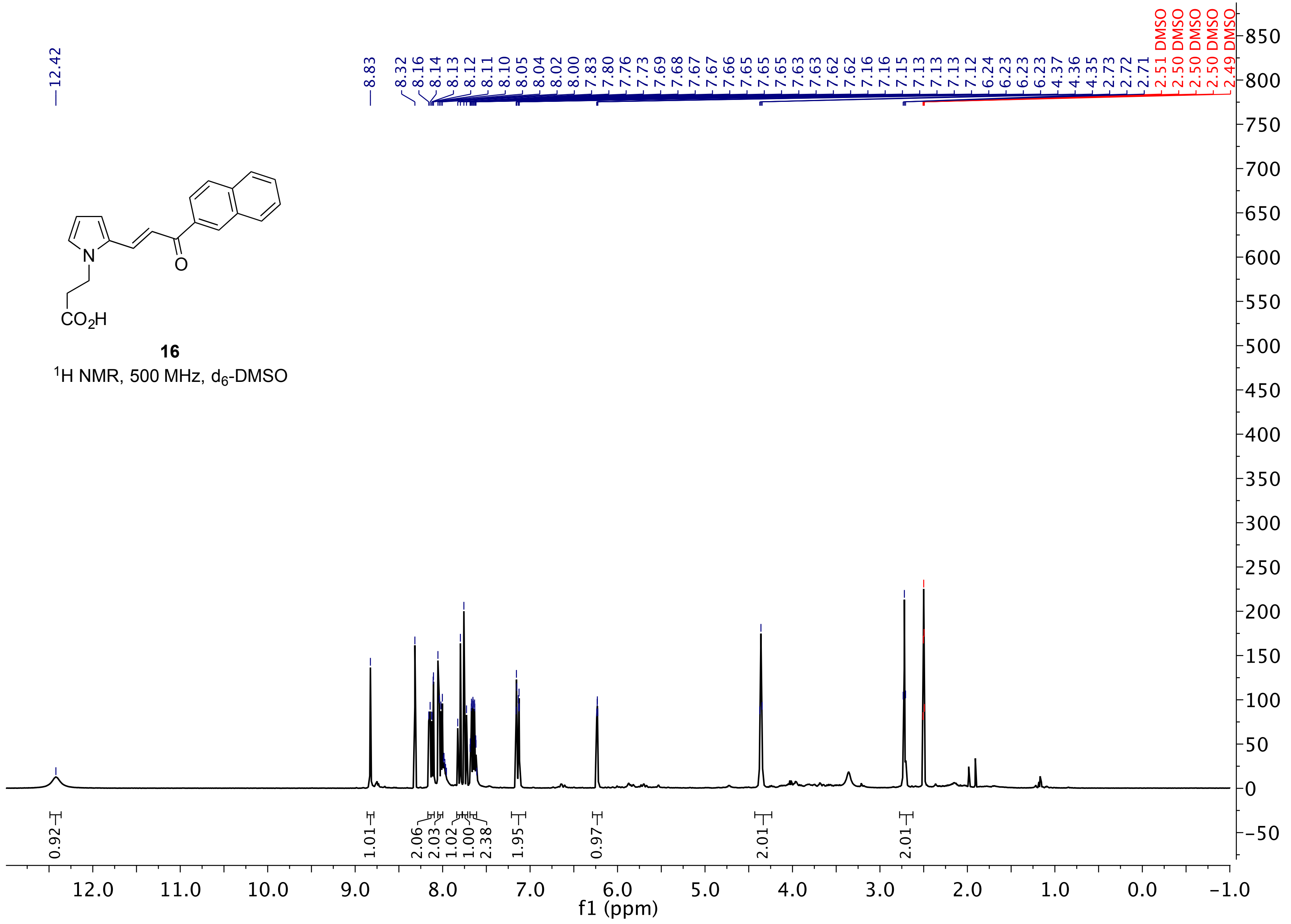
16
^{1}H NMR, 500 MHz, d$_6$-DMSO
CO$_2$H
N
O
12.42
8.83
8.32
8.16
8.14
8.13
8.12
8.11
8.10
8.05
8.04
8.02
8.00
7.83
7.80
7.76
7.73
7.69
7.68
7.67
7.67
7.66
7.65
7.65
7.65
7.63
7.63
7.62
7.62
7.16
7.16
7.15
7.13
7.13
7.13
7.12
6.24
6.23
6.23
6.23
4.37
4.36
4.35
2.73
2.72
2.71
2.51 DMSO
2.50 DMSO
2.50 DMSO
2.50 DMSO
2.49 DMSO
0.92
1.01
2.06
2.03
1.02
1.00
2.38
1.95
0.97
2.01
2.01
f1 (ppm)

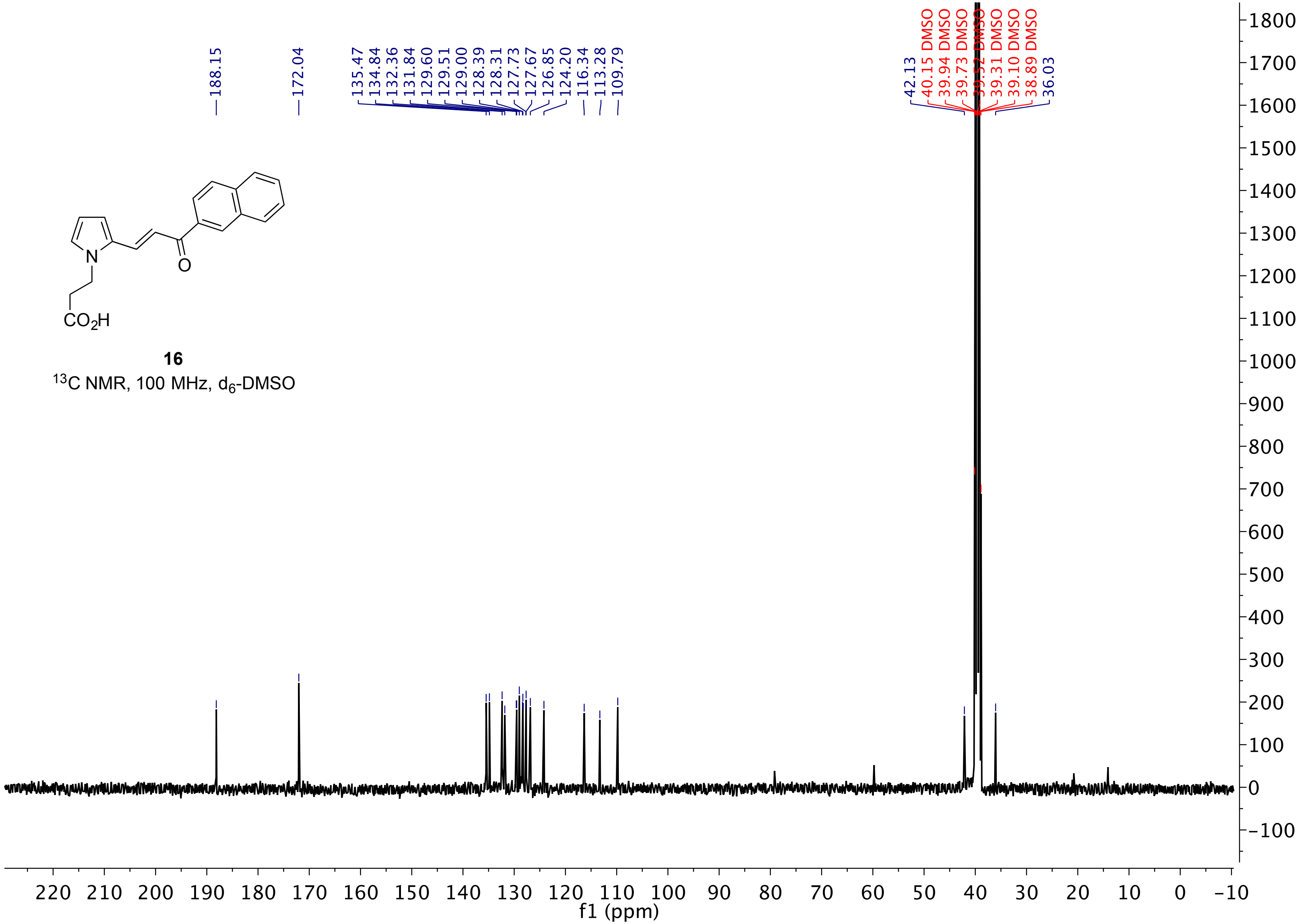

16

^{13}C NMR, 100 MHz, d_6-DMSO

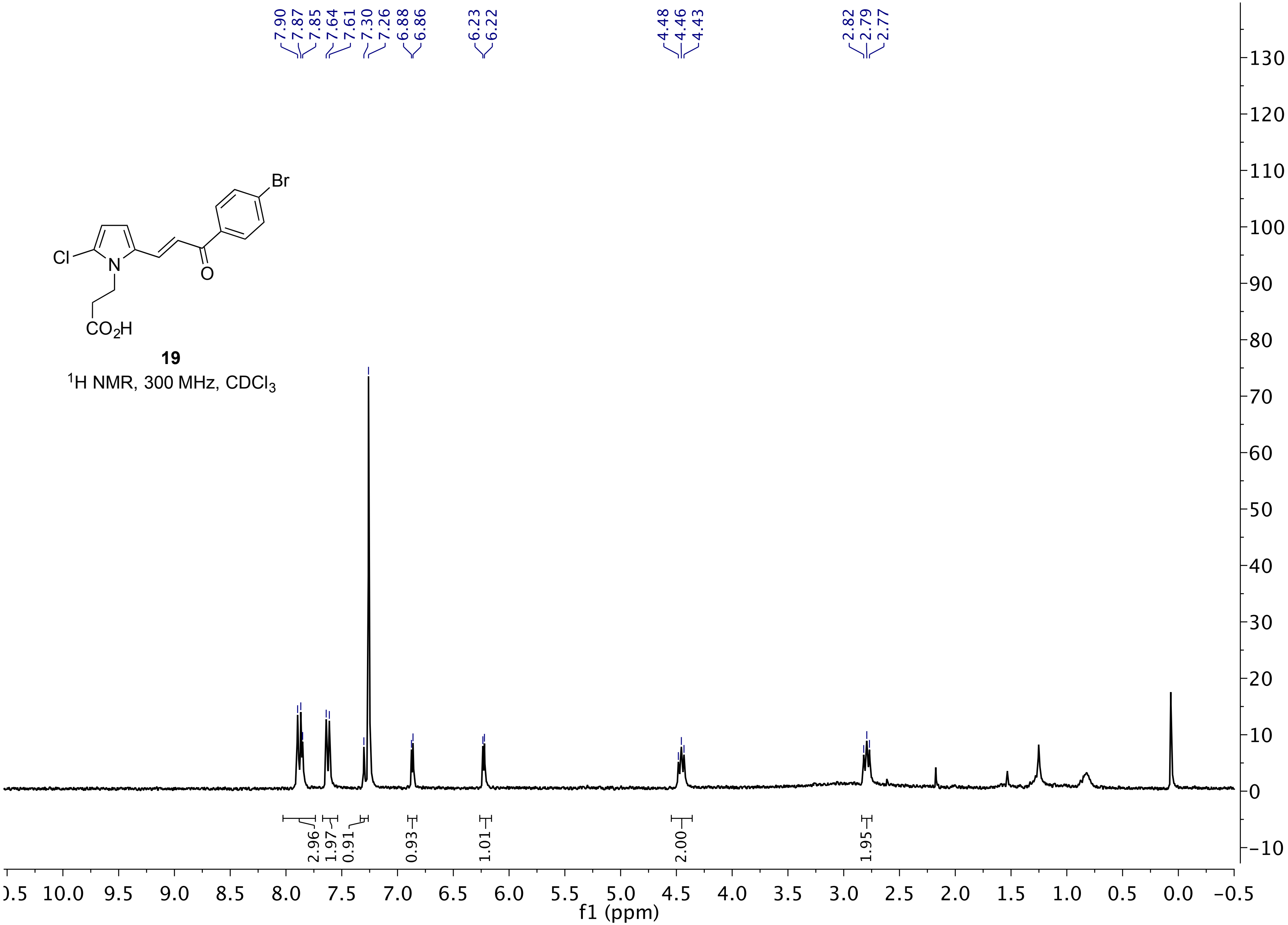

19

^1H NMR, 300 MHz, $CDCl_3$

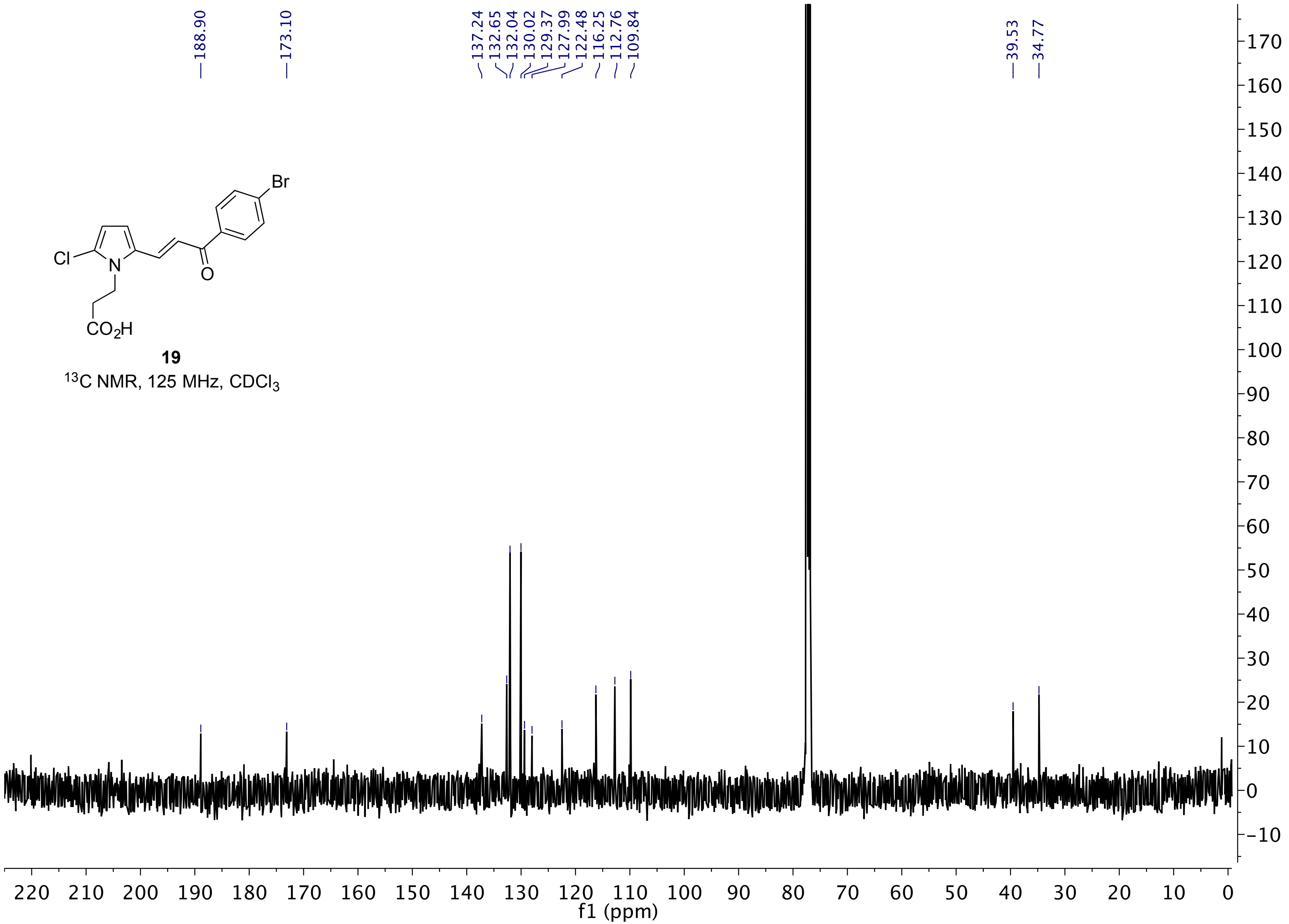
188.90
173.10
137.24
132.65
132.04
130.02
129.37
127.99
122.48
116.25
112.76
109.84
39.53
34.77
Br
Cl
N
O
CO_2H
19
^{13}C NMR, 125 MHz, $CDCl_3$
f1 (ppm)

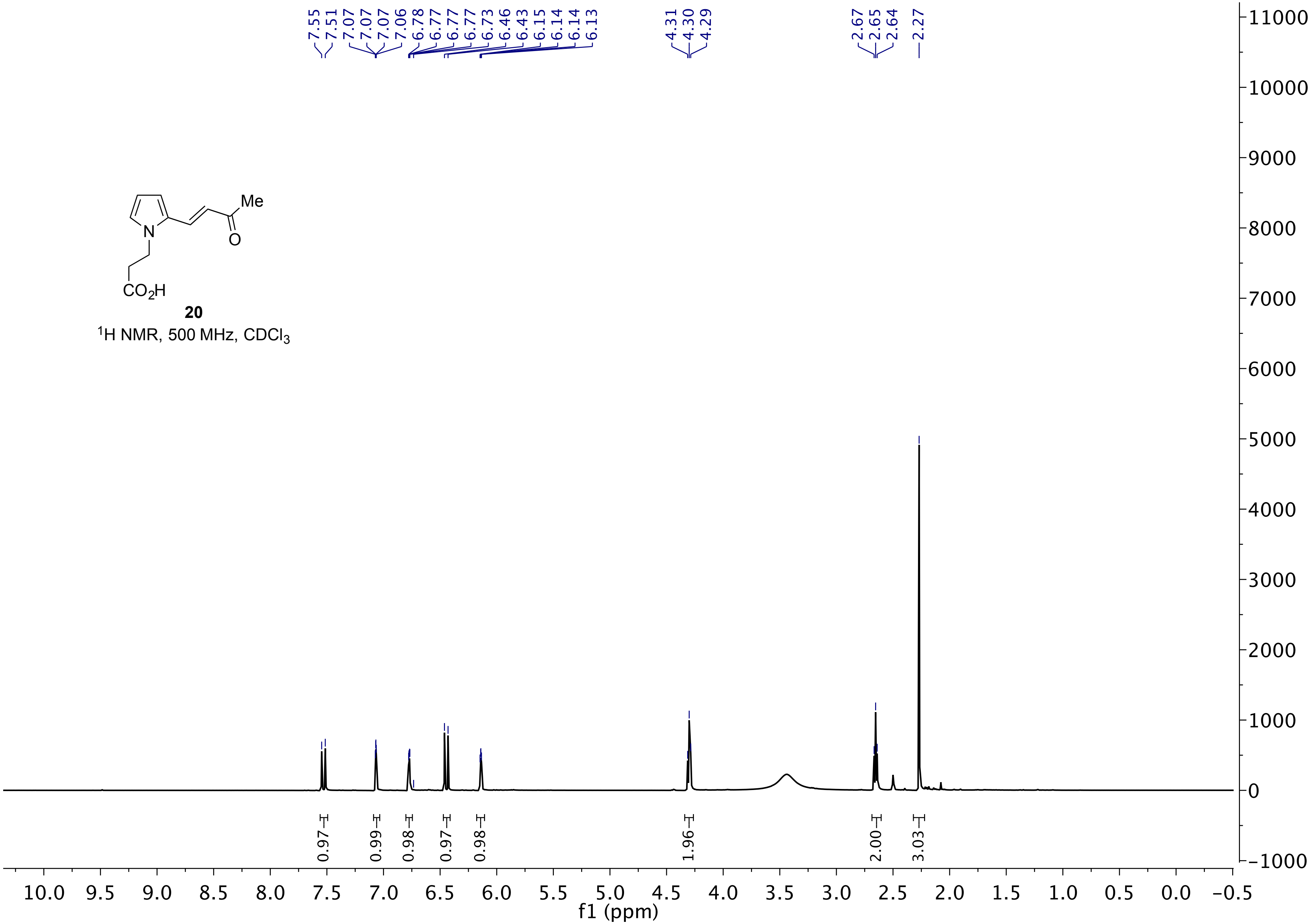

20

^{1}H NMR, 500 MHz, $CDCl_3$

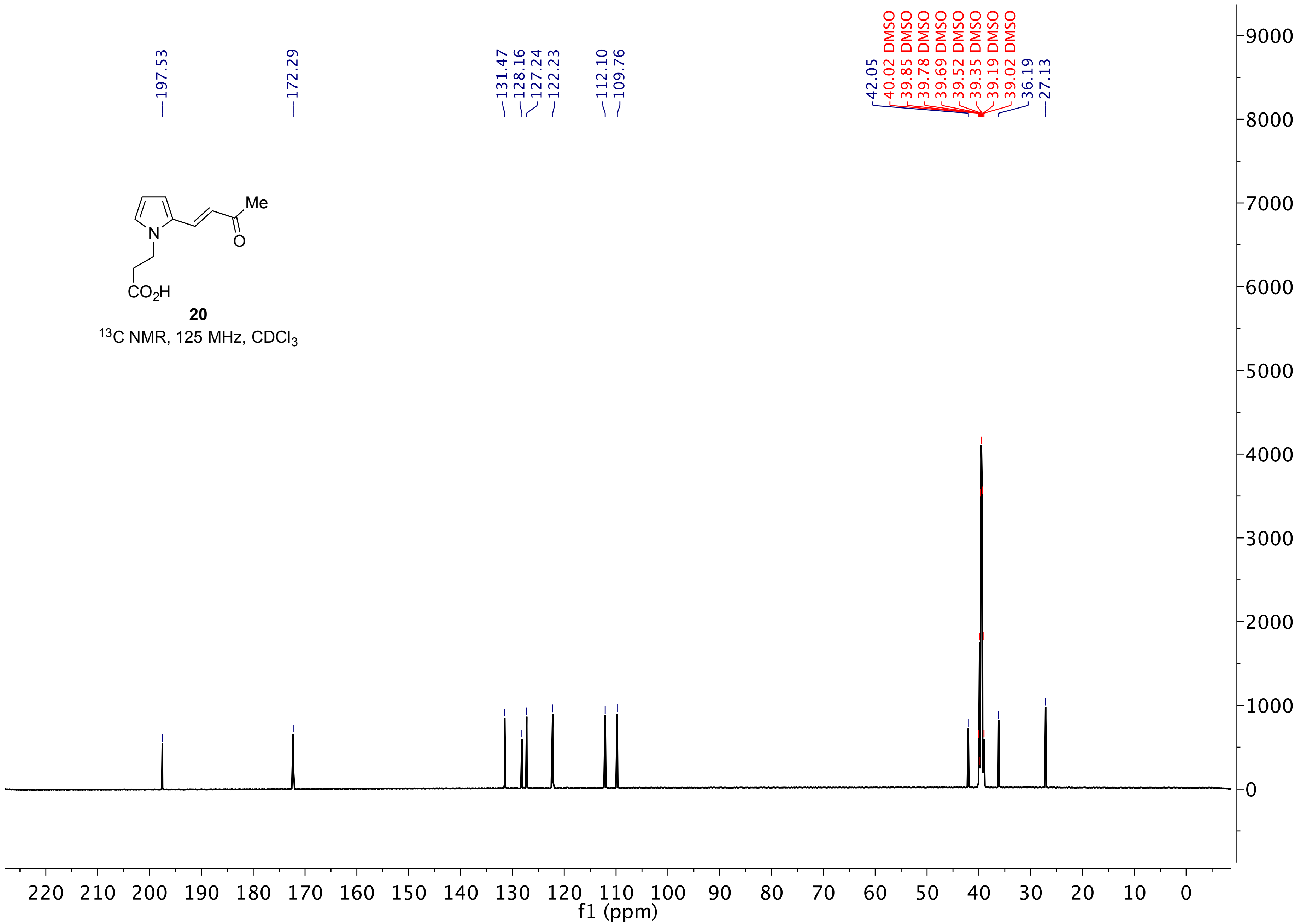
197.53
172.29
131.47
128.16
127.24
122.23
112.10
109.76
42.05
40.02 DMSO
39.85 DMSO
39.78 DMSO
39.69 DMSO
39.52 DMSO
39.35 DMSO
39.19 DMSO
39.02 DMSO
36.19
27.13
Me
N
O
CO2H
20
13C NMR, 125 MHz, CDCl3
f1 (ppm)

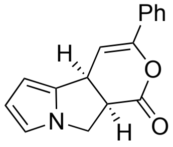

21

^{1}H NMR, 500 MHz, $CDCl_3$

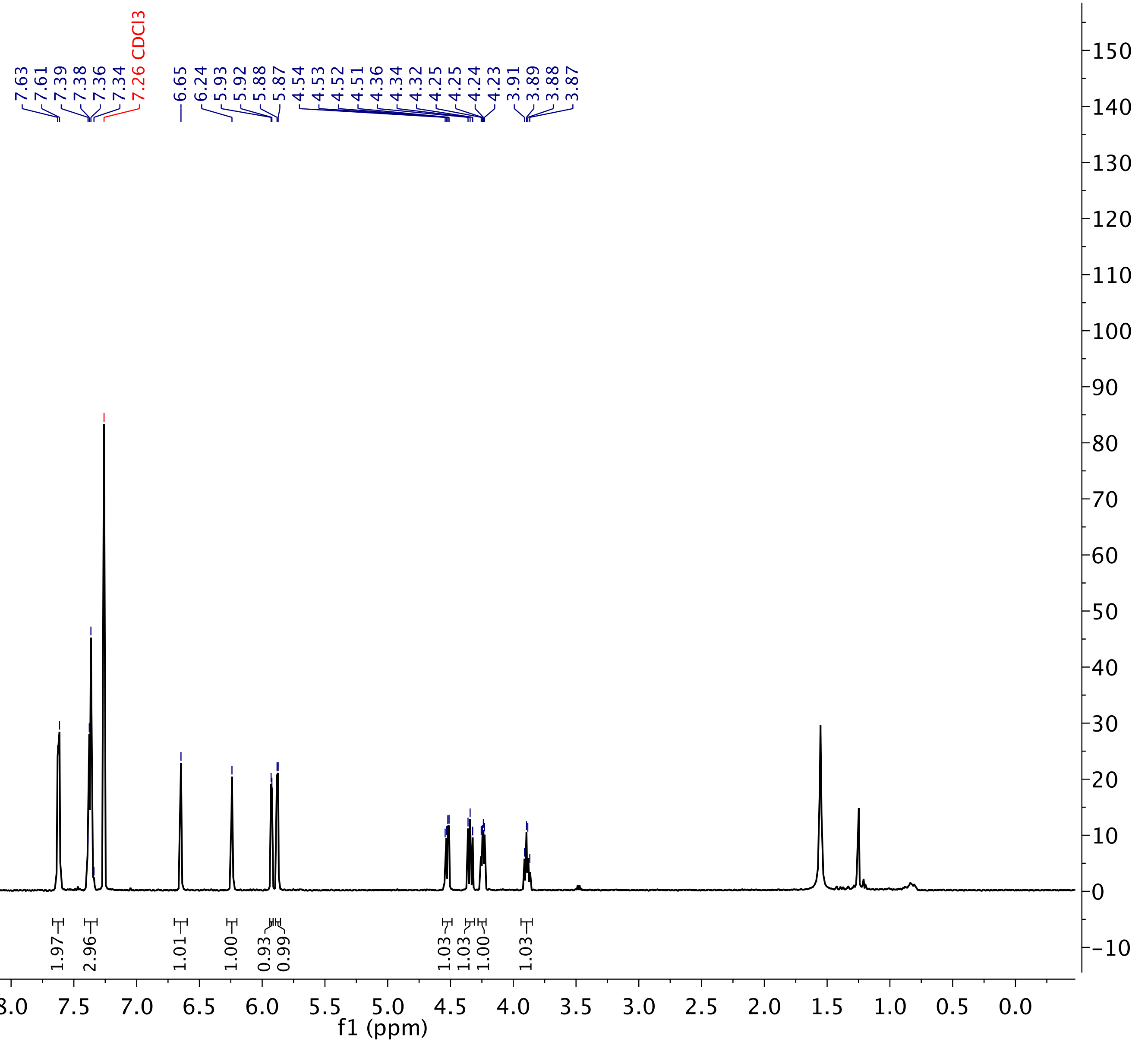

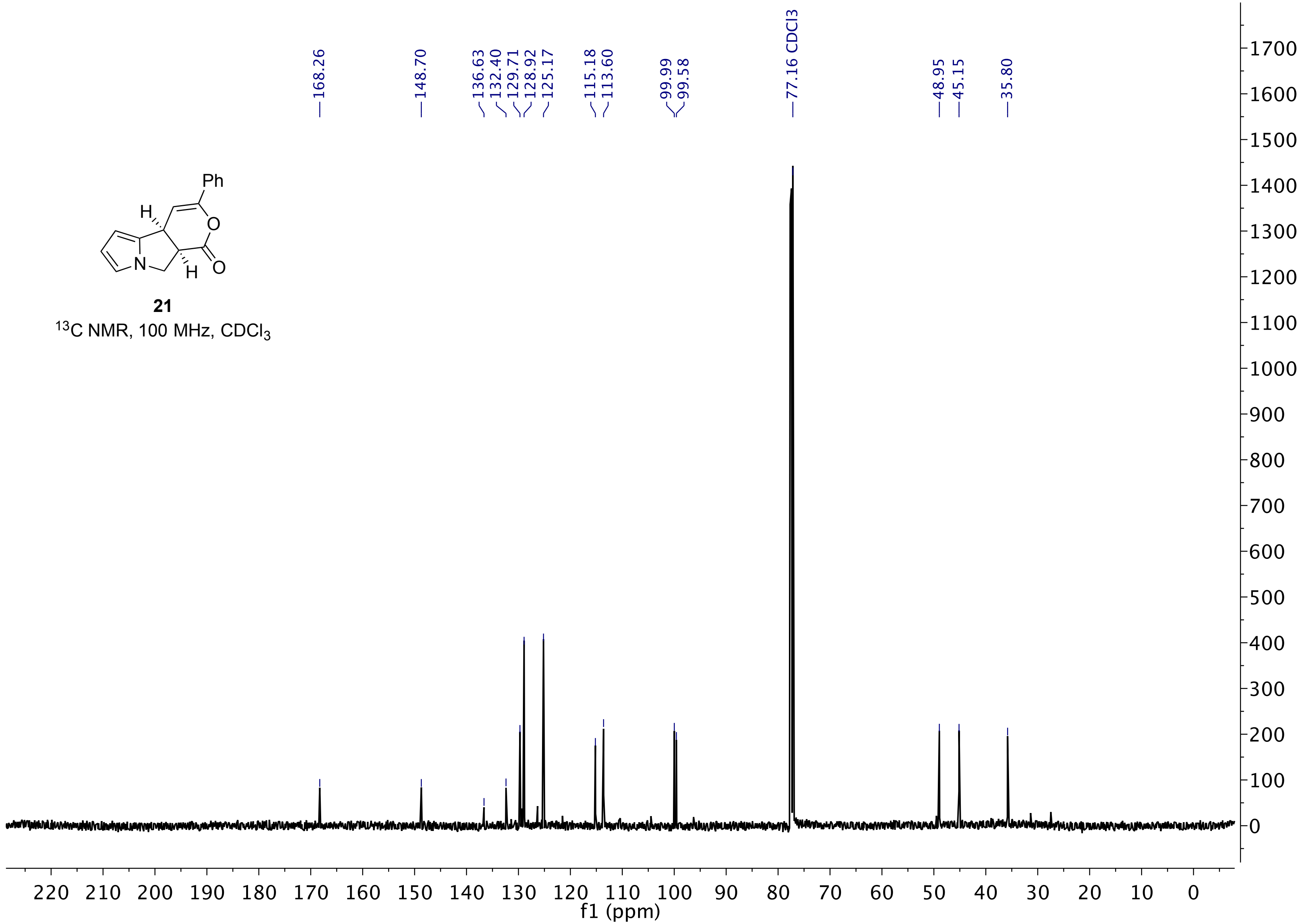

168.26
148.70
136.63
132.40
129.71
128.92
125.17
115.18
113.60
99.99
99.58
77.16 CDCl3
48.95
45.15
35.80
Ph
H
O
N
H
O
21
13C NMR, 100 MHz, CDCl3
f1 (ppm)

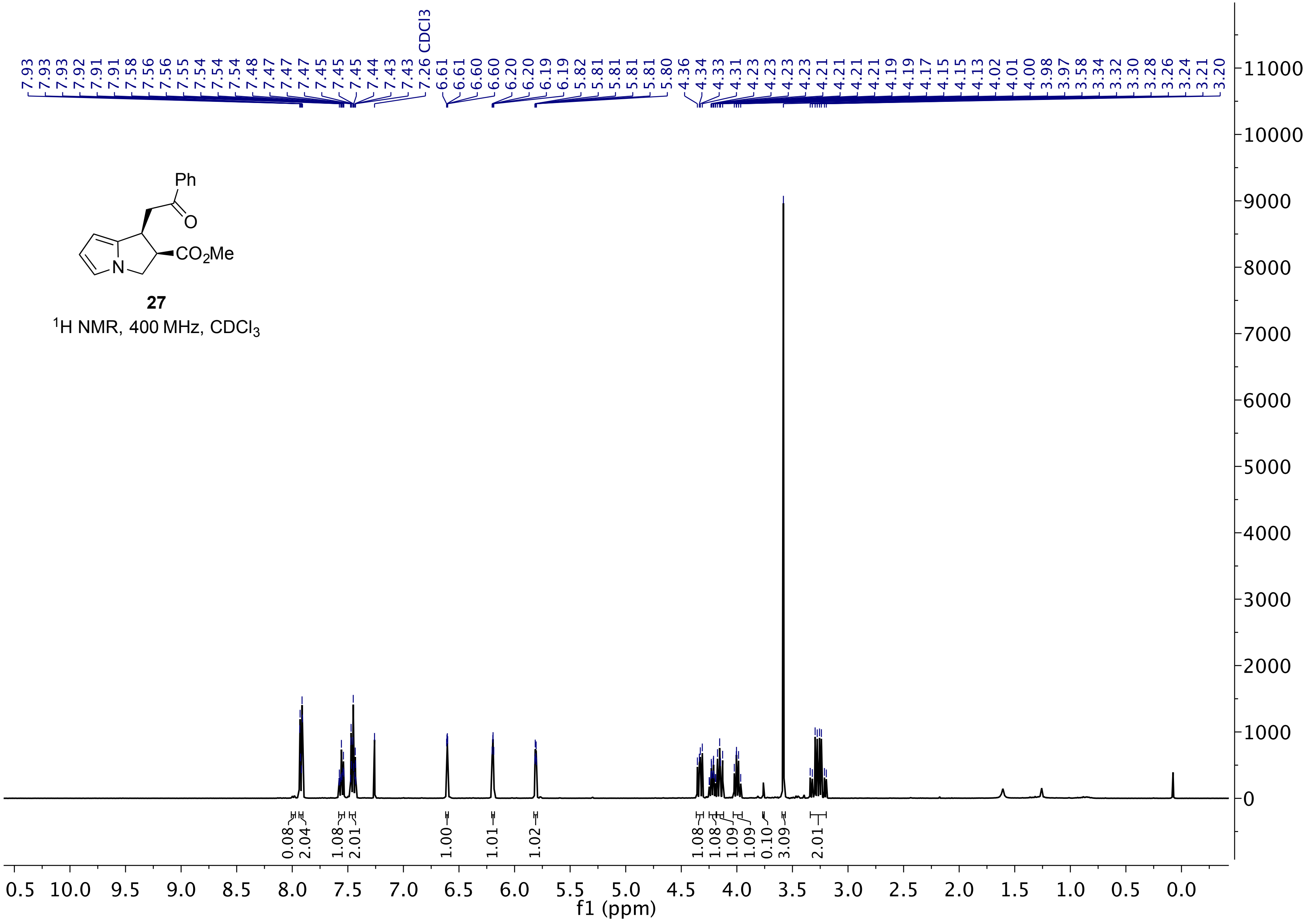

Ph
O
CO2Me
N
27
1H NMR, 400 MHz, CDCl3
f1 (ppm)

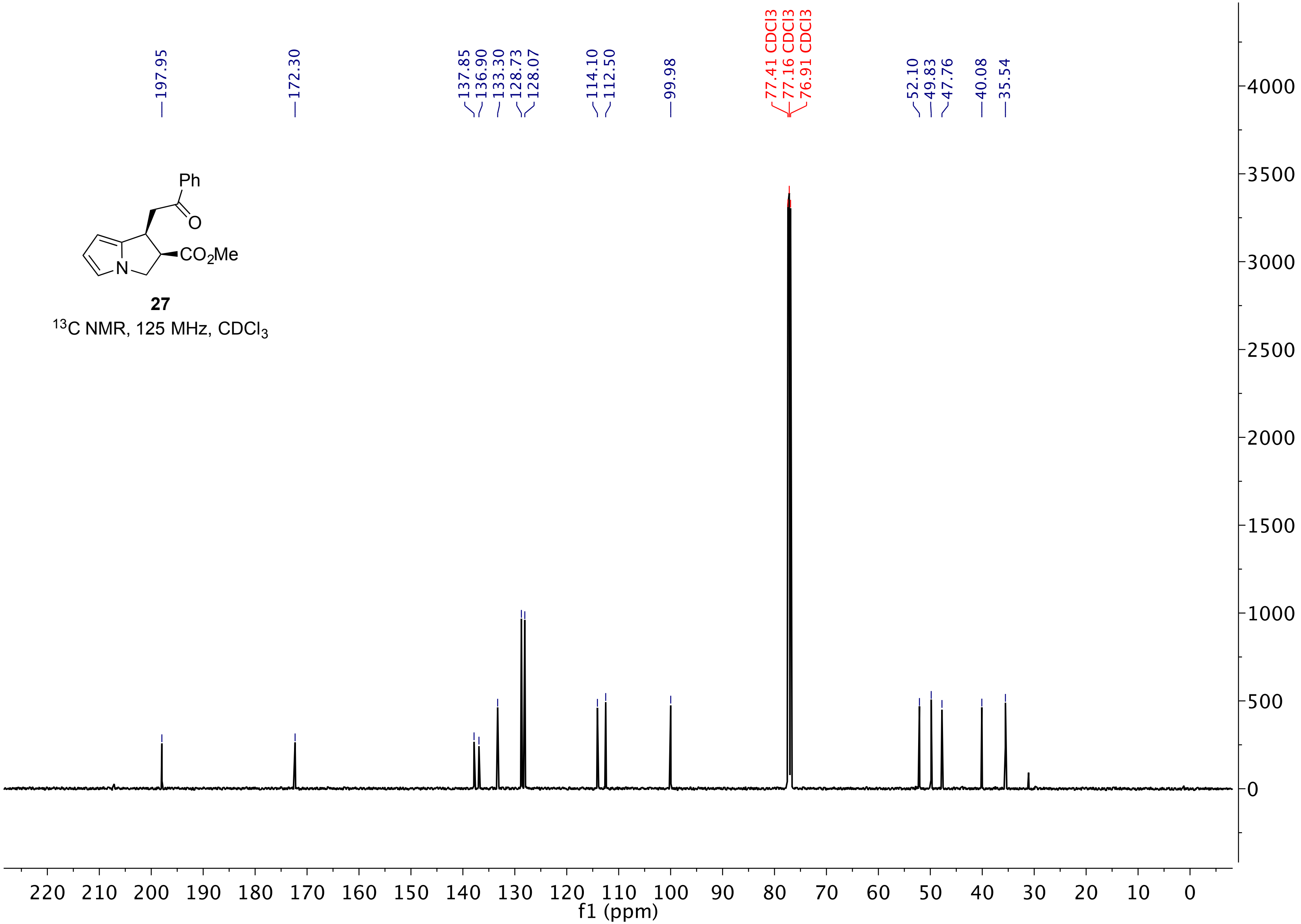

197.95
172.30
137.85
136.90
133.30
128.73
128.07
114.10
112.50
99.98
77.41 CDCl3
77.16 CDCl3
76.91 CDCl3
52.10
49.83
47.76
40.08
35.54
Ph
O
CO2Me
N
27
13C NMR, 125 MHz, CDCl3
f1 (ppm)

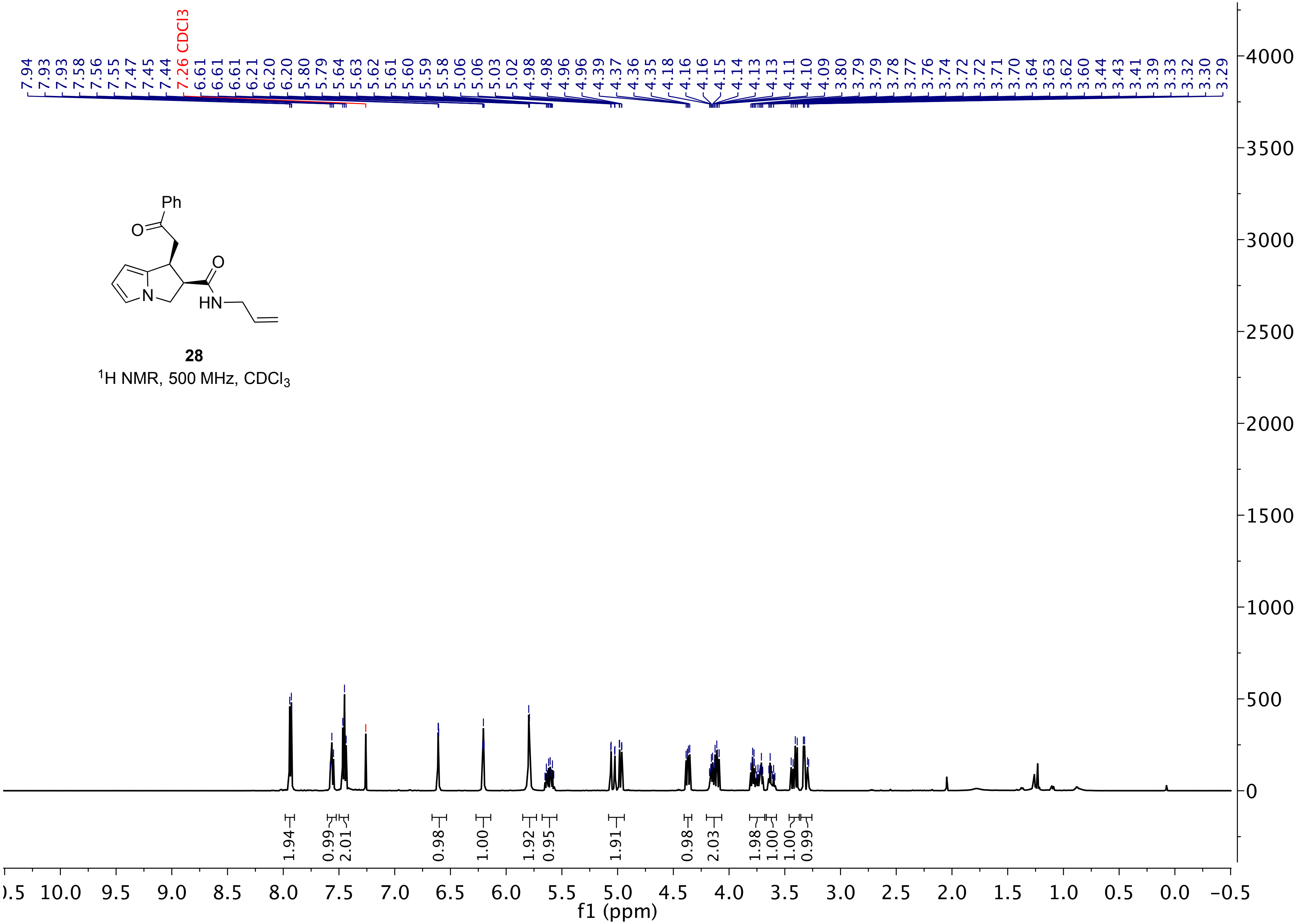

28

^{1}H NMR, 500 MHz, $CDCl_3$

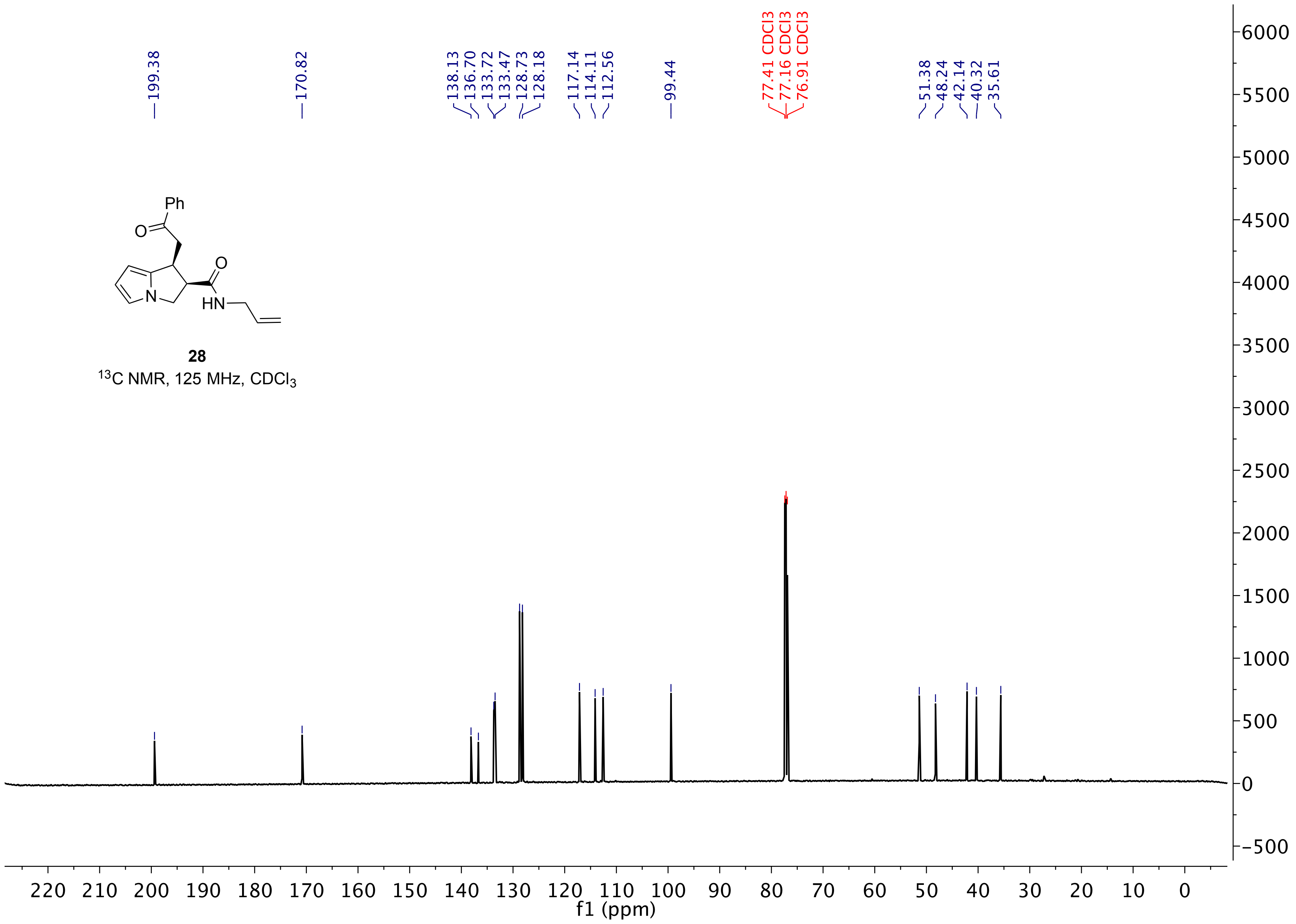

28

^{13}C NMR, 125 MHz, $CDCl_3$

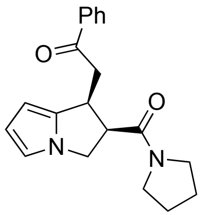

29

^{1}H NMR, 500 MHz, $CDCl_3$

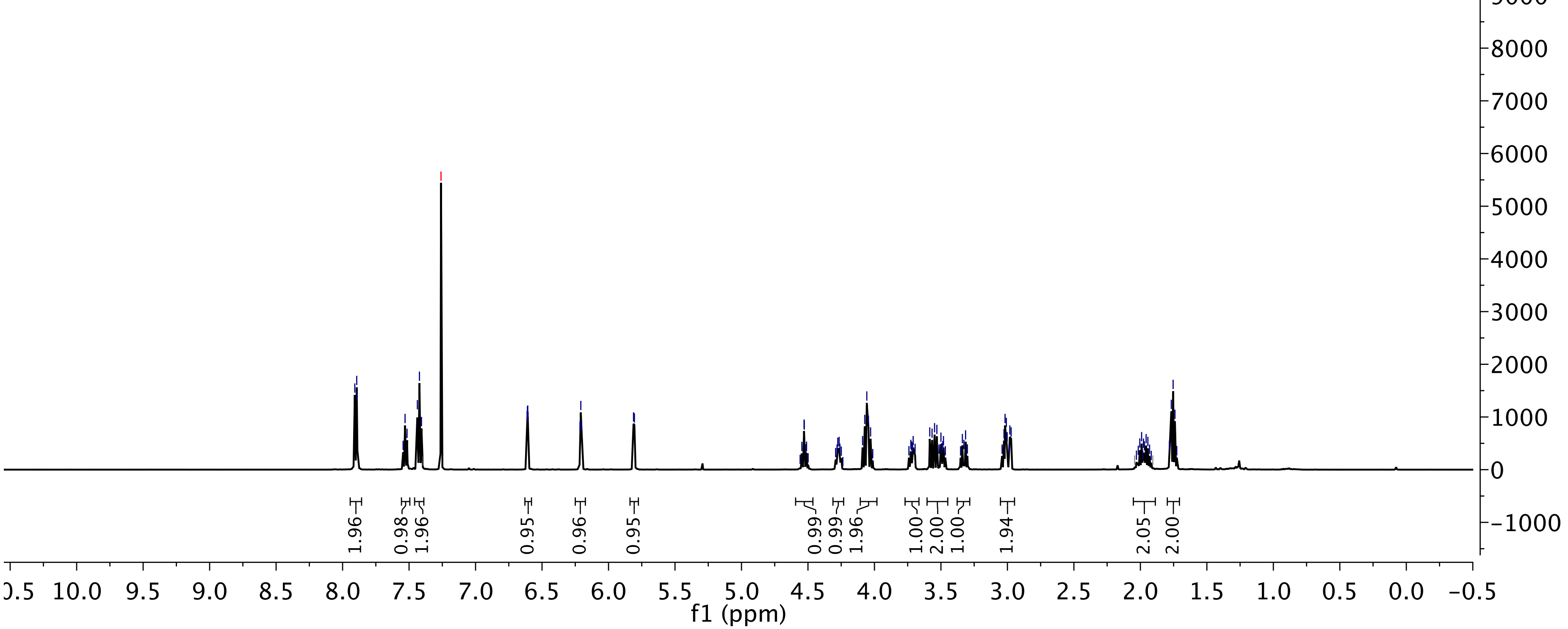

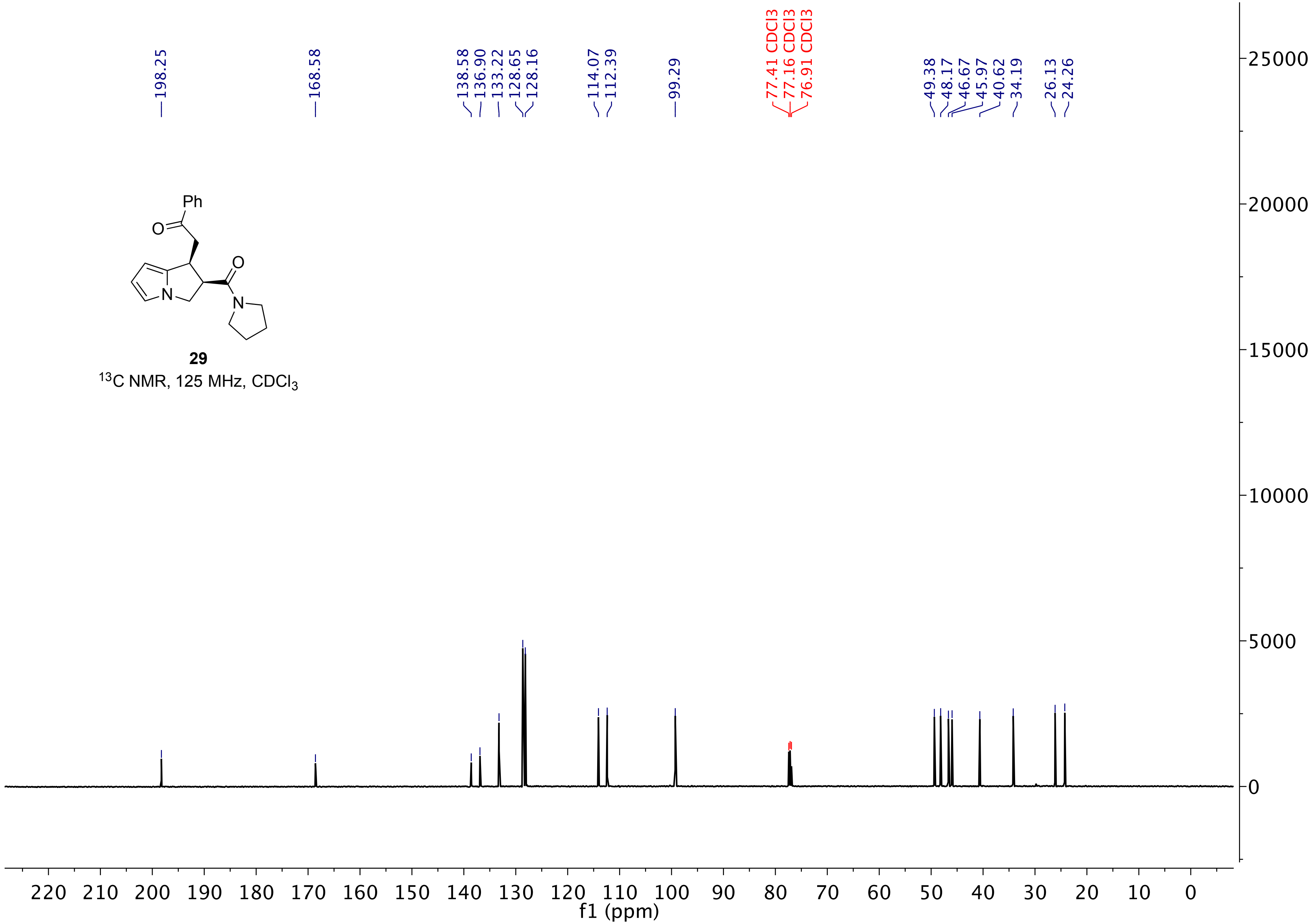
198.25
168.58
138.58
136.90
133.22
128.65
128.16
114.07
112.39
99.29
77.41 CDCl3
77.16 CDCl3
76.91 CDCl3
49.38
48.17
46.67
45.97
40.62
34.19
26.13
24.26
Ph
O
O
N
N
29
^{13}C NMR, 125 MHz, $CDCl_3$
25000
20000
15000
10000
5000
0
220 210 200 190 180 170 160 150 140 130 120 110 100 90 80 70 60 50 40 30 20 10 0
f1 (ppm)

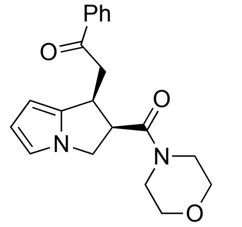

30

^{1}H NMR, 500 MHz, $CDCl_3$

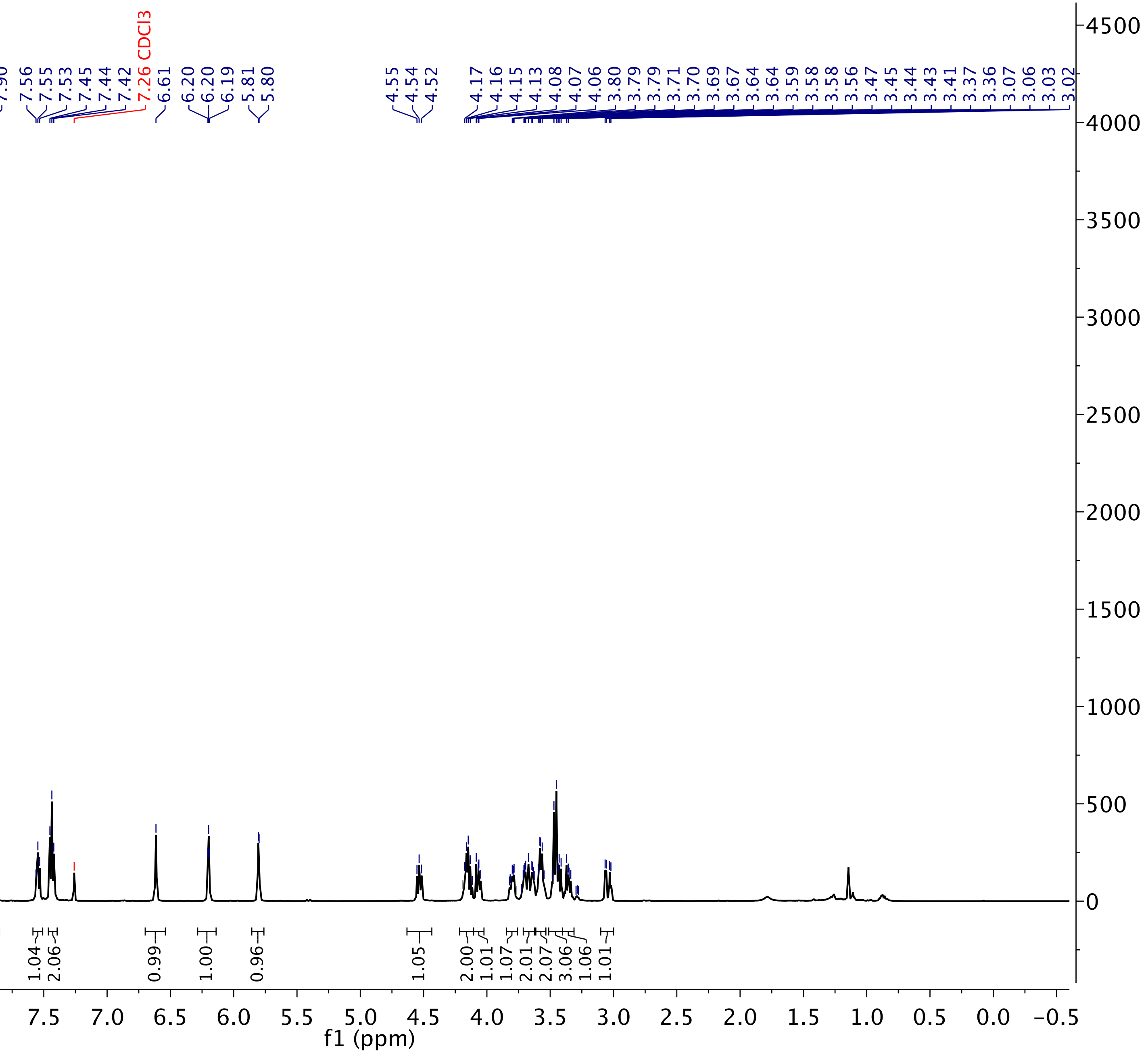

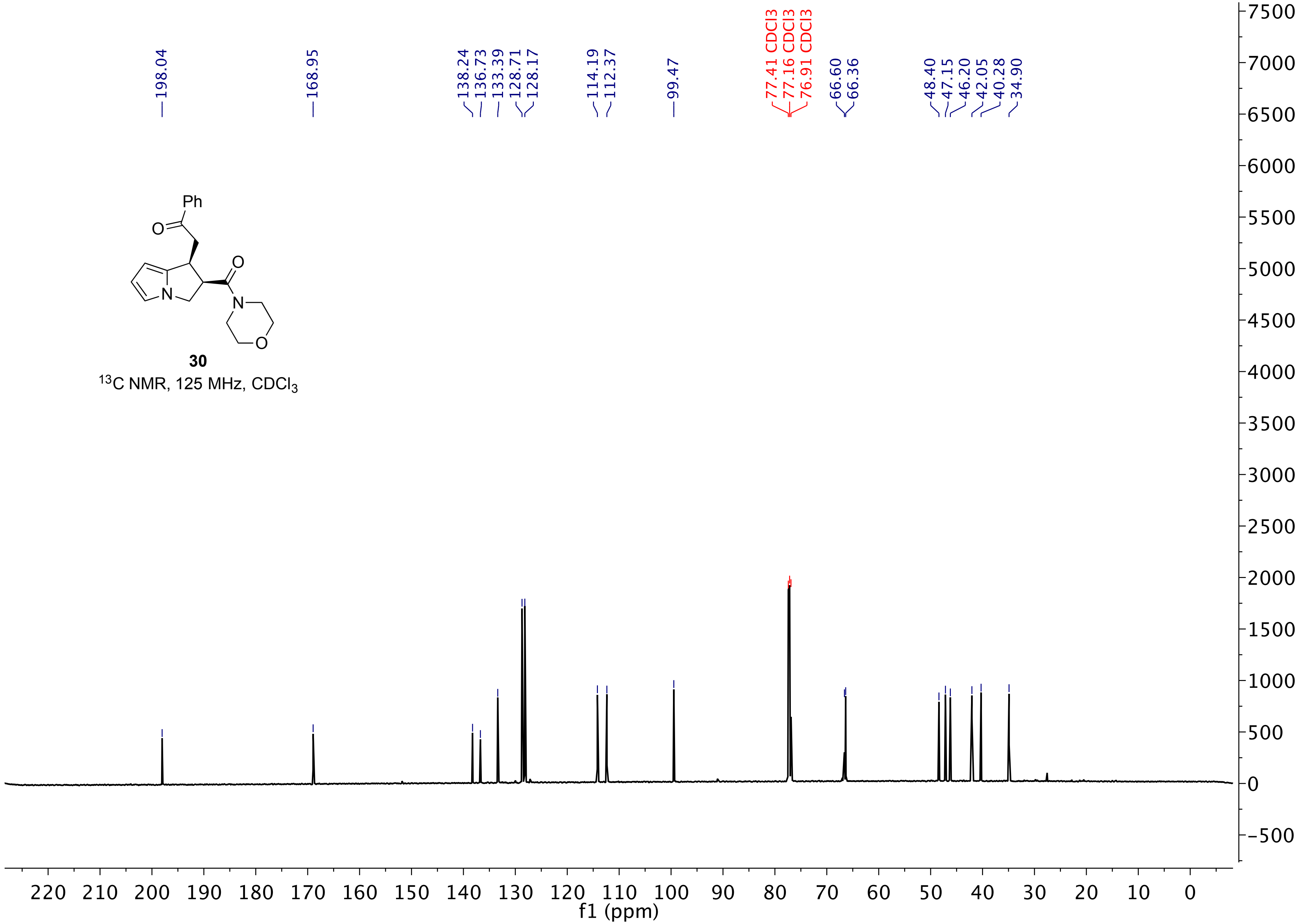

198.04
168.95
138.24
136.73
133.39
128.71
128.17
114.19
112.37
99.47
77.41 CDCl3
77.16 CDCl3
76.91 CDCl3
66.60
66.36
48.40
47.15
46.20
42.05
40.28
34.90
Ph
O
O
N
N
O
30
13C NMR, 125 MHz, CDCl3
f1 (ppm)

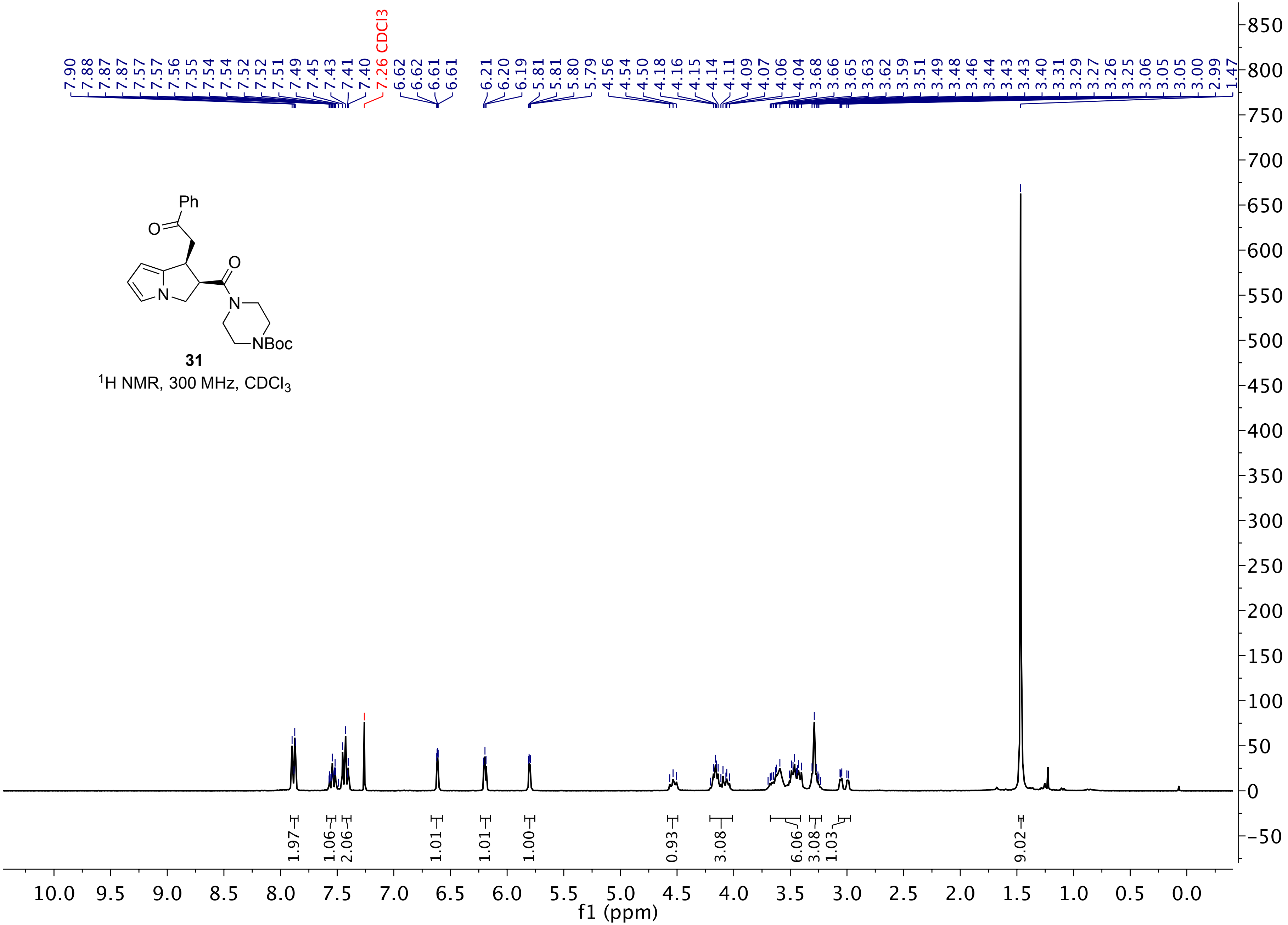

31

^1H NMR, 300 MHz, $CDCl_3$

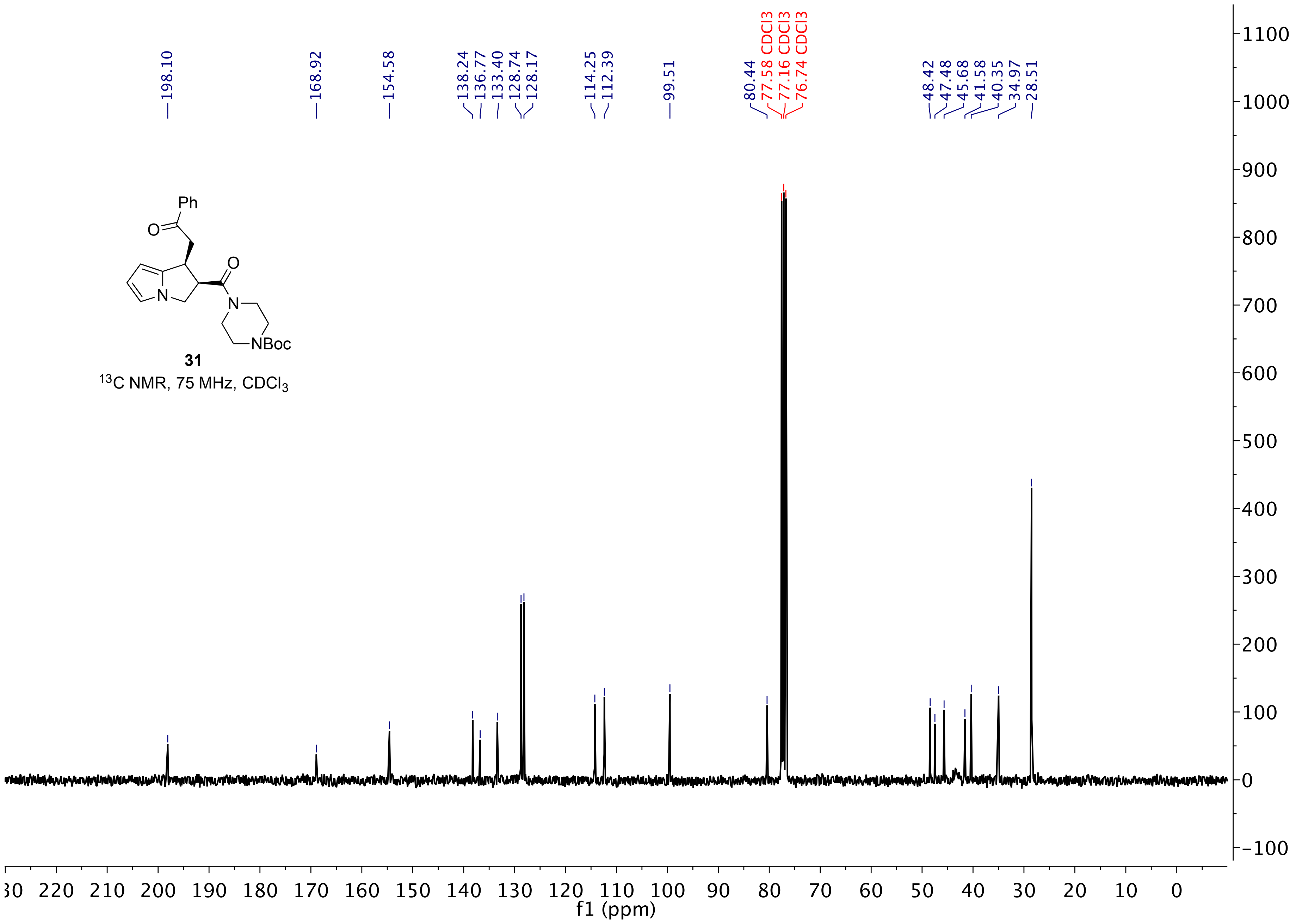
198.10
168.92
154.58
138.24
136.77
133.40
128.74
128.17
114.25
112.39
99.51
80.44
77.58 CDCl3
77.16 CDCl3
76.74 CDCl3
48.42
47.48
45.68
41.58
40.35
34.97
28.51
Ph
O
O
N
N
NBoc
31
13C NMR, 75 MHz, CDCl3
f1 (ppm)

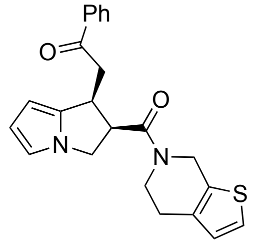

32

1:1 mixture of rotamers

^{1}H NMR, 500 MHz, $CDCl_3$

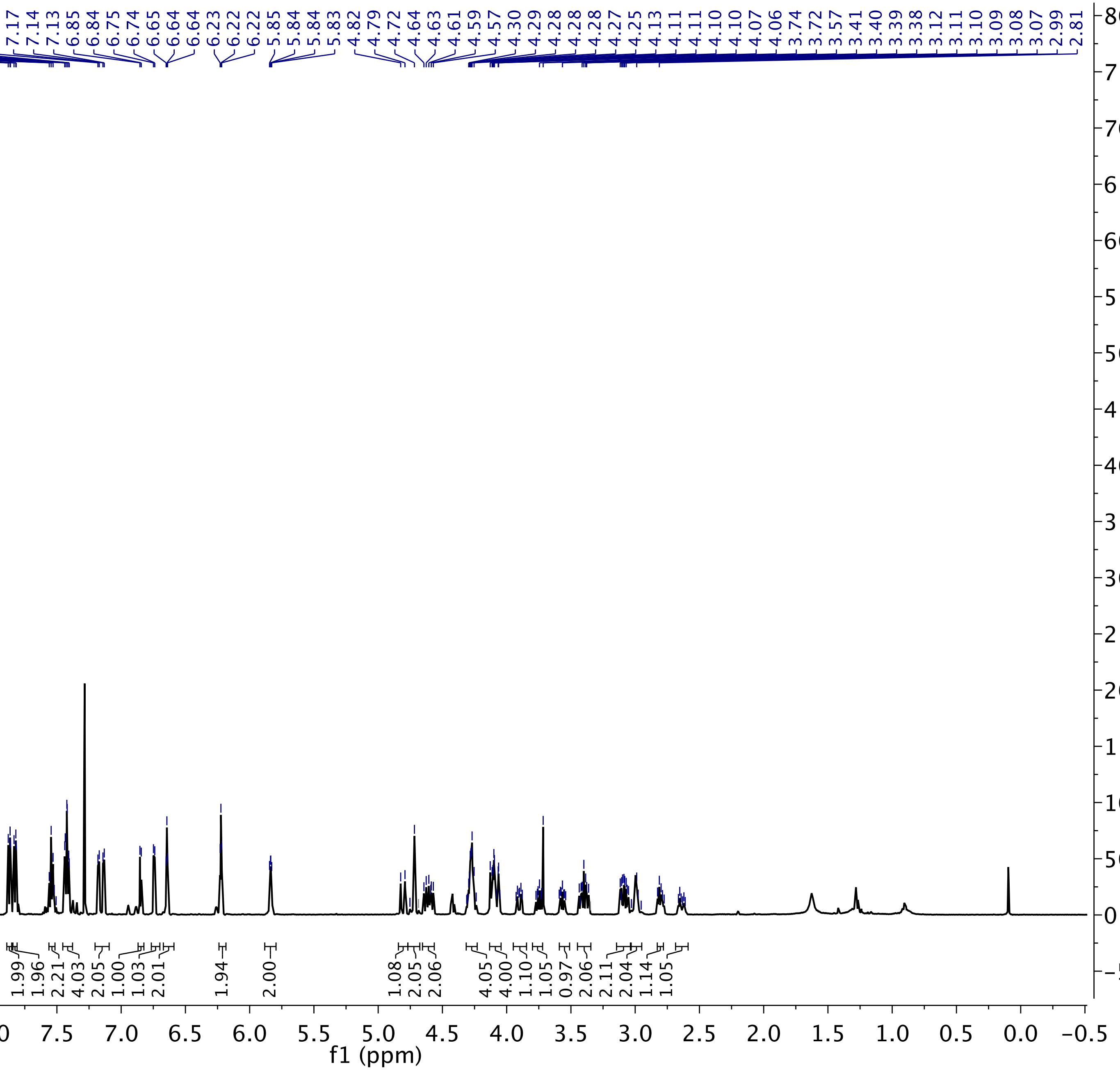

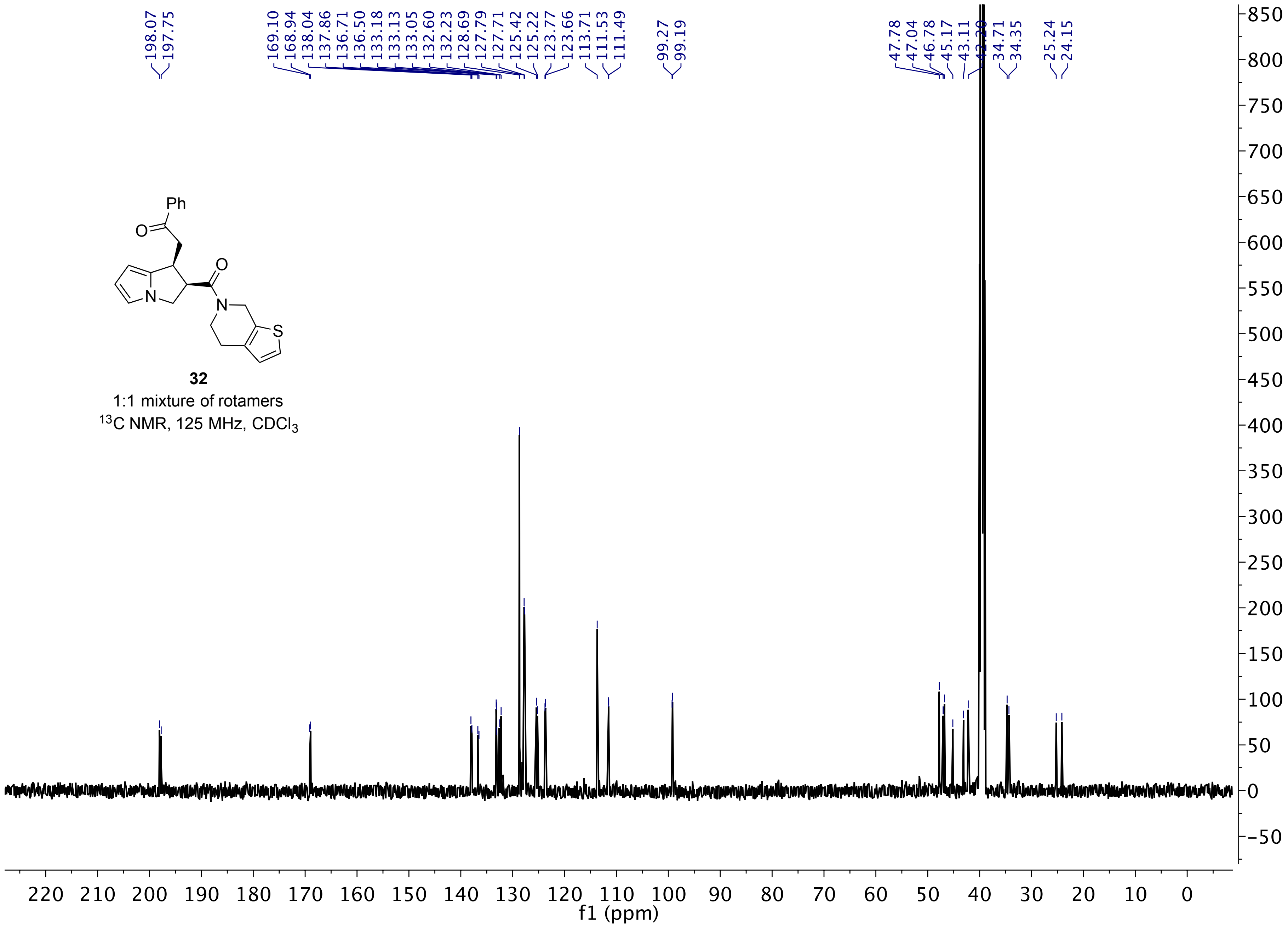

32

1:1 mixture of rotamers

^{13}C NMR, 125 MHz, $CDCl_3$

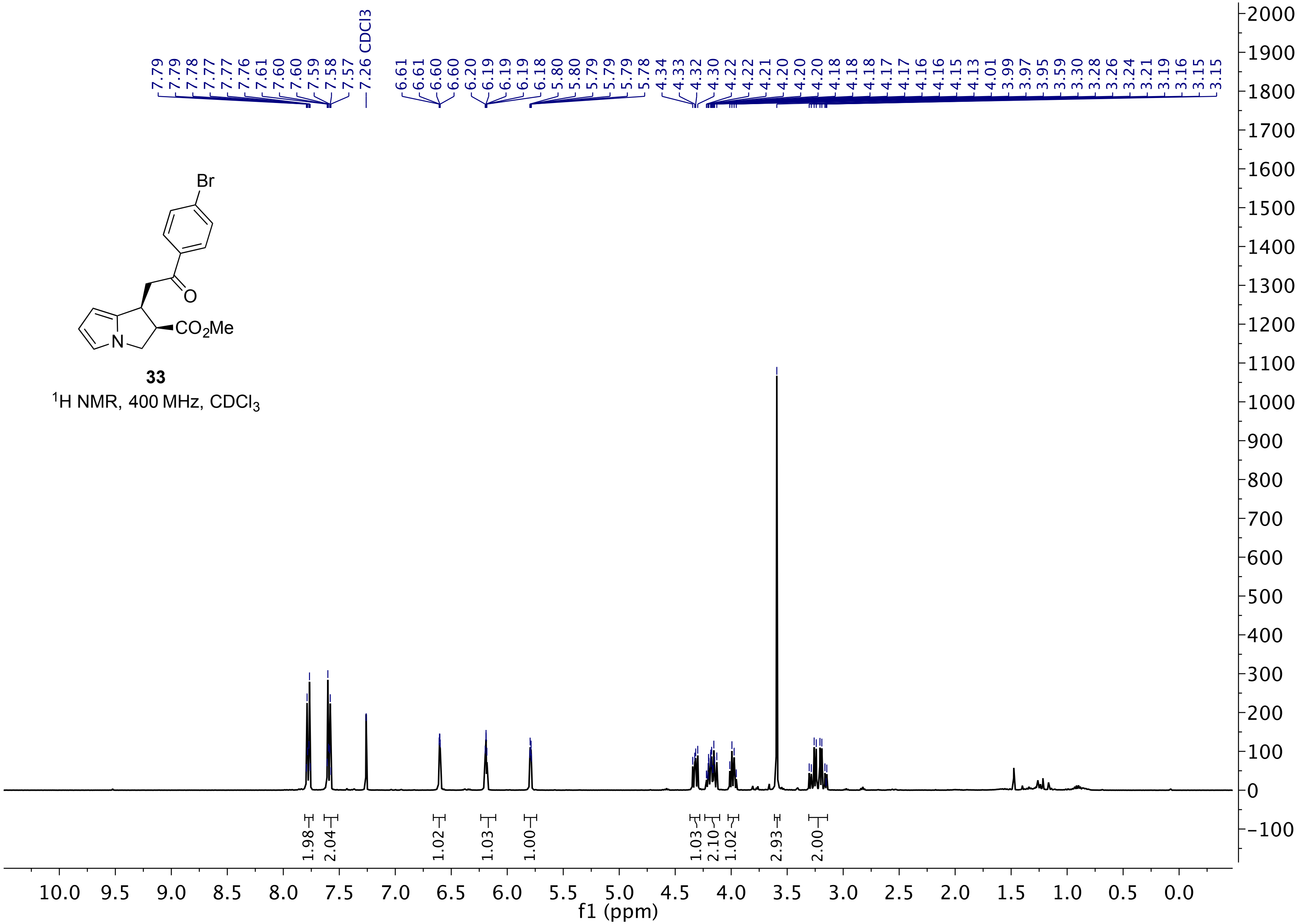
7.79 7.79 7.78 7.77 7.77 7.76 7.61 7.60 7.60 7.59 7.58 7.57
7.26 CDCl3
6.61 6.61 6.60 6.60 6.20 6.19 6.19 6.19 6.18 5.80 5.80 5.79 5.79 5.79 5.78
4.34 4.33 4.32 4.30 4.22 4.22 4.21 4.20 4.20 4.20 4.18 4.18 4.18 4.17 4.17 4.16 4.16 4.15 4.13 4.01 3.99 3.97 3.95 3.59 3.30 3.28 3.26 3.24 3.21 3.19 3.16 3.15 3.15
Br
O
CO2Me
N
33
1H NMR, 400 MHz, CDCl3
1.98 2.04 1.02 1.03 1.00 1.03 2.10 1.02 2.93 2.00
f1 (ppm)

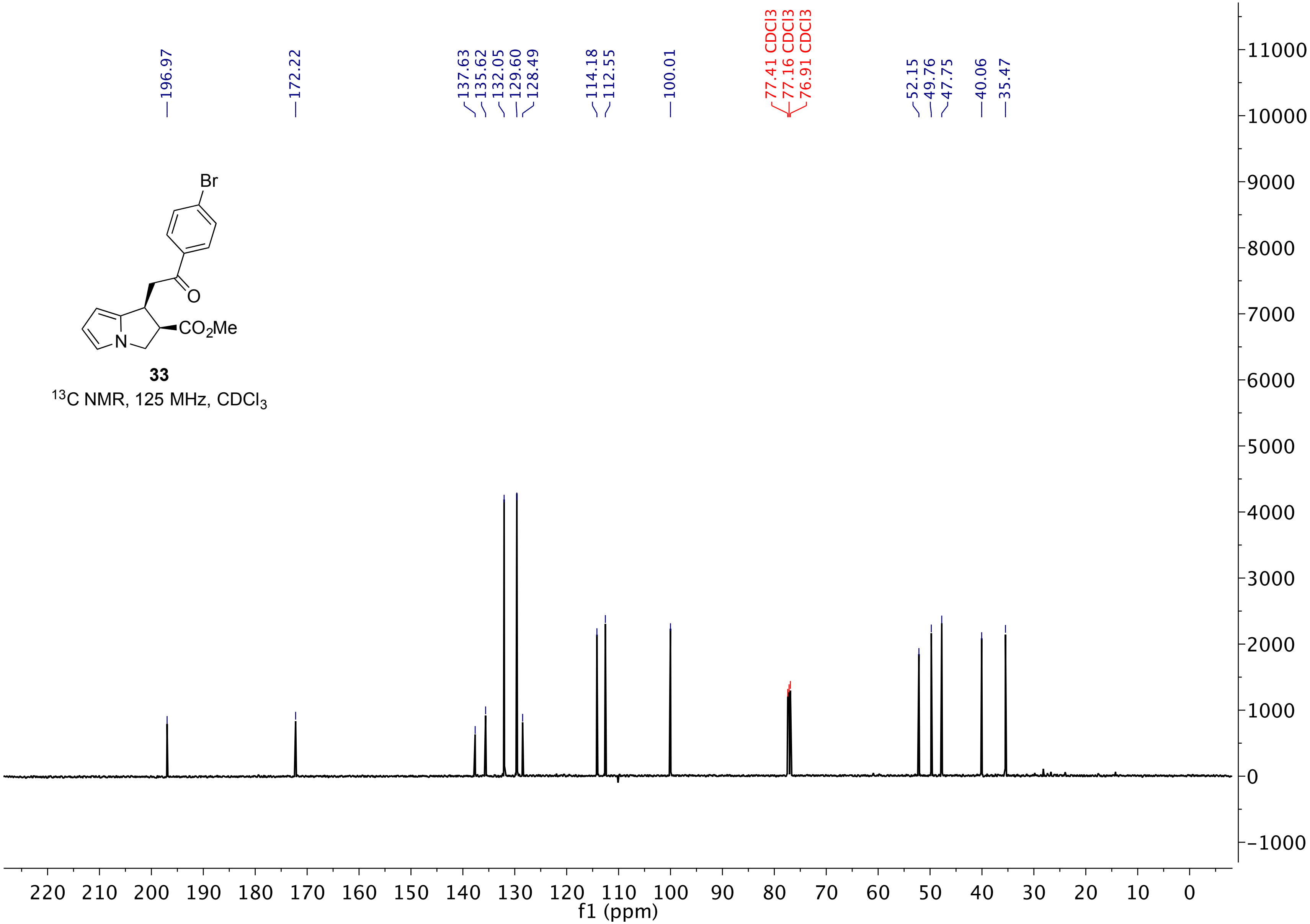

33

^{13}C NMR, 125 MHz, $CDCl_3$

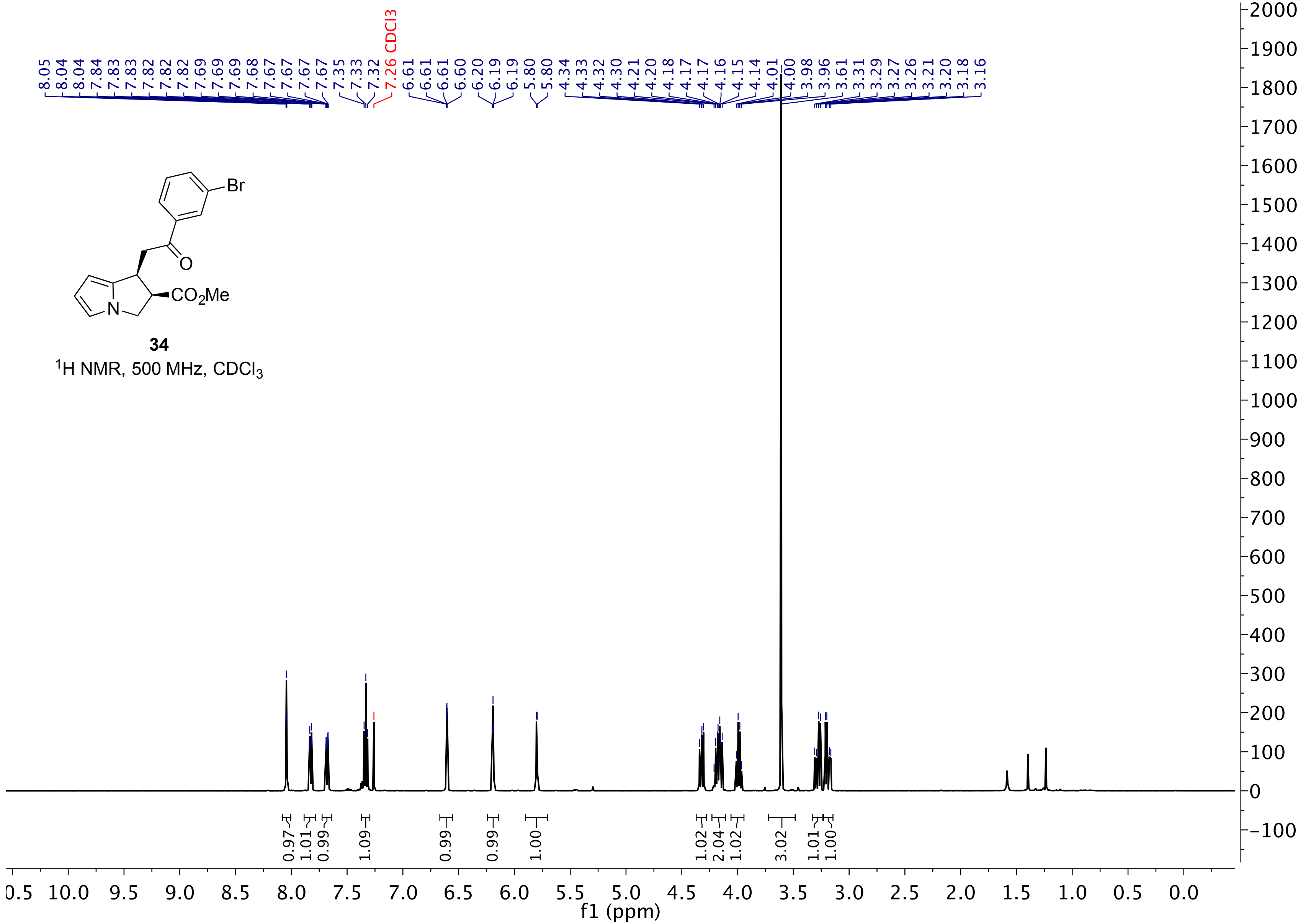

34

^{1}H NMR, 500 MHz, $CDCl_3$

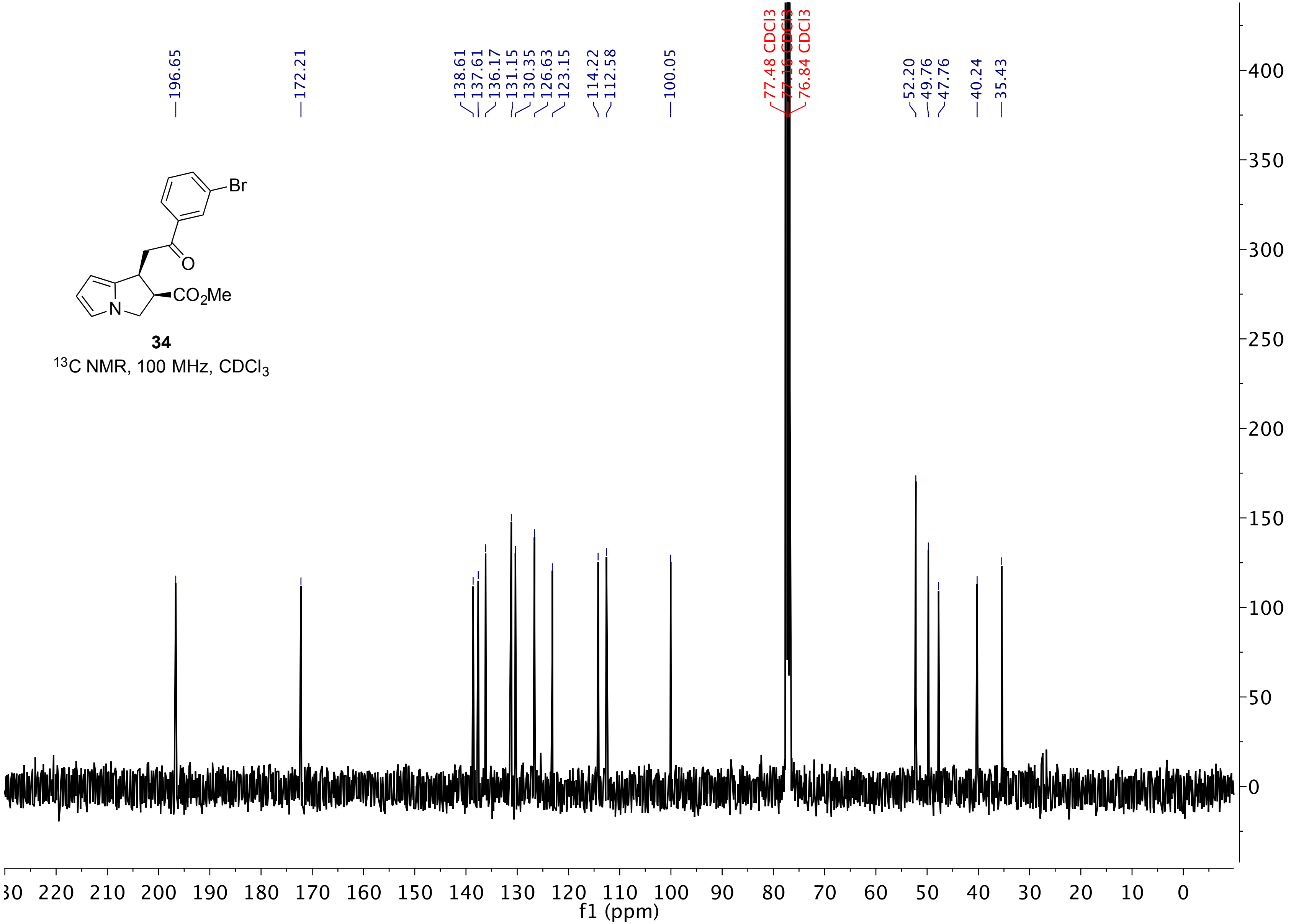

34

^{13}C NMR, 100 MHz, $CDCl_3$

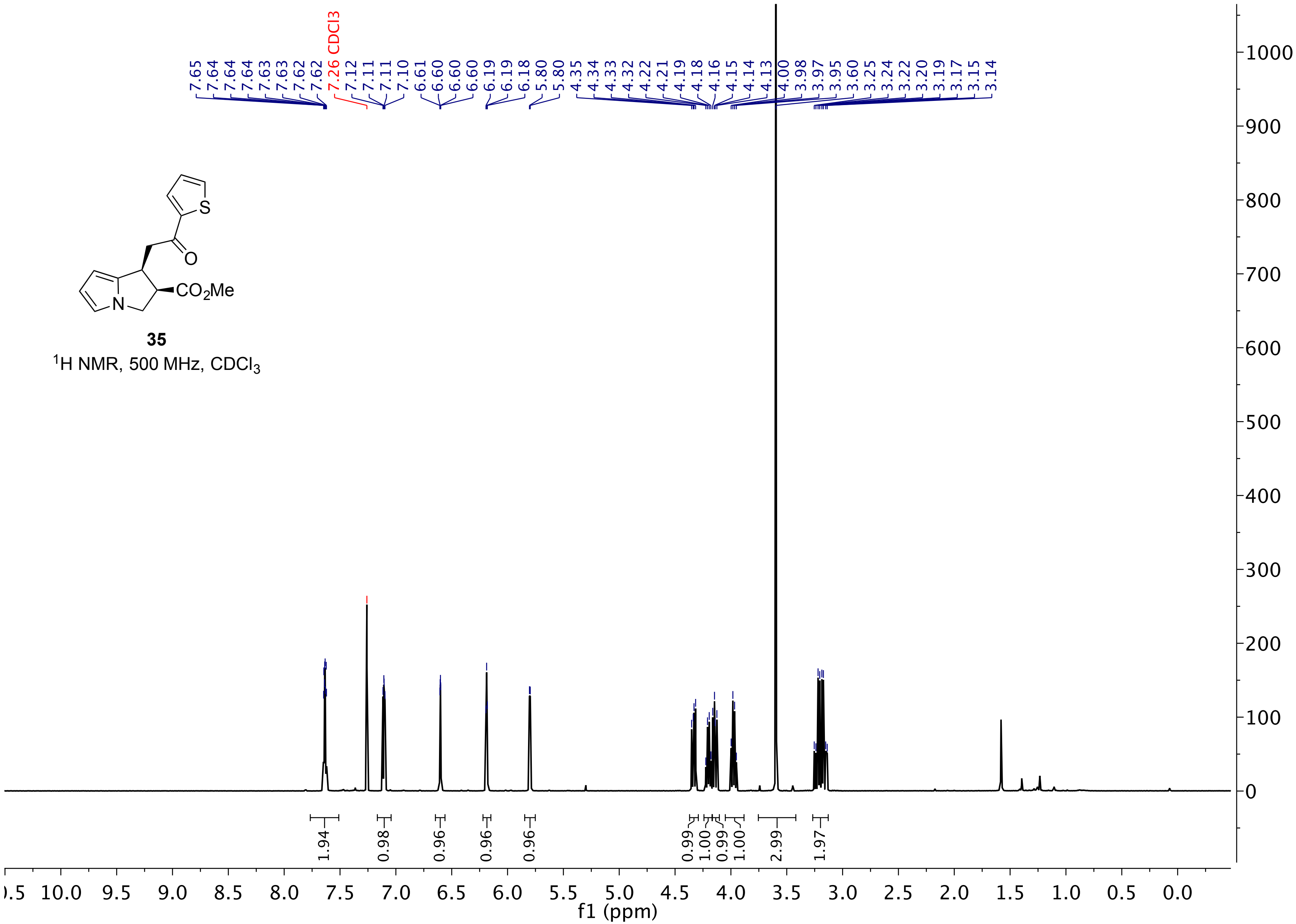

35
^{1}H NMR, 500 MHz, $CDCl_3$
7.65 7.64 7.64 7.64 7.63 7.63 7.62 7.62 7.26 CDCl3 7.12 7.11 7.11 7.10 6.61 6.60 6.60 6.60 6.19 6.19 6.18 5.80 5.80 4.35 4.34 4.33 4.32 4.22 4.21 4.19 4.18 4.16 4.15 4.14 4.13 4.00 3.98 3.97 3.95 3.60 3.25 3.24 3.22 3.20 3.19 3.17 3.15 3.14
1.94 0.98 0.96 0.96 0.96 0.99 1.00 0.99 1.00 2.99 1.97
f1 (ppm)

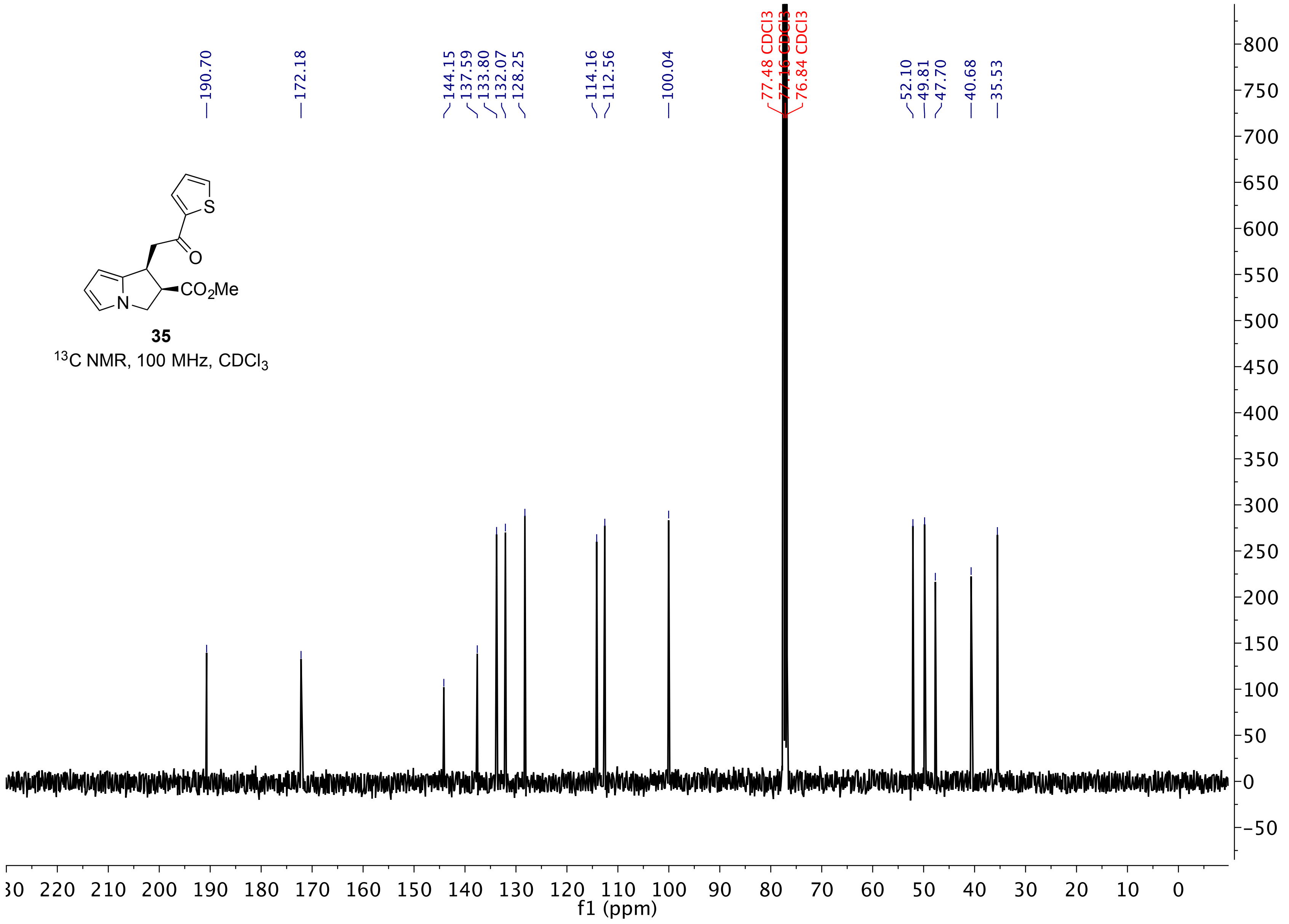
190.70
172.18
144.15
137.59
133.80
132.07
128.25
114.16
112.56
100.04
77.48 CDCl3
77.16 CDCl3
76.84 CDCl3
52.10
49.81
47.70
40.68
35.53
S
O
N
CO2Me
35
13C NMR, 100 MHz, CDCl3
f1 (ppm)

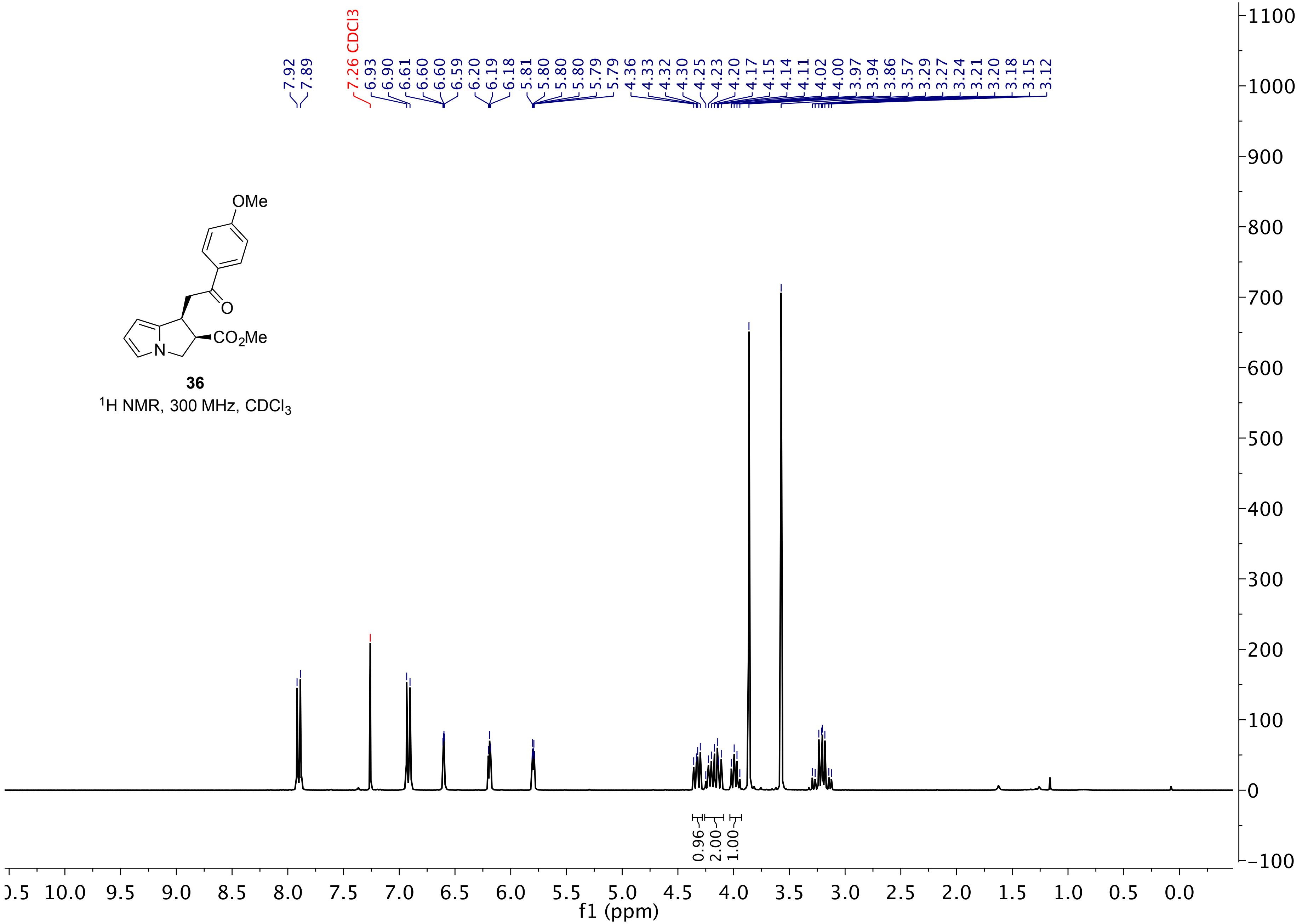

7.92
7.89
7.26 CDCl3
6.93
6.90
6.61
6.60
6.60
6.59
6.20
6.19
6.18
5.81
5.80
5.80
5.80
5.79
5.79
4.36
4.33
4.32
4.30
4.25
4.23
4.20
4.17
4.15
4.14
4.11
4.02
4.00
3.97
3.94
3.86
3.57
3.29
3.27
3.24
3.21
3.20
3.18
3.15
3.12
OMe
O
CO2Me
N
36
1H NMR, 300 MHz, CDCl3
0.96
2.00
1.00
f1 (ppm)

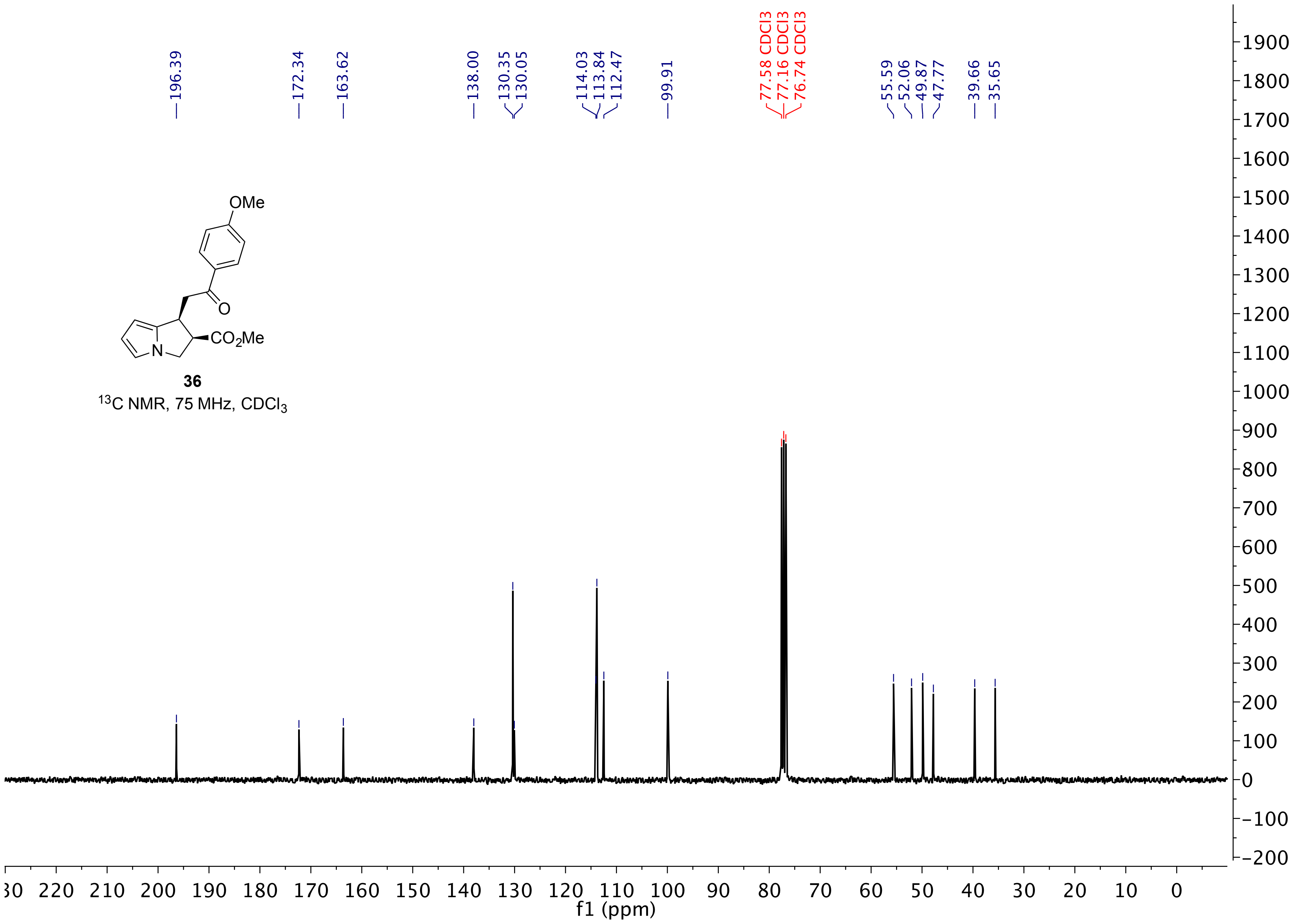

36

^{13}C NMR, 75 MHz, $CDCl_3$

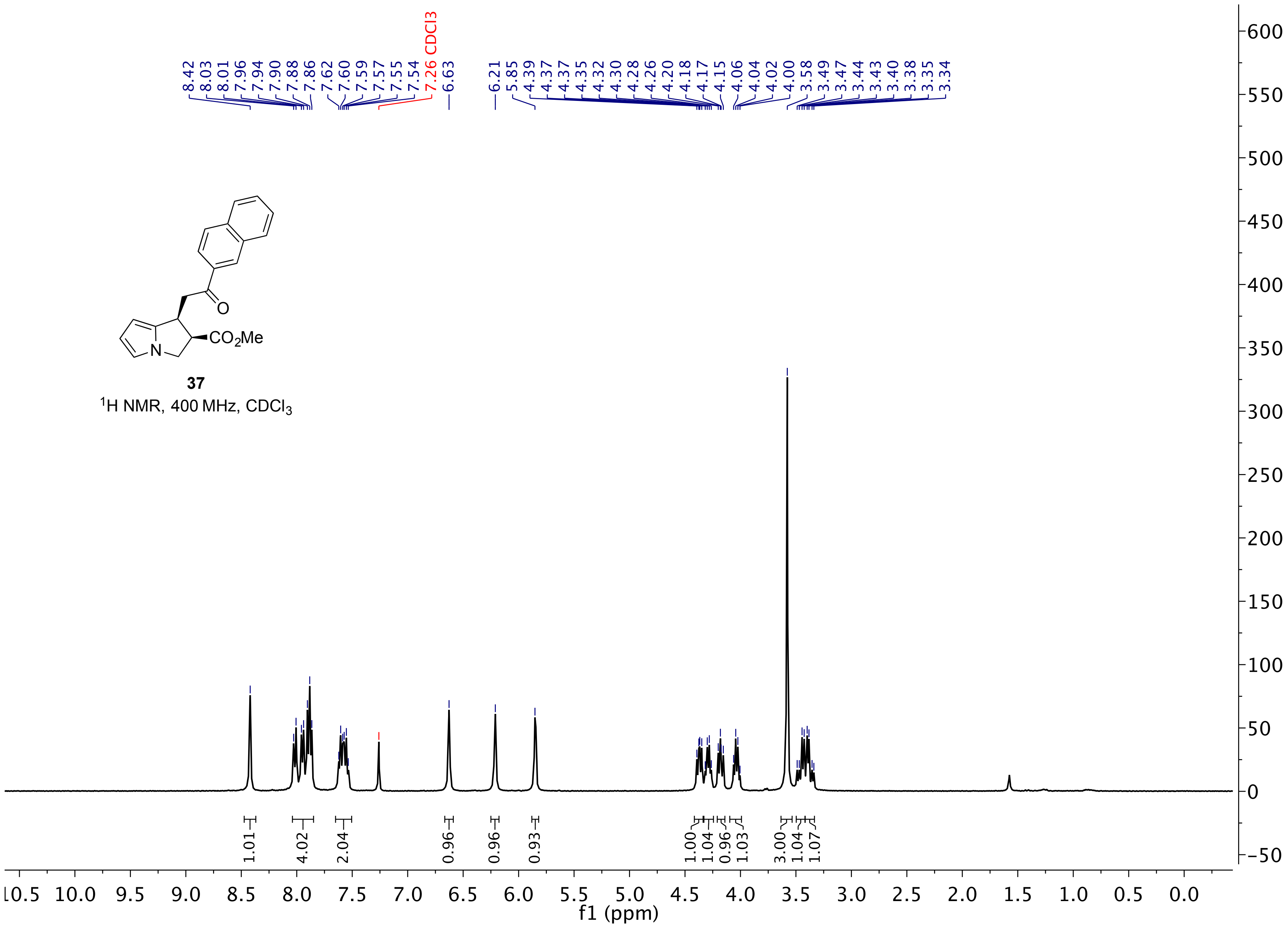
37
¹H NMR, 400 MHz, CDCl3
7.26 CDCl3
f1 (ppm)

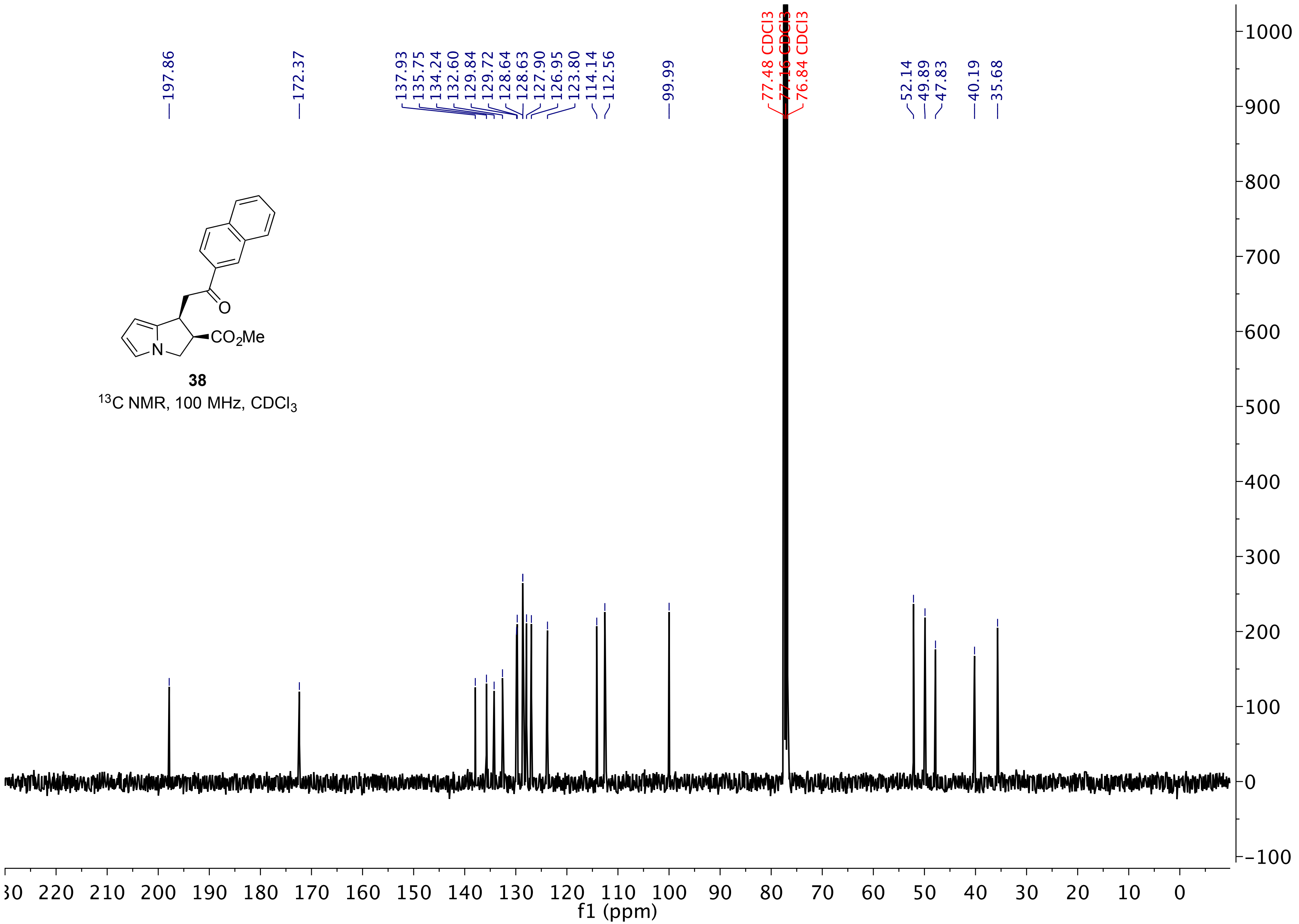

38

^{13}C NMR, 100 MHz, $CDCl_3$

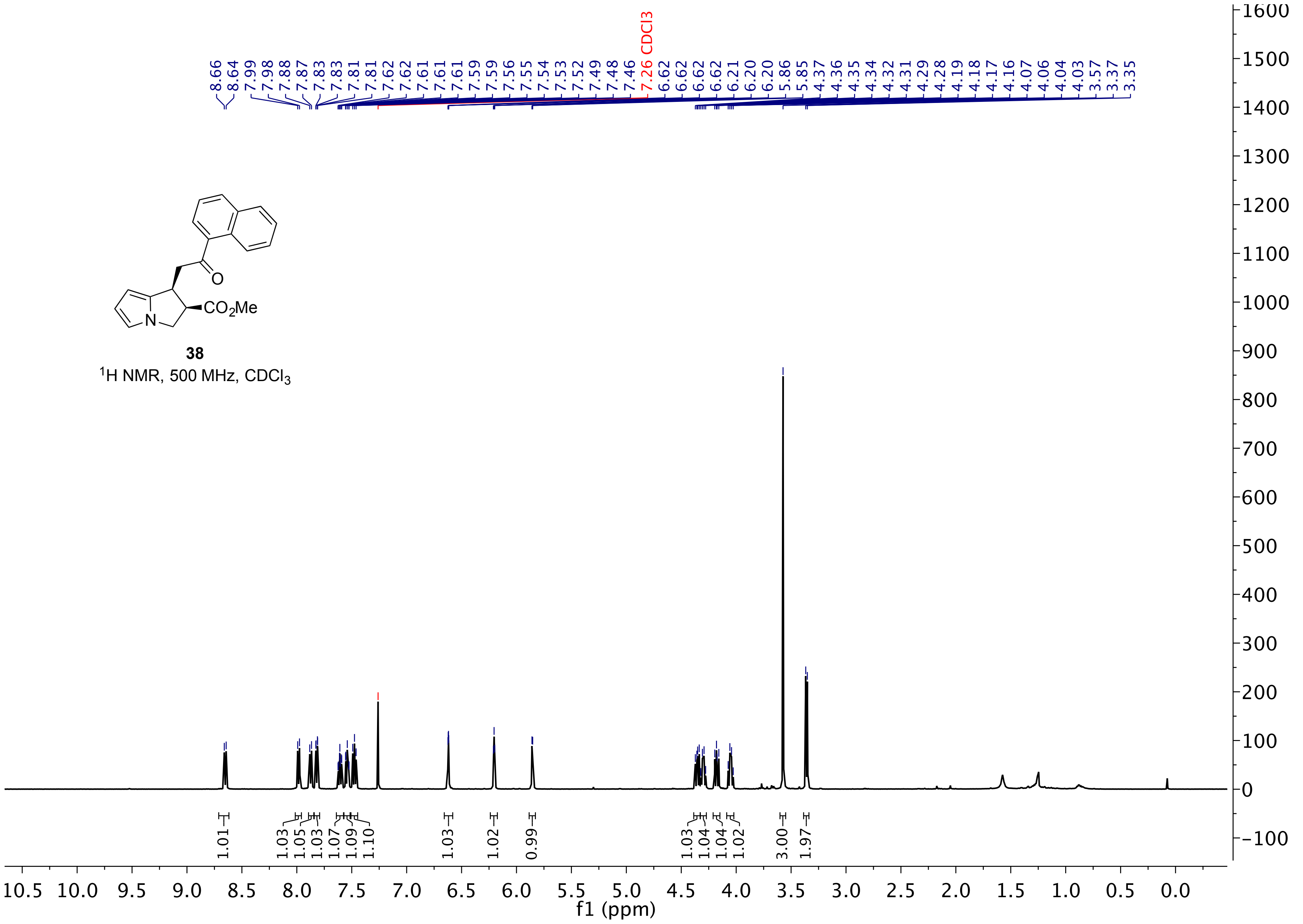

38

^{1}H NMR, 500 MHz, $CDCl_3$

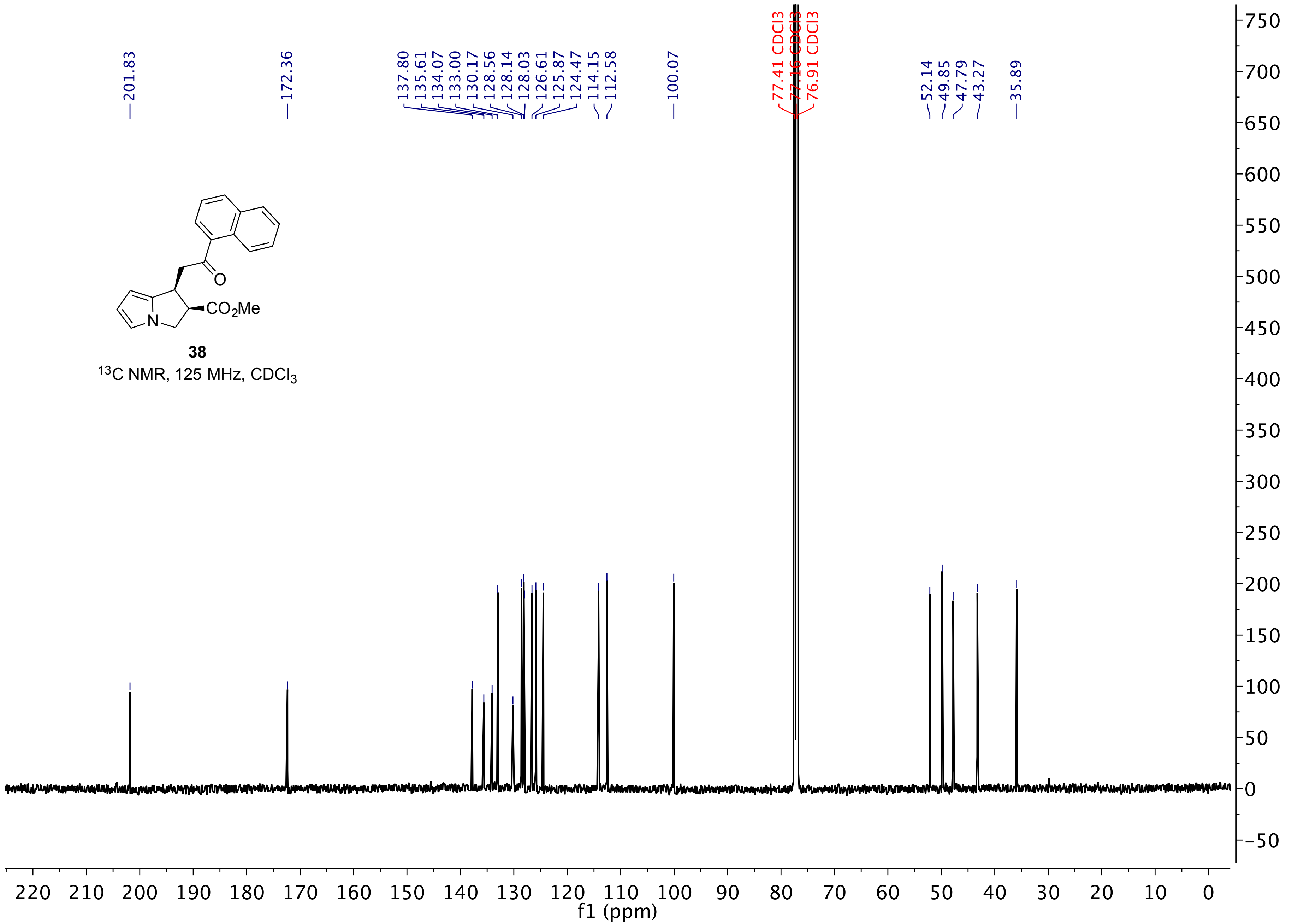

38

^{13}C NMR, 125 MHz, $CDCl_3$

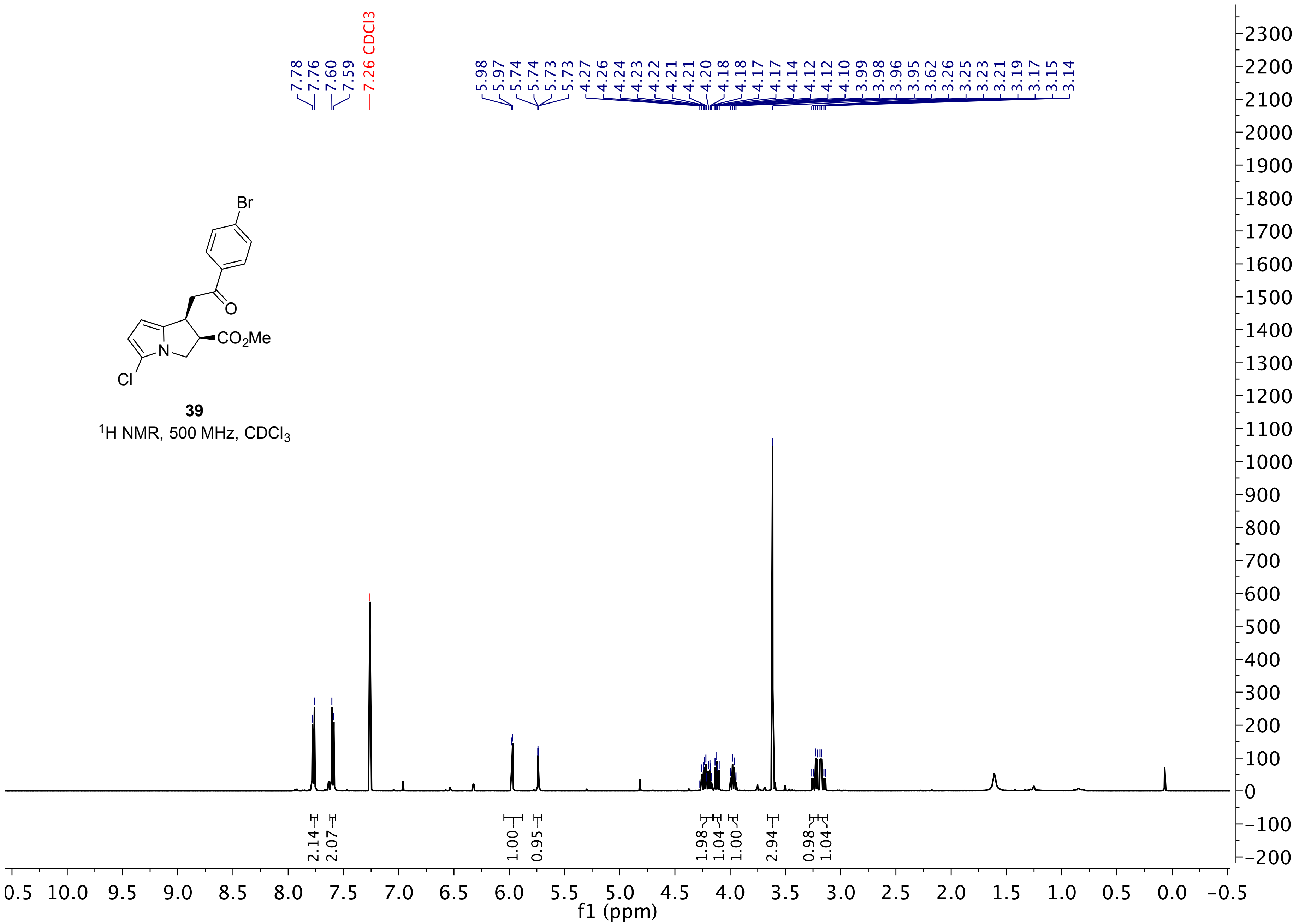
39
^{1}H NMR, 500 MHz, $CDCl_3$
7.78
7.76
7.60
7.59
7.26 CDCl3
5.98
5.97
5.74
5.74
5.73
5.73
4.27
4.26
4.24
4.23
4.22
4.21
4.21
4.20
4.18
4.18
4.17
4.17
4.14
4.12
4.12
4.10
3.99
3.98
3.96
3.95
3.62
3.26
3.25
3.23
3.21
3.19
3.17
3.15
3.14
2.14
2.07
1.00
0.95
1.98
1.04
1.00
2.94
0.98
1.04
f1 (ppm)

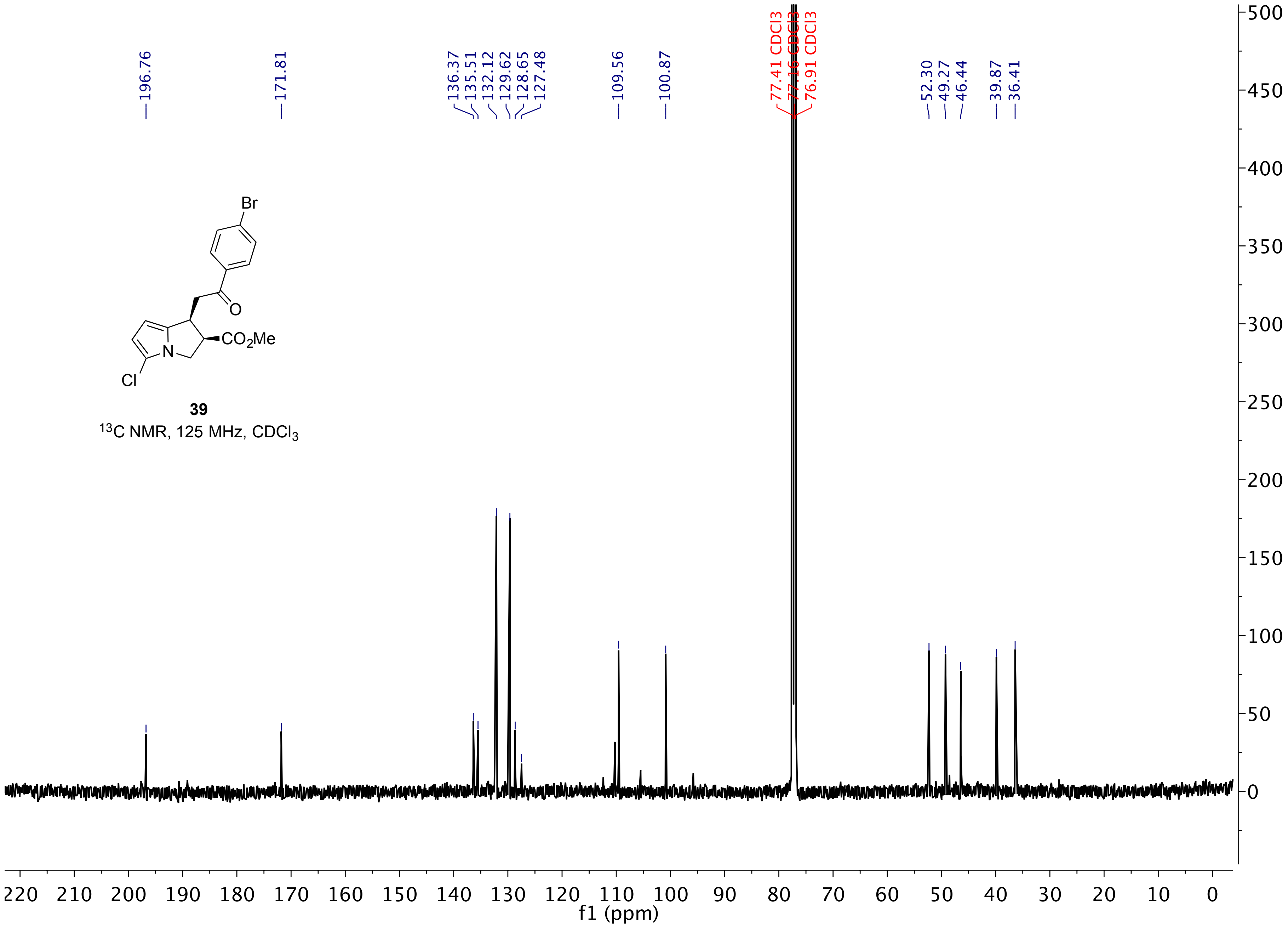

39

^{13}C NMR, 125 MHz, $CDCl_3$

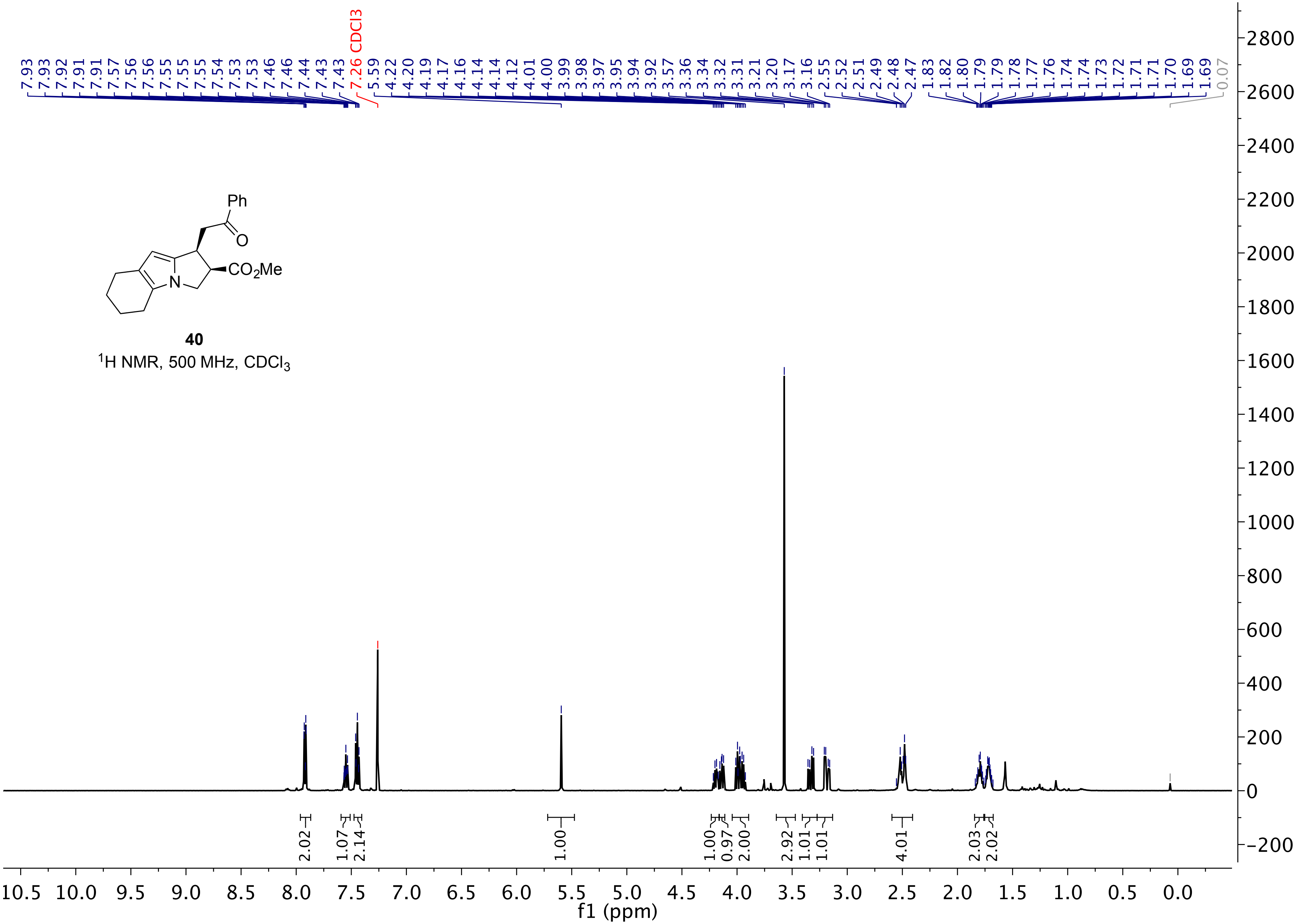

40

^{1}H NMR, 500 MHz, $CDCl_3$

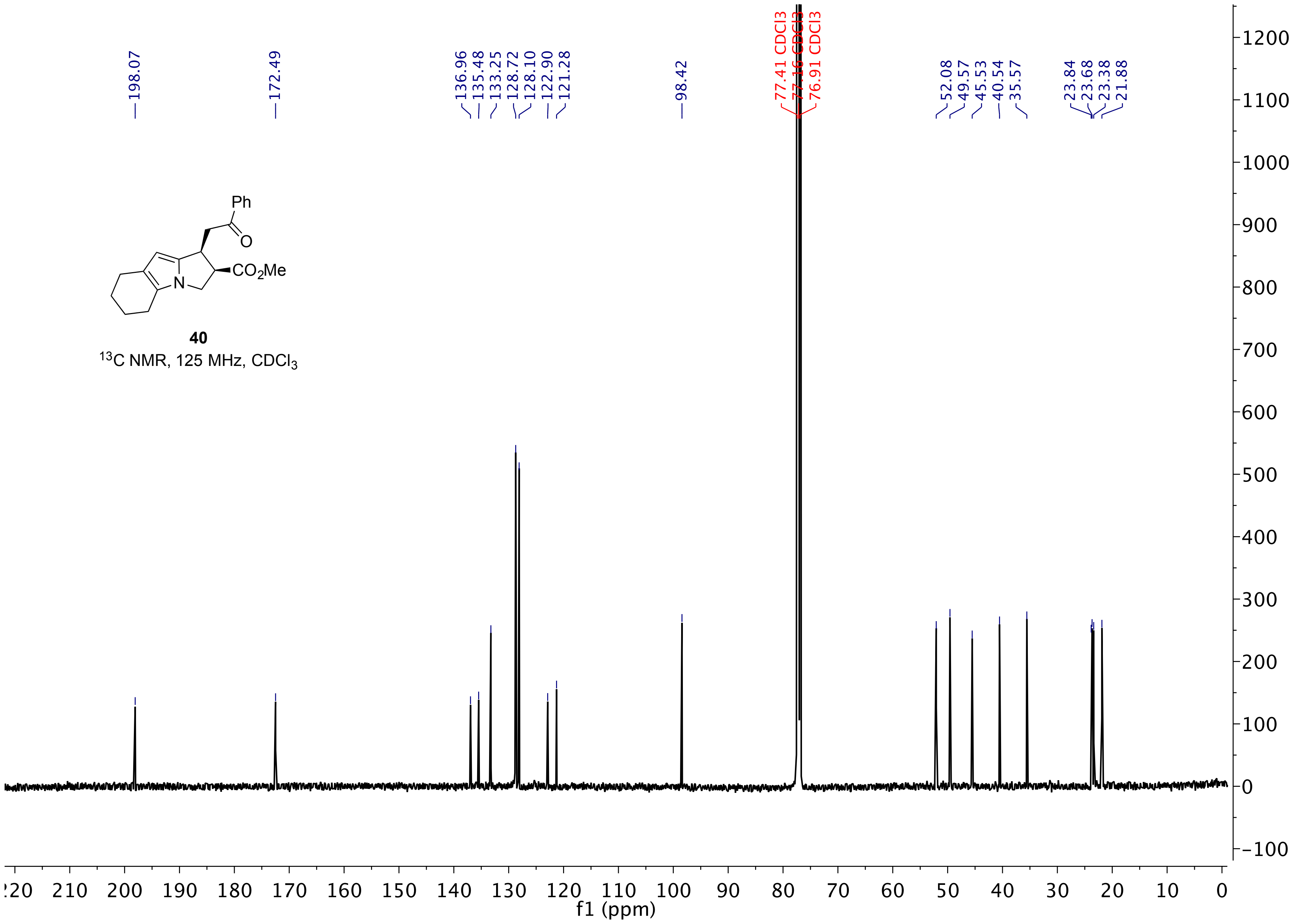
198.07
172.49
136.96
135.48
133.25
128.72
128.10
122.90
121.28
98.42
77.41 CDCl3
77.16 CDCl3
76.91 CDCl3
52.08
49.57
45.53
40.54
35.57
23.84
23.68
23.38
21.88
Ph
O
CO2Me
N
40
13C NMR, 125 MHz, CDCl3
f1 (ppm)

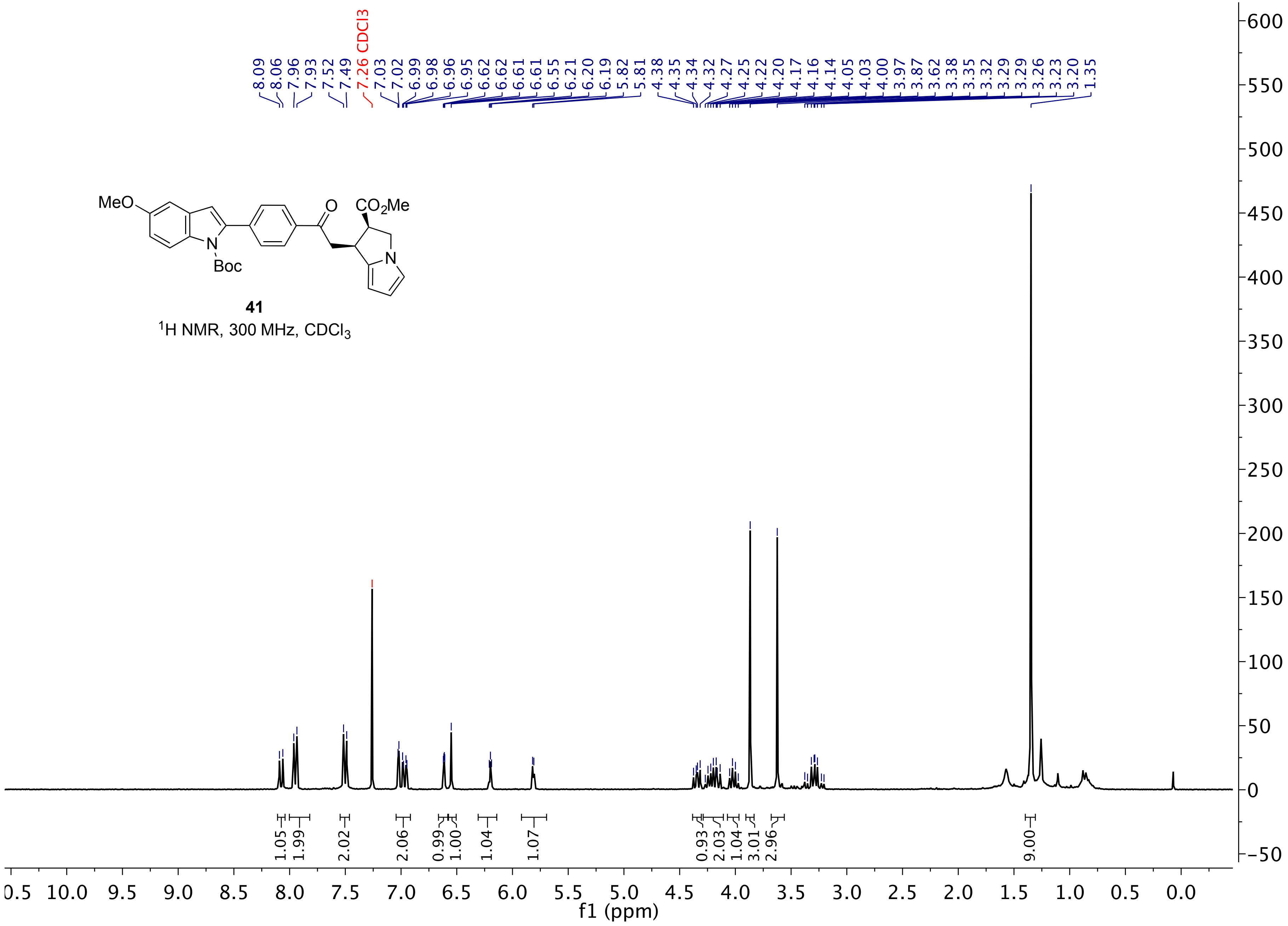

MeO
N
Boc
O
CO_2Me
N
41
^1H NMR, 300 MHz, $CDCl_3$
8.09 8.06 7.96 7.93 7.52 7.49 7.26 CDCl3 7.03 7.02 6.99 6.98 6.96 6.95 6.62 6.62 6.61 6.61 6.55 6.21 6.20 6.19 5.82 5.81 4.38 4.35 4.34 4.32 4.27 4.25 4.22 4.20 4.17 4.16 4.14 4.05 4.03 4.00 3.97 3.87 3.62 3.38 3.35 3.32 3.29 3.29 3.26 3.23 3.20 1.35
1.05 1.99 2.02 2.06 0.99 1.00 1.04 1.07 0.93 2.03 1.04 3.01 2.96 9.00
f1 (ppm)

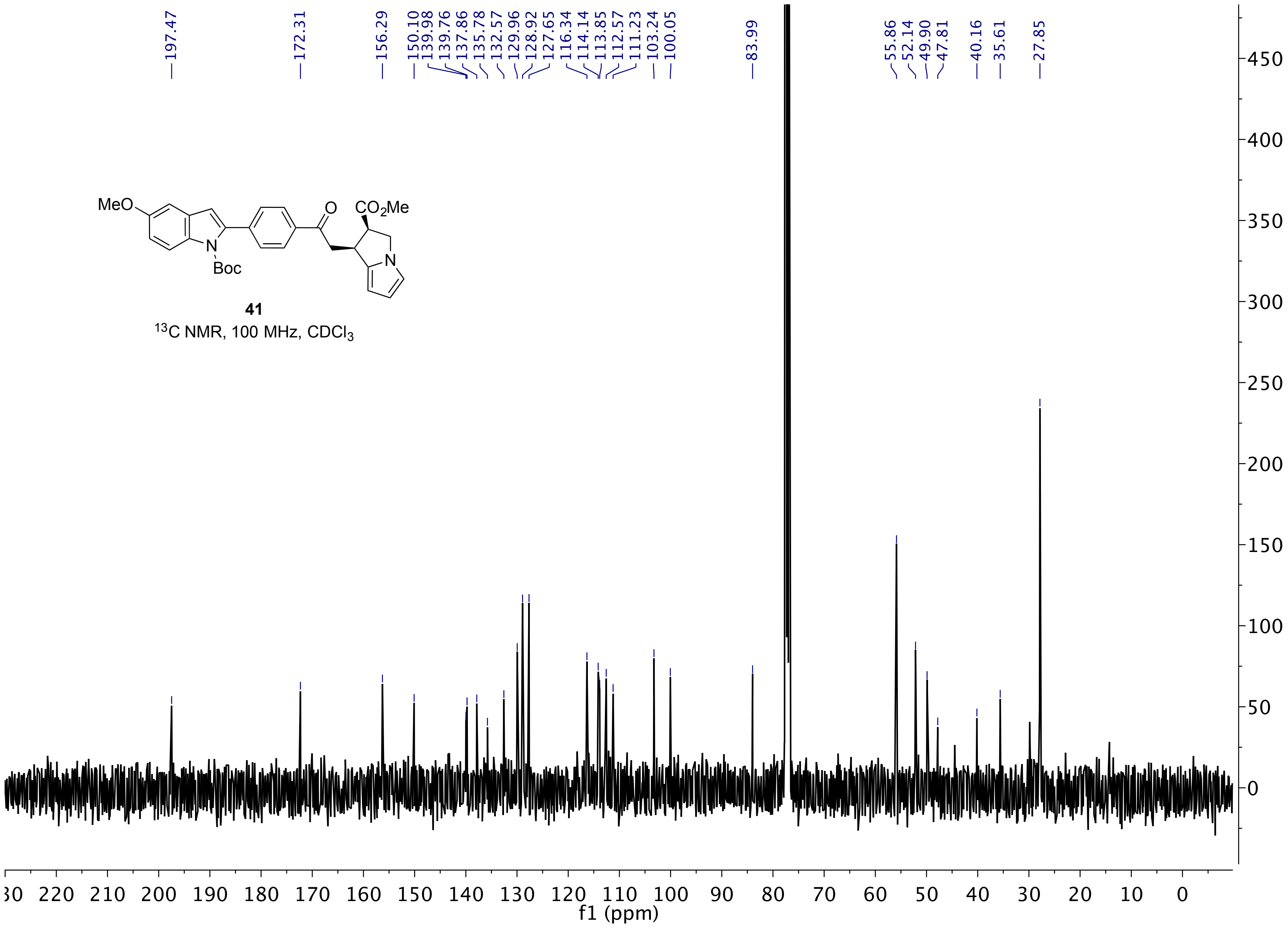

197.47
172.31
156.29
150.10
139.98
139.76
137.86
135.78
132.57
129.96
128.92
127.65
116.34
114.14
113.85
112.57
111.23
103.24
100.05
83.99
55.86
52.14
49.90
47.81
40.16
35.61
27.85
MeO
O
CO_2Me
N
Boc
N
41
^{13}C NMR, 100 MHz, $CDCl_3$
f1 (ppm)

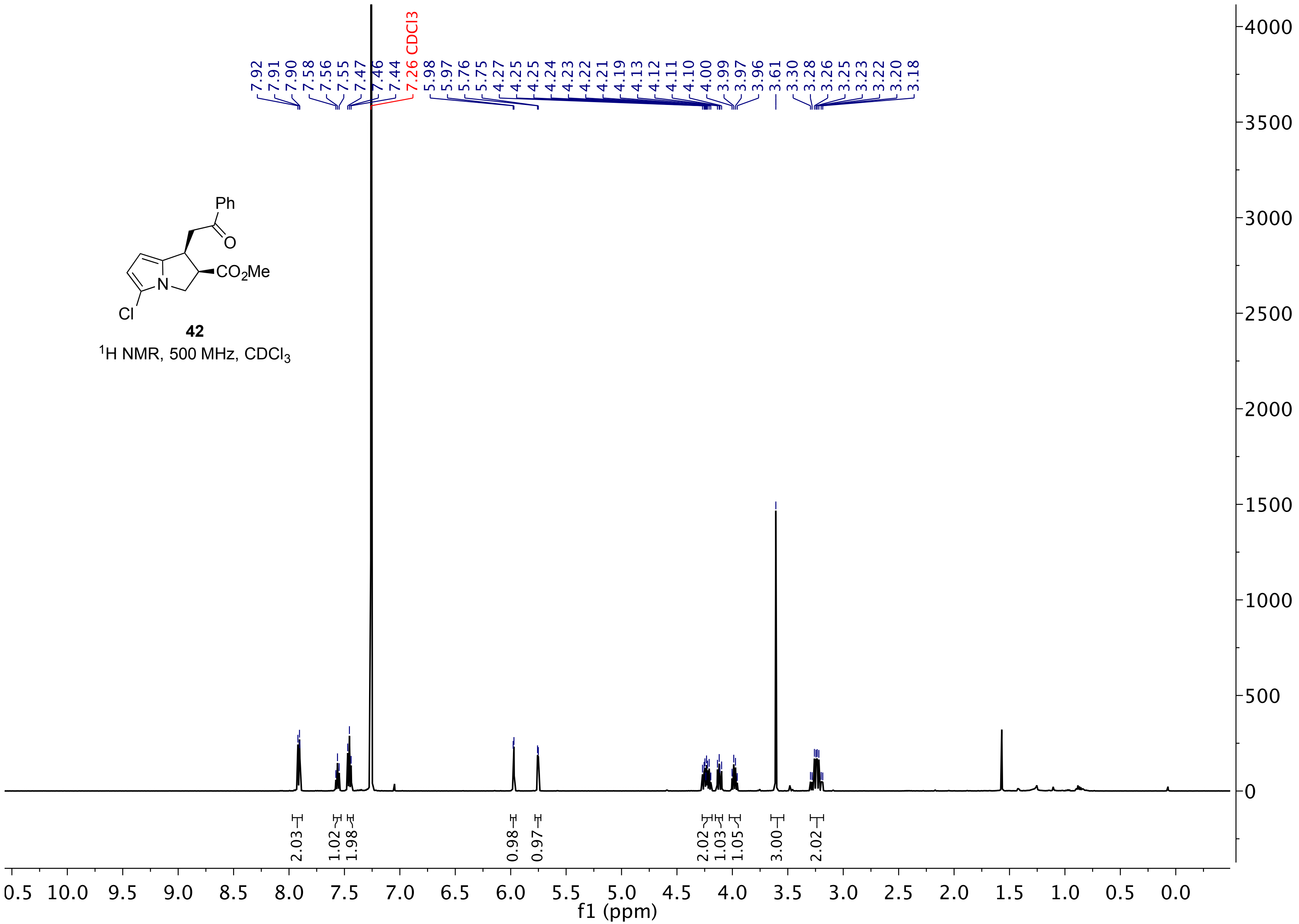
Ph
O
CO2Me
N
Cl
42
1H NMR, 500 MHz, CDCl3
7.92 7.91 7.90 7.58 7.56 7.55 7.47 7.46 7.44 7.26 CDCl3 5.98 5.97 5.76 5.75 4.27 4.25 4.25 4.24 4.23 4.22 4.21 4.19 4.13 4.12 4.11 4.10 4.00 3.99 3.97 3.96 3.61 3.30 3.28 3.26 3.25 3.23 3.22 3.20 3.18
2.03 1.02 1.98 0.98 0.97 2.02 1.03 1.05 3.00 2.02
0.5 10.0 9.5 9.0 8.5 8.0 7.5 7.0 6.5 6.0 5.5 5.0 4.5 4.0 3.5 3.0 2.5 2.0 1.5 1.0 0.5 0.0
f1 (ppm)
4000 3500 3000 2500 2000 1500 1000 500 0

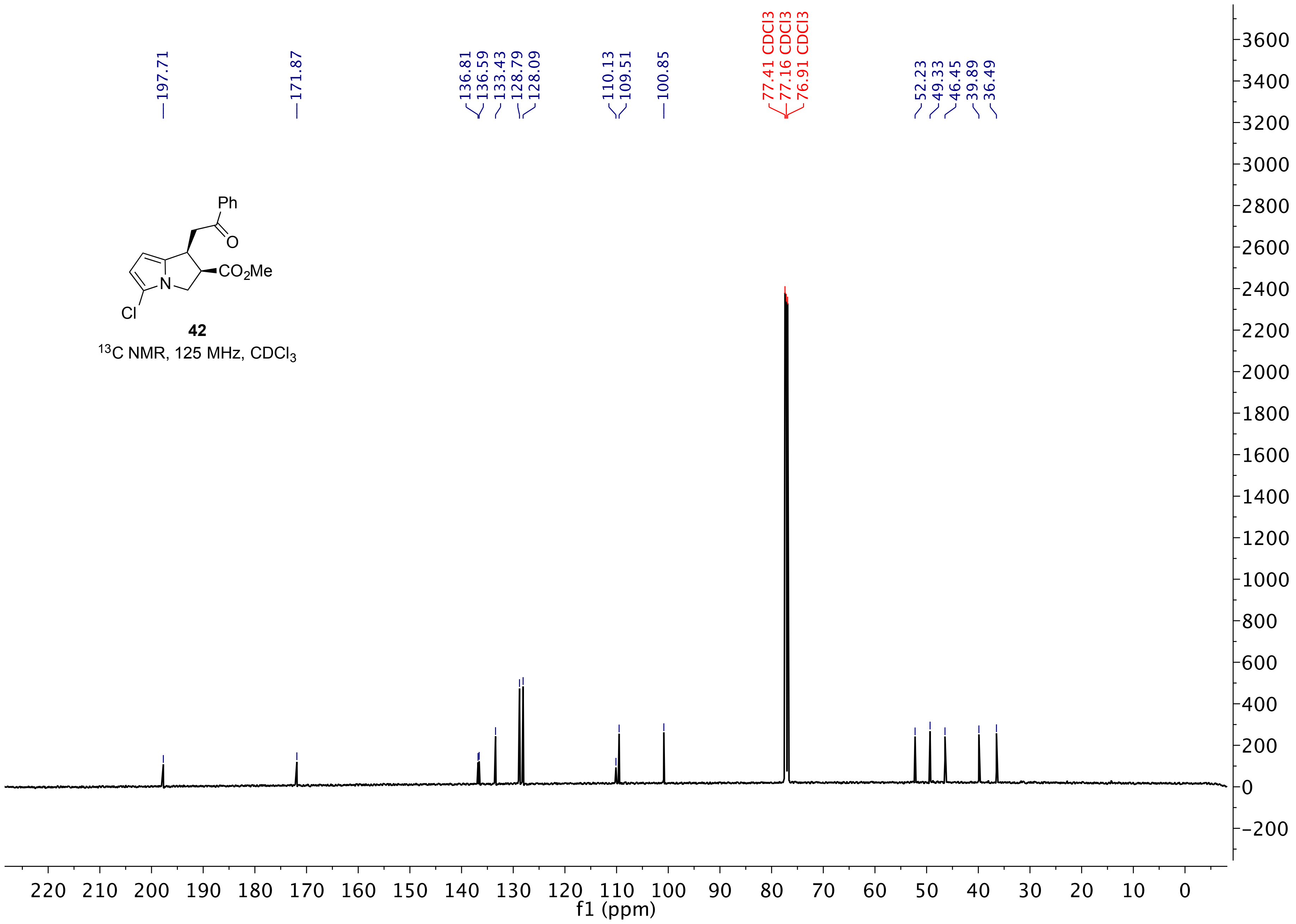
197.71
171.87
136.81
136.59
133.43
128.79
128.09
110.13
109.51
100.85
77.41 CDCl3
77.16 CDCl3
76.91 CDCl3
52.23
49.33
46.45
39.89
36.49
Ph
O
CO2Me
N
Cl
42
13C NMR, 125 MHz, CDCl3
f1 (ppm)

HPLC data for **21**: Chiralpak AD-H (90:10 hexane:IPA, flow rate 1.0 mLmin^{-1}, 270 nm, 30 °C) t_R (4a*S*,9a*R*): 14.3 min, t_R (4a*R*,9a*S*): 16.4 min, >99:1 er.

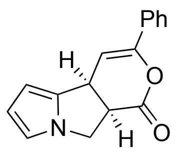

21

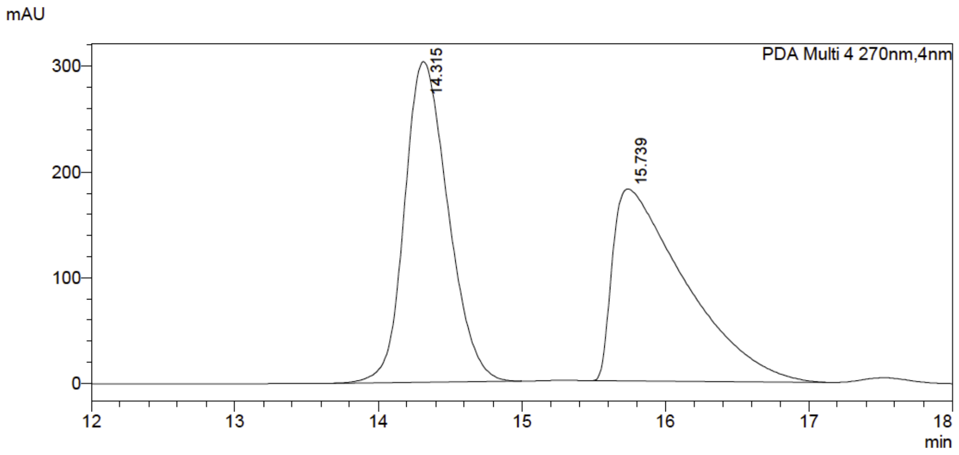

PDA Ch4 270nm

Peak#	Ret. Time	Area%
1	14.315	49.947
2	15.739	50.053
Total		100.000

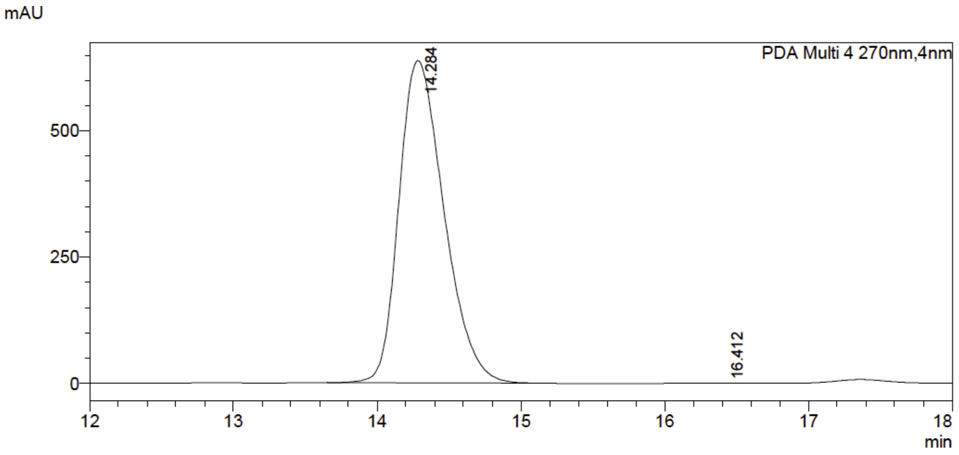

PDA Ch4 270nm

Peak#	Ret. Time	Area%
1	14.284	99.941
2	16.412	0.059
Total		100.000

HPLC data for **27**: Chiralpak AD-H (90:10 hexane:IPA, flow rate 1.0 mLmin^{-1}, 220 nm, 30 °C) t_R (1*S*,2*R*): 12.2 min, >99:1 er.

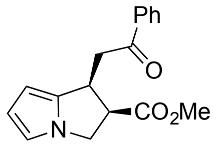

27

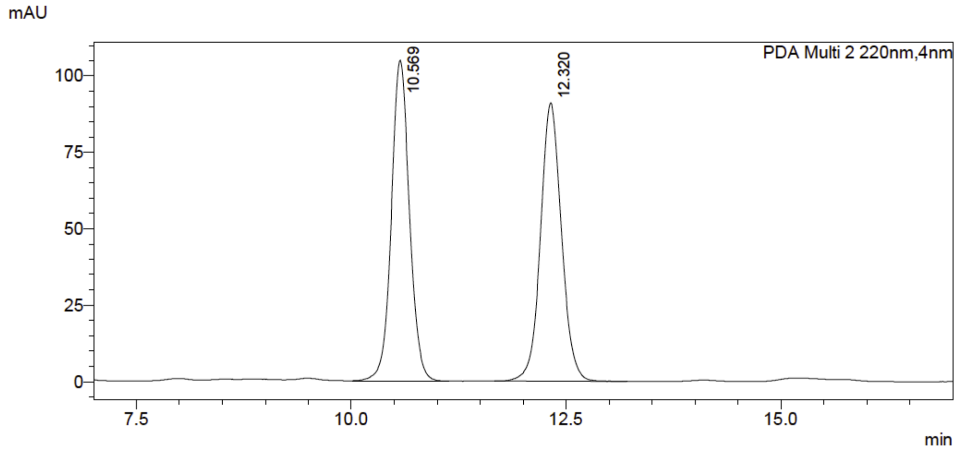

PDA Ch2 220nm

Peak#	Ret. Time	Area%
1	10.569	49.769
2	12.320	50.231
Total		100.000

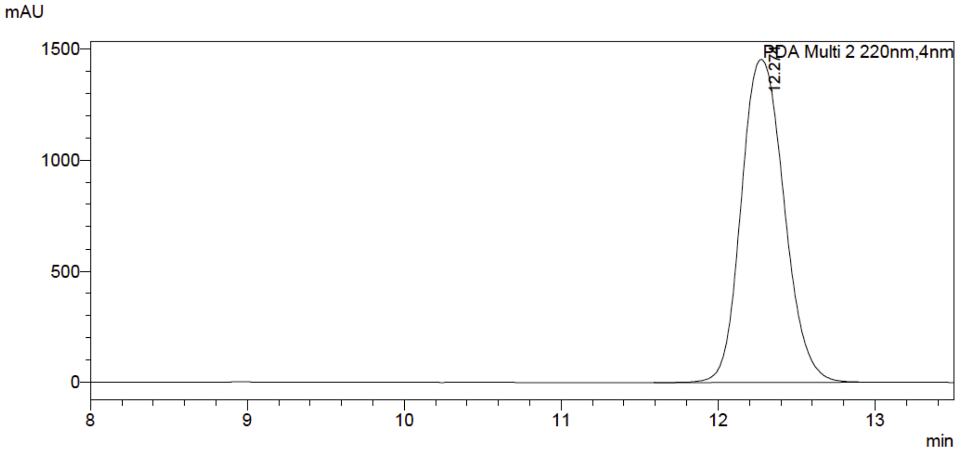

PDA Ch2 220nm

Peak#	Ret. Time	Area%
1	12.274	100.000
Total		100.000

HPLC data for **28**: Chiralpak AD-H (80:20 hexane:IPA, flow rate 1.0 mLmin^{-1}, 254 nm, 30 °C) t_R (1*R*,2*S*): 9.1 min, t_R (1*S*,2*R*): 11.7 min, 98.5:1.5 er.

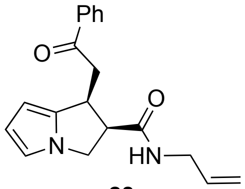

28

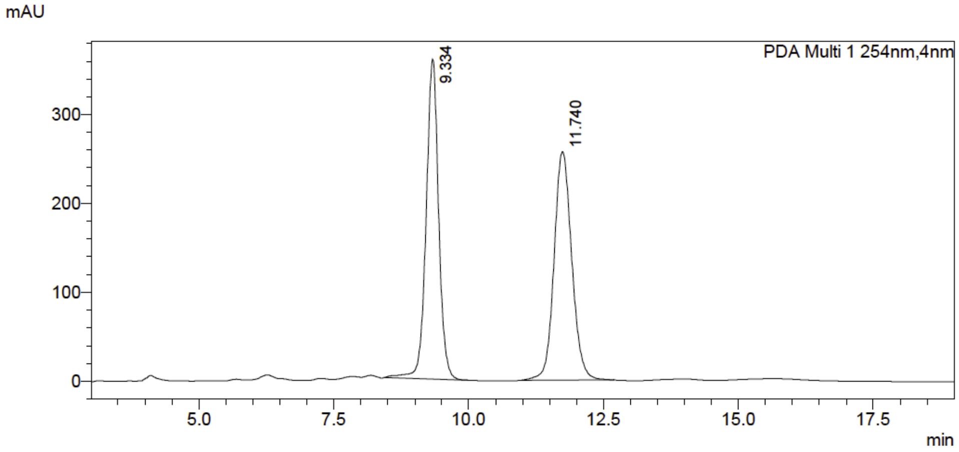

PDA Ch1 254nm

Peak#	Ret. Time	Area%
1	9.334	49.591
2	11.740	50.409
Total		100.000

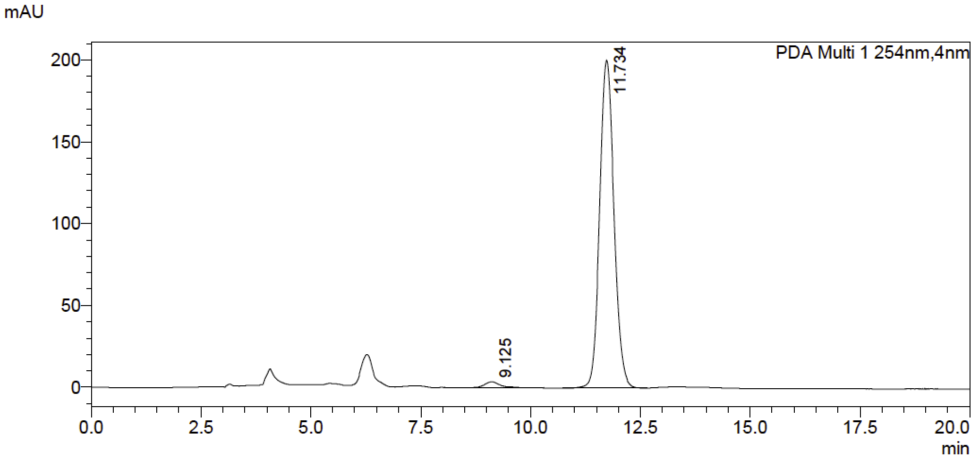

PDA Ch1 254nm

Peak#	Ret. Time	Area%
1	9.125	1.924
2	11.734	98.076
Total		100.000

HPLC data for **29**: Chiralpak IB (90:10 hexane:IPA, flow rate 1.0 mLmin^{-1}, 220 nm, 30 °C) t_R (1*S*,2*R*): 12.6 min, >99:1 er.

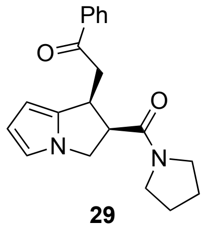

29

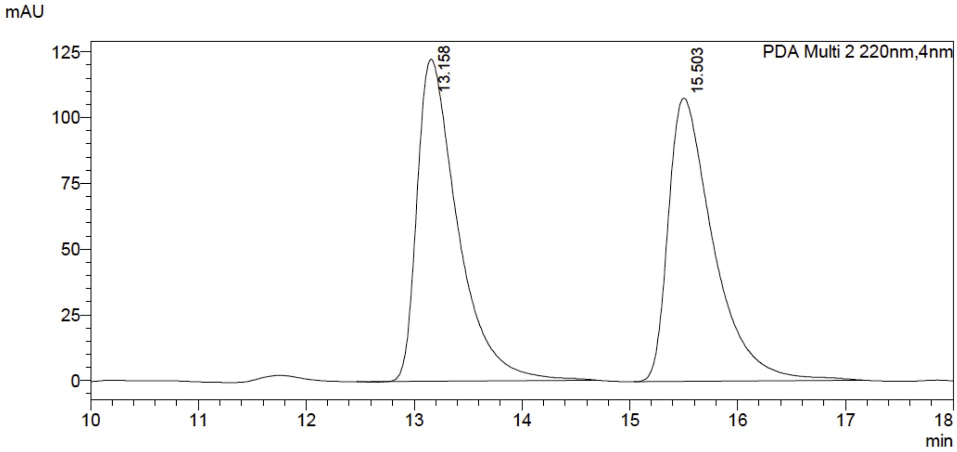

PDA Ch2 220nm

Peak#	Ret. Time	Area%
1	13.158	50.654
2	15.503	49.346
Total		100.000

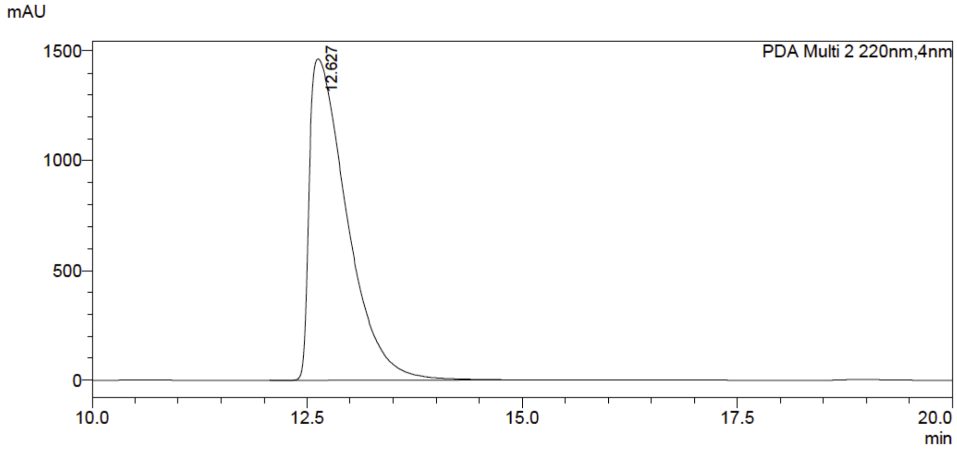

PDA Ch2 220nm

Peak#	Ret. Time	Area%
1	12.627	100.000
Total		100.000

HPLC data for **30**: Chiralpak IA (80:20 hexane:IPA, flow rate 1.0 mLmin^{-1}, 220 nm, 30 °C) t_R (1*S*,2*R*): 13.3 min, >99:1 er.

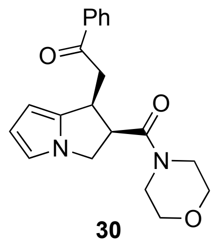

30

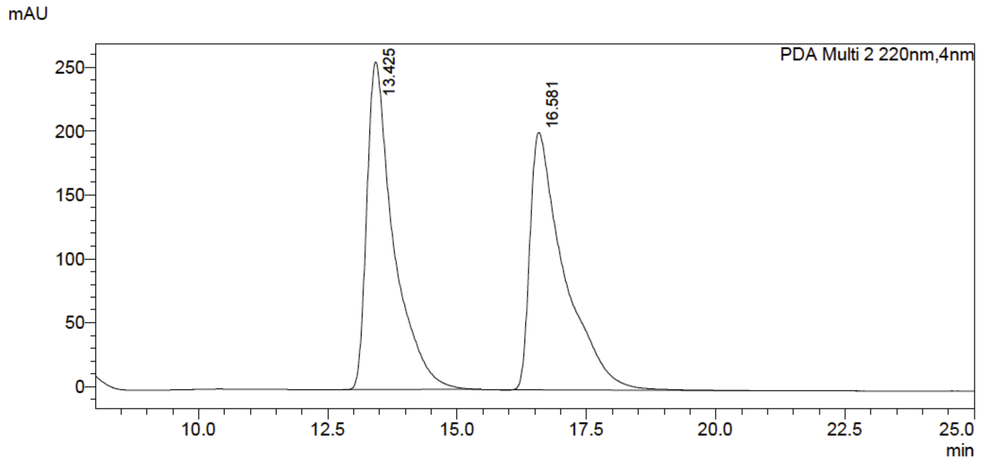

PDA Ch2 220nm

Peak#	Ret. Time	Area%
1	13.425	49.756
2	16.581	50.244
Total		100.000

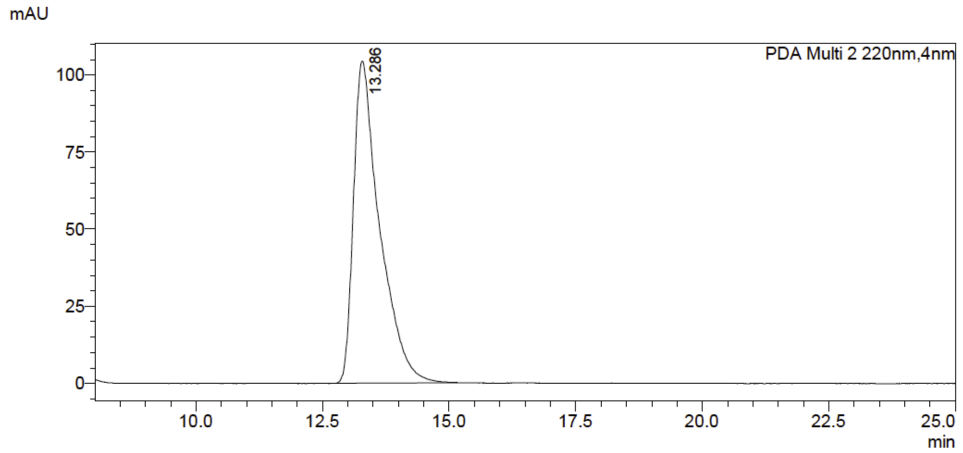

PDA Ch2 220nm

Peak#	Ret. Time	Area%
1	13.286	100.000
Total		100.000

HPLC data for **31**: Chiralpak AD-H (90:10 hexane:IPA, flow rate 1.0 mLmin^{-1}, 211 nm, 30 °C) t_R (1*S*,2*R*): 23.0 min, t_R (1*R*,2*S*): 30.2 min, >99:1 er.

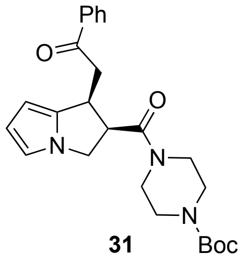

31

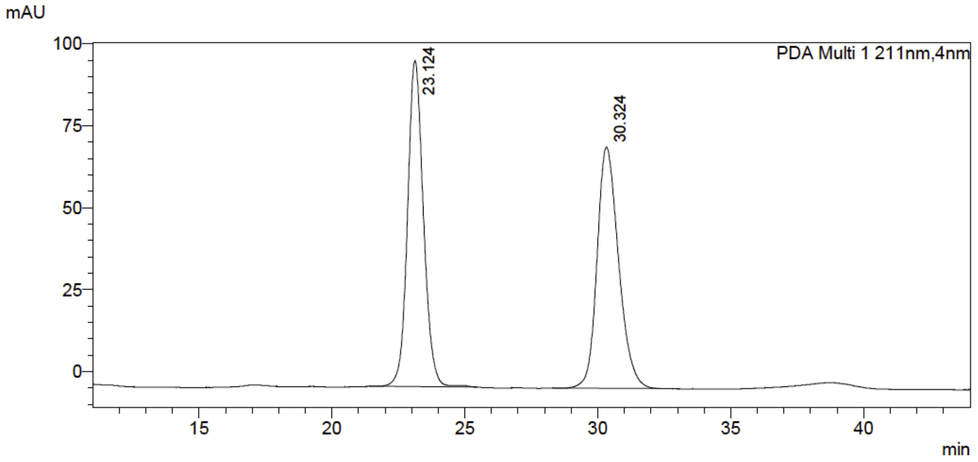

PDA Ch1 211nm

Peak#	Ret. Time	Area%
1	23.124	49.912
2	30.324	50.088
Total		100.000

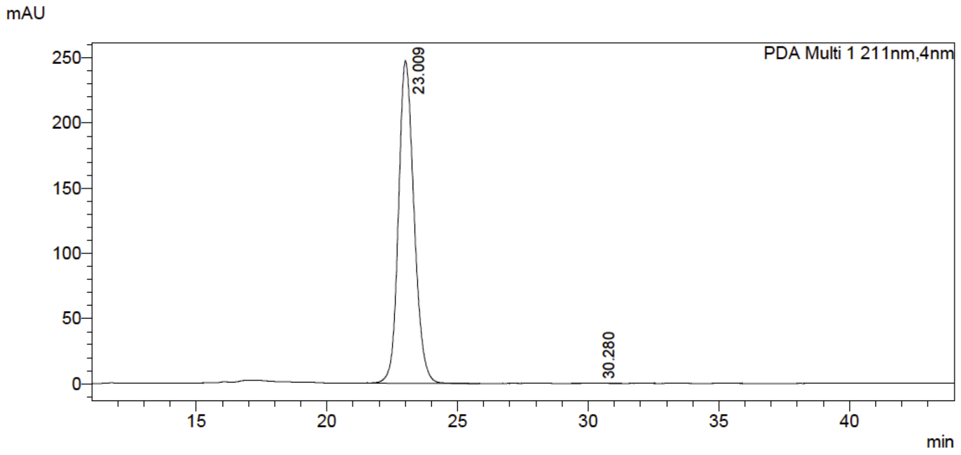

PDA Ch1 211nm

Peak#	Ret. Time	Area%
1	23.009	99.905
2	30.280	0.095
Total		100.000

HPLC data for **32**: Chiralpak AD-H (90:10 hexane:IPA, flow rate 1.0 mLmin^{-1}, 220 nm, 30 °C) t_R (1*S*,2*R*): 35.3 min, t_R (1*R*,2*S*): 46.5 min, >99:1 er.

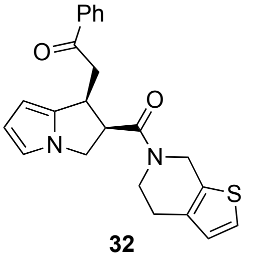

32

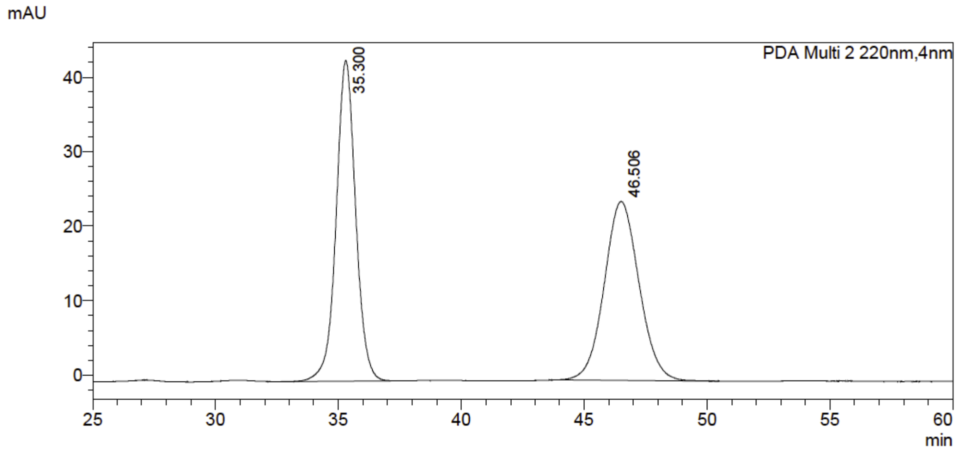

PDA Ch2 220nm

Peak#	Ret. Time	Area%
1	35.300	50.005
2	46.506	49.995
Total		100.000

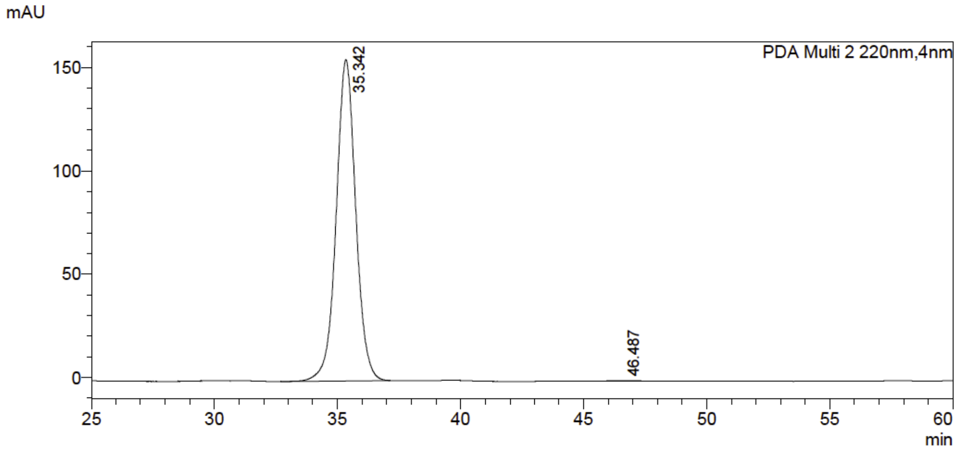

PDA Ch2 220nm

Peak#	Ret. Time	Area%
1	35.342	99.692
2	46.487	0.308
Total		100.000

HPLC data for **33**: Chiralpak AD-H (90:10 hexane:IPA, flow rate 1.0 mLmin^{-1}, 254 nm, 30 °C) t_R (1*R*,2*S*): 14.5 min, t_R (1*S*,2*R*): 17.4 min, >99:1 er.

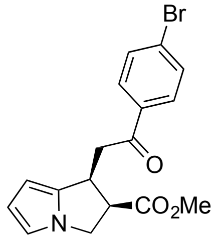

33

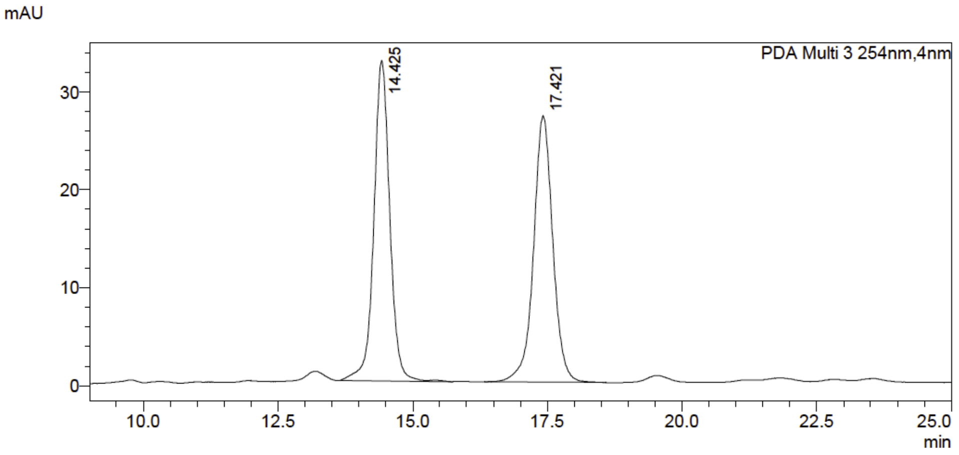

PDA Ch3 254nm

Peak#	Ret. Time	Area%
1	14.425	49.857
2	17.421	50.143
Total		100.000

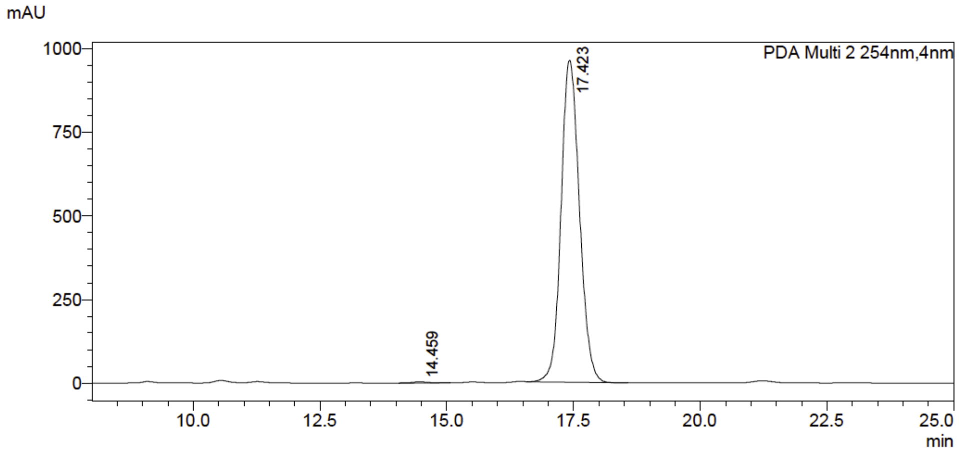

PDA Ch2 254nm

Peak#	Ret. Time	Area%
1	14.459	0.303
2	17.423	99.697
Total		100.000

HPLC data for **34**: Chiralpak OD-H (90:10 hexane:IPA, flow rate 1.0 mLmin^{-1}, 220 nm, 30 °C) t_R (1*S*,2*R*): 17.6 min, >99:1 er.

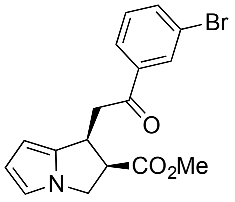

34

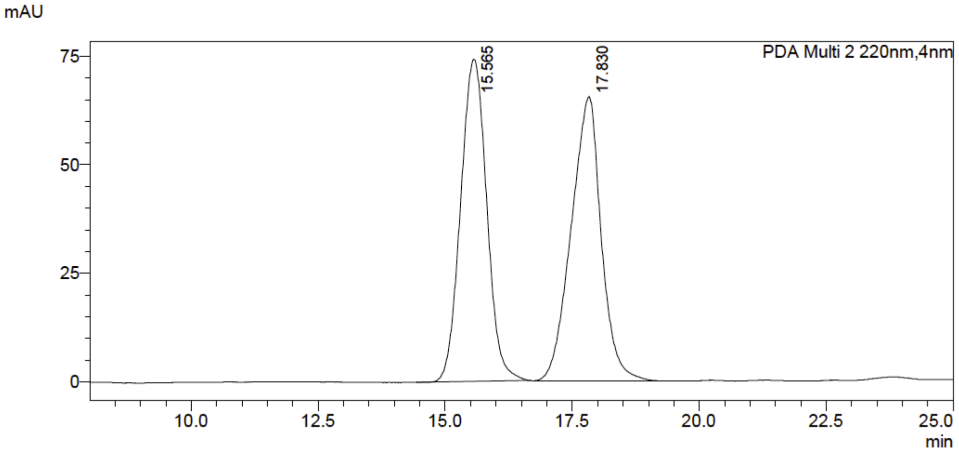

PDA Ch2 220nm

Peak#	Ret. Time	Area%
1	15.565	49.706
2	17.830	50.294
Total		100.000

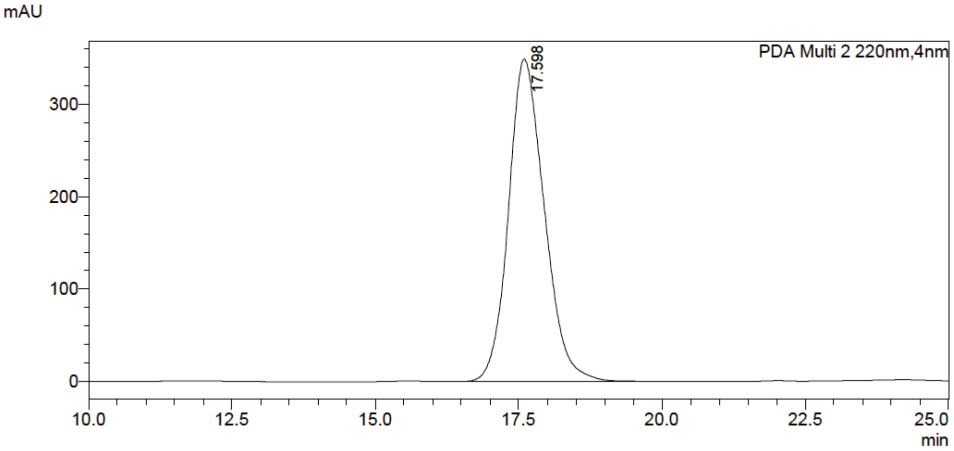

PDA Ch2 220nm

Peak#	Ret. Time	Area%
1	17.598	100.000
Total		100.000

HPLC data for **35**: Chiralpak OD-H (90:10 hexane:IPA, flow rate 1.0 mLmin^{-1}, 270 nm, 30 °C) t_R (1*R*,2*S*): 18.3 min, t_R (1*S*,2*R*): 30.9 min, 99:1 er.

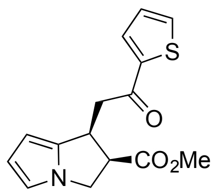

35

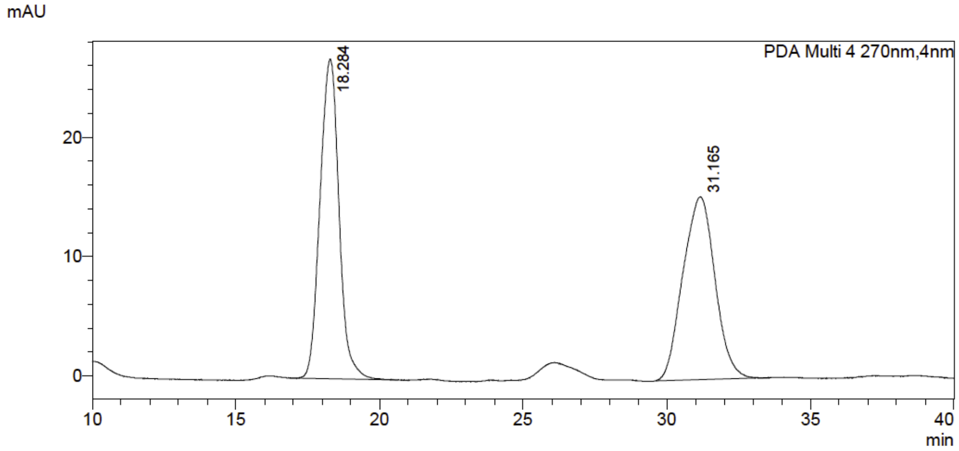

PDA Ch4 270nm

Peak#	Ret. Time	Area%
1	18.284	50.922
2	31.165	49.078
Total		100.000

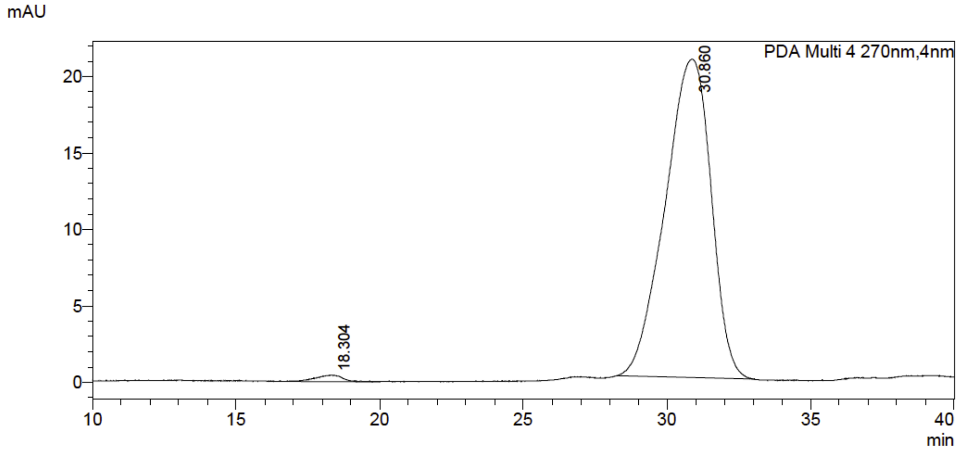

PDA Ch4 270nm

Peak#	Ret. Time	Area%
1	18.304	1.150
2	30.860	98.850
Total		100.000

HPLC data for **36**: Chiralpak AD-H (90:10 hexane:IPA, flow rate 1.0 mLmin^{-1}, 270 nm, 30 °C) t_R (1*R*,2*S*): 20.0 min, t_R (1*S*,2*R*): 25.1 min, >99:1 er.

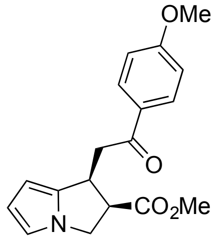

36

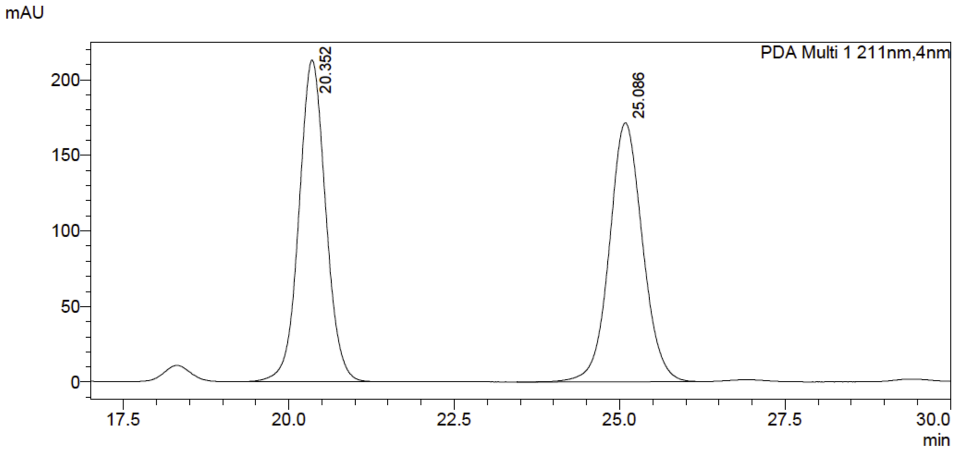

PDA Ch1 211nm

Peak#	Ret. Time	Area%
1	20.352	50.000
2	25.086	50.000
Total		100.000

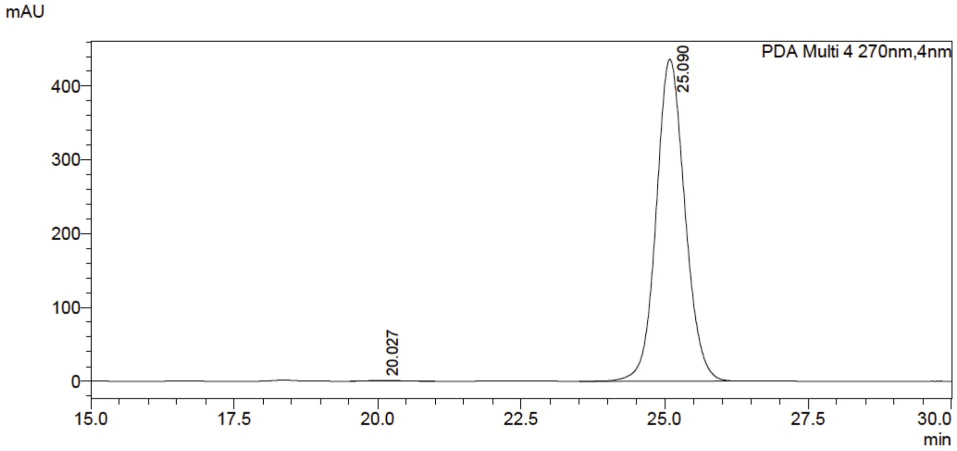

PDA Ch4 270nm

Peak#	Ret. Time	Area%
1	20.027	0.285
2	25.090	99.715
Total		100.000

HPLC data for **37**: Chiralpak OD-H (90:10 hexane:IPA, flow rate 1.0 mLmin^{-1}, 220 nm, 30 °C) t_R (1*R*,2*S*): 20.9 min, t_R (1*S*,2*R*): 27.0 min, >99:1 er.

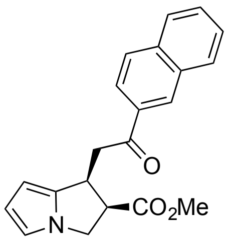

37

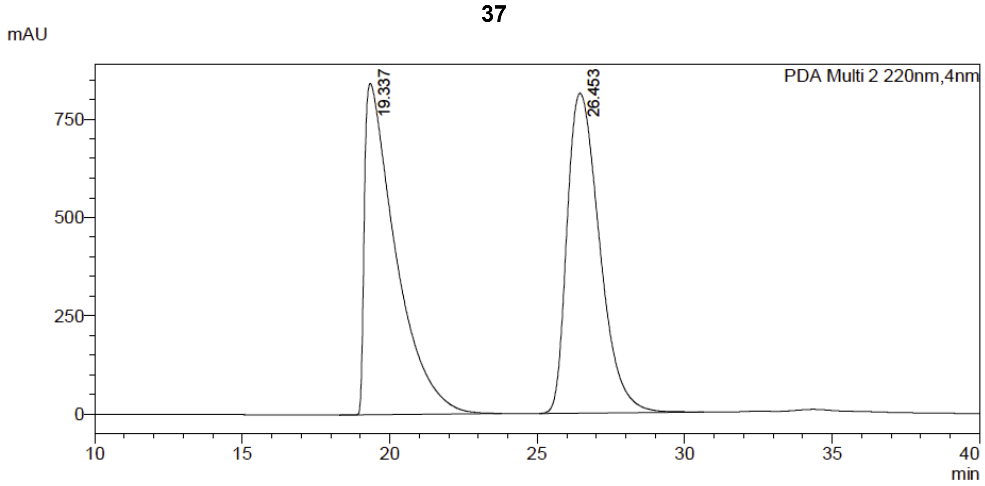

PDA Ch2 220nm

Peak#	Ret. Time	Area%
1	19.337	49.762
2	26.453	50.238
Total		100.000

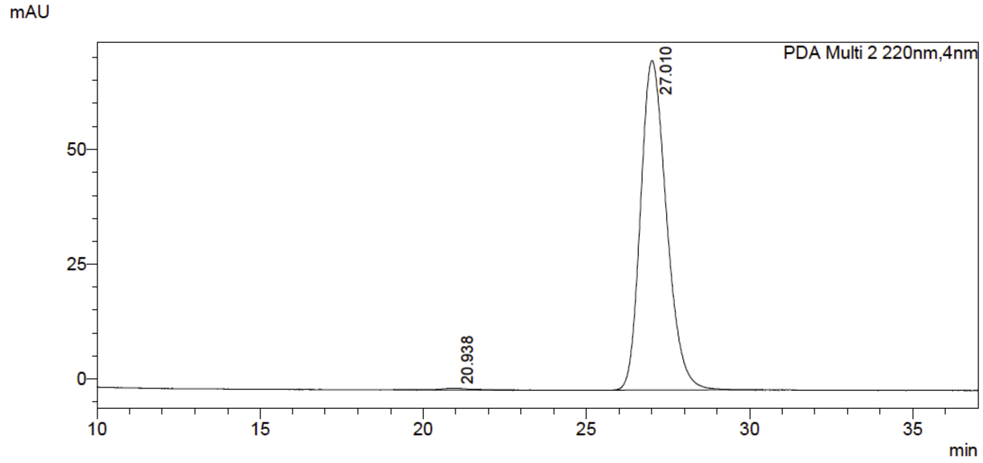

PDA Ch2 220nm

Peak#	Ret. Time	Area%
1	20.938	0.600
2	27.010	99.400
Total		100.000

HPLC data for **38**: Chiralpak AD-H (90:10 hexane:IPA, flow rate 1.0 mLmin^{-1}, 211 nm, 30 °C) t_R (1*R*,2*S*): 12.5 min, t_R (1*S*,2*R*): 14.2 min, >99:1 er.

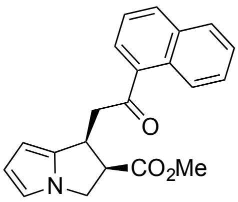

38

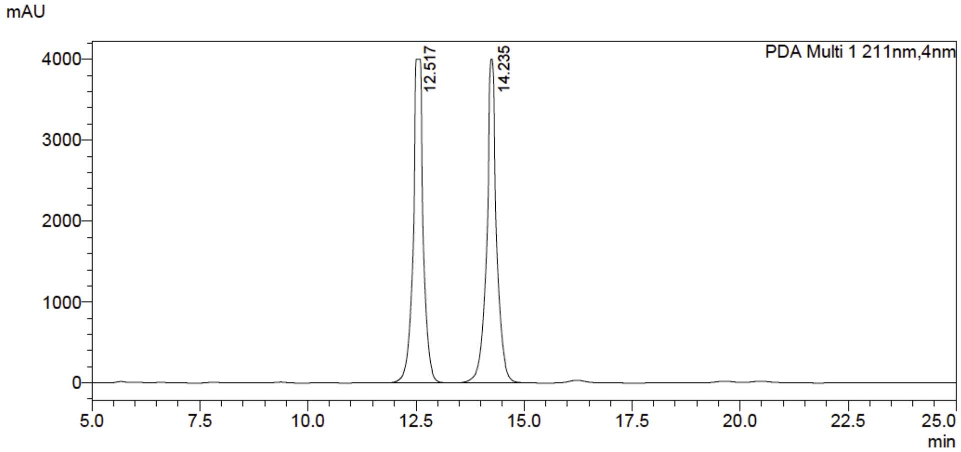

PDA Ch1 211nm

Peak#	Ret. Time	Area%
1	12.517	50.402
2	14.235	49.598
Total		100.000

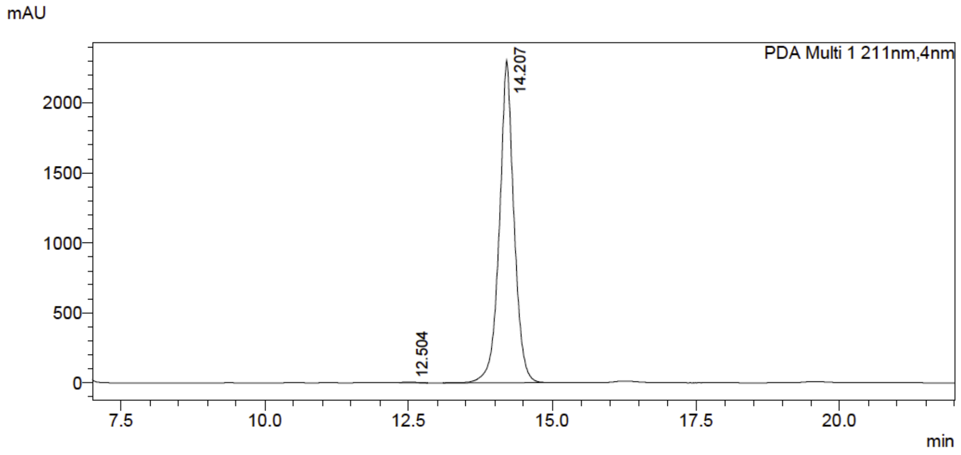

PDA Ch1 211nm

Peak#	Ret. Time	Area%
1	12.504	0.241
2	14.207	99.759
Total		100.000

HPLC data for **39**: Chiralpak OD-H (90:10 hexane:IPA, flow rate 1.0 mLmin^{-1}, 211 nm, 30 °C) t_R (1*S*,2*R*): 21.4 min, >99:1 er.

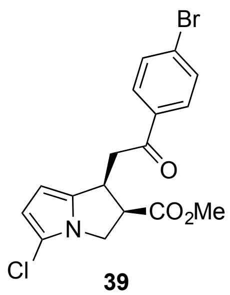

39

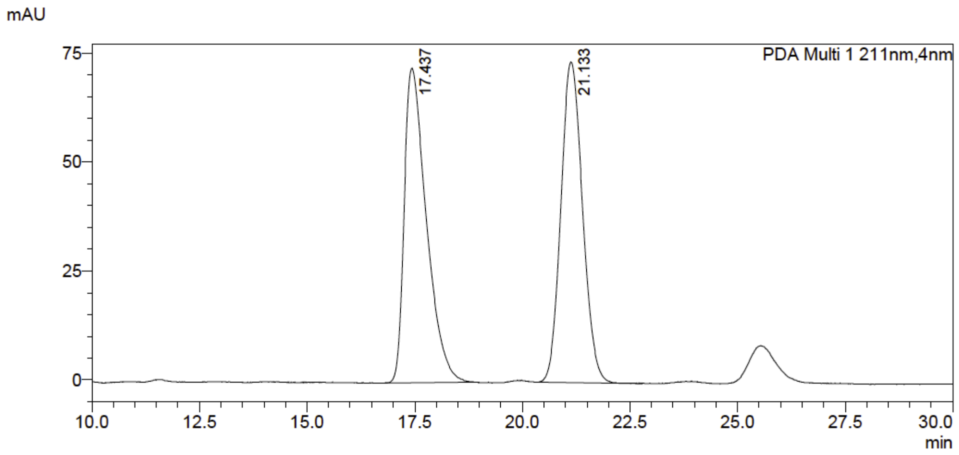

PDA Ch1 211nm

Peak#	Ret. Time	Area%
1	17.437	50.073
2	21.133	49.927
Total		100.000

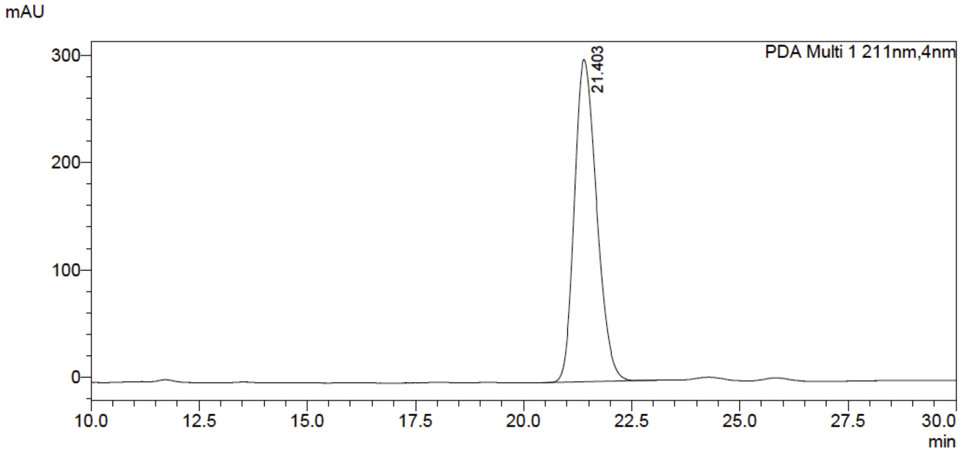

PDA Ch1 211nm

Peak#	Ret. Time	Area%
1	21.403	100.000
Total		100.000

HPLC data for **40**: Chiralpak IC (90:10 hexane:IPA, flow rate 1.0 mLmin^{-1}, 211 nm, 30 °C) t_R (1*S*,2*R*): 14.7 min, t_R (1*R*,2*S*): 17.4 min, >99:1 er.

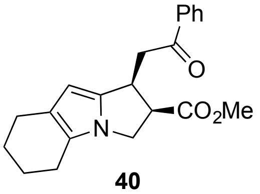

40

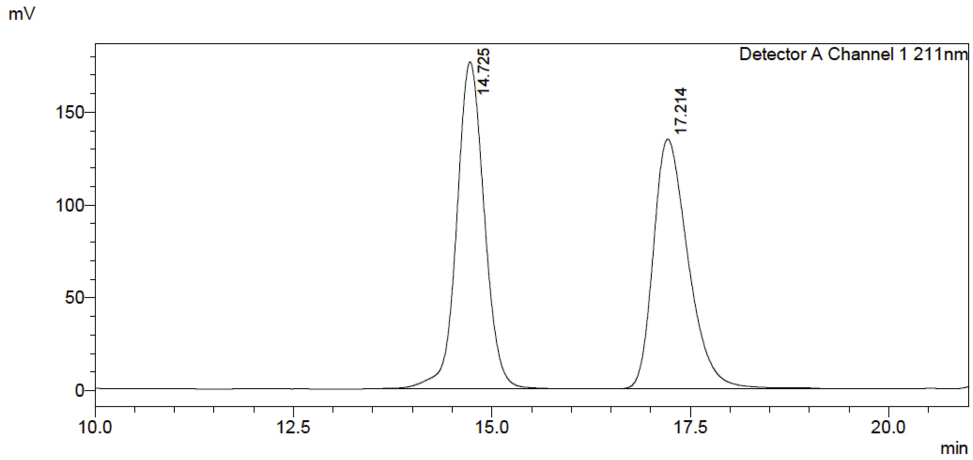

Detector A Channel 1 211nm

Peak#	Ret. Time	Area%
1	14.725	50.880
2	17.214	49.120
Total		100.000

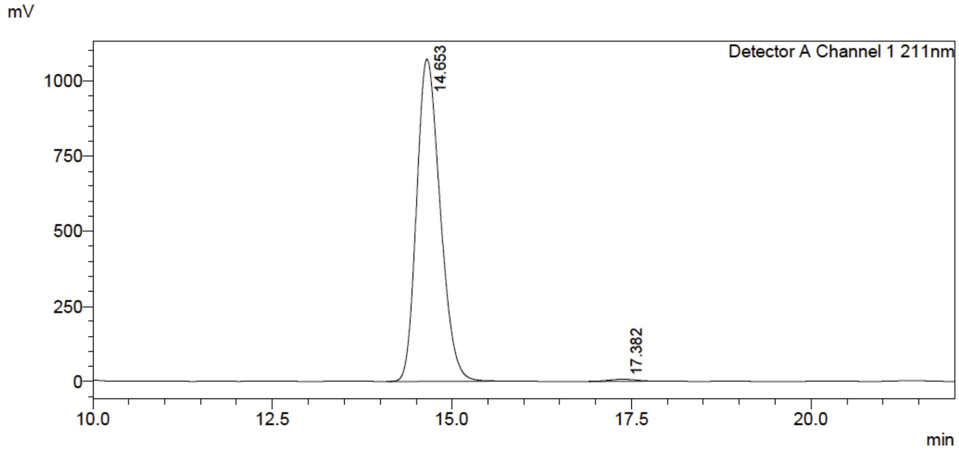

Detector A Channel 1 211nm

Peak#	Ret. Time	Area%
1	14.653	99.210
2	17.382	0.790
Total		100.000

HPLC data for **41**: Chiralpak AD-H (90:10 hexane:IPA, flow rate 1.0 mLmin^{-1}, 254 nm, 30 °C) t_R (1*S*,2*R*): 42.6 min, t_R (1*R*,2*S*): 52.0 min, >99:1 er.

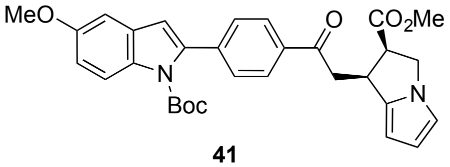

41

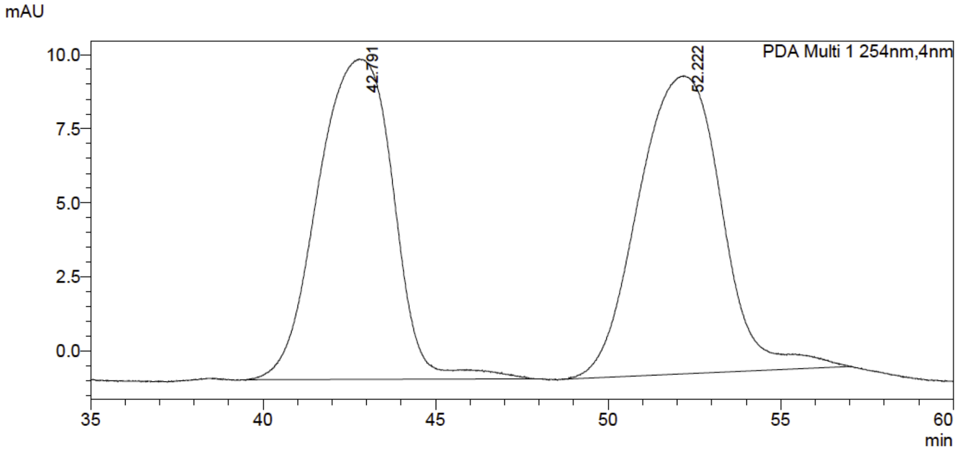

PDA Ch1 254nm

Peak#	Ret. Time	Area%
1	42.791	49.259
2	52.222	50.741
Total		100.000

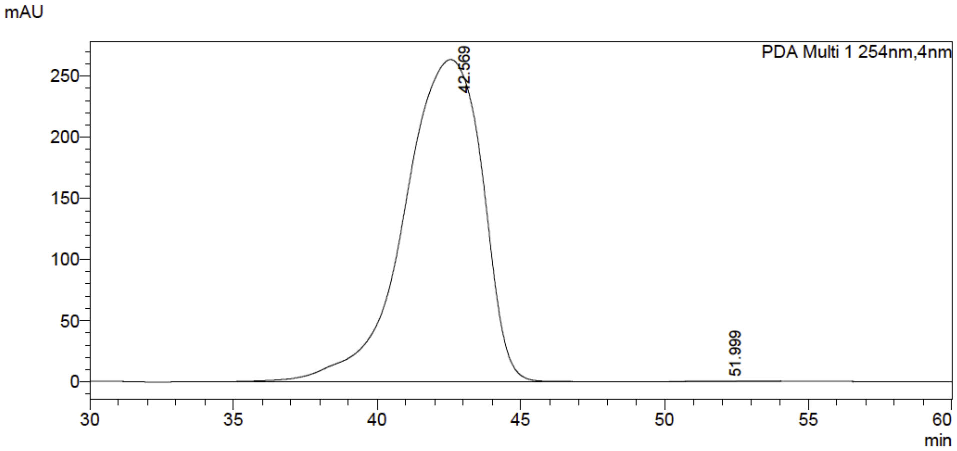

PDA Ch1 254nm

Peak#	Ret. Time	Area%
1	42.569	99.938
2	51.999	0.062
Total		100.000

HPLC data for **42**: Chiralpak OD-H (90:10 hexane:IPA, flow rate 1.0 mLmin^{-1}, 211 nm, 30 °C) t_R (1*R*,2*S*): 24.2 min, t_R (1*S*,2*R*): 39.7 min, >99:1 er.

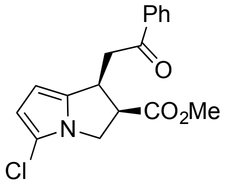

42

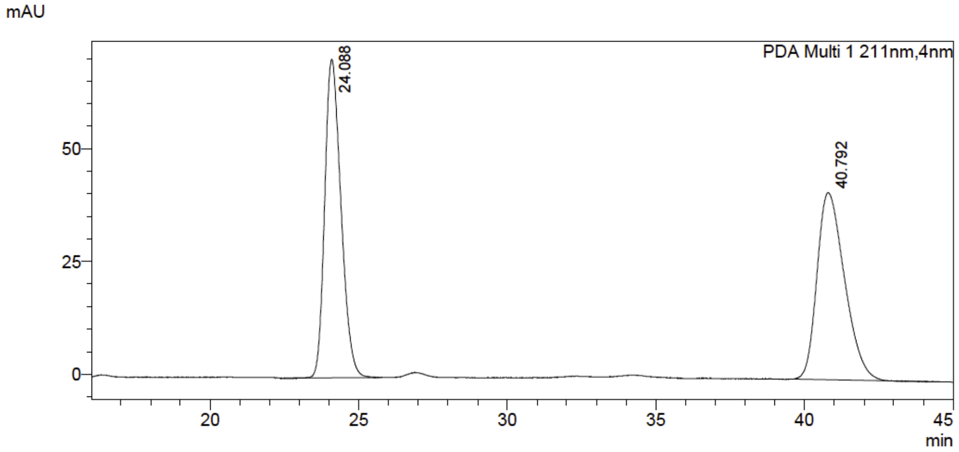

PDA Ch1 211nm

Peak#	Ret. Time	Area%
1	24.088	50.063
2	40.792	49.937
Total		100.000

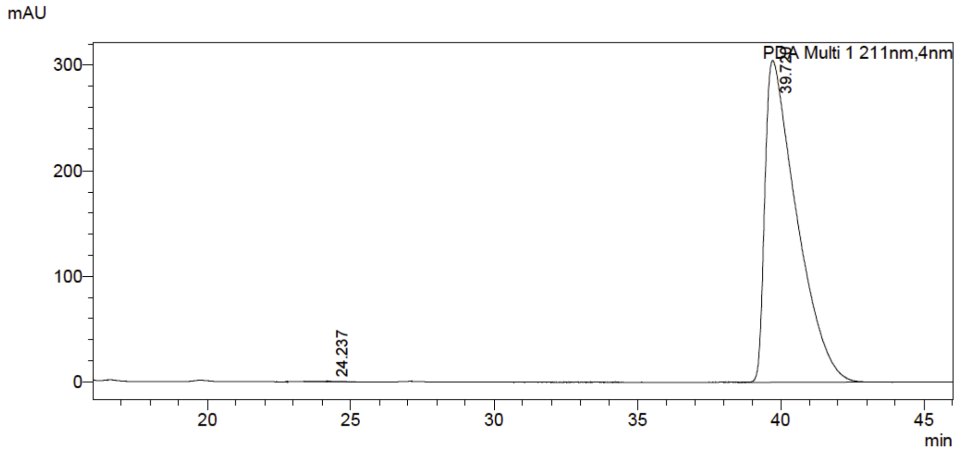

PDA Ch1 211nm

Peak#	Ret. Time	Area%
1	24.237	0.078
2	39.720	99.922
Total		100.000
